# Therapeutic potential of targeting Nrf2 by panobinostat in pituitary neuroendocrine tumors

**DOI:** 10.1186/s40478-024-01775-2

**Published:** 2024-04-18

**Authors:** Yijun Cheng, Yuting Dai, Hao Tang, Xingyu Lu, Jing Xie, Wanqun Xie, Qianqian Zhang, Yanting Liu, Shaojian Lin, Hong Yao, Hanbing Shang, Kun Yang, Hongyi Liu, Xuefeng Wu, Jianming Zhang, Xun Zhang, Li Xue, Zhe Bao Wu

**Affiliations:** 1 Department of Neurosurgery, Center of Pituitary Tumor, Ruijin Hospital, Shanghai Jiao Tong University School of Medicine, 197# Ruijin er road, Shanghai, 200025 China; 2 Shanghai Institute of Hematology, State Key Laboratory of Medical Genomics, National Research Center for Translational Medicine, Ruijin Hospital, Shanghai Jiao Tong University School of Medicine, Shanghai, China; 3 Department of Pathology, Ruijin Hospital, Shanghai Jiao Tong University School of Medicine, Shanghai, China; 4 Department of Neurosurgery, Xinhua Hospital, Shanghai Jiao Tong University School of Medicine, Shanghai, China; 5 National Research Center for Translational Medicine (Shanghai), State Key Laboratory of Medical Genomics, Ruijin Hospital, Shanghai Jiao Tong University School of Medicine, Shanghai, China; 6 Department of Neurosurgery, Nanjing Brain Hospital, Nanjing Medical University, Nanjing, China; 7 Center for Immune-Related DiseasesShanghai Institute of Immunology, Ruijin Hospital, Shanghai Jiao Tong University School of Medicine, Shanghai, China; 8 Neuroendocrine Research Laboratory, Massachusetts General Hospital and Harvard Medical School, Boston, MA USA; 9 Department of Neurosurgery, Center for Immune-Related Diseases at Shanghai Institute of Immunology, Ruijin Hospital, Shanghai Jiao Tong University School of Medicine, Shanghai, China

**Keywords:** PitNETs, High-throughput screening, HDACIs, Panobinostat, Nrf2

## Abstract

We aimed to identify the druggable cell-intrinsic vulnerabilities and target-based drug therapies for PitNETs using the high-throughput drug screening (HTS) and genomic sequencing methods. We examined 9 patient-derived PitNET primary cells in HTS. Based on the screening results, the potential target genes were analyzed with genomic sequencing from a total of 180 PitNETs. We identified and verified one of the most potentially effective drugs, which targeted the Histone deacetylases (HDACs) both in in vitro and in vivo PitNET models. Further RNA sequencing revealed underlying molecular mechanisms following treatment with the representative HDACs inhibitor, Panobinostat. The HTS generated a total of 20,736 single-agent dose responses which were enriched among multiple inhibitors for various oncogenic targets, including HDACs, PI3K, mTOR, and proteasome. Among these drugs, HDAC inhibitors (HDACIs) were, on average, the most potent drug class. Further studies using in vitro, in vivo, and isolated PitNET primary cell models validated HDACIs, especially Panobinostat, as a promising therapeutic agent. Transcriptional surveys revealed substantial alterations to the Nrf2 signaling following Panobinostat treatment. Moreover, Nrf2 is highly expressed in PitNETs. The combination of Panobinostat and Nrf2 inhibitor ML385 had a synergistic effect on PitNET suppression. The current study revealed a class of effective anti-PitNET drugs, HDACIs, based on the HTS and genomic sequencing. One of the representative compounds, Panobinostat, may be a potential drug for PitNET treatment via Nrf2-mediated redox modulation. Combination of Panobinostat and ML385 further enhance the effectiveness for PitNET treatment.

**Supplementary Information:**

The online version contains supplementary material available at 10.1186/s40478-024-01775-2.

## Introduction

Pituitary neuroendocrine tumors (PitNETs) are the second-most common intracranial tumors in humans, accounting for about 10–25% of all the primary tumors in the brain [1]. These disorders have a prevalence rate of approximately 17% in the general population [2]. The clinical symptoms of PitNETs mainly arise from the tumor mass effects and abnormal hormone productions. The former mainly includes headache and vision loss, while the latter includes various hormones-related disorders [3].

Medical treatment plays an increasing role in the management of PitNETs, either as the first-line or adjuvant treatment. A growing awareness regarding all molecular mechanisms involved in both tumor shrinkage and hormonal control might allow for the development of novel drugs for sole medical treatment of PitNETs, especially macroadenomas, rather than surgical therapy [4]. Currently, only dopamine receptors (DR) and somatostatin receptors (SSTR) have been well recognized as effective targets in the pharmacological therapy for some specific subtypes of PitNETs. Even so, the overall prevalence of drug resistance to the stable analogues of dopamine and somatostatin in prolactinoma and acromegaly can be up to 25% and 75% [5, 6], respectively. There are still no effectively therapeutic targets and corresponding drugs for these resistant and other subtypes of PitNETs. A full appreciation of the druggable landscape in PitNETs is still lacking. Thus, finding potentially new therapeutic targets and corresponding drug therapy are urgent unmet needs.

Histone deacetylases (HDACs) are traditionally identified as transcriptional repressors, and human genome-wide mapping of HDAC binding has revealed that it can be localized on the chromatin of different active genes [7]. Therefore, HDACs can be recruited to various genes, where they may be involved in additional co-transcriptional, post-transcriptional, or elongation events, thereby regulating the expression and action of different histones and non-histone proteins [8]. HDACs has been proven to be closely associated with the tumorigenesis in different types of tumors. Thus, compounds targeting HDACs, especially HDAC inhibitors (HDACIs) have attracted significant attention as anti-cancer drugs in various kinds of solid and hematologic cancers [9, 10]. However, the role of HDACs and its correspondingly broad spectrum of inhibitors in PitNETs have not yet been fully exploited.

The development of high-throughput drug screening (HTS) technologies has enabled researchers to identify new drugs targeted to the traditional tumors. In the present study, an unbiased HTS and genomic sequencing were employed to explore the druggable cell-intrinsic vulnerabilities in PitNETs and further identify the target-based drug therapies for PitNETs.

## Methods

### Patients and tissue samples

This study was approved by the Ethical Review Board of Ruijin Hospital, Shanghai Jiao Tong University School of Medicine. All Patients diagnosed as PitNETs were given the informed consent according to the institutional guidelines. For the HTS experiments, a total of 9 fresh specimen were collected at the surgeries from PitNET patients whose detailed clinical data were presented in Additional file 1: Table S1. Notably, all these 9 PitNETs were macroadenoma. Moreover, 6 normal pituitary tissues were obtained from autopsy. In addition, another 180 PitNET tissues were collected for RNA sequencing (RNA-seq) as previously described [11].

### High-throughput drug screening

The HTS customized library (Selleck Chemical, Houston, TX, USA) contains a total of 2097 FDA-approved drugs and bio-active targeted compounds. The detailed data of these compounds have been listed in Additional file 1: Table S2. Nine primary cells derived from PitNET patients (Additional file 1: Table S1) and 3 pituitary adenoma cell lines (MMQ, GH3, and AtT-20) were seeded in a density of about 1,200 cells per well in 384-well plates with 50 μL medium by automated cell seeding and then incubated overnight at 5% CO_2_ and 37 °C. Twenty-four hours later, cells were treated with either DMSO or one of the compounds at a final concentration of 2 μM. These plates were incubated for another 72 h. The cell viability was measured by CellTiter 286 Aqueous One Solution (Promega), and comparisons were made between the drug-treated cells and the control (DMSO-treated) cells. Compounds reducing cell viability compared with the control were considered screen hits.

### RNA sequencing alignment and analysis

For primary cells and PitNET specimen analysis, raw FASTQ files of RNA sequencing were aligned to human reference genome GRCh38 (release 40). The human reference genome and its annotation file were downloaded from GENCODE database (https://www.gencodegenes.org/). Sample preparation, RNA-seq analysis, and data analysis are described in the Supplementary Methods.

### Statistical analysis

The SPSS 25.0S software (SPSS Inc, Chicago, USA) and Graphpad Prism 8.0 (GraphPad Software, San Diego, CA, USA) were used to analyze the results in the study. The statistical comparisons were analyzed with the one-way ANOVA followed by the Student–Newman–Keuls. Differences between 2 groups were compared with the Student’s *t*-test. The immunoreactive scores were analyzed using the Kruskal–Wallis H test. All quantitative data were expressed as the mean ± SD. *p* < 0.05 was considered a statistically significant difference.

### Experimental assays and analyses

All cell culture, reagents, and experimental assays, including RNA-seq alignment and analysis, cell transfection, cell viability, Colony formation, Flow cytometry analysis, RT-PCR, Western blot analysis, Xenograft model, Cell counting in bronchoalveolar lavage fluid (BALF), Immunofluorescence (IF) and Immunohistochemistry (IHC) staining, Terminal Deoxynucleotidyl Transferase-Mediated dUTP Nick 3′-End Labeling (TUNEL), Luminol and lucigenin Chemiluminescence (CL) assays, Malondialdehyde (MDA)assay, total superoxide dismutase (SOD) activity assay, Nrf2 DNA-binding assay, and ELISA, are described in the Supplementary Methods.

## Results

### HTS revealed classes of mechanistic vulnerabilities of PitNETs

**Fig. 1:**
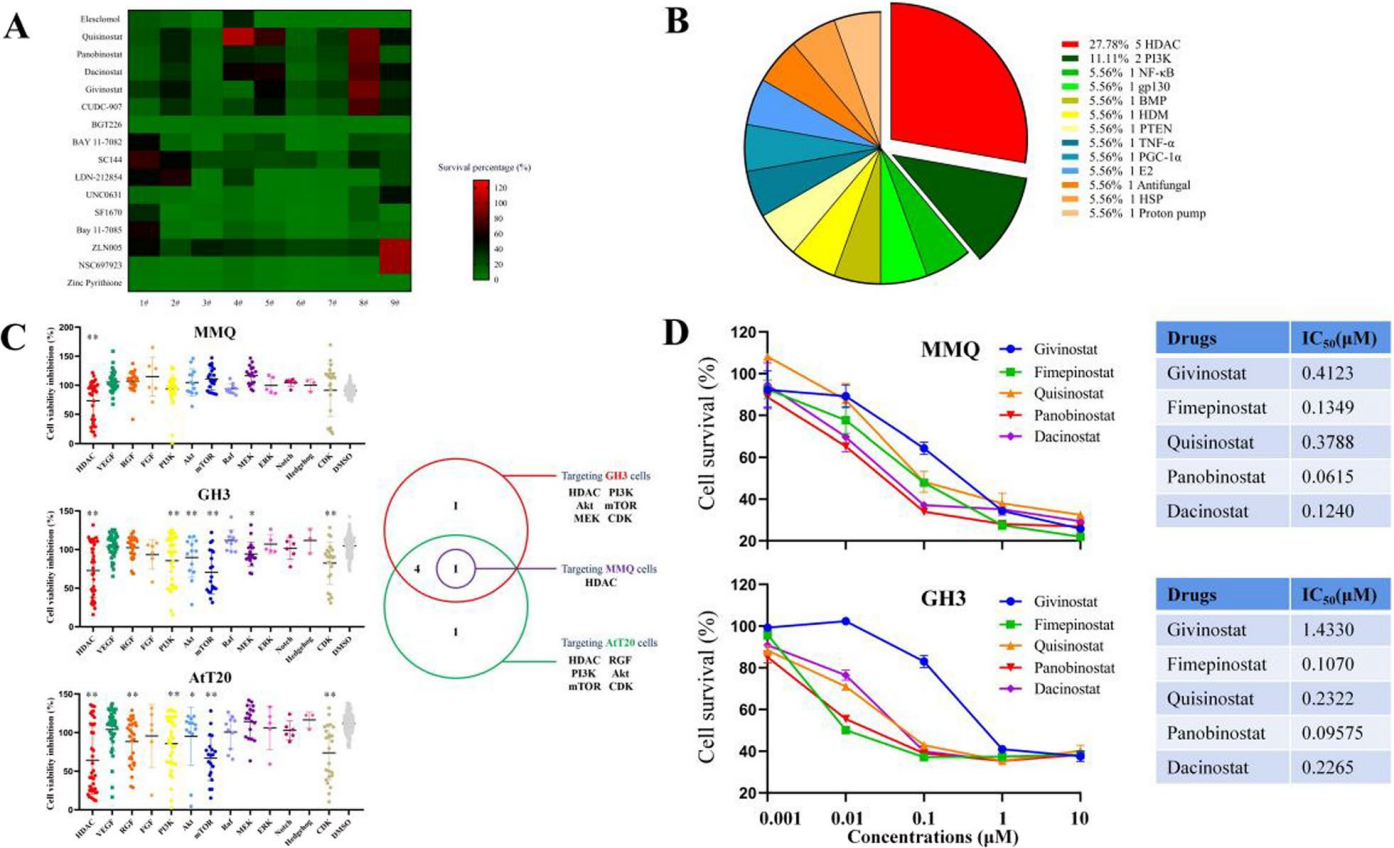
Identification of drug candidates through high-throughput drug screening in PitNETs. **A** Heat-map representation of drug activities for multiple PitNET cells screened through the HTS library. The gradual shift of color from green to red indicates the primary cell survival percentage from small to large. **B** Mechanistic drug classes enriched among the 16 ‘hits’ selected based on consistent potency across multiple PitNET primary cell cultures. **C** Left: An overview of growth inhibition of 3 pituitary adenoma cell lines by compounds targeting various MoAs. Right: A Venn diagram of the left results. **D** The cell survival of MMQ and GH3 cells for selected agents from the key enriched mechanistic classes HDACs

To screen the potentially effective drugs for PitNETs, we used a panel of 9 patient-derived PitNET primary cell cultures (Additional file 1: Table S1) for screening. Among these agents are different inhibitors for well-known oncogenic targets, such as Phosphoinositide-3-kinase (PI3K), mTOR, proteasome, HDACs, and mitogen activated protein kinase kinase (MEK), covering over 181 distinct mechanisms of actions (MoAs, Additional file 1: Table S3). Based on the reduction of cell viability, the screening generated 20,736 single-agent dose response signatures, resulting in a coherent database of PitNET vulnerabilities. Agents inhibiting at least 66.67% (6/9) patient-derived PitNET primary cell cultures by 50% were classified as ‘hits’. A total of 16 hits were identified (Fig. 1A, Additional file 1: Table S4). As shown in Fig. 1B, a total of 13 MoAs relevant to PitNET pathogenesis were enriched among the screened hits, including HDACs, PI3K, NF-*κ*B, gp130, and others (Additional file 1: Table S5). Agents from these mechanistic classes demonstrated a relatively wide potency range, with HDACs inhibitors being the most potent drug class. Furthermore, we assessed the efficacy of agents targeting previously reported therapeutic MoAs to PitNETs [12], including HDAC, VEGF, RGF, FGF, PI3K, Akt, mTOR, Raf, MEK, ERK, Notch, Hedgehog, and CDK in MMQ, GH3, and ATt-20 cell lines using the HTS Library. As shown in Fig. 1C, Only HDAC was the therapeutic target for all three pituitary adenoma cell lines (*p* < 0.05).

To verify the anti-tumor effects of HDACIs on PitNETs, the top 5 HDACIs, including Panobinostat, Quisinostat, Givinostat, Dacinostat, and Fimepinostat (CUDC-907), were used on GH3 and MMQ cells. Consistent with the HTS data, all these HDACIs strongly inhibited GH3 and MMQ cell proliferation at low concentrations, with IC_50_ values in the nanomolar ranges (Fig. 1D). Notably, Panobinostat displayed the lowest IC_50_ values for both MMQ (0.0615 μM) and GH3 (0.09575 μM) cell lines at 24 h, therefore it was chosen for further functional and mechanistic studies.

### Panobinostat conferred anti-PitNET effects both in vivo and in vitro

**Fig. 2:**
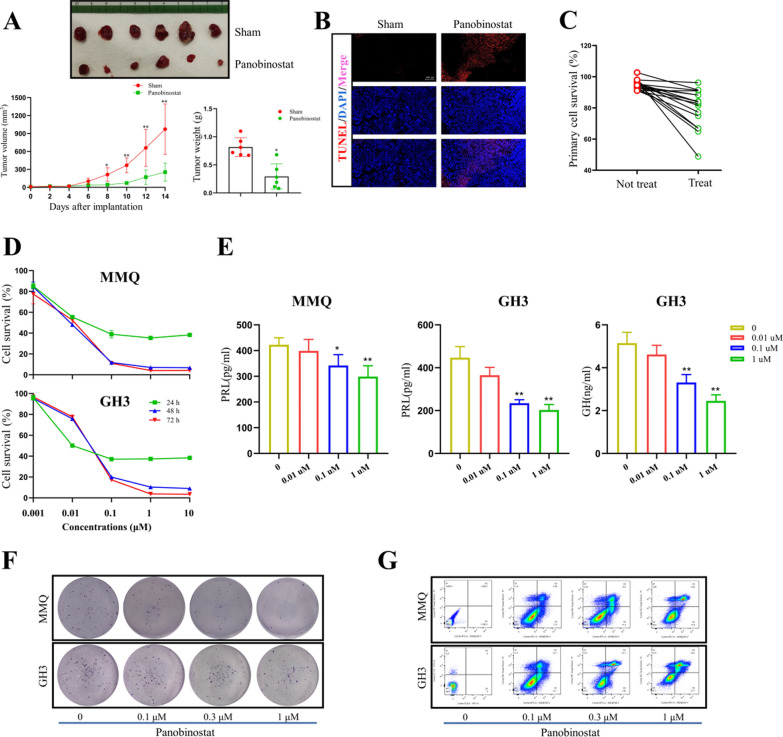
Panobinostat demonstrated therapeutic efficacy in PitNETs. **A** Upper: Representative images of xenograft tumors from mice treated with control vehicle or Panobinostat. Down: Tumor volume growth curves and tumor weights in different treatment groups. n = 6; *p < 0.05, **p < 0.01. **B** Representative images of TUNEL staining from xenograft tumors. **C** The inhibitory responses of 18 primary PitNET cell proliferation by Panobinostat. **D** The cell survival of MMQ and GH3 cells after Panobinostat treatment at different dosage and time points. **E** The levels of PRL and GH secretions after Panobinostat treatment at different dosage at 24 h. n = 3; *p < 0.05, **p < 0.01. **F** Representation of colony formations in MMQ and GH3 cell lines. **G** Representation of Anneix staining in MMQ and GH3 cell lines

To further verify the anti-tumor effects of Panobinostat in PitNETs, we first establish xenograft mouse model by implanting GH3 cells in the flank of nude mice. As shown in Fig. 2A, Panobinostat markedly inhibited the xenograft tumor sizes and weights (*p* < 0.05). In vivo TUNEL staining assays revealed that Panobinostat induced obvious cell apoptosis in xenograft tumors (Fig. 2B). Although it has been reported that Panobinostat has adverse side effects, mainly including diarrhea, pneumonia, and neurotoxicity [13]. Herein, we did not observe obvious mice diarrhea symptoms during the experiment. Moreover, Panobinostat did not affect the body weight, induced pneumonia or neurotoxicity of the mice significantly (all *p* > 0.05, Additional file 1: Figure S1A–E).

To test whether Panobinostat may inhibit human PitNET growth, we cultured the primary tumor cells from 18 PitNET patients, including PIT1-lineage (n = 7), TPIT-lineage (n = 1), SF1-lineage (n = 8), No distinct cell lineage (n = 2) (Additional file 1: Table S1). As shown in Fig. 2C, the growth of 13 of the 18 primary PitNET cultures was suppressed by 1 μM Panobinostat at 24 h, showing a total effective rate was 72.22%.

We next used two rat pituitary tumor cell lines, MMQ and GH3, to evaluate the anti-tumor effect of Panobinostat in vitro. The MTS assay showed that Panobinostat significantly suppressed MMQ and GH3 cell viability in a time course (Fig. 2D). The dose of 0.1 μM showed the most potent cytotoxicity at 24 h; while increase of dosages attenuated the cytotoxicity in both MMQ and GH3 cells. Also, Panobinostat markedly suppressed the PRL and GH hormone secretions from MMQ and GH3 cells (*p* < 0.05, Fig. 2E). Consistent with the cell viability results, Panobinostat significantly suppressed both MMQ and GH3 cell colony formations (Fig. 2F). As HTS evaluated growth inhibition rather than direct tumor cell death, the annexin V staining assays were used to measure apoptosis. Indeed, Panobinostat induced apoptosis in both MMQ and GH3 cells at 24 h (Fig. 2G). These data reveal that Panobinostat effectively inhibits pituitary adenoma cell growth and hormone secretion.

### Panobinostat-treated PitNET cells exhibit altered transcriptional profile

**Fig. 3:**
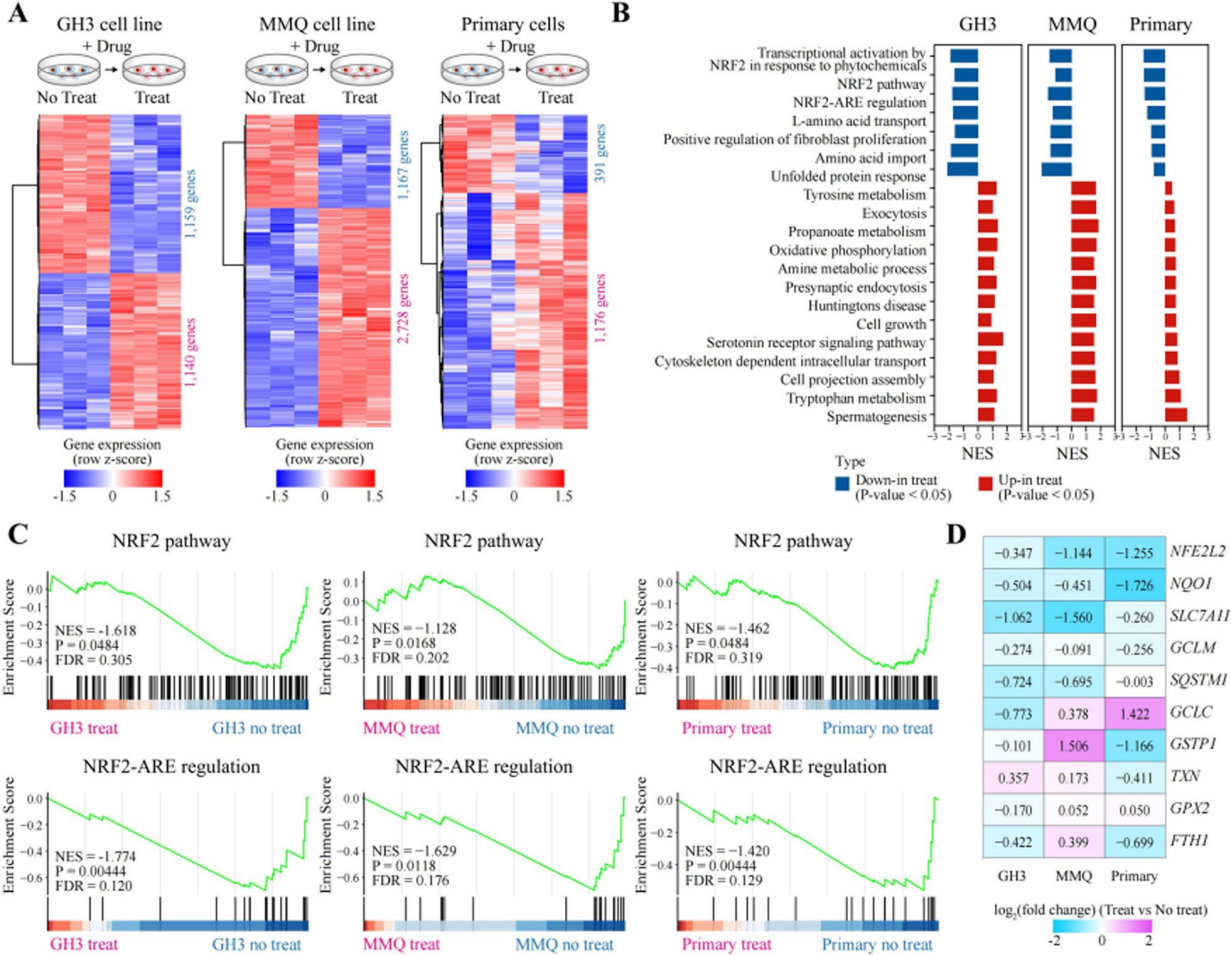
Transcriptome-wide RNA-sequencing assays to identify on-target ability of Panobinostat in PitNET primary, MMQ, and GH3 cells. **A** Transcriptome strategy of RNA sequencing conducted on different PitNET cells exposed to Panobinostat for 24 h is shown in schematic diagram. MMQ, GH3, and primary cell groups contained 3 replicates, respectively. **B** The common core-enriched signaling pathways changed following Panobinostat treatment. **C** Single GSEA was used to analyze the targeted signaling pathway. Normalized enrichment score (NES) indicated the analysis results across gene sets. **D** The gene expressions of Nrf2 and its downstream genes in PitNET cell lines following Panobinostat treatment by RNA sequencing

Prior studies suggested that Panobinostat was a nonselective pan-HDAC inhibitor targeting all four classes of HDACs; however, the exact mechanisms differ [14]. To explore the molecular mechanisms underlying Panobinostat on PitNETs, we performed transcriptome-wide RNA-sequencing analysis in GH3 and MMQ cell lines and 3 primary PitNET cell samples from patients (Additional file 1: Table S1) with or without 24 h treatment of 0.1 μM Panobinostat. We identified expression changes of 2299, 3895, and 1567 genes in GH3, MMQ, and primary cells respectively, upon Panobinostat treatment (Fig. 3A). To identify which pathways were affected by Panobinostat, we performed gene set enrichment analysis (GSEA). As shown in Fig. 3B, the Panobinostat-treated GH3, MMQ, and primary cells shared some same pathway patterns. Strikingly, the top 3 gene sets which were downregulated by Panobinostat were Nrf2-related signaling (all *p* < 0.05; Fig. 3B, C). In addition, Nrf2 downstream antioxidant genes, including NQO1, SLC7A11, GCLM, and SQSTM1, were downregulated as well in GH3, MMQ, and primary PitNET cells following Panobinostat treatment (Fig. 3D). To test whether Panobinostat can inhibit Nrf2 activation, we detected the changes in Nrf2 DNA binding activity in vitro. As shown in Additional file 1: Figure S2, Panobinostat inhibited Nrf2 DNA binding activity in a dose-dependent manner in both MMQ and GH3 cells (both *p* < 0.05). Interestingly, Panobinostat did not induce a significant inhibition in HDAC pathway, whose family members were highly expressed in different PitNET subtypes (Additional file 1: Figure S3). Specially, Panobinostat only slightly downregulated HDAC4 expression with no statistical significance (*p* > 0.05), and even slightly upregulated other HDACs (Additional file 1: Figure S4). These data suggest that Panobinostat may confer anti-tumor effects in PitNETs mainly via inhibiting Nrf2-mediated signaling, rather than directly affecting the expressions of HDACs.

### Nrf2 was highly expressed in human PitNETs

**Fig. 4:**
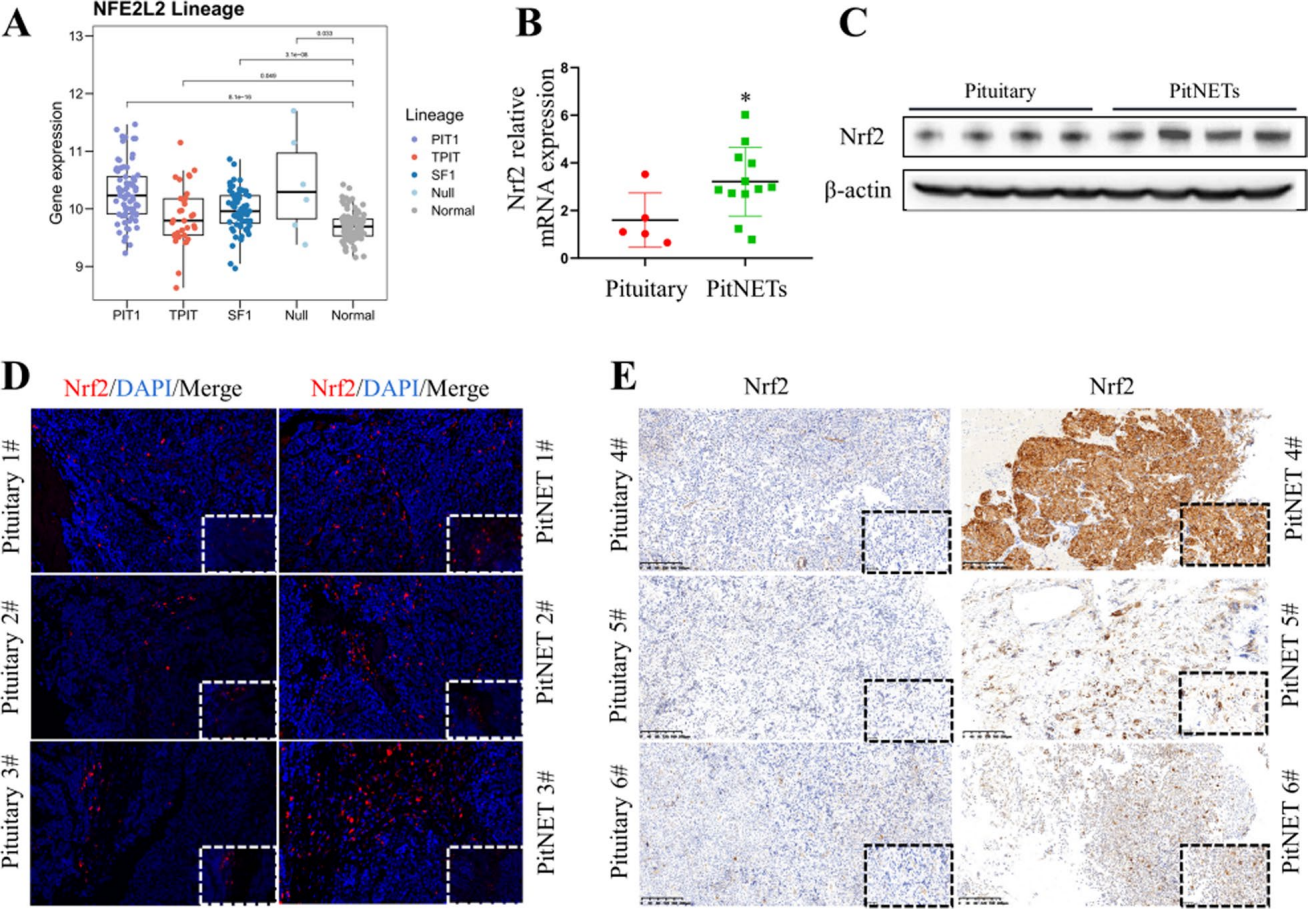
Nrf2 was highly expressed in PitNETs. **A** The gene expressions of Nrf2 in PitNETs by RNA sequencing. n = 180. **B** Validation of Nrf2 mRNA expressions in PitNET specimens. *p < 0.05. **C** Validation of Nrf2 protein expressions in PitNET specimens. **D** Representative IF images of Nrf2 staining in PitNET specimens. **E** Representative IHC images of Nrf2 staining in PitNET specimens

To confirm the potential involvement of Nrf2 in PitNETs, we analyzed the RNA-seq data on a total of 180 different PitNETs from our single expert center. Our data revealed that in general, the Nrf2 transcripts were highly expressed among almost PitNET subtypes (Fig. 4A). Also, the RT-PCR and Western blot analysis were used to detect Nrf2 mRNA and protein expressions in human normal pituitaries and PitNETs. As shown in Fig. 4B, C, Nrf2 mRNA and protein expressions were markedly higher in the PitNETs when compared to that in the normal pituitaries, respectively (*p* < 0*.*05). Furthermore, IF and IHC staining were employed to determine the relative changes in Nrf2 from the clinical PitNET specimens. As shown in Fig. 4D, E, few and abundant Nrf2-positive cells were observed in the normal pituitaries and PitNETs, respectively. To further explore the correlation between Nrf2 expression and PitNET clinical characteristics, we analyzed the Nrf2 expressions using IHC staining on 32 tumor samples from PitNET patients, whose clinical information were summarized in Additional file 1: Table S1. Notably, there was a significantly positive correlation between Nrf2 expression and tumor size, invasion, and recurrence (*p* < 0*.*05, Table 1).

**Table 1:** Correlation of the expression of Nrf2 with clinicopathological features of 32 PitNET patients

	Feature	n	Mean relative Nrf2 expression	*p* value
Age (years)	< 45	13 (40.625%)	2.15	0.464
	≥ 45	19 (59.375%)	2.26	
Sex	Female	21 (65.625%)	2.21	0.453
	Male	11 (34.375%)	2.07	
Tumor diameter	≤ 1 cm	5 (15.625%)	1.64	0.043*
	> 1 cm and ≤ 3 cm	19 (59.375%)	2.04	
	> 3 cm	8 (25%)	2.93	
Knosp grade	0	9 (28.125%)	1.26	0.048*
	1	5 (15.625%)	1.34	
	2	7 (21.875%)	2.03	
	3	4 (12.50%)	2.76	
	4	7 (21.875%)	3.34	
Recurrence	Yes	6 (18.75%)	3.14	0.041*
	No	26 (81.25%)	1.97	

* indicates *p* < 0.05, which is considered a statistically significant difference

### Nrf2 promotes PitNET growth and hormone secretion

**Fig. 5:**
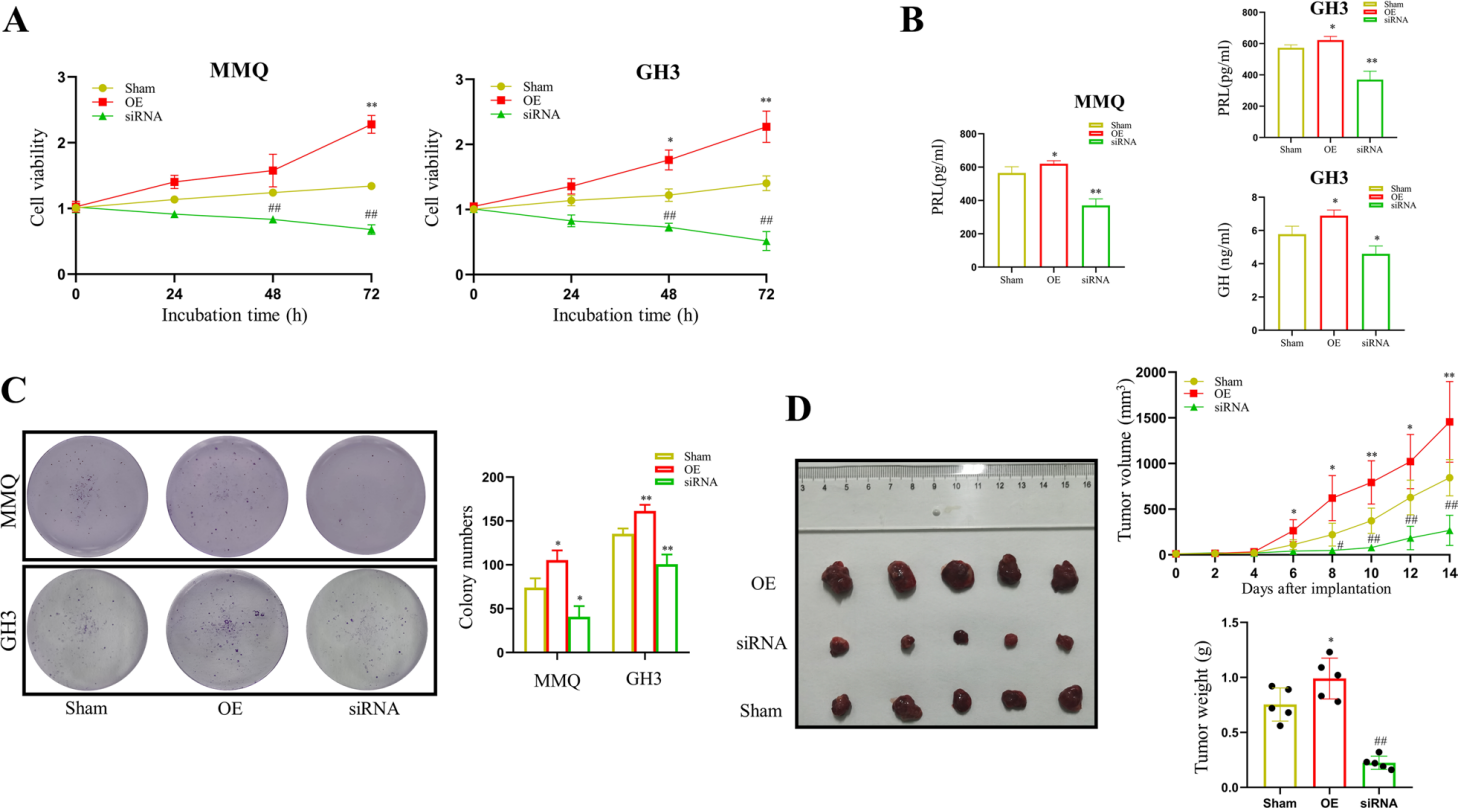
The role of Nrf2 in the pathopoiesis of PitNETs. **A** The inhibitory responses of proliferation for different Nrf2 expressions in MMQ and GH3 cells. n = 3; *p < 0.05, **p < 0.01, ^##^*p* < 0.01. **B** The inhibitory responses of PRL and GH hormone secretions for different Nrf2 expressions in MMQ and GH3 cells. n = 3; *p < 0.05, **p < 0.01, ^#^*p* < 0.05, ^##^*p* < 0.01. **C** Colony formation in MMQ and GH3 cell lines. n = 3; *p < 0.05, **p < 0.01. **D** Left: Representative images of xenograft tumors from mice with different treatment. Right: Tumor volume growth curves and tumor weights of nude mice in different treatment groups. n = 5; *p < 0.05, **p < 0.01, ^#^*p* < 0.05, ^##^*p* < 0.01

To further investigate the effects of Nrf2 on PitNET development, we manipulated its expression in MMQ and GH cells. Over-expression (OE) and knockdown (KD) of Nrf2 were confirmed by western blotting. As shown in Additional file 1: Figure S5, si-R2 was selected in the following studies. The MTS assay showed that KD of Nrf2 significantly suppressed the proliferation of both the MMQ and GH3 cells, while OE of Nrf2 markedly promoted cell proliferation in a time-dependent manner (Fig. 5A, p < 0*.*05). ELISA assays showed that inhibition of Nrf2 expression significantly suppressed the PRL and GH levels in the culture medium from both the MMQ and GH3 cells. Conversely, OE group significantly increased PRL and GH secretion compared to the control group (Fig. 5B, p < 0*.*05). Consistent with the cell proliferation results, the colony formation assays also showed that KD and OE of Nrf2 markedly inhibited and promoted the numbers of MMQ and GH3 colony formations, respectively (Fig. 5C, both *p* < 0*.*05).

Next, the above described Nrf2 OE, Nrf2 KD, and negative control (NC) GH3 cells was injected into nude mice to generate xenograft models. Similar to the results of the in vitro experiments, the mice received Nrf2 OE cells had bigger tumors, while the mice received Nrf2 KD cells had significantly smaller tumors comparing to the mice received the control cells, respectively (Fig. 5D, p < 0*.*05).

### Nrf2 inhibition and Panobinostat demonstrate efficacy as combination treatment in PitNET growth in virto and in vivo

**Fig. 6:**
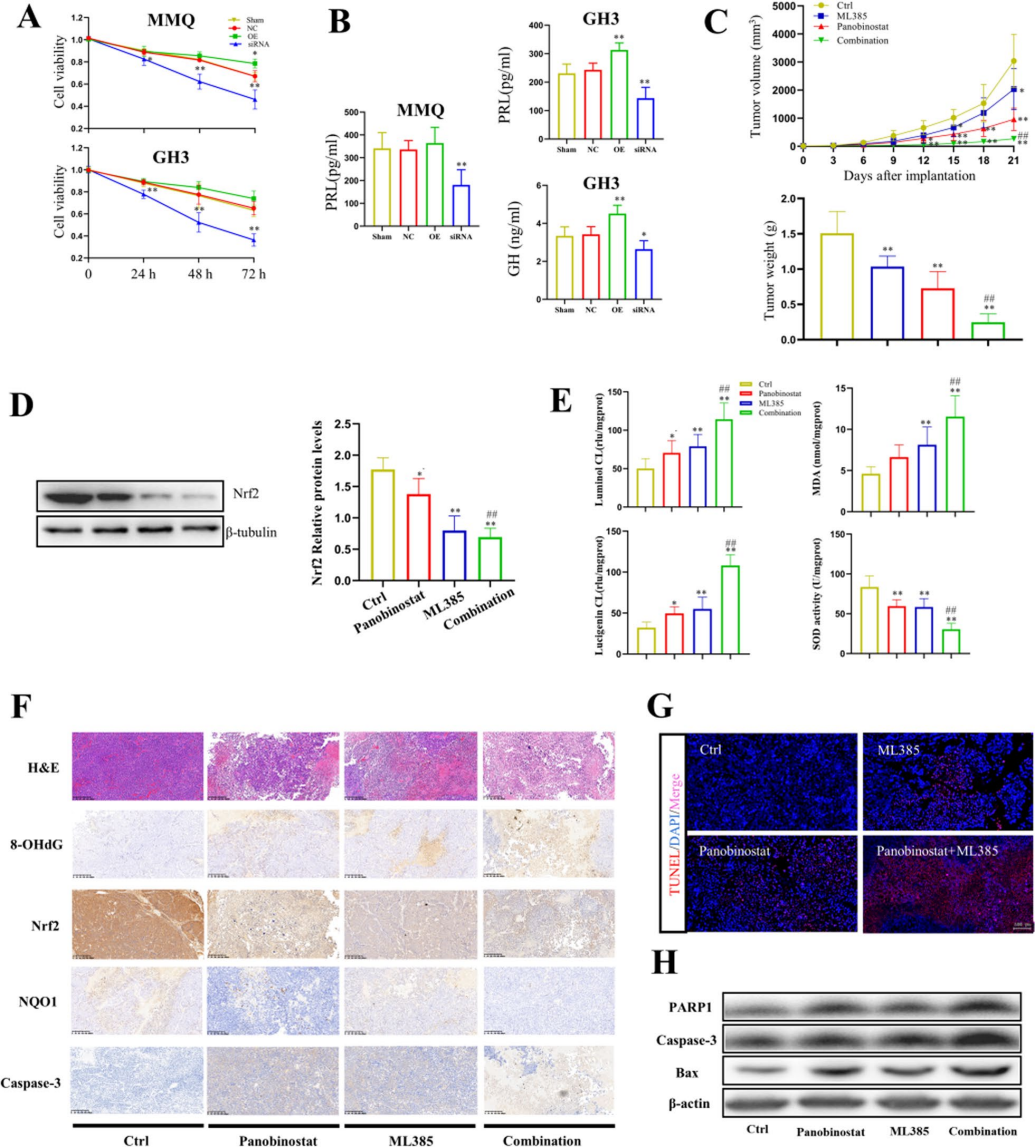
The role of Nrf2 in the cytotoxicity mediated by Panobinostat. **A** The inhibitory responses of proliferation for different Nrf2 expressions with Panobinostat administration in MMQ and GH3 cells. n = 3; *p < 0.05, **p < 0.01. **B** The inhibitory responses of PRL and GH hormone secretions for different Nrf2 expressions with Panobinostat administration in MMQ and GH3 cells. n = 3; *p < 0.05, **p < 0.01. **C** Tumor volume growth curves and tumor weights of nude mice in different treatment groups. n = 5; *p < 0.05, **p < 0.01, ^##^*p* < 0.01. **D** Left: Representation of immuno-blots for different Nrf2 and NQO1 expressions in different groups. Right: Bar graph of densitometric analysis. n = 3; *p < 0.05, **p < 0.01, ^#^*p* < 0.05, ^##^*p* < 0.01. **E** Redox changes evidenced by the levels of luminol CL, lucigenin CL, MDA content, and total SOD activity in each group. n = 3; *p < 0.05, **p < 0.01, ^##^*p* < 0.01. **F** Representative IHC images of H&E, 8-OHdG, Nrf2, NQO1, and caspase-3 staining in different treatment groups. **G** Representative images of TUNEL staining in mice PitNET specimens. **H** Representation of immuno-blots for different apoptosis-related protein expressions in different groups

Given our RNA-seq results showing the potentially modulatory effect of Panobinostat on Nrf2 signaling, Nrf2 OE and KD GH3 and MMQ cells were employed. As shown in Fig. 6A, OE of Nrf2 significantly diminished Panobinostat-induced inhibition of MMQ and GH3 cell proliferation; while KD of Nrf2 markedly increased Panobinostat-induced inhibition of proliferation of MMQ and GH3 cells. Moreover, much lower levels of PRL and GH were detected in the culture media of MMQ and GH3 cells with Nrf2 KD comparing to those from the control group after Panobinostat treatment; but the levels of PRL and GH were significantly higher in the Nrf2 OE GH3 cells (*p* < 0*.*05, Fig. 6B). Similarly, KD of Nrf2 enhanced the suppression of colony formation and promotion of cell apoptosis in MMQ and GH3 cells after Panobinostat treatment. In contrast, OE of Nrf2 alleviated Panobinostat-mediated inhibition of colony formation and induction of cell apoptosis in MMQ and GH3 cells (Additional file 1: Figure S6A–C).

Notably, there were no compounds targeting Nrf2 inhibition in our HTS libraries. To further test the anti-tumor effect of combination of Panobinostat with Nrf2 inhibition, ML385, a special Nrf2 inhibitor, was used. As Fig. 6C, ML385 significantly decreased the GH3 xenograft tumor sizes and weights compared with vehicle treatment (*p* < 0.05). Moreover, the tumor sizes and weights were much smaller in the Combination group compared to those from the Panobinostat group (*p* < 0.05). To further investigate the effects of Panobinostat, used alone or in combination with ML385 on redox homeostasis in vivo, we detected the protein expressions of Nrf2 and its downstream representative target antioxidant gene NQO1 by Western Blot analysis and the levels of oxidative stress by the CL assays of luminol and lucigenin, MDA content, and total SOD activity assays. As shown in Fig. 6D, E, the administration of ML385 enhanced Panobinostat-induced downregulation of Nrf2/NQO1 protein expressions and total SOD activities, and upregulation of luminal CL, lucigenin CL, and MDA content in the GH3 xenograft tumors (*p* < 0.05). IHC staining also showed that the 8-OHdG-positive cells were increased, and Nrf2- and NQO1-positive cells were decreased in the xenograft tumor samples after Panobinostat or ML385 administration; however, the combination group showed much more 8-OHdG-positive cells, and less Nrf2- and NQO1-positive cells compared to the Panobinostat or ML385 group, respectively (Fig. 6F). As for cell death, the number of TUNEL- and caspase-3-positive cells, the protein levels of PARP1, Bax, and caspase-3 were increased in the Combination group compared with those in the Panobinostat group. While the Bcl-2 protein expression was lower in the Combination group compared with that from the Panobinostat group (Fig. 6F–H). Together, the data suggest that the combination of Panobinostat and Nrf2 inhibitor ML385 has a synergistic effect, and effectively inhibits the MMQ and GH3 cell growth and hormone secretion, induces apoptosis, and enhances oxidative stress *in virto* and in vivo.

Previous data indicated that Akt-mediated phosphorylation of mTOR functions downstream of S6K1/4EBP1 to govern the activation of Nrf2 [15, 16]. Herein, Panobinostat decreased the phosphorylation of Akt, mTOR, and 4EBP1 in a dose-dependent manner in both MMQ and GH3 cells, without affecting S6K1 phosphorylation (Additional file 1: Figure S7). These data indicate that Panobinostat markedly inhibit Nrf2 activation, at least in part, via the Akt/mTOR1/4EBP1 phosphorylation.

## Discussion

In this study, we reported for the first time a comprehensive pharmacological landscape for PitNETs based on a panel of patient-derived PitNET primary cell cultures. To our knowledge, the current study represents the first large-scale drug screening for PitNETs providing a detailed overview of the anti-tumor activity using an antineoplastic custom-made drug library. We identified a class of HDACIs as effective anti-PitNET drugs, especially its representative compound, Panobinostat. Panobinostat conferred anti-tumor effects mainly via Nrf2-mediated redox modulation. Notably, combination of Nrf2 inhibitor ML385 and Panobinostat had a synergistic therapeutic effect, and may represent a promising clinical strategy for treating PitNETs.

HTS can directly measure anti-tumor responses which makes it a valuable source of information about the susceptibility of tumor cells to potential therapeutic agents [17]. A successful example of this approach was the identification of actinomycin D as a drug effective against medulloblastoma, which was originally discovered as an antibiotic [18]. Using this approach, we herein identified HDACIs as the most potent drug class for PitNETs. In mouse corticotroph tumor cell line AtT20, several HDACIs, including trichostatin A [19] and CUDC-907 [20] have been demonstrated significantly anti-proliferative efficacy. Of note, it has been reported that the high levels of HDAC1, 2, and 11 were detected in pituitary adenoma cells [21, 22]. More recently, Zhao et al. [23] found that HDAC2/3 are highly expressed in nonfunctional pituitary adenomas. Herein, our comprehensive transcriptional landscape for PitNETs from a total of 180 PitNETs further revealed that HDAC1-11 were widely upregulated across different types of PitNETs. Combined with the HTS results, the present study suggested HDAC inhibition as a promising therapeutic approach in PitNETs.

Among the top ranked screened HDACIs, Panobinostat demonstrated the strongest efficacy with safety at low doses. Notably, Panobinostat has been approved by FDA to treat multiple myeloma in 2015 [13]. In addition, clinical trials for Panobinostat has demonstrated satisfactory clinical efficacy in several other hematologic and solid tumors, including lymphoma [24], sarcoma [25], glioma [26], and melanoma [27]. These clinical data showed that Panobinostat has the potential to be repurposed and fast tracked for the treatment of PitNETs clinically.

Panobinostat is known as a nonselective pan-HDAC inhibitor targeting different HDACs. We find here that Panobinostat did not induce an inhibition in HDAC pathway, and even slightly upregulated most HDACs in PitNET cells via the transcriptional survey. This suggests that Panobinostat may not provide anti-PitNET effects via blocking HDAC enzyme activity. Of note, several other mechanisms have also been proposed, including metabolic reprogramming [14], chromatin stability [28], cell cycle arrest [29], and metabolic collapse [26]. The exact mechanisms depend on the specific type of the tumors [14]. Interestingly, among the most strikingly altered pathways was the downregulation of Nrf2 signaling. As a transcription factor, Nrf2 plays the central role in regulating the celluar redox homeostasis by inducing a wide spectrum of endogenous antioxidative enzymes and thus scavenging excessive reactive oxygen species (ROS) production, thereby optimizing tumor cell survival and drug-resistance. Targeting redox vulnerabilities via regulating Nrf2 has been emerging as an attractive therapeutic modality in cancer [30]. Here, our findings reveal that Panobinostat alters the redox profile in pituitary adenoma cells via downregulating Nrf2, thus reducing cell proliferation and hormone secretion. Moreover, Panobinostat inhibited Nrf2 activation, at least in part, via suppressing the AKT/mTOR/4EBP1 signaling without affecting S6K1 in MMQ and GH3 cells. Together, these data identified Nrf2 as a new therapeutic target following Panobinostat treatment, which brings an update to the mechanisms and concepts of this drug therapy.

Currently, the application of combinatorial drug therapy has revolutionized prognoses for cancers, including PitNETs [31]. We previously identified the combination of Cabergoline and Chloroquine as a promising drug combination, which had potent synergy in PitNET treatment [32]. That study led to ongoing clinical trials of combination therapy in the drug-resistant prolactinomas (NCT02536261) [33]. Given the role Nrf2 played in the PitNET pathogenesis, it would be of importance exploring the effects of combination treatment of Panobinostat and Nrf2 inhibitor. Thus, we evaluated the effects of combination treatment through employing a Nrf2 special inhibitor ML385, which has proven effectiveness in lung cancer [34], colon cancer [30], breast cancer [35], oral squamous cell carcinoma [36], prostate cancer [37]. Encouragingly, Panobinostat and ML385 emerge as a promising combination, demonstrating notable synergy for suppressing PitNET growth in vitro and in vivo. These data support exploring a further clinical trial of panobinostat together with ML385 in PitNETs.

There are still some potential limitations deserving attention in our experiments. We defined PitNET as a whole without subgrouping them to further define whether certain drugs are subtype-specific. In addition, the MMQ and GH3 cell lines are derived from rat, the toxicity of Panobinostat should further be performed in syngenic rat models that can better reflect its potent toxicity in patients. Moreover, the main mechanistic experiments were performed in rat-derived cell lines due to a lack of human PitNET cell lines, more primary PitNET cell cultures from different lineages are further needed to verify our result.

### Supplementary Information

**Additional file 1**. Supplementary Methods, Tables, and Figures.

## Data Availability

Sequencing data can be viewed in NODE (http://www.biosino.org/node) by pasting the accession OEP001353 into the text search box or through the URL: http://www.biosino.org/node/project/detail/OEP001353.
