# Supplementary material for: Therapeutic potential of targeting Nrf2 by panobinostat in pituitary neuroendocrine tumors
Source: Acta Neuropathol Commun. 2024 Apr 18;12:61. doi: 10.1186/s40478-024-01775-2 (PMC11025224; doi:10.1186/s40478-024-01775-2)
Supplement: Supplementary file 1 — Additional file 1. Supplementary Methods, Tables, and Figures. [file 40478_2024_1775_MOESM1_ESM.docx]

**sSupplementary Materials**

**Therapeutic Potential of Targeting Nrf2 by Panobinostat in Pituitary neuroendocrine tumors**

Yijun Cheng, Yuting Dai, Hao Tang, Xingyu Lu, Jing Xie, Wanqun Xie, Qianqian Zhang, Yanting Liu, Hong Yao, Shaojian Lin, Hanbing Shang, Kun Yang, Hongyi Liu, Xuefeng Wu, Jianming Zhang, Xun Zhang, Li Xue, Zhe Bao Wu

**Supplementary Methods**

**Cell culture and reagents**

The pituitary cell lines of GH3, MMQ, and AtT-20 cells were all purchased from the American Type Culture Collection (ATCC, Manassas, VA, USA) and cultured in the F12 medium (Gibco, Grand Island, NY, USA) supplemented with 15% horse serum and 2.5% fetal bovine serum (Gibco). The primary cells from clinical PitNET tissues were harvested and cultured in the Dulbecco's modified Eagle medium (DMEM) medium supplemented with 10% FBS. All the cells were cultured in the humidified atmosphere with 5% CO_2_ at 37 °C.

The chemical compounds used in the present study included dimethyl sulfoxide (DMSO), luminol crystal, lucigenin crystal, L-(+)-Lactic acid (all from Sigma-Aldrich, St. Louis, MO, USA), Panobinostat, and ML385 (both from MedChemExpress, Shanghai, China). CellTiter-Glo®Luminescent Viability Assay kits were purchased from Promega (Madison, WI, USA).

**Isolation and cultivation of PitNETs**

The primary PitNET cells were isolated and cultivated as previously described (1). Briefly, the PitNET specimens were harvested from operation and transferred to DMEM. These tissues were washed with HBSS (Gibco) three times, cut into small pieces of about 3-4 mm using sterile scalpel, and then rinsed and digested with HBSS containing 100 U/ml collagenase, type IV (Gibco) at 37 °C for another 6-8 h. Afterward, the dispersed PitNET cells were sieved using 100 μm cell strainer (Beyotime Biotechnology). Furthermore, the cells were washed with HBSS and resuspended in the ACK Lysing Buffer (Gibco). Five minutes later, these cells were resuspended in DMEM with 10% FBS and seeded in 10 cm cell culture dish for further experiments. Tens of millions primary cells can be isolated from a 1 cm^3^ adenoma.

**RNA sequencing alignment and analysis**

For primary cells analysis, raw FASTQ files of RNA sequencing were aligned to human reference genome GRCh38 (release 40). And for the MMQ and GH3 cell lines, the mouse reference GRCm39 (release 30) was used. The human and mouse reference genome and its annotation file were downloaded from GENCODE database (https://www.gencodegenes.org/). Salmon (v1.8.0) was used to generate the count and transcripts per kilobase of exon model per million mapped reads (TPM) matrix (2). The transcript counts were then merged using DESeq2 and transformed as fragments per kilobase million (FPKM) to evaluate the gene expression level by normalizing the length of genes using the TPM matrix (3). The transformation of gene symbols between human and mouse was performed via R package biomaRt (https://bioconductor.org/packages/biomaRt/). For calculating differentially expressed genes, the limma (4) package was used with significance level setting as *P*-value < 0.05 and log_2_ transformed fold change > 0.58 or < -0.58. For evaluating difference between pituitary tumor and normal pituitary, we download the expression matrix (FPKM) of pituitary from The Genotype-Tissue Expression (GTEx) (5), and merged with the 180 pituitary tumor patients (6). Column-wise median normalization was performed for batch effects adjustment. The R package pheatmap (https://CRAN.R-project.org/package=pheatmap) was used for visualization. For functional enrichment analysis, Gene Ontology (GO) enrichment and Gene set enrichment analysis (GSEA) (7) were performed using the clusterProfiler R package (8). Gene sets used in GSEA were downloaded from the Molecular Signatures Database (MSigDB, v7.5.1) of the Broad Institute. HALLMARK gene sets (H) and KEGG gene sets (C2), and Gene Ontology resource (GO) were used for enrichment analysis.

**Establishment of transiently and stably transfected cells**

The transfection experiment was performed as previously described (9-11). Briefly, the siRNAs against rat Nrf2 was synthesized by GenePharma (Shanghai, China). According to the Manufacturer's instructions, transient transfections of siRNAs or plasmids were conducted with Lipofectamine RNAiMAX reagent (Invitrogen, Carlsbad, CA, USA). The overexpression (OE)-recombinant plasmids were constructed, and sequences confirmed commercially (GenePharma). GH3 cell lines stably expressing Nrf2, Nrf2-specific shRNA or empty vectors were constructed with the lentiviral technique. After 48 h transduction with lentiviral supernatant, MMQ and GH3 cells were selected with 2 μg/mL puromycin for 1-2 w for stable transfectants. The stably transfected cells were further used in the colony formation and tumor xenograft experiments.

**Cell viability assay**

Cell viability assay was performed using MTS-based Cell Titer 96 AQueous One solution cell proliferation assay (Promega). MMQ, GH3, and primary cells isolated from PitNET patients were seeded into 96-well plates in triplicate at approximately 5,000 cells per well. These cells in plates were incubated for 24, 48, and 72 at 37 °C, respectively. At the indicated time points, MTS solution was added and then incubated for another 2 h. The absorbance of each well was detected with a spectrophotometer (TECAN, Mannedorf, Switzerland) at a wavelength of 490 nm.

**Colony formation assay**

A total of 200 parental or stably transfected MMQ or GH3 cells were seeded in 6-well plates. After incubation for 2 weeks, the cells were washed with PBS twice, fixed with Paraformaldehyde for 15 min, and then stained with 0.5% crystal violet dye (Sangon, Shanghai, China) at room temperature. After washing with PBS for three times and complete air drying, the plates were photographed.

**Flow cytometry analysis of cell apoptosis**

Briefly, parental or stably transfected MMQ and GH3 cells were plated into 12-well plates and pretreated with or without Panobinostat at the indicated concentrations. After incubation for 24 h, cells were harvested and the apoptosis was measured by a FITC Annexin V Apoptosis Detection Kit (Beyotime Biotechnology, Haimen, Jiangsu, China) using the CyAn ADP flow cytometry (Beckman Coulter, Brea, USA).

**Quantitative real-time polymerase chain reaction (RT-PCR)**

Total RNA was extracted from cells or tumor tissues using Trizol Reagents (Invitrogen). RNA concentrations were determined by spectrophotometric analysis (OD_260/280_). All primers were purchased from Sangon (Shanghai, China) as following: Nrf2: CAGTCTTCACCACCCCTGAT (forward), CTAATGGCAGCAGAGGAAGG (reverse); β-actin: GTGACGTTGACATCCGTAAAGA (forward), GCCGGACTCATCGTACTCC (reverse). The reverse transcription was performed using a Prime Script RT reagent kit according to the Manufacturer’s protocol (TaKaRa, Otsu, Japan). The RT-PCR was performed with a SYBR Green kit (TaKaRa). The results were calculated using the ΔCt method with the SDS software (Applied Biosystems, Carlsbad, USA).

**Western blot analysis**

Total proteins were extracted from cells and tumor tissues by the RIPA lysis buffer (Millipore, Bedford, USA). The protein concentrations were determined using an enhanced BCA protein assay kit (Beyotime Biotechnology). The proteins (15-50 μg/lane) were subjected to 10% sodium dodecyl sulfate-polyacrylamide gel electrophoresis (SDS-PAGE) and, after electrophoresis, were wet-transferred to 0.22 or 0.45 μm PVDF membranes (Millipore, Temecula, USA). After blocking in the 5% non-fat milk for 1 h at room temperature, the immunoblots were probed with the target primary antibodies (Table S6) at 4 °C overnight. After washing, the immunoblots were incubated with the appropriate secondary antibodies for 1 h at room temperature. The ECL chemiluminescence (CL) solution (Pierce, Rockford, IL, USA) were loaded onto the immunoblots to detect the target protein signals. The final results were quantified with the Image J 1.52a software (NIH, Bethesda, MA, USA).

**Xenograft mouse model**

Female nude mice (4-6 week-old) were purchased from the Experimental Animal Center of the Chinese Academy of Sciences (Shanghai, China). All animal experiments were approved by the Institutional Animal Care and Use Committee at Shanghai Jiao Tong University School of Medicine, and were performed strictly in accordance with the US National Institutes of Health Guidelines. Mice were housed under the specific pathogen-free conditions and provided with sterilized food and water. All efforts have been made to reduce the total number of mice used and to minimize the suffering. The animals were used in three experiments. The first was to observe the anti-tumor and toxic effects of Panobinostat *in vivo.* For this experiment, nude mice were randomly divided into the control and the Panobinostat treatment group (*n* = 18 for each group). The GH3 cells (2 × 10^6^) were injected subcutaneously with a volume of 100 μL in the flank of each mouse. For treatment group, Panobinostat (10 mg/kg) was administered via intraperitoneal (i.p.) on a daily schedule (5-days-on, 2-days-off regimen) for the entire duration of the experiment as previously described (12). An equal volume of indicated concentration of DMSO was used for the control group. On day 14, 6 mice from each group were euthanized and the tumor samples were collected. During the 14 d periods, the tumor size was measured by caliper ruler every 2 days. On day 24, another 12 mice from each group were also euthanized, and the lung and brain samples were harvested for the following adverse side effect experiments. The second experiment was to investigate whether Nrf2 played a pivotal role in the tumourigenicity of PitNETs. In this experiment, a total of 18 mice were equally divided into the NC group, the Nrf2-siRNA (siRNA) group, and the Nrf2-OE (OE) group, with six mice in each group. For each group, parental NC, Nrf2-siRNA, Nrf2-OE transfected GH3 cells (2 × 10^6^) were injected subcutaneously into each mouse, respectively. Tumor size was measured every 2 d after the injection. Animals from each group were euthanized on day 14, and tumor samples were harvested and measured. The third experiment was to investigate the synergetic effect of Nrf2 inhibition and Panobinostat. In this experiment, mice were divided into the control group, the ML385 treatment group, the Panobinostat treatment group, and the combination group of ML385 and Panobinostat (*n* = 6 in each group). The GH3 cells (2 × 10^6^) were injected subcutaneously in the flank of each mouse. ML385 was administered via i.p. injections (30 mg/kg) daily. During the following 21 days, the tumor size was measured every 2 days. On day 21, all mice were euthanized, and the tumor samples were collected and measured followed by IF, IHC, and Western blot assays.

# Cell Counting in bronchoalveolar lavage fluid (BALF)

The BALF-related assays were performed as described with minor modification (13). Briefly, mice were euthanized, the thoracic cavity was revealed, and the trachea was intubated. Then, the right primary bronchus was ligated. Pre-cooling PBS (0.5 mL) was slowly instilled into lungs and subsequently collected for 3 times. The solutions were centrifuged and the supernatants (BALF) were collected. Again, the BALF was centrifuged (1,500 rpm, 10 min at 4°C), and the cell pellet was resuspended in 0.2 mL PBS for detecting total and differential leukocyte count with a Countess automated cell counter (Invitrogen) according to the Manufacturer’s instructions. Differential counting was performed based on the Diff-Quik staining, and the cells were counted according to standard morphologic criteria under a light microscopy. BALF IL-1β and IL-6 can be direct markers of pneumonia (14). Cytokines (IL-1β and IL-6) in BALF were detected with the Enzyme Linked Immunosorbent Assay (ELISA) kits as described below.

**Immunofluorescence (IF) staining**

Tissue samples were cut into 4 μm micron slides (Cryo-sections) and blocked in 10% bovine serum albumin (BSA). After washing, the slides were incubated with the primary antibody (anti-Nrf2) overnight at 4 °C. The specimens were then incubated with the secondary antibodies (Alexa Fluor 488 donkey anti-rabbit secondary antibody) (Table S3) for about 1 h at room temperature. Fluorescence microscope (Leica, Wetzlar, Germany) was used to acquire the fluorescence images.

**Immunohistochemistry (IHC) staining**

Tissue samples from different mice groups and clinical specimens were collected. The tissue sections were dewaxed and rehydrated. Then, an antigen retrieval step was performed. Afterward, the sections were incubated in 3% H_2_O_2_ for 10 min, blocked in PBS containing 5% normal goat serum for 1 h at room temperature, followed by incubation with the primary antibodies at 4 °C overnight. The primary antibodies used were as follows: anti-Nrf2, anti-NQO-1, anti-8-OHdG, and anti-caspsse-3 (Table S3). Futhermore, the sections were counterstained with the ABC kit and detected by DAB (Vector Laboratories, Burlingame, CA, USA). The results were determined by assessing the staining intensity (a total of five grades) as previously described (9,10).

**Terminal Deoxynucleotidyl Transferase-Mediated dUTP Nick 3′-End Labeling (TUNEL)**

The TUNEL experiment was conducted as previously described (10). In brief, the brain and xenograft tumor samples were embedded in paraffin and cut into 4 μm slides. A TUNEL kit (Roche, Basel, Switzerland) was employed to stain the paraffin slides according to the Manufacturer’s protocols.

**Luminol and lucigenin Chemiluminescence (CL) assays**

The xenograft tumor samples were collected at the indicated time points and fractured in the ice-cold working buffer (0.5 M PBS containing 20 mM HEPES, pH 7.2). The luminol and lucigenin crystals were added with a final concentration of 0.2 mM. The changes in the curve area over a total of 5 min were measured as previously described.^9^ The results were expressed as the relative light units (rlu)/mg tumor tissues.

**Malondialdehyde (MDA) and total superoxide dismutase (SOD) activity assays**

Xenograft tumor samples were collected and homogenized in the ice-cold saline. The levels of MDA content and SOD activity were detected using a MDA Content Kit and a Tissue Total SOD activity kit (Jianchen, Nanjing, China), respectively, according to the Manufacturer’s protocols.

**Nrf2 DNA-binding assay**

The activities of Nrf2 DNA binding of celluar samples (MMQ and GH3) were determined with the TransAM Nrf2 Transcription Factor ELISA kit (Active Motif, Carlsbad, USA) according to the Manufacturer’s protocol. A spectrophotometer was used to detect the changes in absorbance at 450 nm.

**ELISA experiment**

The protein levels of prolactin (PRL) and growth hormone (GH) in the culture medium from different cell culture groups were detected by PRL and GH ELISA kit (R&D systems, Minneapolis, USA) according to the Manufacturer’s instructions. The protein levels of IL-1β and IL-6 in the BALF were measured by the IL-1β ELISA kit (Abcam, Cambridge, UK) and IL-6 ELISA kit (R&D Systems). A spectrophotometer was used to measure the 450 nm absorbance value.

**Supplementary References**

1. Liu YT, Liu F, Cao L, Xue L, Gu WT, Zheng YZ, Tang H, Wang Y, Yao H, Zhang Y, Xie WQ, Ren BH, Xiao ZH, Nie YJ, Hu R, Wu ZB. [The KBTBD6/7-DRD2 axis regulates pituitary adenoma sensitivity to dopamine agonist treatment.](https://webvpn.shsmu.edu.cn/https/77726476706e69737468656265737421e0e243912234265e7d0a80e296592e7bb7d62ae2c192eb/32572597/) Acta Neuropathol, 2020; 140(3): 377-396.
2. Patro R, Duggal G, Love MI, et al. Salmon provides fast and bias-aware quantification of transcript expression. Nat Methods, 2017;14:417-9.
3. Love MI, Huber W, Anders S. Moderated estimation of fold change and dispersion for RNA-seq data with DESeq2. Genome Biol. 2014;15:550.
4. Ritchie ME, Phipson B, Wu D, et al. [limma powers differential expression analyses for RNA-sequencing and microarray studies.](https://webvpn.shsmu.edu.cn/https/77726476706e69737468656265737421e0e243912234265e7d0a80e296592e7bb7d62ae2c192eb/25605792/) Nucleic Acids Res. 2015;43(7):e47.
5. GTEx Consortium. [The genotype-tissue expression (GTEx) project.](https://webvpn.shsmu.edu.cn/https/77726476706e69737468656265737421e0e243912234265e7d0a80e296592e7bb7d62ae2c192eb/23715323/) Nat Genet. 2013;45(6):580-585.
6. Yao H, Xie W, Dai Y, et al. [TRIM65 determines the fate of a novel subtype of pituitary neuroendocrine tumors via ubiquitination and degradation of TPIT.](https://webvpn.shsmu.edu.cn/https/77726476706e69737468656265737421e0e243912234265e7d0a80e296592e7bb7d62ae2c192eb/35218667/) Neuro Oncol. 2022;24(8):1286-1297.
7. Subramanian A, Tamayo P, Mootha VK, et al. [Gene set enrichment analysis: a knowledge-based approach for interpreting genome-wide expression profiles.](https://webvpn.shsmu.edu.cn/https/77726476706e69737468656265737421e0e243912234265e7d0a80e296592e7bb7d62ae2c192eb/16199517/) PNAS. 2005;102(43):15545-15550.
8. Yu G, Wang LG, Han Y, et al. [clusterProfiler: an R package for comparing biological themes among gene clusters.](https://webvpn.shsmu.edu.cn/https/77726476706e69737468656265737421e0e243912234265e7d0a80e296592e7bb7d62ae2c192eb/22455463/) OMICS. 2012;16(5):284-287.
9. Yao H, Tang H, Zhang Y, et al. [DEPTOR inhibits cell proliferation and confers sensitivity to dopamine agonist in pituitary adenoma.](https://webvpn.shsmu.edu.cn/https/77726476706e69737468656265737421e0e243912234265e7d0a80e296592e7bb7d62ae2c192eb/31176743/) Cancer Lett. 2019;459:135-144.
10. Cheng Y, Liu M, Tang H, et al. [iTRAQ-based quantitative proteomics indicated Nrf2/OPTN-mediated mitophagy inhibits NLRP3 inflammasome activation after intracerebral hemorrhage.](https://pubmed.ncbi.nlm.nih.gov/33628368/) Oxid Med Cell Longev. 2021;2021:6630281.
11. Cheng YJ, Ding H, Du HQ, et al. [Downregulation of phosphoglycerate kinase 1 by shRNA sensitizes U251 xenografts to radiotherapy.](https://webvpn.shsmu.edu.cn/https/77726476706e69737468656265737421e0e243912234265e7d0a80e296592e7bb7d62ae2c192eb/25175369/) Oncol Rep. 2014;32(4):1513-1520.
12. Crisanti MC, Wallace AF, Kapoor V, et al. [The HDAC inhibitor panobinostat (LBH589) inhibits mesothelioma and lung cancer cells in vitro and in vivo with particular efficacy for small cell lung cancer.](https://webvpn.shsmu.edu.cn/https/77726476706e69737468656265737421e0e243912234265e7d0a80e296592e7bb7d62ae2c192eb/19671764/) Mol Cancer Ther. 2009;8(8):2221-2231.
13. Ma SQ, Wei HL, Zhang X. [TLR2 regulates allergic airway inflammation through NF-κB and MAPK signaling pathways in asthmatic mice.](https://webvpn.shsmu.edu.cn/https/77726476706e69737468656265737421e0e243912234265e7d0a80e296592e7bb7d62ae2c192eb/29863259/) Eur Rev Med Pharmacol Sci. 2018;22(10):3138-3146.
14. Monton C, Torres A. [Lung inflammatory response in pneumonia.](https://webvpn.shsmu.edu.cn/https/77726476706e69737468656265737421e0e243912234265e7d0a80e296592e7bb7d62ae2c192eb/9632909/) Monaldi Arch Chest Dis. 1998;53(1):56-63.

**Supplementary Tables**

**Table S1. The clinical characteristics of 49 PitNET patients for basic experiments.**

| **Patient** | **Application** | **Sex** | **Age (yr)** | **Mo of onset** | **Knosp degree** | **Pre-Symptom** | **Pre-Plasma Hormone** | **Post-Plasma Hormone** | **Pre-Tumor size (mm)** | **Post-Tumor size (mm)** | **IHC** | **Classification** | **Duration of follow-up (Mo)** |
| --- | --- | --- | --- | --- | --- | --- | --- | --- | --- | --- | --- | --- | --- |
| **1** | HTS, PCV | M | 50 | 6 | 4 | Visual defect | PRL>200ng/ml | 72.24ng/ml | 60*51*52 | 29×31×47 | Pit-1(+),PRL(+) | PIT-1 | 51 |
| **2** | HTS, PCV | M | 34 | 0.3 | 4 | Hypopituitarism | PRL>200ng/ml | 0.32ng/ml | 31*28*21 | 0 | Pit-1(+),PRL(+) | PIT-1 | 50 |
| **3** | HTS, PCV, RNA-seq,IHC | M | 30 | 12 | 4 | Sexual dysfunction | PRL>200ng/ml | 23.34ng/ml | 30*30*26 | 0 | Pit-1(+),PRL(+) | PIT-1 | 50 |
| **4** | HTS, PCV | F | 26 | 0.7 | 1 | Recurrence | ACTH: 43.76pg/ml Cor:11.12μg/ml | ACTH: 24.16pg/ml Cor:5.08μg/ml | 20*16*16 | 0 | T-pit(+),ACTH(+) | T-pit | 48 |
| **5** | HTS, PCV | F | 44 | 6 | 3 | Visual defect | N | N | 25*18*18 | 0 | Pit-1(-),T-pit(-),SF(-) | Null cell | 47 |
| **6** | HTS | M | 70 | 2 | 4 | Visual defect | PRL:68.4ng/ml | N | 30*27*26 | 26*22*11 | T-pit(+),ACTH(few+) | T-pit | 46 |
| **7** | HTS, PCV | M | 28 | 12 | 4 | Visual defect | PRL>200ng/ml | PRL>200ng/ml | 34*45*31 | 7*6*5 | Pit-1(+),PRL(+) | PIT-1 | 33 |
| **8** | HTS,IHC, PCV | M | 62 | 12 | 4 | Visual defect and headache | N | N | 52*36*33 | 19*17*18 | SF-1(+) | SF-1 | 33 |
| **9** | HTS | F | 63 | 8 | 4 | Visual defect | N | N | 31*28*22 | 0 | T-pit(+),ACTH(few+) | T-pit | 29 |
| **10** | PCV | M | 46 | 11 | 3 | Headache | N | N | 26*22*18 | 0 | SF-1(+) | SF-1 | 51 |
| **11** | PCV,IHC | F | 35 | 2 | 4 | Visual defect | N | N | 28*27*30 | 0 | SF-1(+) | SF-1 | 45 |
| **12** | PCV | F | 55 | 12 | 3 | Headache | N | N | 19*21*16 | 0 | SF-1(+) | SF-1 | 28 |
| **13** | PCV | F | 64 | 22 | 3 | Visual defect | N | N | 27*24*22 | 0 | SF-1(+) | SF-1 | 28 |
| **14** | PCV | M | 57 | 12 | 2 | Visual defect and diziniess | N | N | 23*19*19 | 0 | SF-1(+) | SF-1 | 27 |
| **15** | PCV,IHC | F | 53 | 1 | 2 | None | N | N | 22*21*20 | 0 | SF-1(+) | SF-1 | 27 |
| **16** | PCV | M | 37 | 6 | 2 | Headache | N | N | 28*23*18 | 0 | Pit-1(-),T-pit(-),SF(-) | Null cell | 26 |
| **17** | PCV,IHC | M | 52 | 24 | 1 | Visual defect | PRL>200ng/ml | 23.16ng/ml | 19*19*13 | 0 | Pit-1(+),PRL(+) | PIT-1 | 20 |
| **18** | PCV | F | 25 | 2 | 1 | Headache | N | N | 21*20*15 | 0 | Pit-1(+),GH(+) | PITt-1 | 20 |
| **19** | PCV | F | 21 | 2 | 2 | Amenorrhea | PRL:137.75ng/ml | PRL:38.92ng/ml | 17*15*12 | 0 | Pit-1(+),PRL(+) | PITt-1 | 20 |
| **20** | PCV | M | 60 | 0.1 | 2 | Headache and nausea | N | N | 24*16*15 | 0 | SF-1(+) | SF-1 | 19 |
| **21** | RNA-seq, IHC | M | 55 | 6 | 4 | Visual defect | N | N | 36*32*29 | 11*9*7 | SF-1(+) | SF-1 | 56 |
| **22** | RNA-seq | F | 44 | 2 | 4 | Visual defect | N | N | 32*28*30 | 0 | SF-1(+) | SF-1 | 47 |
| **23** | IHC | M | 57 | 52 | 0 | Recurrence | N | N | 13*12*12 | 0 | SF-1(+) | SF-1 | 86 |
| **24** | IHC | F | 29 | 4 | 0 | Menstrual disorder | PRL>200ng/ml | PRL:22.34ng/ml | 9*8*8 | 0 | Pit-1(+),PRL(+) | PIT-1 | 54 |
| **25** | IHC | M | 51 | 5 | 2 | Visual defect | PRL:168.32ng/ml | PRL:12.14ng/ml | 28*22*22 | 19×17×11 | Pit-1(+),PRL(+) | PIT-1 | 54 |
| **26** | IHC | F | 24 | 22 | 3 | Headache | N | N | 35*23*25 | 11*10*10 | SF-1(+) | SF-1 | 51 |
| **27** | IHC | F | 52 | 2 | 2 | Headache | N | N | 21*20*15 | 0 | SF-1(+) | SF-1 | 51 |
| **28** | IHC | M | 34 | 0.3 | 4 | Headache and diziness | PRL>200ng/ml | PRL:4.22ng/ml | 34*22*28 | 0 | Pit-1(+),PRL(+) | PIT-1 | 51 |
| **29** | IHC | F | 26 | 48 | 1 | Menstrual disorder | N | N | 24*16*16 | 7*5*5 | T-pit(+),ACTH(+) | T-pit | 48 |
| **30** | IHC | F | 49 | 8 | 4 | Visual defect | N | N | 45*38*38 | 0 | SF-1(+) | SF-1 | 48 |
| **32** | IHC | F | 45 | 32 | 0 | Menstrual disorder | PRL: 68.65ng/ml | PRL:44.14ng/ml | 9*9*8 | 0 | Pit-1(+),PRL(+) | PIT-1 | 42 |
| **33** | IHC | M | 44 | 48 | 0 | Physical examination | N | N | 16*16*14 | 0 | SF-1(+) | SF-1 | 40 |
| **34** | IHC | F | 55 | 8 | 3 | Visual defect | N | N | 38*36*34 | 0 | SF-1(+) | SF-1 | 38 |
| **35** | IHC | M | 48 | 8 | 4 | Visual defect | PRL>200ng/ml | PRL>200ng/ml | 44*42*40 | 12*11*11 | Pit-1(+),PRL(+) | PIT-1 | 34 |
| **36** | IHC | F | 24 | 21 | 0 | Decreased libido | PRL:89.27ng/ml | PRL:8.12ng/ml | 14*12*12 | 0 | Pit-1(+),PRL(+) | PIT-1 | 31 |
| **37** | IHC | M | 60 | 1 | 1 | Physical examination | N | N | 19*18*12 | 0 | SF-1(+) | SF-1 | 31 |
| **38** | IHC | F | 54 | 4 | 1 | Headache | N | N | 16*15*15 | 0 | SF-1(+) | SF-1 | 30 |
| **39** | IHC | F | 48 | 12 | 1 | Headache | N | N | 21*20*17 | 0 | SF-1(+) | SF-1 | 29 |
| **40** | IHC | M | 54 | 11 | 3 | Visual defect | N | N | 28*22*19 | 0 | SF-1(+) | SF-1 | 29 |
| **41** | IHC | F | 51 | 120 | 2 | Acromegaly | GH:4.2ng/ml, IGF-1: 245ng/ml | GH:3.4ng/ml, IGF-1: 168ng/ml | 24*21*22 | 0 | Pit-1(+),GH(+) | PIT-1 | 28 |
| **42** | IHC | F | 26 | 23 | 2 | Hyperglycemia | ACTH:88.12pg/ml, Cor:132.52μg/ml | ACTH:42.71pg/ml, Cor:19.37μg/ml | 21*18*12 | 0 | T-pit(+),ACTH(+) | T-pit | 27 |
| **43** | IHC | F | 49 | 1 | 3 | Headache | N | N | 27*22*22 | 0 | Pit-1(-),T-pit(-),SF(-) | Null cell | 27 |
| **44** | IHC | F | 41 | 8 | 0 | Menstrual disorder | PRL:126.82ng/ml | 78.34ng/ml | 12*11*11 | 0 | Pit-1(+),PRL(+) | PIT-1 | 27 |
| **45** | IHC | F | 36 | 6 | 2 | Headache, Hypopituitarism | N | N | 20*16*14 | 0 | SF-1(+) | SF-1 | 26 |
| **46** | IHC | F | 14 | 28 | 2 | Headache | PRL>200ng/ml | PRL>200ng/ml | 24*21*18 | 8*6*6 | Pit-1(+),PRL(+) | PIT-1 | 26 |
| **47** | IHC | F | 48 | 18 | 0 | Hyperglycemia, acromegaly | GH:4.6ng/ml, IGF-1: 282ng/ml | GH:2.6ng/ml, IGF-1: 143ng/ml | 9*9*8 | 0 | Pit-1(+),GH(+) | PIT-1 | 25 |
| **48** | IHC | F | 24 | 6 | 0 | Menstrual disorder | PRL>200ng/ml | 6.74ng/ml | 8*6*6 | 0 | Pit-1(+),PRL(+) | PIT-1 | 24 |
| **49** | IHC | F | 47 | 140 | 0 | Follow-up | GH:6.2ng/ml, IGF-1: 412ng/ml | GH:4.5ng/ml, IGF-1: 84ng/ml | 8*8*6 | 0 | Pit-1(+),GH(+) | PIT-1 | 24 |
| HTS, high-throughput drug screening; PCV, PitNET primary cell viability; IHC, immunohistochemistry; RNA-seq, RNA sequencing; M, male; F, female; Yr, year; Mo, month; BRC, bromocriptine; PRL, prolactin; ACTH, Adrenocorticotropic hormone; Cor, cortisol; GH, growth hormone; Y: yes; N: normal. | | | | | | | | | | | | | |
| Patient 2, 18, 24, 36, 44, and 48# accepted the DA treatment, showing severe side effects, including severe dizziness, nausea, and vomiting; patient 3, 17, 32, and 46# accepted the DA treatment, showing DA resistance; patient 1, 7, 25, 28, and 35# had visual defect who underwent emergency surgery. | | | | | | | | | | | | | |

**Table S2. The detailed data of compounds from the HTS customized library.**

| **Catalog Number** | **Product Name** | **M.w.** | **CAS Number** | **Target** | **DMSO (mg/mL)Max Solubility** | **DMSO (mM)Max Solubility** | **water (mg/mL)Max Solubility** | **water (mM)Max Solubility** | **Formula** |
| --- | --- | --- | --- | --- | --- | --- | --- | --- | --- |
| S2229 | Eltrombopag Olamine | 564.63 | 496775-62-3 | Others | 89 | 157.6253476 | <1 |  | C29H36N6O6 |
| S7099 | (-)-Blebbistatin | 292.33 | 856925-71-8 | ATPase | 58 | 198.4059111 | <1 |  | C18H16N2O2 |
| S2250 | (-)-Epigallocatechin Gallate | 458.37 | 989-51-5 | Others | 72 | 157.0783428 | 23 | 50.17780396 | C22H18O11 |
| S2876 | (-)-MK 801 Maleate | 337.37 | 121917-57-5 | GluR | 68 | 201.5591191 | <1 |  | C20H19NO4 |
| S2341 | (-)-Parthenolide | 248.32 | 20554-84-1 | Others | 49 | 197.3260309 | <1 |  | C15H20O3 |
| S7383 | (-)-p-Bromotetramisole Oxalate | 373.22 | 62284-79-1 | Others | 74 | 198.2744762 | 30 | 80.3815444 | C13H13BrN2O4S |
| S7071 | (+)-Bicuculline | 367.35 | 485-49-4 | GABA Receptor | 21 | 57.17 | <1 |  | C20H17NO6 |
| S7110 | (+)-JQ1 | 456.99 | 1268524-70-4 | BET | 91 | 199.1290838 | <1 |  | C23H25ClN4O2S |
| S2322 | (+)-Matrine | 248.36 | 519-02-8 | Opioid Receptor | 49 | 197.2942503 | 11 | 44.29054598 | C15H24N2O |
| S2252 | (+)-Usniacin | 344.32 | 7562-61-0 | Others | 4 | 11.61710037 | <1 |  | C18H16O7 |
| S3188 | (+,-)-Octopamine HCl | 189.64 | 770-05-8 | Others | 38 | 200.3796667 | 38 | 200.3796667 | C8H12ClNO2 |
| S1671 | （6-）ε-​Aminocaproic acid | 131.17 | 60-32-2 | Others | 6 | 45.74216665 | 26 | 198.2160555 | C6H13NO2 |
| S4926 | (R)-Nepicastat HCl | 331.81 | 195881-94-8 | Hydroxylase | 66 | 198.9090142 | <1 |  | C14H16ClF2N3S |
| S2423 | *(S)-10-Hydroxycamptothecin* | *364.35* | 19685-09-7 | Topoisomerase | 8 | 21.95690956 | <1 |  | C20H16N2O5 |
| S7505 | (S)-crizotinib | 450.34 | 1374356-45-2 | Others | 42 | 93.26286806 | <1 |  | C21H22Cl2FN5O |
| S7153 | 10058-F4 | 249.35 | 403811-55-2 | c-Myc | 50 | 200.5213555 | <1 |  | C12H11NOS2 |
| S2409 | 10-Deacetylbaccatin-III | 544.59 | 32981-86-5 | Others | 109 | 200.150572 | <1 |  | C29H36O10 |
| S1141 | 17-AAG (Tanespimycin) | 585.69 | 75747-14-7 | HSP | 100 | 170.7387867 | <1 |  | C31H43N3O8 |
| S1142 | 17-DMAG (Alvespimycin) HCl | 653.21 | 467214-21-7 | HSP | 131 | 200.5480626 | <1 |  | C32H49ClN4O8 |
| S7193 | 1-Azakenpaullone | 328.16 | 676596-65-9 | GSK-3 | 66 | 201.1214042 | <1 |  | C15H10BrN3O |
| S4173 | 1-Hexadecanol | 242.44 | 36653-82-4 | Others | 68 | 280.4817687 | 68 | 280.4817687 | C16H34O |
| S2417 | 20-Hydroxyecdysone | 480.63 | 5289-74-7 | Others | 96 | 199.7378441 | <1 |  | C27H44O7 |
| S1233 | 2-Methoxyestradiol (2-MeOE2) | 302.41 | 362-07-2 | HIF | 60 | 198.4061374 | <1 |  | C19H26O3 |
| S3100 | 2-Thiouracil | 128.15 | 141-90-2 | Others | 26 | 202.8872415 | <1 |  | C4H4N2OS |
| S7120 | 3-Deazaneplanocin A (DZNeP) | 262.26 | 120964-45-6 | Histone Methyltransferase | 52 | 198.2765195 | 52 | 198.2765195 | C12H15ClN4O3 |
| S2253 | 3-Indolebutyric acid (IBA) | 203.24 | 133-32-4 | Others | 41 | 201.7319425 | <1 |  | C12H13NO2 |
| S2767 | 3-Methyladenine | 149.15 | 5142-23-4 | PI3K | 3 | 20.11397922 | 20 | 134.0931948 | C6H7N5 |
| S2883 | 4-Aminohippuric Acid | 194.19 | 61-78-9 | Others | 39 | 200.8342345 | 3 | 15.44878727 | C9H10N2O3 |
| S7370 | 4E1RCat | 478.45 | 328998-25-0 | Others | 96 | 200.6479256 | <1 |  | C28H18N2O6 |
| S7369 | 4EGI-1 | 451.28 | 315706-13-9 | Others | 90 | 199.4327247 | <1 |  | C18H12Cl2N4O4S |
| S2256 | 4-Methylumbelliferone (4-MU) | 176.17 | 90-33-5 | Others | 35 | 198.6717375 | <1 |  | C10H8O3 |
| S7555 | *4SC-202* | *447.51* | 910462-43-0 | HDAC | 89 | 198.8782374 | <1 |  | C23H21N5O3S |
| S7272 | 4μ8C | 204.18 | 14003-96-4 | Others | 19 | 93.05514742 | <1 |  | C11H8O4 |
| S2553 | 5-Aminolevulinic acid HCl | 167.59 | 5451-09-2 | Others | 34 | 202.8760666 | 34 | 202.8760666 | C5H10ClNO3 |
| S2659 | 5-hydroxymethyl Tolterodine (PNU 200577, 5-HMT, 5-HM) | 341.49 | 207679-81-0 | AChR | 68 | 199.1273537 | <1 |  | C22H31NO2 |
| S2374 | 5-hydroxytryptophan (5-HTP) | 220.22 | 56-69-9 | Others | 44 | 199.8001998 | 4 | 18.16365453 | C11H12N2O3 |
| S7330 | 6H05 | 590.14 | NA | Rho | 100 | 169.4513166 | 100 | 169.4513166 | C22H31ClF3N3O4S3 |
| S7857 | *8-Bromo-cAMP* | *430.08* | 76939-46-3 | PKA | 35 | 81.38020833 | 80 | 186.0119048 | C10H10BrN5NaO6P |
| S2885 | A-205804 | 300.4 | 251992-66-2 | Others | 60 | 199.7336884 | <1 |  | C15H12N2OS2 |
| S7705 | *A-438079 HCl* | *342.61* | 899431-18-6 | P2 Receptor | 61 | 178.0450074 | 61 | 178.0450074 | C13H10Cl3N5 |
| S2636 | A66 | 393.53 | 1166227-08-2 | PI3K | 79 | 200.7470841 | <1 |  | C17H23N5O2S2 |
| S2670 | A-674563 | 358.44 | 552325-73-2 | Akt, CDK, PKA | 72 | 200.8704386 | 72 | 200.8704386 | C22H22N4O |
| S2697 | A-769662 | 360.39 | 844499-71-4 | AMPK | 72 | 199.7835678 | <1 |  | C20H12N2O3S |
| S2785 | A-803467 | 357.79 | 944261-79-4 | Sodium Channel | 72 | 201.2353615 | <1 |  | C19H16ClNO4 |
| S2674 | A922500 | 428.48 | 959122-11-3 | Diacylglycerol Acyltransferase 1 | 86 | 200.7094847 | <1 |  | C26H24N2O4 |
| S2197 | *A-966492* | *324.35* | 934162-61-5 | PARP | 64 | 197.3177123 | <1 |  | C18H17FN4O |
| S7594 | Abscisic Acid (Dormin*）* | *264.32* | 21293-29-8 | Others | 52 | 196.7312349 | <1 |  | C15H20O4 |
| S8048 | ABT-199 (GDC-0199) | 868.44 | 1257044-40-8 | Bcl-2 | 100 | 115.1490028 | <1 |  | C45H50ClN7O7S |
| S1001 | ABT-263 (Navitoclax) | 974.61 | 923564-51-6 | Bcl-2 | 100 | 102.6051446 | <1 |  | C47H55ClF3N5O6S3 |
| S1002 | ABT-737 | 813.43 | 852808-04-9 | Bcl-2 | 100 | 122.9362084 | <1 |  | C42H45ClN6O5S2 |
| S1165 | ABT-751 (E7010) | 371.41 | 141430-65-1 | Microtubule Associated | 74 | 199.2407313 | <1 |  | C18H17N3O4S |
| S1056 | AC480 (BMS-599626) | 567.01 | 714971-09-2 | HER2 | 113 | 199.2910178 | <1 |  | C27H27FN8O3 |
| S1802 | Acadesine | 258.23 | 2627-69-2 | AMPK | 51 | 197.4983542 | <1 |  | C9H14N4O5 |
| S1271 | Acarbose | 645.6 | 56180-94-0 | Others | 129 | 199.8141264 | 129 | 199.8141264 | C25H43NO18 |
| S4010 | Acebutolol HCl | 372.89 | 34381-68-5 | Adrenergic Receptor | 75 | 201.131701 | 75 | 201.131701 | C18H29ClN2O4 |
| S2602 | Acemetacin | 415.82 | 53164-05-9 | Others | 83 | 199.6055986 | <1 |  | C21H18ClNO6 |
| S2884 | Acesulfame Potassium | 202.25 | 55589-62-3 | Others | 40 | 197.7750309 | 40 | 197.7750309 | C4H5KNO4S |
| S2538 | Acetanilide | 135.16 | 103-84-4 | Others | 27 | 199.7632436 | <1 |  | C8H9NO |
| S1805 | Acetylcholine Chloride | 181.66 | 60-31-1 | AChR | 36 | 198.17241 | 36 | 198.17241 | C7H16ClNO2 |
| S1623 | Acetylcysteine | 163.19 | 616-91-1 | AChR | 33 | 202.2182732 | 33 | 202.2182732 | C5H9NO3S |
| S1807 | Aciclovir | 225.2 | 59277-89-3 | Others | 45 | 199.8223801 | <1 |  | C8H11N5O3 |
| S1806 | Acipimox | 154.12 | 51037-30-0 | Others | 31 | 201.1419673 | <1 |  | C6H6N2O3 |
| S1368 | Acitretin | 326.43 | 55079-83-9 | Others | 20 | 61.26887847 | <1 |  | C21H26O3 |
| S4031 | Aclidinium Bromide | 564.55 | 320345-99-1 | AChR | 113 | 200.159419 | <1 |  | C26H30BrNO4S2 |
| S1276 | Adapalene | 412.52 | 106685-40-9 | Others | 8 | 19.39299913 | <1 |  | C28H28O3 |
| S1718 | Adefovir Dipivoxil | 501.47 | 142340-99-6 | Others | 100 | 199.4137237 | <1 |  | C20H32N5O8P |
| S1983 | Adenine HCl | 171.59 | 2922-28-3 | DNA/RNA Synthesis | 8 | 46.62276356 | 4 | 23.31138178 | C5H6ClN5 |
| S1647 | Adenosine | 267.24 | 58-61-7 | Others | 12 | 44.90345757 | <1 |  | C10H13N5O4 |
| S2082 | Adiphenine HCl | 347.88 | 50-42-0 | Others | 30 | 86.23663332 | 70 | 201.2188111 | C20H26ClNO2 |
| S7365 | AdipoRon | 428.52 | 924416-43-3 | Others | 86 | 200.6907496 | <1 |  | C27H28N2O3 |
| S1139 | ADL5859 HCl | 428.95 | 850173-95-4 | Opioid Receptor | 86 | 200.4895675 | 5 | 11.65637021 | C24H29ClN2O3 |
| S3185 | Adrenalone HCl | 217.65 | 62-13-5 | Adrenergic Receptor | 44 | 202.1594303 | 44 | 202.1594303 | C9H12ClNO3 |
| S2690 | ADX-47273 | 369.36 | 851881-60-2 | GluR | 74 | 200.3465454 | <1 |  | C20H17F2N3O2 |
| S7378 | AEBSF HCl | 239.69 | 30827-99-7 | Serine Protease | 47 | 196.0866119 | 20 | 83.44111144 | C8H11ClFNO2S |
| S1486 | AEE788 (NVP-AEE788) | 440.58 | 497839-62-0 | EGFR, Flt, VEGFR, HER2 | 88 | 199.7367107 | <1 |  | C27H32N6 |
| S1011 | Afatinib (BIBW2992) | 485.94 | 439081-18-2 | EGFR | 97 | 199.613121 | <1 |  | C24H25ClFN5O3 |
| S7810 | *Afatinib (BIBW2992) Dimaleate* | *717.18* | 850140-73-7 | EGFR | 100 | 139.4350093 | <1 |  | C32H33ClFN5O11 |
| S7521 | *Afuresertib (GSK2110183)* | *427.32* | 1047644-62-1 | Akt | 85 | 198.9141627 | <1 |  | C18H17Cl2FN4OS |
| S1234 | AG-1024 | 305.17 | 65678-07-1 | IGF-1R | 61 | 199.8885867 | <1 |  | C14H13BrN2O |
| S2178 | AG-14361 | 320.39 | 328543-09-5 | PARP | 12 | 37.45435251 | <1 |  | C19H20N4O |
| S2728 | AG-1478 (Tyrphostin AG-1478) | 315.75 | 153436-53-4 | EGFR | 25 | 79.17656374 | <1 |  | C16H14ClN3O2 |
| S8009 | AG-18 | 186.17 | 118409-57-7 | EGFR | 37 | 198.7430843 | <1 |  | C10H6N2O2 |
| S1143 | AG-490 (Tyrphostin B42) | 294.3 | 133550-30-8 | JAK, EGFR | 59 | 200.4757051 | <1 |  | C17H14N2O3 |
| S7185 | AGI-5198 | 462.56 | 1355326-35-0 | Dehydrogenase | 24 | 51.88516084 | <1 |  | C27H31FN4O2 |
| S7241 | AGI-6780 | 481.51 | 1432660-47-3 | Dehydrogenase | 96 | 199.3728064 | <1 |  | C21H18F3N3O3S2 |
| S7577 | *AGK2* | *434.27* | 304896-28-4 | Sirtuin | 10 | 23.02714901 | <1 |  | C23H13Cl2N3O2 |
| S1243 | Agomelatine | 243.3 | 138112-76-2 | 5-HT Receptor | 49 | 201.3974517 | <1 |  | C15H17NO2 |
| S7814 | AI-10-49 | 660.52 | 1256094-72-0 | Others | 100 | 151.40 | <1 |  | C30H22F6N6O5 |
| S1640 | Albendazole | 265.33 | 54965-21-8 | Others | 17 | 64.07115667 | <1 |  | C12H15N3O2S |
| S1836 | Albendazole Oxide | 281.33 | 54029-12-8 | Others | 11 | 39.09998934 | <1 |  | C12H15N3O3S |
| S1468 | Alfacalcidol | 400.64 | 41294-56-8 | Others | 80 | 199.6805112 | <1 |  | C27H44O2 |
| S1409 | Alfuzosin HCl | 425.91 | 81403-68-1 | Adrenergic Receptor | 85 | 199.5726797 | 85 | 199.5726797 | C19H28ClN5O4 |
| S1928 | Alibendol | 251.28 | 26750-81-2 | Others | 50 | 198.9812162 | <1 |  | C13H17NO4 |
| S1133 | Alisertib (MLN8237) | 518.92 | 1028486-01-2 | Aurora Kinase | 27 | 52.0311416 | <1 |  | C27H20ClFN4O4 |
| S2199 | Aliskiren Hemifumarate | 609.83 | 173334-58-2 | RAAS | 100 | 163.9801256 | 100 | 163.9801256 | C64H110N6O16 |
| S4257 | Alizapride HCl | 351.83 | 59338-87-3 | Others | 42 | 119.3758349 | 70 | 198.9597249 | C16H22ClN5O2 |
| S2526 | Alizarin | 240.21 | 72-48-0 | Others | 48 | 199.825153 | <1 |  | C14H8O4 |
| S1630 | Allopurinol | 136.11 | 315-30-0 | Others | 27 | 198.3689663 | 4 | 29.387995 | C5H4N4O |
| S1631 | Allopurinol Sodium | 159.1 | 17795-21-0 | Others | 32 | 201.1313639 | 32 | 201.1313639 | C5H4N4NaO+ |
| S4017 | Allylthiourea | 116.18 | 109-57-9 | Others | 23 | 197.9686693 | <1 |  | C4H8N2S |
| S2160 | Almorexant HCl | 549.02 | 913358-93-7 | OX Receptor | 72 | 131.1427635 | <1 |  | C29H32ClF3N2O3 |
| S2096 | Almotriptan Malate | 469.55 | 181183-52-8 | 5-HT Receptor | 94 | 200.1916729 | <1 |  | C21H31N3O7S |
| S2259 | Aloe-emodin | 270.24 | 481-72-1 | Others | 3 | 11.10124334 | <1 |  | C15H10O5 |
| S2868 | Alogliptin | 339.39 | 850649-61-5 | DPP-4 | 68 | 200.3594685 | 2 | 5.892925543 | C18H21N5O2 |
| S2375 | Aloin | 418.39 | 1415-73-2 | Others | 83 | 198.3795024 | <1 |  | C21H22O9 |
| S7393 | Aloxistatin | 342.43 | 88321-09-9 | Cysteine Protease | 68 | 198.5807318 | <1 |  | C17H30N2O5 |
| S1508 | Alprostadil | 354.48 | 745-65-3 | Others | 71 | 200.2933875 | <1 |  | C20H34O5 |
| S3167 | Altrenogest | 310.43 | 850-52-2 | Estrogen/progestogen Receptor | 62 | 199.7229649 | <1 |  | C21H26O2 |
| S1278 | Altretamine | 210.28 | 645-05-6 | Others | 15 | 71.33346015 | <1 |  | C9H18N6 |
| S7218 | Alvelestat (AZD9668) | 545.53 | 848141-11-7 | Others | 100 | 183.3079757 | <1 |  | C24H20F3N5O4S |
| S3054 | Alverine Citrate | 473.56 | 5560-59-8 | Others | 95 | 200.6081595 | <1 |  | C26H35NO7 |
| S1544 | AM1241 | 503.33 | 444912-48-5 | Cannabinoid Receptor | 101 | 200.6635806 | <1 |  | C22H22IN3O3 |
| S2819 | AM251 | 555.24 | 183232-66-8 | Cannabinoid Receptor | 40 | 72.04091924 | <1 |  | C22H21Cl2IN4O |
| S2451 | Amantadine HCl | 187.7 | 665-66-7 | Dopamine Receptor | 38 | 202.4507192 | 38 | 202.4507192 | C10H18ClN |
| S2097 | Ambrisentan | 378.42 | 177036-94-1 | Others | 76 | 200.835051 | <1 |  | C22H22N2O4 |
| S3064 | Ambroxol HCl | 414.56 | 23828-92-4 | Sodium Channel |  | 10 |  |  | C13H19Br2ClN2O |
| S2452 | Amfebutamone HCl | 276.2 | 31677-93-7 | Dopamine Receptor | 8 | 28.96451846 | <1 |  | C13H19Cl2NO |
| S4149 | Amfenac Sodium Monohydrate | 295.27 | 61618-27-7 | Others | 59 | 199.8171165 | 59 | 199.8171165 | C15H14NNaO4 |
| S2747 | AMG-458 | 539.58 | 913376-83-7 | c-Met | 21 | 38.91915935 | <1 |  | C30H29N5O5 |
| S7115 | AMG-517 | 430.4 | 659730-32-2 | TRPV | 86 | 199.8141264 | <1 |  | C20H13F3N4O2S |
| S2719 | AMG-900 | 503.58 | 945595-80-2 | Aurora Kinase | 100 | 198.5781802 | <1 |  | C28H21N7OS |
| S7884 | *AMI-1* | *548.45* | 20324-87-2 | Histone Methyltransferase | 100 | 182.3320266 | 10 | 18.23320266 | C21H14N2Na2O9S2 |
| S3209 | Amidopyrine | 231.29 | 58-15-1 | Others | 46 | 198.8845173 | 10 | 43.23576462 | C13H17N3O |
| S1811 | Amiloride HCl | 266.09 | 2016-88-8 | Others | 53 | 199.1807283 | 6 | 22.5487617 | C6H9Cl2N7O |
| S2560 | Amiloride hydrochloride dihydrate | 302.12 | 17440-83-4 | Sodium Channel | 60 | 198.5965841 | <1 |  | C6H13Cl2N7O3 |
| S1672 | Aminoglutethimide | 232.28 | 125-84-8 | Aromatase | 20 | 86.10297916 | <1 |  | C13H16N2O2 |
| S1673 | Aminophylline | 420.43 | 317-34-0 | PDE | 53 | 126.0614133 | 84 | 199.7954475 | C16H24N10O4 |
| S4198 | Aminothiazole | 100.14 | 96-50-4 | Others | 20 | 199.7203915 | 20 | 199.7203915 | C3H4N2S |
| S1979 | Amiodarone HCl | 681.77 | 19774-82-4 | Potassium Channel | 23 | 33.73571732 | <1 |  | C25H30ClI2NO3 |
| S1280 | Amisulpride | 369.48 | 71675-85-9 | Dopamine Receptor | 74 | 200.2814767 | <1 |  | C17H27N3O4S |
| S3183 | Amitriptyline HCl | 313.86 | 549-18-8 | 5-HT Receptor | 63 | 200.7264385 | 15 | 47.79200918 | C20H24ClN |
| S1905 | Amlodipine | 408.88 | 88150-42-9 | Calcium Channel | 82 | 200.547838 | <1 |  | C20H25ClN2O5 |
| S1813 | Amlodipine besylate (Norvasc) | 567.05 | 111470-99-6 | Others | 113 | 199.2769597 | <1 |  | C26H31ClN2O8S |
| S2376 | Ammonium Glycyrrhizinate | 839.96 | 1407-03-0 | Others | 168 | 200.0095243 | 5 | 5.952664413 | C42H65NO16 |
| S1367 | Amonafide | 283.33 | 69408-81-7 | Topoisomerase | 57 | 201.1788374 | <1 |  | C16H17N3O2 |
| S1676 | Amorolfine HCl | 353.97 | 78613-38-4 | Others | 28 | 79.10274882 | 9 | 25.42588355 | C21H36ClNO |
| S3015 | Amoxicillin | 365.4 | 26787-78-0 | Others | 73 | 199.7810619 | <1 |  | C16H19N3O5S |
| S2565 | Amoxicillin Sodium | 387.39 | 34642-77-8 | Others | 78 | 201.3474793 | 78 | 201.3474793 | C16H18N3NaO5S |
| S1636 | Amphotericin B | 924.08 | 1397-89-3 | Others | 22 | 23.80746256 | <1 |  | C47H73NO17 |
| S3170 | Ampicillin sodium | 371.39 | 69-52-3 | Others | 37 | 99.62573036 | 74 | 199.2514607 | C16H18N3NaO4S |
| S4148 | Ampicillin Trihydrate | 403.45 | 7177-48-2 | Others | 81 | 200.7683728 | 2 | 4.957243772 | C16H25N3O7S |
| S4011 | Ampiroxicam | 447.46 | 99464-64-9 | COX | 90 | 201.135297 | <1 |  | C20H21N3O7S |
| S1639 | Amprenavir | 505.63 | 161814-49-9 | HIV Protease | 16 | 31.64369203 | <1 |  | C25H35N3O6S |
| S4144 | Amprolium HCl | 315.24 | 137-88-2 | Others | 2 | 6.344372542 | 63 | 199.8477351 | C14H20Cl2N4 |
| S1244 | Amuvatinib (MP-470) | 447.51 | 850879-09-3 | c-Met, c-Kit, PDGFR, Flt, c-RET | 32 | 71.50678197 | <1 |  | C23H21N5O3S |
| S7745 | *ANA-12* | *407.49* | 219766-25-3 | Trk receptor | 37 | 90.79977423 | <1 |  | C22H21N3O3S |
| S7582 | *Anacardic Acid* | *348.52* | 16611-84-0 | Histone Acetyltransferase | 69 | 197.9800298 | <1 |  | C22H36O3 |
| S2748 | Anacetrapib (MK-0859) | 637.51 | 875446-37-0 | CETP | 127 | 199.21 | <1 |  | C30H25F10NO3 |
| S3172 | Anagrelide HCl | 292.55 | 58579-51-4 | PDE | 14 | 47.85506751 | <1 |  | C10H8Cl3N3O |
| S1188 | Anastrozole | 293.37 | 120511-73-1 | Aromatase | 59 | 201.1112247 | <1 |  | C17H19N5 |
| S1140 | Andarine | 441.36 | 401900-40-1 | Androgen Receptor | 88 | 199.383723 | <1 |  | C19H18F3N3O6 |
| S2261 | Andrographolide | 350.45 | 5508-58-7 | Others | 70 | 199.7431873 | <1 |  | C20H30O5 |
| S4286 | Anidulafungin (LY303366) | 1140.24 | 166663-25-8 | Others | 100 | 87.70083491 | <1 |  | C58H73N7O17 |
| S1281 | Aniracetam | 219.24 | 72432-10-1 | AMPA Receptor-kainate Receptor-NMDA Receptor | 44 | 200.6933041 | <1 |  | C12H13NO3 |
| S7409 | *Anisomycin* | *265.3* | 22862-76-6 | JNK | 41 | 154.5420279 | <1 |  | C14H19NO4 |
| S4199 | Antazoline HCl | 301.81 | 2508-72-7 | Others | 40 | 132.5337133 | 14 | 46.38679964 | C17H20ClN3 |
| S3173 | Antipyrine | 188.23 | 60-80-0 | Others | 38 | 201.8806779 | 38 | 201.8806779 | C11H12N2O |
| S7000 | AP26113 | 529.01 | 1197958-12-5 | ALK | 45 | 85.06455455 | <1 |  | C26H34ClN6O2P |
| S2221 | Apatinib | 493.58 | 811803-05-1 | VEGFR | 22 | 44.57230844 | <1 |  | C25H27N5O4S |
| S2262 | Apigenin | 270.24 | 520-36-5 | P450 | 54 | 199.8223801 | <1 |  | C15H10O5 |
| S1593 | Apixaban | 459.5 | 503612-47-3 | Factor Xa | 18 | 39.17301415 | <1 |  | C25H25N5O4 |
| S2425 | Apocynin | 166.17 | 498-02-2 | Others | 33 | 198.5918036 | <1 |  | C9H10O3 |
| S2927 | Apoptosis Activator 2 | 306.14 | 79183-19-0 | Caspase | 61 | 199.2552427 | <1 |  | C15H9Cl2NO2 |
| S8034 | Apremilast (CC-10004) | 460.5 | 608141-41-9 | PDE | 92 | 199.7828447 | <1 |  | C22H24N2O7S |
| S1189 | Aprepitant | 534.43 | 170729-80-3 | Substance P | 107 | 200.2133114 | <1 |  | C23H21F7N4O3 |
| S2244 | AR-42 | 312.36 | 935881-37-1 | HDAC | 63 | 201.69 | <1 |  | C18H20N2O3 |
| S7435 | AR-A014418 | 308.31 | 487021-52-3 | GSK-3 | 62 | 201.0962992 | <1 |  | C12H12N4O4S |
| S2120 | Arbidol HCl | 513.88 | 131707-23-8 | Others | 103 | 200.4358994 | <1 |  | C22H26BrClN2O3S |
| S2263 | Arbutin | 272.25 | 497-76-7 | Others | 55 | 202.020202 | 55 | 202.020202 | C12H16O7 |
| S2614 | Arecoline | 236.11 | 300-08-3 | AChR | 47 | 199.0597603 | 47 | 199.0597603 | C8H14BrNO2 |
| S2069 | Argatroban | 508.63 | 74863-84-6 | Others | 9 | 17.69459135 | <1 |  | C23H36N6O5S |
| S1975 | Aripiprazole | 448.39 | 129722-12-9 | 5-HT Receptor | 90 | 200.7181248 | <1 |  | C23H27Cl2N3O2 |
| S2840 | ARN-509 | 477.43 | 956104-40-8 | Adrenergic Receptor | 18 | 37.70186205 | <1 |  | C21H15F4N5O2S |
| S7355 | ARQ 621 | 552.43 | 1095253-39-6 | Kinesin | 100 | 181.0184096 | <1 |  | C28H24Cl2FN5O2 |
| S2264 | Artemether | 298.37 | 71963-77-4 | Others | 60 | 201.0926031 | <1 |  | C16H26O5 |
| S1282 | Artemisinin | 282.33 | 63968-64-9 | Others | 57 | 201.8914037 | <1 |  | C15H22O5 |
| S2265 | Artesunate | 384.42 | 88495-63-0 | Others | 77 | 200.3017533 | <1 |  | C19H28O8 |
| S3150 | Articaine HCl | 320.84 | 23964-57-0 | Others | 64 | 199.4763745 | 64 | 199.4763745 | C13H21ClN2O3S |
| S2671 | AS-252424 | 305.28 | 900515-16-4 | PI3K | 61 | 199.8165618 | <1 |  | C14H8FNO4S |
| S2681 | AS-604850 | 285.22 | 648449-76-7 | PI3K | 57 | 199.8457331 | <1 |  | C11H5F2NO4S |
| S2531 | Asaraldehyde | 196.2 | 4460-86-0 | COX | 39 | 198.7767584 | <1 |  | C10H12O4 |
| S7411 | *Ascomycin (FK520)* | *792.01* | 104987-12-4 | Others | 100 | 126.2610321 | <1 |  | C43H69NO12 |
| S1283 | Asenapine | 401.84 | 65576-45-6, 135883-08-8 (Maleic acid) | Adrenergic Receptor, 5-HT Receptor | 80 | 199.0842126 | <1 |  | C21H20ClNO5 |
| S2266 | Asiatic Acid | 488.7 | 464-92-6 | p38 MAPK | 97 | 198.4857786 | <1 |  | C30H48O5 |
| S8054 | ASP3026 | 580.74 | 1097917-15-1 | ALK | 14 | 24.11 | <1 |  | C29H40N8O3S |
| S2036 | Aspartame | 294.3 | 22839-47-0 | Others | 33 | 112.1304791 | 4 | 13.59157322 | C14H18N2O5 |
| S3017 | Aspirin | 180.16 | 50-78-2 | Others | 36 | 199.8223801 | <1 |  | C9H8O4 |
| S2185 | AST-1306 | 621.08 | 1050500-29-2 | EGFR | 124 | 199.6522187 | <1 |  | C31H26ClFN4O5S |
| S2415 | Astragaloside A | 784.97 | 83207-58-3 | others | 100 | 127.3934036 | <1 |  | C41H68O14 |
| S2812 | AT101 | 578.61 | 866541-93-7 | Bcl-2 | 116 | 200.4804618 | <1 |  | C32H34O10 |
| S7563 | *AT13148* | *313.78* | 1056901-62-2 | Akt | 62 | 197.5906686 | <1 |  | C17H16ClN3O |
| S1163 | AT13387 | 409.52 | 912999-49-6 | HSP | 25 | 61.04707951 | <1 |  | C24H31N3O3 |
| S1524 | AT7519 | 382.24 | 844442-38-2 | CDK | 10 | 26.16157388 | <1 |  | C16H17Cl2N5O2 |
| S7808 | *AT7519 HCl* | *418.71* | 902135-91-5 | CDK | 52 | 124.1909675 | 43 | 102.696377 | C16H18Cl3N5O2 |
| S1558 | AT7867 | 337.85 | 857531-00-1 | Akt, S6 kinase | 68 | 201.2727542 | <1 |  | C20H20ClN3 |
| S1134 | AT9283 | 381.43 | 896466-04-9 | Bcr-Abl, JAK, Aurora Kinase | 76 | 199.2501901 | <1 |  | C19H23N7O2 |
| S6003 | Ataluren (PTC124) | 284.24 | 775304-57-9 | Others | 57 | 200.5347594 | <1 |  | C15H9FN2O3 |
| S1457 | Atazanavir Sulfate | 802.93 | 229975-97-7 | HIV Protease | 104 | 129.5256124 | <1 |  | C38H54N6O11S |
| S7364 | Atglistatin | 283.37 | 1469924-27-3 | Others | 57 | 201.1504394 | <1 |  | C17H21N3O |
| S3175 | Atomoxetine HCl | 291.82 | 82248-59-7 | 5-HT Receptor | 58 | 198.7526557 | 2 | 6.853539853 | C17H22ClNO |
| S2077 | Atorvastatin Calcium | 1155.34 | 134523-03-8 | HMG-CoA Reductase | 100 | 86.5546073 | <1 |  | C66H68CaF2N4O10 |
| S3079 | Atovaquone | 366.84 | 95233-18-4 | Others | 5 | 13.6299204 | <1 |  | C22H19ClO3 |
| S1832 | Atracurium Besylate | 1243.48 | 64228-81-5 | Others | 100 | 80.41946794 | 33 | 26.53842442 | C65H82N2O18S2 |
| S2130 | Atropine | 694.83 | 5908-99-6 | Others | 139 | 200.0489328 | 139 | 200.0489328 | C34H50N2O11S |
| S1451 | Aurora A Inhibitor I | 588.07 | 1158838-45-9 | Aurora Kinase | 118 | 200.6563844 | <1 |  | C31H31ClFN7O2 |
| S1069 | AUY922 (NVP-AUY922) | 465.54 | 747412-49-3 | HSP | 93 | 199.7680113 | <1 |  | C26H31N3O5 |
| S1262 | Avagacestat (BMS-708163) | 520.88 | 1146699-66-2 | Gamma-secretase | 104 | 199.6621103 | <1 |  | C20H17ClF4N4O4S |
| S4019 | Avanafil | 483.95 | 330784-47-9 | PDE | 97 | 200.4339291 | <1 |  | C23H26ClN7O3 |
| S2187 | Avasimibe | 501.72 | 166518-60-1 | P450 | 100 | 199.3143586 | <1 |  | C29H43NO4S |
| S7173 | AVL-292 | 423.44 | 1202757-89-8 | BTK | 85 | 200.7368222 | <1 |  | C22H22FN5O3 |
| S1904 | Avobenzone | 310.39 | 70356-09-1 | Others | 62 | 199.7487032 | <1 |  | C20H22O3 |
| S1005 | Axitinib | 386.47 | 319460-85-0 | VEGFR, PDGFR, c-Kit | 35 | 90.56330375 | <1 |  | C22H18N4OS |
| S2731 | AZ 3146 | 452.55 | 1124329-14-1 | Kinesin | 28 | 61.8716164 | <1 |  | C24H32N6O3 |
| S2746 | AZ 628 | 451.52 | 878739-06-1 | Raf | 90 | 199.3267186 | <1 |  | C27H25N5O2 |
| S2214 | AZ 960 | 354.36 | 905586-69-8 | JAK | 71 | 200.3612146 | <1 |  | C18H16F2N6 |
| S7338 | AZ191 | 429.52 | 1594092-37-1 | Others | 86 | 200.22 | <1 |  | C24H27N7O |
| S7050 | AZ20 | 412.51 | 1233339-22-4 | ATM/ATR | 83 | 201.2072435 | <1 |  | C21H24N4O3S |
| S7298 | *AZ5104* | *485.58* | 1421373-98-9 | EGFR | 97 | 199.7611104 | <1 |  | C27H31N7O2 |
| S1782 | Azacitidine | 244.2 | 320-67-2 | DNA/RNA Synthesis | 48 | 196.5601966 | <1 |  | C8H12N4O5 |
| S3196 | Azacyclonol | 267.37 | 115-46-8 | Others | 53 | 198.2271758 | 2 | 7.480270786 | C18H21NO |
| S4194 | Azaguanine-8 | 152.11 | 134-58-7 | Others | 6 | 39.44513839 | <1 |  | C4H4N6O |
| S4219 | Azaperone | 327.4 | 1649-18-9 | Others | 65 | 198.5339035 | <1 |  | C19H22FN3O |
| S3186 | Azatadine dimaleate | 522.55 | 3978-86-7 | Histamine Receptor | 105 | 200.9377093 | 105 | 200.9377093 | C28H30N2O8 |
| S1721 | Azathioprine | 277.26 | 446-86-6 | Others | 54 | 194.7630383 | <1 |  | C9H7N7O2S |
| S7145 | AZD1080 | 334.37 | 612487-72-6 | GSK-3 | 52 | 155.5163442 | <1 |  | C19H18N4O2 |
| S7104 | AZD1208 | 379.48 | 1204144-28-4 | Pim | 75 | 197.6388742 | <1 |  | C21H21N3O2S |
| S2162 | AZD1480 | 348.77 | 935666-88-9, 1260222-79-4 (TFA) | JAK | 69 | 197.8381168 | <1 |  | C14H14ClFN8 |
| S7263 | AZD1981 | 388.87 | 802904-66-1 | GPR | 11 | 28.28708823 | <1 |  | C19H17ClN2O3S |
| S2783 | AZD2014 | 462.54 | 1009298-59-2 | mTOR | 38 | 82.15505686 | <1 |  | C25H30N6O3 |
| S7029 | AZD2461 | 395.43 | 1174043-16-3 | PARP | 79 | 199.7825152 | <1 |  | C22H22FN3O3 |
| S7253 | AZD2858 | 453.52 | 486424-20-8 | GSK-3 | 7 | 15.43482096 | <1 |  | C21H23N7O3S |
| S7003 | *AZD2932* | *447.49* | 883986-34-3 | PDGFR | 89 | 198.887126 | <1 |  | C24H25N5O4 |
| S7106 | AZD3463 | 448.95 | 1356962-20-3 | ALK | 24 | 53.45806883 | <1 |  | C24H25ClN6O |
| S7040 | AZD3514 | 519.56 | 1240299-33-5 | Androgen Receptor | 100 | 192.470552 | <1 |  | C25H32F3N7O2 |
| S7731 | *AZD3839* | *431.41* | 1227163-84-9 | BACE | 86 | 199.3463295 | <1 |  | C24H16F3N5 |
| S2801 | AZD4547 | 463.57 | 1035270-39-3 | FGFR | 92 | 198.4597795 | <1 |  | C26H33N5O3 |
| S8019 | AZD5363 | 428.92 | 1143532-39-1 | Akt | 86 | 200.5035904 | <1 |  | C21H25ClN6O2 |
| S2621 | AZD5438 | 371.46 | 602306-29-6 | CDK | 74 | 199.2139127 | <1 |  | C18H21N5O2S |
| S1462 | AZD6482 | 408.45 | 1173900-33-8 | PI3K | 82 | 200.7589668 | <1 |  | C22H24N4O4 |
| S7517 | AZD7545 | 478.87 | 252017-04-2 | Others | 95 | 198.3836949 | <1 |  | C19H18ClF3N2O5S |
| S1532 | AZD7762 | 362.42 | 860352-01-8 | Chk | 50 | 137.9614812 | <1 |  | C17H19FN4O2S |
| S1555 | AZD8055 | 465.54 | 1009298-09-2 | mTOR | 50 | 107.4021566 | <1 |  | C25H31N5O4 |
| S7694 | *AZD8186* | *457.47* | 1627494-13-6 | PI3Kβ, PI3Kδ | 91 | 198.9201478 | <1 |  | C24H25F2N3O4 |
| S2134 | AZD8330 | 461.23 | 869357-68-6 | MEK | 92 | 199.4666435 | <1 |  | C16H17FIN3O4 |
| S2192 | AZD8931 (Sapitinib) | 473.93 | 848942-61-0 | EGFR, HER2 | 40 | 84.40064989 | <1 |  | C23H25ClFN5O3 |
| S7297 | AZD9291 | 499.61 | 1421373-65-0 | EGFR | 99 | 198.1545606 | <1 |  | C28H33N7O2 |
| S2552 | Azelastine HCl | 418.36 | 79307-93-0 | Histamine Receptor | 84 | 200.7840138 | 35 | 83.66000574 | C22H25Cl2N3O |
| S3053 | Azelnidipine | 582.65 | 123524-52-7 | Calcium Channel | 117 | 200.8066592 | <1 |  | C33H34N4O6 |
| S3046 | Azilsartan | 456.45 | 147403-03-0 | RAAS | 91 | 199.3646621 | <1 |  | C25H20N4O5 |
| S3057 | Azilsartan Medoxomil | 568.53 | 863031-21-4 | RAAS | 114 | 200.5171231 | <1 |  | C30H24N4O8 |
| s1835 | Azithromycin | 748.98 | 83905-01-5 | autophagy | 100 | 133.5149136 | <1 |  | C38H72N2O12 |
| S4147 | Azithromycin Dihydrate | 785.02 | 117772-70-0 | Others | 100 | 127.3852895 | 10 | 12.73852895 | C38H76N2O14 |
| S3195 | Azlocillin sodium salt | 484.48 | 37091-65-9 | Others | 97 | 200.2146631 | 97 | 200.2146631 | C20H23N5NaO6S |
| S2267 | Azomycin | 113.07 | 527-73-1 | Others | 22 | 194.5697356 | <1 |  | C3H3N3O2 |
| S1505 | Aztreonam | 435.43 | 78110-38-0 | Others | 87 | 199.8024941 | 11 | 25.26238431 | C13H17N5O8S2 |
| S4146 | Bacitracin | 1408.67 | 1405-87-4 | Others | 100 | 70.98894702 | 100 | 70.98894702 | C65H101N17O16S |
| S7179 | *BAF312 (Siponimod)* | *516.6* | 1230487-00-9 | S1P Receptor | 100 | 193.5733643 | <1 |  | C29H35F3N2O3 |
| S1369 | *Bafetinib (INNO-406)* | *576.62* | 859212-16-1 | Bcr-Abl | 100 | 173.424439 | <1 |  | C30H31F3N8O |
| S2268 | Baicalein | 270.24 | 491-67-8 | P450 | 54 | 199.8223801 | <1 |  | C15H10O5 |
| S2269 | Baicalin | 446.36 | 21967-41-9 | Others | 89 | 199.3906264 | <1 |  | C21H18O11 |
| S2064 | Balofloxacin | 389.42 | 127294-70-6 | Others | 5 | 12.83960762 | <1 |  | C20H24FN3O4 |
| S4277 | Bambuterol HCl | 403.9 | 81732-46-9 | Adrenergic Receptor | 80 | 198.0688289 | 72 | 178.261946 | C18H30ClN3O5 |
| S4920 | *b-AP15* | *419.39* | 1009817-63-3 | DUB | 48 | 114.4519421 | <1 |  | C22H17N3O6 |
| S7534 | BAPTA-AM | 764.68 | 126150-97-8 | Others | 20 | 26.15473139 | <1 |  | C34H40N2O18 |
| S1147 | Barasertib (AZD1152-HQPA) | 507.56 | 722544-51-6 | Aurora Kinase | 102 | 200.9614627 | <1 |  | C26H30FN7O3 |
| S8078 | Bardoxolone Methyl | 505.69 | 218600-53-4 | IκB/IKK | 21 | 41.53 | <1 |  | C32H43NO4 |
| S2851 | Baricitinib (LY3009104, INCB028050) | 371.42 | 1187594-09-7 | JAK | 74 | 199.235367 | <1 |  | C16H17N7O2S |
| S7155 | Batimastat (BB-94) | 477.64 | 130370-60-4 | MMP | 96 | 200.9881919 | <1 |  | C23H31N3O4S2 |
| S2913 | BAY 11-7082 | 207.25 | 19542-67-7 | IκB/IKK | 41 | 197.8287093 | <1 |  | C10H9NO2S |
| S7352 | *Bay 11-7085* | *249.33* | 196309-76-9 | IκB/IKK | 50 | 200.5374403 | <1 |  | C13H15NO2S |
| S2128 | Bazedoxifene HCl | 507.06 | 198480-56-7 | Estrogen/progestin receptor | 101 | 199.1874729 | <1 |  | C30H35ClN2O3 |
| S3078 | Beclomethasone dipropionate | 521.04 | 5534-09-8 | Others | 104 | 199.6007984 | <1 |  | C28H37ClO7 |
| S1085 | Belinostat (PXD101) | 318.35 | 414864-00-9 | HDAC | 64 | 201.0365949 | <1 |  | C15H14N2O4S |
| S4197 | Bemegride | 155.19 | 64-65-3 | Others | 31 | 199.7551389 | <1 |  | C8H13NO2 |
| S1284 | Benazepril HCl | 460.95 | 86541-74-4 | RAAS | 92 | 199.5878078 | 19 | 41.21922117 | C24H29ClN2O5 |
| S1212 | Bendamustine HCl | 394.72 | 3543-75-7 | Others | 78 | 197.6084313 | 2 | 5.066882854 | C16H22Cl3N3O2 |
| S2017 | Benidipine HCl | 542.02 | 91599-74-5 | calcium channel | 8 | 14.75960297 | <1 |  | C28H32ClN3O6 |
| S2453 | Benserazide HCl | 293.7 | 14919-77-8 | Dopamine Receptor | 58 | 197.4804222 | 58 | 197.4804222 | C10H16ClN3O5 |
| S4221 | Benzbromarone | 424.08 | 3562-84-3 | Others | 85 | 200.4338804 | <1 |  | C17H12Br2O3 |
| S4162 | Benzethonium Chloride | 448.08 | 121-54-0 | Others | 90 | 200.8569898 | 90 | 200.8569898 | C27H42ClNO2 |
| S4210 | Benzocaine | 165.19 | 94-09-7 | Others | 33 | 199.7699619 | <1 |  | C9H11NO2 |
| S4161 | Benzoic Acid | 122.12 | 65-85-0 | Others | 24 | 196.5280052 | <1 |  | C7H6O2 |
| S3163 | Benztropine mesylate | 403.53 | 132-17-2 | Histamine Receptor | 50 | 123.9065249 | 81 | 200.7285704 | C22H29NO4S |
| S4165 | Benzydamine HCl | 345.87 | 132-69-4 | Others | 69 | 199.4969208 | 69 | 199.4969208 | C19H24ClN3O |
| S3037 | Bepotastine Besilate | 547.06 | 190786-44-8 | Histamine Receptor | 109 | 199.2468833 | <1 |  | C27H31ClN2O6S |
| S2271 | Berberine HCl | 371.82 | 633-65-8 | Others | 74 | 199.0210317 | <1 |  | C20H18ClNO4 |
| S4239 | Bergapten | 216.19 | 484-20-8 | Others | 5 | 23.12780425 | <1 |  | C12H8O4 |
| S2270 | Bergenin | 328.27 | 477-90-7 | Others | 66 | 201.0540104 | <1 |  | C14H16O9 |
| S3176 | Betahistine 2HCl | 209.12 | 5579-84-0 | Histamine Receptor | 38 | 181.7138485 | 38 | 181.7138485 | C8H14Cl2N2 |
| S7261 | Beta-Lapachone | 242.27 | 4707-32-8 | Topoisomerase | 33 | 136.2116647 | <1 |  | C15H14O3 |
| S1500 | Betamethasone | 392.46 | 378-44-9 | Others | 79 | 201.2943994 | <1 |  | C22H29FO5 |
| S1688 | *Betamethasone Dipropionate* | *504.59* | 5593-20-4 | Others | 101 | 200.1625082 | <1 |  | C28H37FO7 |
| S1690 | Betamethasone valerate (Betnovate) | 476.58 | 2152-44-5 | Others | 95 | 199.3369424 | <1 |  | C27H37FO6 |
| S4191 | Betamipron | 193.2 | 3440-28-6 | Others | 39 | 201.863354 | <1 |  | C10H11NO3 |
| S2091 | Betaxolol | 307.43 | 659-18-7 | Adrenergic Receptor | 62 | 201.6719253 | 62 | 201.6719253 | C18H29NO3 |
| S1827 | Betaxolol hydrochloride (Betoptic) | 343.89 | 63659-19-8 | Adrenergic Receptor | 69 | 200.6455553 | 69 | 200.6455553 | C18H30ClNO3 |
| S2455 | Bethanechol chloride | 196.68 | 590-63-6 | AChR | 39 | 198.2916412 | 39 | 198.2916412 | C7H17ClN2O2 |
| S3603 | Betulinic acid | 456.7 | 472-15-1 | N/A | 20 | 43.79242391 | <1 |  | C30H48O3 |
| S2098 | Bexarotene | 348 | 153559-49-0 | Others | 8 | 22.98850575 | <1 |  | C24H28O2 |
| S4159 | Bezafibrate | 361.82 | 41859-67-0 | Others | 72 | 198.9939749 | <1 |  | C19H20ClNO4 |
| S7689 | *BG45* | *214.22* | 926259-99-6 | HDAC | 42 | 196.0601251 | <1 |  | C11H10N4O |
| S2749 | BGT226 (NVP-BGT226) | 650.6 | 1245537-68-1 | PI3K, mTOR | 30 | 46.11128189 | <1 |  | C32H29F3N6O6 |
| S1109 | BI 2536 | 521.66 | 755038-02-9 | PLK | 21 | 40.25610551 | <1 |  | C28H39N7O3 |
| S7843 | *BI-847325* | *464.56* | 1207293-36-4 | MEK | 19 | 40.8989151 | <1 |  | C29H28N4O2 |
| S1186 | BIBR 1532 | 331.36 | 321674-73-1 | Telomerase | 66 | 199.1791405 | <1 |  | C21H17NO3 |
| S1190 | Bicalutamide | 430.37 | 90357-06-5 | Androgen Receptor, P450 | 86 | 199.8280549 | <1 |  | C18H14F4N2O4S |
| S2843 | BI-D1870 | 391.42 | 501437-28-1 | S6 Kinase | 78 | 199.2744367 | <1 |  | C19H23F2N5O2 |
| S1854 | Bifonazole | 310.39 | 60628-96-8 | others | 62 | 199.7487032 | <1 |  | C22H18N2 |
| S1175 | BIIB021 | 318.76 | 848695-25-0 | HSP | 64 | 200.7780148 | <1 |  | C14H15ClN6O |
| S7722 | *Bikinin* | *273.08* | 188011-69-0 | GSK-3 | 54 | 197.7442508 | <1 |  | C9H9BrN2O3 |
| S2276 | Bilobalide | 326.3 | 33570-04-6 | Others | 65 | 199.2031873 | <1 |  | C15H18O8 |
| S1407 | Bimatoprost | 415.57 | 155206-00-1 | Others | 36 | 86.62800491 | <1 |  | C25H37NO4 |
| S3032 | Bindarit | 324.37 | 130641-38-2 | Others | 65 | 200.3884453 | <1 |  | C19H20N2O3 |
| S7198 | BIO | 356.17 | 667463-62-9 | GSK-3 | 71 | 199.3430104 | <1 |  | C16H10BrN3O2 |
| S2377 | Biochanin A | 284.26 | 491-80-5 | Others | 57 | 200.5206501 | <1 |  | C16H12O5 |
| S3130 | Biotin (Vitamin B7) | 244.31 | 58-85-5 | Others | 49 | 200.5648561 | <1 |  | C10H16N2O3S |
| S1574 | BIRB 796 (Doramapimod) | 527.66 | 285983-48-4 | p38 MAPK | 106 | 200.8869348 | <1 |  | C31H37N5O3 |
| S7015 | Birinapant | 806.94 | 1260251-31-7 | Caspase | 100 | 123.924951 | <1 |  | C42H56F2N8O6 |
| S4047 | Bisacodyl | 361.39 | 30652-11-0 | Others | 72 | 199.2307479 | <1 |  | C22H19NO4 |
| S1206 | Bisoprolol fumarate | 441.52 | 104344-23-2 | Adrenergic Receptor | 88 | 199.3114695 | 88 | 199.3114695 | C22H35NO8 |
| S8006 | BIX 01294 | 600.02 | 1392399-03-9 | Methyltransferase | 98 | 163.3278891 | 98 | 163.3278891 | C28H41Cl3N6O2 |
| S1530 | BIX 02188 | 412.48 | 1094614-84-2 | MEK | 43 | 104.2474787 | <1 |  | C26H26N4O2 |
| S1531 | BIX 02189 | 440.54 | 1094614-85-3 | MEK | 60 | 136.1964861 | <1 |  | C27H28N4O2 |
| S2247 | BKM120 (NVP-BKM120, Buparlisib) | 410.39 | 944396-07-0 | PI3K | 82 | 199.8099369 | <1 |  | C18H21F3N6O2 |
| S1214 | Bleomycin Sulfate | 1512.62 | 9041-93-4 | DNA/RNA Synthesis | 100 | 66.11045735 | 100 | 66.11045735 | C55H85N17O25S4 |
| S7725 | *BLZ945* | *398.48* | 953769-46-5 | CSF-1R | 79 | 198.2533628 | <1 |  | C20H22N4O3S |
| S2854 | BML-190 | 426.89 | 2854-32-2 | Cannabinoid Receptor | 22 | 51.53552437 | <1 |  | C23H23ClN2O4 |
| S2014 | BMS-265246 | 345.34 | 582315-72-8 | CDK | 20 | 57.91393989 | <1 |  | C18H17F2N3O2 |
| S8044 | BMS-345541 | 255.32 | 445430-58-0 | IκB/IKK | 9 | 35.2498825 | <1 |  | C14H17N5 |
| S2632 | BMS-378806 | 406.43 | 357263-13-9 | gp120/CD4 | 81 | 199.30 | <1 |  | C22H22N4O4 |
| S1012 | BMS-536924 | 479.96 | 468740-43-4 | IGF-1R | 96 | 200.0166681 | <1 |  | C25H26ClN5O3 |
| S1366 | BMS-707035 | 410.42 | 729607-74-3 | Integrase | 38 | 92.5880805 | <1 |  | C17H19FN4O5S |
| S1124 | BMS-754807 | 461.49 | 1001350-96-4 | IGF-1R | 92 | 199.3542655 | <1 |  | C23H24FN9O |
| S1561 | BMS-777607 | 512.89 | 1025720-94-8 | c-Met | 47 | 91.63758311 | <1 |  | C25H19ClF2N4O4 |
| S2201 | BMS-794833 | 468.84 | 1174046-72-0 | c-Met, VEGFR | 94 | 200.4948383 | <1 |  | C23H15ClF2N4O3 |
| S7138 | BMS-833923 | 473.57 | 1059734-66-5 | Hedgehog/Smoothened | 95 | 200.6039234 | <1 |  | C30H27N5O |
| S2691 | BMY 7378 | 458.42 | 21102-95-4 | 5-HT Receptor | 92 | 200.6893242 | 92 | 200.6893242 | C22H33Cl2N3O3 |
| S1013 | Bortezomib (PS-341) | 384.24 | 179324-69-7 | Proteasome | 76 | 197.793046 | <1 |  | C19H25BN4O4 |
| S4220 | Bosentan | 551.61 | 147536-97-8 | Others | 100 | 181.2875039 | <1 |  | C27H29N5O6S |
| S3051 | Bosentan Hydrate | 569.63 | 157212-55-0 | ETA Receptor | 100 | 175.5525517 | 0.001 | 0.001755526 | C27H31N5O7S |
| S1014 | Bosutinib (SKI-606) | 530.45 | 380843-75-4 | Src | 100 | 188.5191818 | <1 |  | C26H29Cl2N5O3 |
| S7753 | *BPTES* | *524.68* | 314045-39-1 | Others | 100 | 190.5923611 | <1 |  | C24H24N6O2S3 |
| S7883 | *BQ-123* | *610.7* | 136553-81-6 | Endothelin Receptor | 100 | 163.7465204 | <1 |  | C31H42N6O7 |
| S7607 | *BQU57* | *334.1* | 1637739-82-2 | Others | 66 | 197.545645 | <1 |  | C16H13F3N4O |
| S7591 | *BRD4770* | *413.47* | 1374601-40-7 | Histone Methyltransferase | 27 | 65.30098919 | <1 |  | C25H23N3O3 |
| S7726 | *BRD73954* | *284.31* | 1440209-96-0 | HDAC | 56 | 196.9680982 | <1 |  | C16H16N2O3 |
| S7363 | *BRD7552* | *711.63* | 1137359-47-7 | Others | 100 | 140.5224625 | <1 |  | C33H33N3O15 |
| S7046 | Brefeldin A | 280.36 | 20350-15-6 | ATPase | 4 | 14.26737052 | <1 |  | C16H24O4 |
| S4266 | Brimonidine Tartrate | 442.22 | 70359-46-5 | Others | 88 | 198.9959749 | 75 | 169.5988422 | C15H16BrN5O6 |
| S3178 | Brinzolamide | 383.51 | 138890-62-7 | Carbonic Anhydrase | 77 | 200.7770332 | <1 |  | C12H21N3O5S3 |
| S1084 | Brivanib (BMS-540215) | 370.38 | 649735-46-6 | VEGFR, FGFR | 74 | 199.7948053 | <1 |  | C19H19FN4O3 |
| S1138 | Brivanib Alaninate (BMS-582664) | 441.46 | 649735-63-7 | VEGFR, FGFR | 88 | 199.3385584 | <1 |  | C22H24FN5O4 |
| S2677 | BRL-15572 | 479.87 | 193611-72-2 | 5-HT Receptor | 96 | 200.0541813 | <1 |  | C25H29Cl3N2O |
| S2852 | BRL-54443 | 230.31 | 57477-39-1 | 5-HT Receptor | 46 | 199.7307976 | <1 |  | C14H18N2O |
| S4248 | Bromfenac Sodium | 356.15 | 91714-93-1 | Others | 71 | 199.3542047 | 71 | 199.3542047 | C15H11BrNNaO3 |
| S2060 | Bromhexine HCl | 412.59 | 611-75-6 | Others | 6 | 14.54228168 | <1 |  | C14H21Br2ClN2 |
| S7918 | *Bromodeoxyuridine (BrdU)* | *307.1* | 59-14-3 | DNA/RNA Synthesis | 61 | 198.6323673 | <1 |  | C9H11BrN2O5 |
| S7233 | Bromosporine | 404.44 | 1619994-69-2 | Epigenetic Reader Domain | 81 | 200.2769261 | <1 |  | C17H20N6O4S |
| S2585 | Brompheniramine hydrogen maleate | 435.31 | 980-71-2 | Histamine Receptor | 87 | 199.8575728 | 46 | 105.6718201 | C20H23BrN2O4 |
| S4195 | Broxyquinoline | 302.95 | 521-74-4 | Others | 17 | 56.11487044 | <1 |  | C9H5Br2NO |
| S1572 | BS-181 HCl | 416.99 | 1397219-81-6 | CDK | 83 | 199.0455407 | 3 | 7.194417132 | C22H33ClN6 |
| S7460 | BTB06584 | 417.82 | 219793-45-0 | ATPase | 84 | 201.0435116 | <1 |  | C19H12ClNO6S |
| S1097 | BTZ043 Racemate | 431.39 | 957217-65-1 | Others | 22 | 50.9979369 | <1 |  | C17H16F3N3O5S |
| S1286 | Budesonide | 430.53 | 51333-22-3 | Others | 86 | 199.7537918 | <1 |  | C25H34O6 |
| S3023 | Bufexamac | 223.27 | 2438-72-4 | HDAC | 45 | 201.5496932 | <1 |  | C12H17NO3 |
| S2607 | Buflomedil HCl | 343.85 | 35543-24-9 | Others | 31 | 90.1555911 | 69 | 200.6688963 | C17H26ClNO4 |
| S1287 | Bumetanide | 364.42 | 28395-03-1 | Others | 73 | 200.318314 | <1 |  | C17H20N2O5S |
| S2454 | Bupivacaine HCl | 324.89 | 18010-40-7 | Others | 65 | 200.0677152 | 23 | 70.79319154 | C18H29ClN2O |
| S4256 | Buspirone HCl | 421.96 | 33386-08-2 | Others | 84 | 199.071002 | 84 | 199.071002 | C21H32ClN5O2 |
| S1692 | Busulfan | 246.3 | 55-98-1 | NULL | 49 | 198.9443768 | <1 |  | C6H14O6S2 |
| S8036 | Butein | 272.25 | 487-52-5 | EGFR | 55 | 202.020202 | <1 |  | C15H12O5 |
| S3154 | Butenafine HCl | 353.93 | 101827-46-7 | Others | 70 | 197.7792219 | <1 |  | C23H28ClN |
| S1833 | Butoconazole nitrate | 474.79 | 64872-77-1 | Others | 95 | 200.0884602 | <1 |  | C19H18Cl3N3O3S |
| S7597 | BV-6 | 1205.57 | 1001600-56-1 | IAP | 100 | 82.9483149 | 25 | 20.73707873 | C70H96N10O8R |
| S1274 | BX-795 | 591.47 | 702675-74-9 | PDK-1, IKK | 100 | 169.0702825 | <1 |  | C23H26IN7O2S |
| S1275 | BX-912 | 471.35 | 702674-56-4 | PDK-1 | 94 | 199.4271773 | <1 |  | C20H23BrN8O |
| S2814 | BYL719 | 441.47 | 1217486-61-7 | PI3K | 88 | 199.3340431 | <1 |  | C19H22F3N5O2S |
| S7152 | C646 | 445.42 | 328968-36-1 | Histone Acetyltransferase | 13 | 29.18593687 | <1 |  | C24H19N3O6 |
| S3022 | Cabazitaxel | 835.93 | 183133-96-2 | Others | 100 | 119.6272415 | <1 |  | C45H57NO14 |
| S7766 | *Cabotegravir (GSK744, GSK1265744)* | *405.35* | 1051375-10-0 | Integrase | 38 | 93.74614531 | <1 |  | C19H17F2N3O5 |
| S1119 | Cabozantinib (XL184, BMS-907351) | 501.51 | 849217-68-1 | VEGFR, c-Met, Flt, Tie-2, c-Kit | 100 | 199.3978186 | <1 |  | C28H24FN3O5 |
| S4001 | Cabozantinib malate (XL184) | 635.59 | 1140909-48-3 | VEGFR | 100 | 157.3341305 | <1 |  | C32H30FN3O10 |
| S2277 | Caffeic Acid | 180.16 | 331-39-5 | Others | 36 | 199.8223801 | <1 |  | C9H8O4 |
| S7414 | Caffeic Acid Phenethyl Ester | 284.31 | 104594-70-9 | NF-κB | 57 | 200.4853857 | <1 |  | C17H16O4 |
| S2226 | CAL-101 (Idelalisib, GS-1101) | 415.42 | 870281-82-6 | PI3K | 83 | 199.797795 | <1 |  | C22H18FN7O |
| S1469 | Calcifediol | 400.64 | 19356-17-3 | Others | 80 | 199.68 | <1 |  | C27H44O2 |
| S7396 | *Calpeptin* | *362.46* | 117591-20-5 | Cysteine Protease | 72 | 198.6426088 | <1 |  | C20H30N2O4 |
| S2874 | Camostat Mesilate | 494.52 | 59721-29-8 | HCV Protease | 99 | 200.1941276 | 10 | 20.22162905 | C21H26N4O8S |
| S2760 | Canagliflozin | 444.52 | 842133-18-0 | SGLT | 88 | 197.9663457 | <1 |  | C24H25FO5S |
| S1578 | Candesartan | 440.45 | 139481-59-7 | RAAS | 88 | 199.7956635 | <1 |  | C24H20N6O3 |
| S2037 | Candesartan Cilexetil | 610.66 | 145040-37-5 | Others | 122 | 199.7838404 | <1 |  | C33H34N6O6 |
| S1156 | Capecitabine | 359.35 | 154361-50-9 | DNA/RNA Synthesis | 72 | 200.3617643 | 6 | 16.69681369 | C15H22FN3O6 |
| S2051 | Captopril | 217.29 | 62571-86-2 | RAAS | 43 | 197.8922178 | 2 | 9.204289199 | C9H15NO3S |
| S1693 | Carbamazepine | 236.27 | 298-46-4 | Others | 47 | 198.9249587 | <1 |  | C15H12N2O |
| S3000 | Carbazochrome sodium sulfonate (AC-17) | 322.27 | 51460-26-5 | Others | 64 | 198.5912434 | 3 | 9.308964533 | C10H11N4NaO5S |
| S3179 | Carbenicillin disodium | 422.36 | 4800-94-6 | Others | 5 | 11.83824226 | 84 | 198.8824699 | C17H16N2Na2O6S |
| S1891 | Carbidopa | 226.23 | 28860-95-9 | others | 4 | 17.68112098 | <1 |  | C10H14N2O4 |
| S4048 | Carbimazole | 186.23 | 22232-54-8 | Others | 38 | 204.0487569 | <1 |  | C7H10N2O2S |
| S2853 | Carfilzomib (PR-171) | 719.91 | 868540-17-4 | Proteasome | 50 | 69.45312609 | <1 |  | C40H57N5O7 |
| S1289 | Carmofur | 257.26 | 61422-45-5 | Antimetabolites | 52 | 202.1301407 | <1 |  | C11H16FN3O3 |
| S4136 | Carprofen | 273.71 | 53716-49-7 | Others | 55 | 200.9426035 | <1 |  | C15H12ClNO2 |
| S4278 | Carteolol HCl | 328.83 | 51781-21-6 | Adrenergic Receptor | 65 | 197.6705288 | 65 | 197.6705288 | C16H25ClN2O3 |
| S1831 | Carvedilol | 406.47 | 72956-09-3 | Adrenergic Receptor | 81 | 199.2766994 | <1 |  | C24H26N2O4 |
| S3073 | Caspofungin Acetate | 1213.42 | 179463-17-3 | Others | 100 | 82.41169587 | 100 | 82.41169587 | C56H96N10O19 |
| S3202 | Catharanthine | 336.43 | 2468-21-5 | Others | 67 | 199.1498975 | <1 |  | C21H24N2O2 |
| S2847 | Cathepsin Inhibitor 1 | 401.89 | 225120-65-0 | Others | 80 | 199.0594441 | <1 |  | C20H24ClN5O2 |
| S2682 | CAY10505 | 289.28 | 1218777-13-9 | PI3K | 58 | 200.50 | <1 |  | C14H8FNO3S |
| S7596 | *CAY10603* | *446.5* | 1045792-66-2 | HDAC | 89 | 199.3281075 | <1 |  | C22H30N4O6 |
| S7655 | *CB-839* | *571.57* | 1439399-58-2 | Others | 100 | 174.9566982 | <1 |  | C26H24F3N7O3S |
| S2901 | CCG 50014 | 316.35 | 883050-24-6 | Others | 63 | 199.1465149 | <1 |  | C16H13FN2O2S |
| S7719 | *CCG-1423* | *454.75* | 285986-88-1 | Rho | 90 | 197.9109401 | <1 |  | C18H13ClF6N2O3 |
| S2635 | CCT128930 | 341.84 | 885499-61-6 | Akt | 68 | 198.923473 | <1 |  | C18H20ClN5 |
| S1017 | Cediranib (AZD2171) | 450.51 | 288383-20-0 | VEGFR, Flt | 90 | 199.7735899 | <1 |  | C25H27FN4O3 |
| S1605 | Cefdinir | 395.41 | 91832-40-5 | Others | 79 | 199.7926203 | <1 |  | C14H13N5O5S2 |
| S1768 | Cefditoren Pivoxil | 620.72 | 117467-28-4 | 5-alpha Reductase | 100 | 161.103235 | <1 |  | C25H28N6O7S3 |
| S1611 | Cefoperazone | 645.67 | 62893-19-0 | Others | 100 | 154.8778788 | <1 |  | C25H27N9O8S2 |
| S2543 | Ceftiofur HCl | 560.02 | 103980-44-5 | Others | 112 | 199.9928574 | <1 |  | C19H18ClN5O7S3 |
| S1290 | *Celastrol* | *450.61* | 34157-83-0 | Others | 90 | 199.7292559 | <1 |  | C29H38O4 |
| S1261 | Celecoxib | 381.37 | 169590-42-5 | COX | 76 | 199.2815376 | <1 |  | C17H14F3N3O2S |
| S1157 | CEP-18770 (Delanzomib) | 413.28 | 847499-27-8 | Proteasome | 83 | 200.8323655 | <1 |  | C21H28BN3O5 |
| S8015 | CEP-32496 | 517.46 | 1188910-76-0 | Raf | 9 | 17.39264871 | <1 |  | C24H22F3N5O5 |
| S2806 | CEP-33779 | 462.57 | 1257704-57-6 | JAK | 93 | 201.0506518 | <1 |  | C24H26N6O2S |
| S1502 | Cephalexin | 347.39 | 15686-71-2 | Others | 2 | 5.757218112 | 10 | 28.78609056 | C16H17N3O4S |
| S2408 | Cephalomannine | 831.9 | 71610-00-9 | Others | 100 | 120.2067556 | <1 |  | C45H53NO14 |
| S4238 | Cepharanthine | 606.71 | 481-49-2 | Others | 100 | 164.8233917 | <1 |  | C37H38N2O6 |
| S7634 | *Cerdulatinib (PRT062070, PRT2070)* | *482* | 1369761-01-2 | JAK | 43 | 89.21161826 | <1 |  | C20H28ClN7O3S |
| S1291 | Cetirizine DiHCl | 461.81 | 83881-52-1 | Histamine Receptor | 92 | 199.2161278 | 92 | 199.2161278 | C21H27Cl3N2O3 |
| S4242 | Cetrimonium Bromide (CTAB) | 364.45 | 57-09-0 | Others | 5 | 13.71930306 | 100 | 274.3860612 | C19H42BrN |
| S4172 | Cetylpyridinium Chloride | 339.99 | 123-03-5 | Others | 68 | 200.0058825 | 68 | 200.0058825 | C21H38ClN |
| S7139 | CFTRinh-172 | 409.4 | 307510-92-5 | CFTR | 82 | 200.2931119 | <1 |  | C18H10F3NO3S2 |
| S7051 | CGI1746 | 579.69 | 910232-84-7 | BTK | 100 | 172.5059946 | <1 |  | C34H37N5O4 |
| S7136 | CGK 733 | 555.84 | 905973-89-9 | ATM/ATR | 100 | 179.9078872 | <1 |  | C23H18Cl3FN4O3S |
| S7421 | CGP 57380 | 244.23 | 522629-08-9 | Others | 48 | 196.5360521 | <1 |  | C11H9FN6 |
| S2153 | CGS 21680 HCl | 535.98 | 124431-80-7 | 5-alpha Reductase | 107 | 199.6343147 | <1 |  | C23H30ClN7O6 |
| S7711 | *CH-223191* | *333.39* | 301326-22-7 | Others | 66 | 197.9663457 | <1 |  | C19H19N5O |
| S2699 | CH5132799 | 377.42 | 1007207-67-1 | PI3K, mTOR | 12 | 31.79481744 | <1 |  | C15H19N7O3S |
| S7340 | CH5138303 | 415.9 | 959763-06-5 | HSP (e.g. HSP90) | 83 | 199.5672037 | <1 |  | C19H18ClN5O2S |
| S7665 | *CH5183284 (Debio-1347)* | *356.38* | 1265229-25-1 | FGFR | 71 | 199.2255458 | <1 |  | C20H16N6O |
| S1843 | Chenodeoxycholic Acid | 392.57 | 474-25-9 | NULL | 79 | 201.2379958 | <1 |  | C24H40O4 |
| S2683 | CHIR-124 | 419.91 | 405168-58-3 | Chk | 7 | 16.67023886 | <1 |  | C23H22ClN5O |
| S1263 | *CHIR-99021 (CT99021)* | *465.34* | 252917-06-9 | GSK-3 | 78 | 167.6193751 | <1 |  | C22H18Cl2N8 |
| S2924 | CHIR-99021 (CT99021) HCl | 501.8 | 252917-06-9 (free base) | PI3K/Akt/mTOR | 93 | 185.3328019 | <1 |  | C22H19Cl3N8 |
| S1677 | Chloramphenicol | 323.13 | 56-75-7 | Others | 65 | 201.1574289 | <1 |  | C11H12Cl2N2O5 |
| S3067 | Chlorhexidine HCl | 578.37 | 3697-42-5 | Others | 100 | 172.8997009 | <1 |  | C22H32Cl4N10 |
| S2021 | Chlormezanone | 273.74 | 80-77-3 | Others | 55 | 200.9205816 | <1 |  | C11H12ClNO3S |
| S4288 | Chloroambucil | 304.21 | 305-03-3 | DNA/RNA Synthesis | 60 | 197.2321751 | <1 |  | C14H19Cl2NO2 |
| S4209 | Chlorocresol | 142.58 | 59-50-7 | Others | 29 | 203.3945855 | <1 |  | C7H7ClO |
| S2280 | Chlorogenic Acid | 354.31 | 327-97-9 | Others | 71 | 200.3894894 | 18 | 50.80296915 | C16H18O9 |
| S4284 | Chloroprocaine HCl | 307.22 | 3858-89-7 | Others | 61 | 198.5547816 | 61 | 198.5547816 | C13H20Cl2N2O2 |
| S1641 | Chlorothiazide | 295.72 | 58-94-6 | Others | 59 | 199.5130529 | <1 |  | C7H6ClN3O4S2 |
| S1839 | Chloroxine | 214.05 | 773-76-2 | Others | 43 | 200.8876431 | <1 |  | C9H5Cl2NO |
| S1816 | Chlorpheniramine Maleate | 390.86 | 113-92-8 | Histamine Receptor | 78 | 199.5599447 | 78 | 199.5599447 | C20H23ClN2O4 |
| S2456 | Chlorpromazine HCl | 355.33 | 69-09-0 | Dopamine Receptor, Potassium Channel | 71 | 199.8142572 | 71 | 199.8142572 | C17H20Cl2N2S |
| S4166 | Chlorpropamide | 276.74 | 94-20-2 | Others | 55 | 198.742502 | <1 |  | C10H13ClN2O3S |
| S1771 | Chlorprothixene | 315.86 | 113-59-7 | Others | 6 | 18.99575761 | <1 |  | C18H18ClNS |
| S4192 | Chlorquinaldol | 228.07 | 72-80-0 | Others | 46 | 201.6924628 | <1 |  | C10H7Cl2NO |
| S4155 | Chlorzoxazone | 169.57 | 95-25-0 | Others | 34 | 200.5071652 | <1 |  | C7H4ClNO2 |
| S4171 | Choline Chloride | 139.62 | 67-48-1 | Others | 28 | 200.5443346 | 28 | 200.5443346 | C5H14ClNO |
| S4208 | Chromocarb | 190.15 | 4940-39-0 | Others | 38 | 199.8422298 | <1 |  | C10H6O4 |
| S2281 | Chrysin | 254.24 | 480-40-0 | Others | 51 | 200.5978603 | <1 |  | C15H10O4 |
| S2406 | Chrysophanic Acid | 254.24 | 481-74-3 | EGFR, mTOR | 5 | 19.66645689 | <1 |  | C15H10O4 |
| S2818 | CI994 (Tacedinaline) | 269.3 | 112522-64-2 | HDAC | 54 | 200.5198663 | <1 |  | C15H15N3O2 |
| S2528 | Ciclopirox | 207.27 | 29342-05-0 | Others | 42 | 202.6342452 | <1 |  | C12H17NO2 |
| S3019 | Ciclopirox ethanolamine | 268.35 | 41621-49-2 | ATPase | 6 | 22.3588597 | <1 |  | C14H24N2O3 |
| S7188 | *CID755673* | *219.24* | 521937-07-5 | Others | 43 | 196.1320927 | <1 |  | C12H11NO3 |
| S2081 | Cilazapril Monohydrate | 435.51 | 92077-78-6 | RAAS | 87 | 199.77 | <1 |  | C22H33N3O6 |
| S7077 | Cilengitide | 588.66 | 188968-51-6 | Integrin | 100 | 169.8773486 | 8 | 13.59018788 | C29H41F3N8O9 |
| S1293 | Cilnidipine | 492.52 | 132203-70-4 | Calcium Channel | 99 | 201.0070657 | <1 |  | C27H28N2O7 |
| S1455 | Cilomilast | 343.42 | 153259-65-5 | PDE | 69 | 200.9201561 | <1 |  | C20H25NO4 |
| S1294 | Cilostazol | 369.46 | 73963-72-1 | PDE | 74 | 200.2923185 | <1 |  | C20H27N5O2 |
| S1845 | Cimetidine | 252.34 | 51481-61-9 | NULL | 51 | 202.1082666 | <1 |  | C10H16N6S |
| S1260 | Cinacalcet HCl | 393.87 | 364782-34-3 | CaSR | 79 | 200.5737934 | <1 |  | C22H23ClF3N |
| S2282 | Cinchonidine | 294.39 | 485-71-2 | Others | 59 | 200.4144163 | <1 |  | C19H22N2O |
| S4190 | Cinchophen | 249.26 | 132-60-5 | Others | 50 | 200.5937575 | <1 |  | C16H11NO2 |
| S3045 | Cinepazide maleate | 533.57 | 26328-04-1 | Others | 107 | 200.5360121 | 107 | 200.5360121 | C26H35N3O9 |
| S2665 | Ciprofibrate | 289.15 | 52214-84-3 | PPAR | 58 | 200.5879301 | <1 |  | C13H14Cl2O3 |
| S2813 | Ciproxifan | 386.4 | 184025-19-2 | Histamine Receptor | 54 | 139.7515528 | 1 | 2.587991718 | C20H22N2O6 |
| S2113 | Cisatracurium Besylate | 1243.48 | 96946-42-8 | Adrenergic Receptor | 249 | 200.2444752 | 47 | 37.79714993 | C65H82N2O18S2 |
| S7497 | CK-636 | 284.38 | 442632-72-6 | Microtubule Associated | 57 | 200.4360363 | <1 |  | C16H16N2OS |
| S7557 | *CL-387785 (EKI-785)* | *381.23* | 194423-06-8 | EGFR | 63 | 165.2545707 | <1 |  | C18H13BrN4O |
| S1199 | Cladribine | 285.69 | 4291-63-8 | DNA/RNA Synthesis | 57 | 199.5169589 | <1 |  | C10H12ClN5O3 |
| S2555 | Clarithromycin | 747.95 | 81103-11-9 | P450 |  | 10 |  |  | C38H69NO13 |
| S1847 | Clemastine Fumarate | 459.96 | 14976-57-9 | Histamine Receptor | 35 | 76.09357335 | <1 |  | C25H30ClNO5 |
| S2080 | Clevidipine Butyrate | 456.32 | 167221-71-8 | Calcium Channel | 91 | 199.4214586 | <1 |  | C21H23Cl2NO6 |
| S3001 | Clevudine | 260.22 | 163252-36-6 | DNA/RNA Synthesis | 52 | 199.83 | 52 | 199.8309123 | C10H13FN2O5 |
| S4178 | Climbazole | 292.76 | 38083-17-9 | Others | 59 | 201.5302637 | <1 |  | C15H17ClN2O2 |
| S2830 | Clindamycin | 424.98 | 18323-44-9 | Others | 85 | 200.0094122 | <1 |  | C18H33ClN2O5S |
| S2457 | Clindamycin HCl | 461.44 | 21462-39-5 | Others | 92 | 199.3758669 | 92 | 199.3758669 | C18H34Cl2N2O5S |
| S2596 | Clindamycin palmitate HCl | 699.85 | 25507-04-4 | Others | 140 | 200.0428663 | 140 | 200.0428663 | C34H64Cl2N2O6S |
| S2664 | Clinofibrate | 468.58 | 30299-08-2 | RAAS | 94 | 200.6060865 | <1 |  | C28H36O6 |
| S2584 | Clobetasol propionate | 466.97 | 25122-46-7 | Others | 93 | 199.1562627 | <1 |  | C25H32ClFO5 |
| S1218 | Clofarabine | 303.68 | 123318-82-1 | DNA/RNA Synthesis | 60 | 197.5763962 | <1 |  | C10H11ClFN5O3 |
| S4107 | Clofazimine | 473.4 | 2030-63-9 | Others | 5 | 10.56189269 | <1 |  | C27H22Cl2N4 |
| S4207 | Clofibric Acid | 214.65 | 882-09-7 | Others | 43 | 200.3261123 | <1 |  | C10H11ClO3 |
| S2561 | Clomifene citrate | 598.08 | 50-41-9 | Estrogen/progestogen Receptor | 120 | 200.6420546 | <1 |  | C32H36ClNO8 |
| S2541 | Clomipramine HCl | 351.31 | 17321-77-6 | 5-HT Receptor | 70 | 199.2542199 | 70 | 199.2542199 | C19H24Cl2N2 |
| S2458 | Clonidine HCl | 266.5 | 4205-91-8 | Adrenergic Receptor | 53 | 198.8742964 | 53 | 198.8742964 | C9H10Cl3N3 |
| S1415 | Clopidogrel | 419.9 | 120202-66-6 | P2 Receptor | 83 | 197.666111 | 78 | 185.7585139 | C16H18ClNO6S2 |
| S4135 | Clorprenaline HCl | 250.16 | 6933-90-0 | Others | 50 | 199.8720819 | 50 | 199.8720819 | C11H17Cl2NO |
| S2613 | Clorsulon | 380.66 | 60200-06-8 | Others | 76 | 199.6532339 | <1 |  | C8H8Cl3N3O4S2 |
| S4106 | Closantel | 663.07 | 57808-65-8 | Others | 100 | 150.8136396 | <1 |  | C22H14Cl2I2N2O2 |
| S4105 | Closantel Sodium | 685.06 | 61438-64-0 | Others | 100 | 145.9726155 | <1 |  | C22H13Cl2I2N2NaO2 |
| S1606 | Clotrimazole | 344.84 | 23593-75-1 | Others | 11 | 31.89885164 | <1 |  | C22H17ClN2 |
| S2564 | Cloxacillin Sodium | 475.88 | 7081-44-9 | Others | 55 | 115.5753551 | 55 | 115.5753551 | C19H19ClN3NaO6S |
| S2459 | Clozapine | 326.82 | 5786-21-0 | 5-HT Receptor | 65 | 198.8862371 | <1 |  | C18H19ClN4 |
| S7206 | CNX-2006 | 545.53 | 1375465-09-0 | EGFR | 100 | 183.3079757 | <1 |  | C26H27F4N7O2 |
| S7257 | CNX-774 | 499.5 | 1202759-32-7 | BTK | 100 | 200.2002002 | <1 |  | C26H22FN7O3 |
| S7284 | CO-1686 (AVL-301) | 555.55 | 1374640-70-6 | EGFR | 100 | 180.0018 | <1 |  | C27H28F3N7O3 |
| S2900 | Cobicistat (GS-9350) | 776.02 | 1004316-88-4 | P450 (e.g. CYP17) | 100 | 128.8626582 | <1 |  | C40H53N7O5S2 |
| S8041 | *Cobimetinib (GDC-0973, RG7420)* | *531.31* | 934660-93-2 | MEK | 100 | 188.214037 | <1 |  | C21H21F3IN3O2 |
| S7783 | *Combretastatin A4* | *316.35* | 117048-59-6 | Microtubule Associated | 63 | 199.1465149 | <1 |  | C18H20O5 |
| S2116 | Conivaptan HCl | 535.04 | 168626-94-6 | Others | 107 | 199.9850478 | <1 |  | C32H27ClN4O2 |
| S7448 | CORM-3 | 294.61 | 475473-26-8 | Others | 38 | 128.9840806 | <1 |  | C5H6ClNO5RRu |
| S2559 | Cortisone acetate | 402.48 | 50-04-4 | Others | 7 | 17.39216855 | <1 |  | C23H30O6 |
| S1319 | Costunolide | 232.32 | 553-21-9 | Telomerase | 47 | 202.3071625 | <1 |  | C15H20O2 |
| S4170 | Coumarin | 146.14 | 91-64-5 | Others | 29 | 198.4398522 | <1 |  | C9H6O2 |
| S1536 | CP-673451 | 417.5 | 343787-29-1 | Others | 28 | 67.06586826 | <1 |  | C24H27N5O2 |
| S1167 | CP-724714 | 469.53 | 537705-08-1 | EGFR, HER2 | 94 | 200.2002002 | <1 |  | C27H27N5O3 |
| S2717 | CP-91149 | 399.87 | 186392-40-5 | Phosphorylase | 80 | 200.0650211 | <1 |  | C21H22ClN3O3 |
| S7616 | *CPI-169* | *528.66* | 1450655-76-1 | Histone Methyltransferase | 100 | 189.1574925 | <1 |  | C27H36N4O5S |
| S7304 | CPI-203 | 399.9 | 1446144-04-2 | Epigenetic Reader Domain | 79 | 197.5493873 | <1 |  | C19H18ClN5OS |
| S7656 | *CPI-360* | *437.53* | 1802175-06-9 | Histone Methyltransferase | 20 | 45.71115124 | <1 |  | C25H31N3O4 |
| S2776 | CPI-613 | 388.59 | 95809-78-2 | Dehydrogenase | 78 | 200.7257006 | <1 |  | C22H28O2S2 |
| S2730 | Crenolanib (CP-868596) | 443.54 | 670220-88-9 | PDGFR | 89 | 200.6583397 | <1 |  | C26H29N5O2 |
| S1068 | Crizotinib (PF-02341066) | 450.34 | 877399-52-5, 877399-53-6 (acetate) | c-Met, ALK | 9 | 19.9849003 | <1 |  | C21H22Cl2FN5O |
| S7449 | CRT0044876 | 206.15 | 6960-45-8 | DNA/RNA Synthesis | 41 | 198.8843075 | <1 |  | C9H6N2O4 |
| S2285 | Cryptotanshinone | 296.36 | 35825-57-1 | STAT | 5 | 16.87137265 | <1 |  | C19H20O3 |
| S1917 | Crystal Violet | 407.98 | 548-62-9 | Others | 82 | 200.9902446 | 14 | 34.31540762 | C25H30ClN3 |
| S2861 | CTEP (RO4956371) | 391.77 | 871362-31-1 | GluR | 78 | 199.0964086 | <1 |  | C19H13ClF3N3O |
| S1194 | CUDC-101 | 434.49 | 1012054-59-9 | HDAC, EGFR, HER2 | 20 | 46.03097885 | <1 |  | C24H26N4O4 |
| S2759 | CUDC-907 | 508.55 | 1339928-25-4 | HDAC, PI3K | 102 | 200.5702487 | <1 |  | C23H24N8O4S |
| S1848 | Curcumin | 368.38 | 458-37-7 | Others | 74 | 200.8795266 | <1 |  | C21H20O6 |
| S2407 | Curcumol | 236.35 | 4871-97-0 | Others | 47 | 198.8576264 | <1 |  | C15H24O2 |
| S7336 | CW069 | 500.33 | 1594094-64-0 | Microtubule Associated | 100 | 199.8680871 | <1 |  | C23H21IN2O3 |
| S2248 | CX-4945 (Silmitasertib) | 349.77 | 1009820-21-6 | PKC | 16 | 45.74434628 | <1 |  | C19H12ClN3O2 |
| S7041 | CX-6258 HCl | 498.4 | 1353859-00-3 | Pim | 57 | 114.3659711 | 89 | 178.5714286 | C26H25Cl2N3O3 |
| S1171 | CYC116 | 368.46 | 693228-63-6 | Aurora Kinase, VEGFR | 24 | 65.13597134 | <1 |  | C18H20N6OS |
| S4015 | Cyclamic acid | 179.24 | 100-88-9 | Others | 36 | 200.848025 | 36 | 200.848025 | C6H13NO3S |
| S4189 | Cyclandelate | 276.37 | 456-59-7 | Others | 55 | 199.0085755 | <1 |  | C17H24O3 |
| S4139 | Cyclizine 2HCl | 339.3 | 5897-18-7 | Histamine Receptor | 7 | 20.63071029 | 68 | 200.4126142 | C18H24Cl2N2 |
| S7834 | *Cyclo (-RGDfK)* | *717.69* | 161552-03-0 | Integrin | 100 | 139.335925 | 84 | 117.042177 | C29H42F3N9O9 |
| S7844 | *Cyclo(RGDyK)* | *847.72* | 250612-42-1 | Integrin | 100 | 117.9634785 | 100 | 117.9634785 | C31H45F6N9O12 |
| S4283 | Cyclobenzaprine HCl | 311.85 | 6202-23-9 | Others | 62 | 198.8135321 | 62 | 198.8135321 | C20H22ClN |
| S1973 | Cyclocytidine HCl | 261.66 | 10212-25-6 | Others | 52 | 198.7311779 | 44 | 168.1571505 | C9H12ClN3O4 |
| S2057 | *Cyclophosphamide Monohydrate* | *279.1* | 6055-19-2 | Others | 55 | 197.061985 | 7 | 25.08061627 | C7H17Cl2N2O3P |
| S2286 | Cyclosporin A | 1202.61 | 59865-13-3 | Others | 100 | 83.1524767 | <1 |  | C62H111N11O12 |
| S1514 | Cyclosporine | 1202.61 | 79217-60-0 | Others | 100 | 83.1524767 | <1 |  | C62H111N11O12 |
| S2044 | Cyproheptadine HCl | 323.86 | 969-33-5 | Histamine Receptor | 65 | 200.7040079 | <1 |  | C21H22ClN |
| S2042 | Cyproterone Acetate | 416.94 | 427-51-0 | Androgen Receptor/P450 | 83 | 199.0694105 | <1 |  | C24H29ClO4 |
| S4167 | Cyromazine | 166.18 | 66215-27-8 | Others | 33 | 198.5798532 | 1 | 6.017571308 | C6H10N6 |
| S4206 | Cysteamine HCl | 113.61 | 156-57-0 | Others | 23 | 202.4469677 | 23 | 202.4469677 | C2H8ClNS |
| S2219 | CYT387 | 414.46 | 1056634-68-4 | JAK | 74 | 178.5455774 | <1 |  | C23H22N6O2 |
| S2195 | CYT997 (Lexibulin) | 434.53 | 917111-44-5 | Microtubule Associated | 86 | 197.9149886 | <1 |  | C24H30N6O2 |
| S2053 | Cytidine | 243.22 | 65-46-3 | Others | 49 | 201.4636954 | 49 | 201.4636954 | C9H13N3O5 |
| S2287 | Cytisine | 190.24 | 485-35-8 | Others | 38 | 199.7476871 | 38 | 199.7476871 | C11H14N2O |
| S7018 | CZC24832 | 364.4 | 1159824-67-5 | PI3Kγ | 5 | 13.72118551 | <1 |  | C15H17FN6O2S |
| S7642 | *D 4476* | *398.41* | 301836-43-1 | Others | 79 | 198.2881956 | <1 |  | C23H18N4O3 |
| S2154 | Dabigatran Etexilate | 627.73 | 211915-06-9 | Others | 126 | 200.7232409 | <1 |  | C34H41N7O5 |
| S2807 | Dabrafenib (GSK2118436) | 519.56 | 1195765-45-7 | Raf | 30 | 57.7411656 | <1 |  | C23H20F3N5O2S2 |
| S1221 | Dacarbazine | 182.18 | 4342-03-4 | DNA/RNA Synthesis | 3 | 16.46723021 | <1 |  | C6H10N6O |
| S1482 | Daclatasvir (BMS-790052) | 738.88 | 1009119-64-5 | HCV Protease | 148 | 200.3031615 | <1 |  | C40H50N8O6 |
| S2727 | Dacomitinib (PF299804, PF299) | 469.94 | 1110813-31-4 | EGFR | 19 | 40.43069328 | <1 |  | C24H25ClFN5O2 |
| S1849 | Daidzein | 254.24 | 486-66-8 | Others | 51 | 200.5978603 | <1 |  | C15H10O4 |
| S2772 | Dalcetrapib (JTT-705, RO4607381) | 389.59 | 211513-37-0 | CETP | 78 | 200.2104777 | <1 |  | C23H35NO2S |
| S1183 | Danoprevir (ITMN-191) | 731.83 | 850876-88-9, 916881-67-9, 1001913-18-3, 1225266-12-5 | Proteasome, HCV Protease | 144 | 196.7670087 | <1 |  | C35H46FN5O9S |
| S1107 | Danusertib (PHA-739358) | 474.55 | 827318-97-8 | Aurora Kinase, FGFR, Bcr-Abl, c-RET, Src | 95 | 200.1896534 | <1 |  | C26H30N6O3 |
| S1548 | Dapagliflozin | 408.87 | 461432-26-8 | SGLT | 82 | 200.5527429 | <1 |  | C21H25ClO6 |
| S2554 | Daphnetin | 178.14 | 486-35-1 | DNA/RNA Synthesis | 35 | 196.47 | <1 |  | C9H6O4 |
| S2914 | Dapivirine (TMC120) | 329.4 | 244767-67-7 | Reverse Transcriptase | 34 | 103.2179721 | <1 |  | C20H19N5 |
| S1869 | Dapoxetine HCl | 341.87 | 129938-20-1 | 5-HT Receptor | 68 | 198.9060169 | 68 | 198.9060169 | C21H24ClNO |
| S2215 | DAPT (GSI-IX) | 432.46 | 208255-80-5 | Gamma-secretase, Beta Amyloid | 86 | 198.8623225 | <1 |  | C23H26F2N2O4 |
| S7520 | Darapladib (SB-480848) | 666.77 | 356057-34-6 | Phospholipase (e.g. PLA) | 100 | 149.9767536 | <1 |  | C36H38F4N4O2S |
| S3144 | Darifenacin HBr | 507.46 | 133099-07-7 | AChR | 117 | 230.5600441 | <1 |  | C28H31BrN2O2 |
| S1620 | Darunavir Ethanolate | 593.73 | 635728-49-3 | HIV Protease | 100 | 168.426726 | <1 |  | C29H43N3O8S |
| S1021 | Dasatinib | 488.01 | 302962-49-8 | Src, Bcr-Abl, c-Kit | 98 | 200.8155571 | <1 |  | C22H26ClN7O2S |
| S7782 | *Dasatinib Monohydrate* | *506.02* | 863127-77-9 | Src | 21 | 41.50033596 | <1 |  | C22H28ClN7O3S |
| S3035 | Daunorubicin HCl | 563.98 | 23541-50-6 | Telomerase | 100 | 177.3112522 | 100 | 177.3112522 | C27H30ClNO10 |
| S7199 | DBeQ | 340.42 | 177355-84-9 | Others | 68 | 199.753246 | <1 |  | C22H20N4 |
| S2634 | DCC-2036 (Rebastinib) | 553.59 | 1020172-07-9 | Bcr-Abl | 111 | 200.5094023 | <1 |  | C30H28FN7O3 |
| S4072 | Decamethonium Bromide | 418.29 | 541-22-0 | AChR | 34 | 81.28332018 | 84 | 200.8176146 | C16H38Br2N2 |
| S1200 | Decitabine | 228.21 | 2353-33-5 | DNA/RNA Synthesis | 45 | 197.1868016 | 10 | 43.81928925 | C8H12N4O4 |
| S1712 | Deferasirox | 373.36 | 201530-41-8 | Others | 74 | 198.2001286 | <1 |  | C21H15N3O4 |
| S1888 | Deflazacort | 441.52 | 14484-47-0 | Others | 88 | 199.3114695 | <1 |  | C25H31NO6 |
| S2243 | Degrasyn (WP1130) | 384.27 | 856243-80-6 | DUB, Bcr-Abl | 77 | 200.3799412 | <1 |  | C19H18BrN3O |
| S2604 | Dehydroepiandrosterone (DHEA) | 288.43 | 53-43-0 | Androgen Receptor | 57 | 197.6216066 | <1 |  | C19H28O2 |
| S7224 | Deltarasin | 603.75 | 1440898-61-2 | PDE | 100 | 165.63147 | <1 |  | C40H37N5O |
| S4279 | Demeclocycline HCl | 501.31 | 64-73-3 | Others | 100 | 199.4773693 | 26 | 51.86411602 | C21H22Cl2N2O8 |
| S4132 | Deoxyarbutin | 194.23 | 53936-56-4 | Others | 39 | 200.7928744 | <1 |  | C11H14O3 |
| S4243 | *Deoxycorticosterone acetate* | *372.5* | 56-47-3 | Others | 11 | 29.53020134 | <1 |  | C23H32O4 |
| S4012 | Desloratadine | 310.82 | 100643-71-8 | Histamine Receptor | 26 | 83.64970079 | <1 |  | C19H19ClN2 |
| S1701 | Desonide | 416.51 | 638-94-8 | Others | 83 | 199.2749274 | <1 |  | C24H32O6 |
| S4113 | Desvenlafaxine | 263.38 | 93413-62-8 | 5-HT Receptor | 37 | 140.4814337 | <1 |  | C16H25NO2 |
| S4112 | Desvenlafaxine Succinate | 381.46 | 386750-22-7 | 5-HT Receptor | 80 | 209.7205474 | <1 |  | C20H31NO6 |
| S2092 | Detomidine HCl | 222.71 | 90038-01-0 | Adrenergic Receptor | 45 | 202.056486 | 45 | 202.056486 | C12H15ClN2 |
| S1322 | Dexamethasone (DHAP) | 392.46 | 50-02-2 | IL Receptor | 79 | 201.2943994 | <1 |  | C22H29FO5 |
| S3124 | *Dexamethasone Acetate* | *434.5* | 1177-87-3 | Others | 87 | 200.2301496 | <1 |  | C24H31FO6 |
| S4099 | Dexlansoprazole | 369.36 | 138530-94-6 | Others | 74 | 200.3465454 | 74 | 200.3465454 | C16H14F3N3O2S |
| S3075 | Dexmedetomidine | 200.28 | 113775-47-6 | Adrenergic Receptor | 40 | 199.7203915 | 40 | 199.7203915 | C13H16N2 |
| S2090 | Dexmedetomidine HCl (Precedex) | 236.74 | 145108-58-3 | Androgen Receptor | 48 | 202.7540762 | <1 |  | C13H17ClN2 |
| S1222 | Dexrazoxane HCl (ICRF-187, ADR-529) | 304.73 | 149003-01-0 | Others | 60 | 196.8956125 | 60 | 196.8956125 | C11H17ClN4O4 |
| S2123 | Dextrose | 180.16 | 50-99-7 | Others | 36 | 199.8223801 | 36 | 199.8223801 | C6H12O6 |
| S4267 | Diacerein | 368.29 | 13739-02-1 | Others | 48 | 130.3320753 | <1 |  | C19H12O8 |
| S4205 | Dibenzothiophene | 184.26 | 132-65-0 | Others | 37 | 200.8032129 | <1 |  | C12H8S |
| S4038 | Dibucaine HCl | 379.92 | 61-12-1 | Sodium Channel | 76 | 200.0421141 | 76 | 200.0421141 | C20H30ClN3O2 |
| S7858 | *Dibutyryl-cAMP (Bucladesine)* | *491.37* | 16980-89-5 | PKA | 98 | 199.4423754 | 98 | 199.4423754 | C18H23N5NaO8P |
| S2177 | Dichlorphenamide | 305.16 | 120-97-8 | Carbonic Anhydrase | 61 | 199.90 | <1 |  | C6H6Cl2N2O4S2 |
| S2028 | Diclazuril | 407.64 | 101831-37-2 | Others | 16 | 39.25031891 | <1 |  | C17H9Cl3N4O2 |
| S3063 | Diclofenac Diethylamine | 369.29 | 78213-16-8 | Others | 74 | 200.3845216 | <1 |  | C18H22Cl2N2O2 |
| S3062 | Diclofenac Potassium | 334.24 | 15307-81-0 | Others | 69 | 206.4384873 | 10 | 29.91862135 | C14H10Cl2KNO2 |
| S1903 | Diclofenac Sodium | 318.13 | 15307-79-6 | COX | 64 | 201.17562 | 14 | 44.00716688 | C14H10Cl2NNaO2 |
| S4111 | Dicloxacillin Sodium | 510.32 | 13412-64-1 | Others | 100 | 195.9554789 | 100 | 195.9554789 | C19H18Cl2N3NaO6S |
| S4299 | Dicoumarol | 336.29 | 66-76-2 | Others | 67 | 199.232805 | <1 |  | C19H12O6 |
| S1702 | Didanosine | 236.23 | 69655-05-6 | NULL | 47 | 198.958642 | <1 |  | C10H12N4O3 |
| S1251 | Dienogest | 311.42 | 65928-58-7 | Estrogen/progestogen Receptor | 62 | 199.0880483 | <1 |  | C20H25NO2 |
| S1859 | Diethylstilbestrol | 268.35 | 56-53-1 | Others | 54 | 201.2297373 | <1 |  | C18H20O2 |
| S4095 | Difluprednate | 508.55 | 23674-86-4 | Others | 102 | 200.5702487 | <1 |  | C27H34F2O7 |
| S4290 | Digoxin | 780.94 | 20830-75-5 | Sodium Channel | 100 | 128.0508106 | <1 |  | C41H64O14 |
| S2290 | Dihydroartemisinin (DHA) | 284.35 | 71939-50-9 | Others | 56 | 196.94 | <1 |  | C15H24O5 |
| S2399 | Dihydromyricetin | 320.25 | 27200-12-0 | GABA Receptor | 64 | 199.84 | <1 |  | C15H12O8 |
| S1865 | Diltiazem HCl | 450.98 | 33286-22-5 | Others | 90 | 199.5653909 | 90 | 199.5653909 | C22H27ClN2O4S |
| S1201 | Dimesna | 326.34 | 16208-51-8 | Others | 65 | 199.1787706 | 65 | 199.1787706 | C4H8Na2O6S4 |
| S2586 | Dimethyl Fumarate | 144.13 | 624-49-7 | Others | 29 | 201.2072435 | <1 |  | C6H8O4 |
| S4104 | Diminazene Aceturate | 515.52 | 908-54-3 | Others | 19 | 36.85599007 | 100 | 193.9788951 | C22H29N9O6 |
| S2768 | Dinaciclib (SCH727965) | 396.49 | 779353-01-4 | CDK | 26 | 65.57542435 | <1 |  | C21H28N6O2 |
| S4141 | Dinitolmide | 225.16 | 148-01-6 | Others | 45 | 199.8578788 | <1 |  | C8H7N3O5 |
| S2379 | Dioscin | 869.04 | 19057-60-4 | Others | 100 | 115.069502 | <1 |  | C45H72O16 |
| S2380 | Diosmetin | 300.26 | 520-34-3 | Others | 60 | 199.8268168 | <1 |  | C16H12O6 |
| S4034 | Diphemanil Methylsulfate | 389.51 | 62-97-5 | AChR | 25 | 64.18320454 | 28 | 71.88518908 | C21H27NO4S |
| S1866 | Diphenhydramine HCl | 291.82 | 147-24-0 | Others | 58 | 198.7526557 | 58 | 198.7526557 | C17H22ClNO |
| S4292 | Diphenidol HCl | 345.91 | 3254-89-5 | AChR | 69 | 199.4738516 | 13 | 37.58203001 | C21H28ClNO |
| S1895 | Dipyridamole | 504.63 | 58-32-2 | Others | 101 | 200.1466421 | <1 |  | C24H40N8O4 |
| S4213 | Dirithromycin | 835.07 | 62013-04-1 | Others | 11 | 13.17254841 | <1 |  | C42H78N2O14 |
| S1680 | Disulfiram | 296.54 | 97-77-8 | Others | 59 | 198.9613543 | <1 |  | C10H20N2S4 |
| S1703 | Divalproex Sodium | 310.41 | 76584-70-8 | Others | 62 | 199.7358333 | 62 | 199.7358333 | C16H31NaO4 |
| S2293 | DL-Carnitine HCl | 197.66 | 461-05-2 | Others | 40 | 202.3677021 | 40 | 202.3677021 | C7H16ClNO3 |
| S2381 | D-Mannitol | 182.17 | 69-65-8 | Others | 36 | 197.6176099 | 36 | 197.6176099 | C6H14O6 |
| S7146 | DMH1 | 380.44 | 1206711-16-1 | TGF-beta/Smad | 22 | 57.82777836 | <1 |  | C24H20N4O |
| S1537 | DMXAA (Vadimezan) | 282.29 | 117570-53-3 | VDA | 7 | 24.79719437 | <1 |  | C17H14O4 |
| S1148 | Docetaxel | 807.88 | 114977-28-5 | Microtubule Associated | 100 | 123.7807595 | <1 |  | C43H53NO14 |
| S7787 | *Docetaxel Trihydrate* | *861.93* | 148408-66-6 | Microtubule Associated | 100 | 116.0187022 | <1 |  | C43H59NO17 |
| S1658 | Dofetilide | 441.56 | 115256-11-6 | Others | 88 | 199.2934143 | <1 |  | C19H27N3O5S2 |
| S2667 | Dolutegravir (GSK1349572) | 419.38 | 1051375-16-6 | Integrase | 83 | 197.9112023 | <1 |  | C20H19F2N3O5 |
| S4186 | Domiphen Bromide | 414.46 | 538-71-6 | Others | 83 | 200.26058 | 83 | 200.26058 | C22H40BrNO |
| S2461 | Domperidone | 425.91 | 57808-66-9 | Dopamine Receptor | 48 | 112.6998662 | <1 |  | C22H24ClN5O2 |
| S2529 | Dopamine HCl | 189.64 | 62-31-7 | Dopamine Receptor | 38 | 200.3796667 | 38 | 200.3796667 | C8H12ClNO2 |
| S1374 | Doripenem Hydrate | 438.52 | 364622-82-2 | Others | 36 | 82.09431725 | 26 | 59.29034024 | C15H26N4O7S2 |
| S2769 | Dovitinib (TKI-258) Dilactic Acid | 572.59 | 852433-84-2 | Flt, FGFR, PDGFR, VEGFR, c-Kit | 90 | 157.1805306 | 70 | 122.2515238 | C27H33FN6O7 |
| S7765 | *Dovitinib (TKI258) Lactate* | *482.51* | 915769-50-5 | FLT3 | 100 | 207.2495907 | 66 | 136.7847299 | C24H27FN6O4 |
| S1018 | Dovitinib (TKI-258, CHIR-258) | 392.43 | 405169-16-6 | c-Kit, FGFR, Flt, VEGFR, PDGFR | 30 | 76.44675484 | <1 |  | C21H21FN6O |
| S4037 | Doxapram HCl | 432.98 | 7081-53-0 | Others | 87 | 200.9330685 | 25 | 57.7393875 | C24H33ClN2O3 |
| S1324 | Doxazosin Mesylate | 547.58 | 77883-43-3 | Adrenergic Receptor | 15 | 27.39325761 | <1 |  | C24H29N5O8S |
| S1467 | Doxercalciferol | 412.65 | 54573-75-0 | Others | 83 | 201.1389798 | <1 |  | C28H44O2 |
| S2045 | Doxifluridine | 246.19 | 3094-09-5 | Others | 49 | 199.033267 | 49 | 199.033267 | C9H11FN2O5 |
| S4164 | Doxofylline | 266.25 | 69975-86-6 | Others | 53 | 199.0610329 | 24 | 90.14084507 | C11H14N4O4 |
| S1208 | Doxorubicin (Adriamycin) | 579.98 | 25316-40-9 | Topoisomerase | 100 | 172.4197386 | 20 | 34.48394772 | C27H30ClNO11 |
| S4005 | Doxycycline HCl | 480.9 | 10592-13-9 | MMP |  | 10 |  |  | C22H25ClN2O8 |
| S4163 | Doxycycline Hyclate | 512.94 | 24390-14-5 | Others | 100 | 194.9545756 | 100 | 194.9545756 | C46H58Cl2N4O18 |
| S4240 | Doxylamine Succinate | 388.46 | 562-10-7 | Others | 78 | 200.7928744 | 78 | 200.7928744 | C21H28N2O5 |
| S7909 | *Dp44mT* | *285.37* | 152095-12-0 | Others | 57 | 199.7406875 | <1 |  | C14H15N5S |
| S2114 | Dronedarone HCl | 593.22 | 141625-93-6 | Others | 80 | 134.8572199 | <1 |  | C31H45ClN2O5S |
| S4096 | Droperidol | 379.43 | 548-73-2 | Others | 76 | 200.3004507 | <1 |  | C22H22FN3O2 |
| S4138 | Dropropizine | 236.31 | 17692-31-8 | Others | 47 | 198.8912869 | 11 | 46.54902459 | C13H20N2O2 |
| S1377 | Drospirenone | 366.49 | 67392-87-4 | Estrogen/progestogen Receptor | 73 | 199.186881 | <1 |  | C24H30O3 |
| S1422 | Droxinostat | 243.69 | 99873-43-5 | HDAC | 49 | 201.0751364 | <1 |  | C11H14ClNO3 |
| S7637 | *DTP3* | *525.6* | NA | JNK | 100 | 190.2587519 | 100 | 190.2587519 | C26H35N7O5 |
| S2084 | Duloxetine HCl | 333.88 | 136434-34-9 | 5-HT Receptor | 67 | 200.6708997 | <1 |  | C18H20ClNOS |
| S1202 | Dutasteride | 528.53 | 164656-23-9 | 5-alpha Reductase | 62 | 117.3064916 | <1 |  | C27H30F6N2O2 |
| S2041 | Dyclonine HCl | 325.87 | 536-43-6 | Others | 15 | 46.03062571 | 6 | 18.41225028 | C18H28ClNO2 |
| S4097 | Dydrogesterone | 312.45 | 152-62-5 | Others | 63 | 201.6322612 | <1 |  | C21H28O2 |
| S8047 | Dynasore | 322.31 | 304448-55-3 | Dynamin | 64 | 198.5665974 | <1 |  | C18H14N2O4 |
| S7163 | *Dyngo-4a* | *338.31* | 1256493-34-1 | Dynamin | 67 | 198.0432148 | <1 |  | C18H14N2O5 |
| S1504 | Dyphylline | 254.24 | 479-18-5 | PDE | 51 | 200.5978603 | 51 | 200.5978603 | C10H14N4O4 |
| S7445 | *E3330* | *378.46* | 136164-66-4 | DNA/RNA Synthesis | 75 | 198.1715373 | <1 |  | C21H30O6 |
| S7379 | E-64 | 357.41 | 66701-25-5 | Cysteine Protease | 71 | 198.6514087 | 11 | 30.77697882 | C15H27N5O5 |
| S2535 | Econazole nitrate | 444.7 | 24169-02-6 | Others | 89 | 200.1349224 | <1 |  | C18H16Cl3N3O4 |
| S1326 | Edaravone | 174.2 | 89-25-8 | Others | 35 | 200.9184845 | <1 |  | C10H10N2O |
| S7280 | Edoxaban | 548.06 | 1229194-11-9 | Factor Xa | 100 | 182.4617743 | <1 |  | C31H40ClN7O8S2 |
| S4263 | Efaproxiral Sodium | 363.38 | 170787-99-2 | Others | 73 | 200.8916286 | 73 | 200.8916286 | C20H22NNaO4 |
| S7319 | EHop-016 | 430.55 | 1380432-32-5 | Rac | 86 | 199.7445128 | <1 |  | C25H30N6O |
| S7482 | EHT 1864 | 581.47 | 754240-09-0 | Rho | 100 | 171.977918 | 100 | 171.977918 | C25H29Cl2F3N2O4S |
| S7611 | *EI1* | *390.48* | 1418308-27-6 | Histone Methyltransferase | 42 | 107.5599262 | <1 |  | C23H26N4O2 |
| S7772 | *Elacridar (GF120918)* | *563.64* | 143664-11-3 | P-gp | 41 | 72.74146618 | <1 |  | C34H33N3O5 |
| S1052 | Elesclomol (STA-4783) | 400.5 | 488832-69-5 | HSP | 80 | 199.7503121 | <1 |  | C19H20N4O2S2 |
| S3180 | Eletriptan HBr | 463.43 | 177834-92-3 | 5-HT Receptor | 93 | 200.6775565 | <1 |  | C22H27BrN2O2S |
| S4502 | *Eltrombopag* | *442.47* | 496775-61-2 | Others | 26 | 58.76104595 | <1 |  | C25H22N4O4 |
| S2001 | Elvitegravir (GS-9137, JTK-303) | 447.88 | 697761-98-1 | Integrase | 90 | 200.9466821 | <1 |  | C23H23ClFNO5 |
| S7025 | Embelin | 294.39 | 550-24-3 | IAP | 59 | 200.4144163 | <1 |  | C17H26O4 |
| S2295 | Emodin | 270.24 | 518-82-1 | Others | 54 | 199.8223801 | <1 |  | C15H10O5 |
| S8022 | Empagliflozin (BI 10773) | 450.91 | 864070-44-0 | SGLT | 90 | 199.5963718 | <1 |  | C23H27ClO7 |
| S1704 | Emtricitabine | 247.25 | 143491-57-0 | Reverse Transcriptase | 49 | 198.1799798 | 49 | 198.1799798 | C8H10FN3O3S |
| S1941 | Enalapril Maleate | 492.52 | 76095-16-4 | Opioid Receptor | 99 | 201.0070657 | <1 |  | C24H32N2O9 |
| S1657 | Enalaprilat Dihydrate | 348.4 | 84680-54-6 | RAAS | 70 | 200.9184845 | <1 |  | C18H28N2O7 |
| S7839 | *Endoxifen HCl* | *409.95* | 1032008-74-4 | Estrogen/progestogen Receptor | 74 | 180.5098183 | <1 |  | C25H28ClNO2 |
| S1181 | ENMD-2076 | 375.47 | 934353-76-1 | Flt, Aurora Kinase, VEGFR | 105 | 279.649506 | 1 | 2.663328628 | C21H25N7 |
| S2018 | ENMD-2076 L-(+)-Tartaric acid | 525.56 | 1291074-87-7 | Aurora Kinase,FLT3,VEGFR | 100 | 190.27 | <1 |  | C25H31N7O6 |
| S1756 | Enoxacin | 320.32 | 74011-58-8 | Others | 64 | 199.8001998 | <1 |  | C15H17FN4O3 |
| S2296 | Enoxolone | 470.68 | 471-53-4 | Others | 94 | 199.7110563 | <1 |  | C30H46O4 |
| S3147 | Entacapone | 305.29 | 130929-57-6 | Others | 61 | 199.8100167 | <1 |  | C14H15N3O5 |
| S1252 | Entecavir Hydrate | 295.29 | 209216-23-9 | Others | 59 | 199.8035829 | <1 |  | C12H17N5O4 |
| S1053 | Entinostat (MS-275) | 376.41 | 209783-80-2 | HDAC | 75 | 199.2508169 | <1 |  | C21H20N4O3 |
| S1250 | Enzalutamide (MDV3100) | 464.44 | 915087-33-1 | Androgen Receptor, P450 | 92 | 198.08802 | <1 |  | C21H16F4N4O2S |
| S1055 | Enzastaurin (LY317615) | 515.61 | 170364-57-5 | PKC | 30 | 58.18351079 | <1 |  | C32H29N5O2 |
| S7910 | *Epacadostat (INCB024360)* | *438.23* | 1204669-58-8 | IDO | 87 | 198.5258882 | <1 |  | C11H13BrFN7O4S |
| S2035 | Epalrestat | 319.4 | 82159-09-9 | Others |  | 10 |  |  | C15H13NO3S2 |
| S2832 | Epiandrosterone | 290.44 | 481-29-8 | Estrogen/progestogen Receptor | 29 | 99.84850572 | <1 |  | C19H30O2 |
| S4253 | Epinastine HCl | 285.77 | 108929-04-0 | Others | 57 | 199.4611051 | 57 | 199.4611051 | C16H16ClN3 |
| S2521 | Epinephrine Bitartrate | 333.29 | 51-42-3 | Adrenergic Receptor | 67 | 201.0261334 | 67 | 201.0261334 | C13H19NO9 |
| S3061 | Epinephrine HCl | 219.67 | 55-31-2 | Adrenergic Receptor | 44 | 200.3004507 | 44 | 200.3004507 | C9H14ClNO3 |
| S1223 | Epirubicin HCl | 579.98 | 56390-09-1 | Topoisomerase | 100 | 172.4197386 | 100 | 172.4197386 | C27H30ClNO11 |
| S1297 | Epothilone A | 493.66 | 152044-53-6 | Microtubule Associated | 99 | 200.5428838 | <1 |  | C26H39NO6S |
| S1364 | Epothilone B (EPO906, Patupilone) | 507.68 | 152044-54-7 | Microtubule Associated | 102 | 200.9139616 | <1 |  | C27H41NO6S |
| S4102 | Eprosartan Mesylate | 520.62 | 144143-96-4 | Others | 104 | 199.7618224 | <1 |  | C24H28N2O7S2 |
| S7353 | EPZ004777 | 539.67 | 1338466-77-5 | Histone Methyltransferase | 100 | 185.2984231 | <1 |  | C28H41N7O4 |
| S7748 | *EPZ015666* | *383.44* | 1616391-65-1 | Histone Methyltransferase | 60 | 156.4781974 | <1 |  | C20H25N5O3 |
| S7062 | EPZ5676 | 562.71 | 1380288-87-8 | Methyltransferase | 100 | 177.7114322 | <1 |  | C30H42N8O3 |
| S7128 | EPZ-6438 | 572.74 | 1403254-99-8 | Histone Methyltransferase |  | 10 |  |  | C34H44N4O4 |
| S2450 | Equol | 242.27 | 531-95-3 | Estrogen/progestogen Receptor | 48 | 198.1260577 | <1 |  | C15H14O3 |
| S7242 | Erastin | 547.04 | 571203-78-6 | Ferroptosis | 19 | 34.73237789 | <1 |  | C30H31ClN4O4 |
| S1825 | Erdosteine | 249.31 | 84611-23-4 | Others | 50 | 200.5535277 | <1 |  | C8H11NO4S2 |
| S7334 | *ERK5-IN-1* | *638.81* | 1435488-37-1 | ERK | 100 | 156.5410685 | <1 |  | C36H46N8O3 |
| S7786 | *Erlotinib* | *393.44* | 183321-74-6 | EGFR | 78 | 198.2513217 | <1 |  | C22H23N3O4 |
| S1023 | Erlotinib HCl (OSI-744) | 429.9 | 183319-69-9 | EGFR |  | 10 |  |  | C22H24ClN3O4 |
| S4224 | Erythritol | 122.12 | 149-32-6 | Others | 24 | 196.5280052 | 24 | 196.5280052 | C4H10O4 |
| s1635 | Erythromycin | 733.93 | 114-07-8 | Others | 147 | 200.2915809 | 3 | 4.087583285 | C37H67NO13 |
| S7550 | *Erythromycin Cyclocarbonate* | *759.92* | 55224-05-0 | Others | 100 | 131.5927992 | <1 |  | C38H65NO14 |
| S4060 | *Erythromycin Ethylsuccinate* | *862.05* | 1264-62-6 | Others | 172 | 199.5243895 | <1 |  | C43H75NO16 |
| S4064 | Escitalopram Oxalate | 414.43 | 219861-08-2 | 5-HT Receptor | 83 | 200.2750766 | 25 | 60.32381826 | C22H23FN2O5 |
| S2258 | Esculin | 340.28 | 531-75-9 | Others | 68 | 199.8354296 | 2 | 5.877512637 | C15H16O9 |
| S7499 | ESI-09 | 330.77 | 263707-16-0 | Others | 66 | 199.5344197 | <1 |  | C16H15ClN4O2 |
| S4100 | Esmolol HCl | 331.83 | 81161-17-3, 81147-92-4(free base) | Others | 66 | 198.8970256 | 66 | 198.8970256 | C16H26ClNO4 |
| S1743 | *Esomeprazole Magnesium* | *713.12* | 161973-10-0 | 5-alpha Reductase | 143 | 200.5272605 | <1 |  | C34H36MgN6O6S2 |
| S2233 | Esomeprazole Sodium | 367.4 | 161796-78-7 | ATPase | 73 | 198.693522 | 73 | 198.693522 | C17H18N3NaO3S |
| S1709 | Estradiol | 272.38 | 50-28-2 | Others | 54 | 198.2524414 | <1 |  | C18H24O2 |
| S4110 | Estradiol Benzoate | 376.49 | 50-50-0 | Others | 75 | 199.2084783 | <1 |  | C25H28O3 |
| S4046 | Estradiol Cypionate | 396.56 | 313-06-4 | Estrogen/progestogen Receptor | 79 | 199.2132338 | <1 |  | C26H36O3 |
| S3149 | Estradiol valerate | 356.5 | 979-32-8 | Estrogen/progestogen Receptor | 71 | 199.1584853 | <1 |  | C23H32O3 |
| S2466 | Estriol | 288.39 | 50-27-1 | Estrogen/progestogen Receptor | 57 | 197.649017 | <1 |  | C18H24O3 |
| S1665 | Estrone | 270.37 | 53-16-7 | Others | 54 | 199.726301 | <1 |  | C18H22O2 |
| S4196 | Ethacridine lactate monohydrate | 361.39 | 6402-23-9 | Others | 72 | 199.2307479 | 5 | 13.83546861 | C18H23N3O5 |
| S4004 | Ethambutol HCl | 277.23 | 1070-11-7 | Others | 56 | 201.9983407 | 56 | 201.9983407 | C10H26Cl2N2O2 |
| S4152 | Ethamsylate | 263.31 | 2624-44-4 | Others | 53 | 201.283658 | 53 | 201.283658 | C10H17NO5S |
| S1625 | Ethinyl Estradiol | 296.4 | 57-63-6 | Others | 59 | 199.0553306 | <1 |  | C20H24O2 |
| S1777 | Ethionamide | 166.24 | 536-33-4 | Others | 33 | 198.5081809 | <1 |  | C8H10N2S |
| S3160 | Ethynodiol diacetate | 384.51 | 297-76-7 | Estrogen/progestogen Receptor | 77 | 200.2548698 | 77 | 200.2548698 | C24H32O4 |
| S4276 | Etizolam | 342.85 | 40054-69-1 | Others | 26 | 75.83491323 | <1 |  | C17H15ClN4S |
| S1328 | Etodolac | 287.35 | 41340-25-4 | COX | 58 | 201.8444406 | <1 |  | C17H21NO3 |
| S4264 | Etofibrate | 363.79 | 31637-97-5 | Others | 73 | 200.6652189 | <1 |  | C18H18ClNO5 |
| S1329 | Etomidate | 244.29 | 33125-97-2 | GABA Receptor | 49 | 200.5812764 | <1 |  | C14H16N2O2 |
| S1225 | Etoposide | 588.56 | 33419-42-0 | Topoisomerase | 100 | 169.9062118 | <1 |  | C29H32O13 |
| S8050 | ETP-46464 | 470.52 | 1345675-02-6 | mTOR | 6 | 12.75184902 | <1 |  | C30H22N4O2 |
| S3080 | Etravirine (TMC125) | 435.28 | 269055-15-4 | Reverse Transcriptase | 42 | 96.48961588 | <1 |  | C20H15BrN6O |
| S4261 | EUK 134 | 416.74 | 81065-76-1 | Beta Amyloid | 83 | 199.164947 | 13 | 31.19450977 | C18H18ClMnN2O4 |
| S2925 | Evacetrapib (LY2484595) | 638.65 | 1186486-62-3 | CETP | 12.8 | 20.04227668 | <1 |  | C31H36F6N6O2 |
| S1120 | Everolimus (RAD001) | 958.22 | 159351-69-6 | mTOR | 30 | 31.30805034 | <1 |  | C53H83NO14 |
| S7530 | *EW-7197* | *399.42* | 1352608-82-2 | TGF-beta/Smad | 79 | 197.7867908 | <1 |  | C22H18FN7 |
| S1541 | EX 527 (Selisistat) | 248.71 | 49843-98-3 | Sirtuin | 50 | 201.0373527 | <1 |  | C13H13ClN2O |
| S1196 | Exemestane | 296.4 | 107868-30-4 | Aromatase | 54 | 182.1862348 | <1 |  | C20H24O2 |
| S1655 | Ezetimibe | 409.4 | 163222-33-1 | Others | 82 | 200.2931119 | <1 |  | C24H21F2NO3 |
| S2467 | Famciclovir | 321.33863 | 104227-87-4 | Others | 64 | 199.1668415 | 64 | 199.1668415 | C14H19N5O4 |
| S2078 | Famotidine | 337.45 | 76824-35-6 | Histamine Receptor | 67 | 198.547933 | <1 |  | C8H15N7O2S3 |
| S1573 | Fasudil (HA-1077) HCl | 327.83 | 105628-07-7 | ROCK | 5 | 15.25180734 | 65 | 198.2734954 | C14H18ClN3O2S |
| S1547 | Febuxostat | 316.37 | 144060-53-7 | Others | 63 | 199.1339255 | <1 |  | C16H16N2O3S |
| S1330 | Felbamate | 238.24 | 25451-15-4 | Others | 48 | 201.4775017 | <1 |  | C11H14N2O4 |
| S1885 | Felodipine | 384.25 | 72509-76-3 | NULL | 77 | 200.3903709 | <1 |  | C18H19Cl2NO4 |
| S2468 | Fenbendazole | 299.35 | 43210-67-9 | Others | 4 | 13.36228495 | <1 |  | C15H13N3O2S |
| S1794 | Fenofibrate | 360.83 | 49562-28-9 | NULL | 72 | 199.5399496 | <1 |  | C20H21ClO4 |
| S1823 | Fenoprofen Calcium | 522.6 | 34597-40-5 | Others | 105 | 200.9184845 | <1 |  | C30H26CaO6 |
| S3027 | *Fenoprofen calcium hydrate* | *558.63* | 71720-56-4 | Others | 48 | 85.92449385 | <1 |  | C30H30CaO8 |
| S4090 | Fenspiride HCl | 296.79 | 5053-08-7, 5053-06-5(free base) | Others | 9 | 30.32447185 | 59 | 198.7937599 | C15H21ClN2O2 |
| S2031 | Fenticonazole Nitrate | 518.41 | 73151-29-8 | Others | 104 | 200.6134141 | <1 |  | C24H21Cl2N3O4S |
| S7243 | Ferrostatin-1 (Fer-1) | 262.35 | 347174-05-4 | Ferroptosis | 52 | 198.2085001 | <1 |  | C15H22N2O2 |
| S2300 | Ferulic Acid | 194.19 | 1135-24-6 | Others | 39 | 200.8342345 | <1 |  | C10H10O4 |
| S2240 | Fesoterodine Fumarate | 527.65 | 286930-03-8 | AChR | 100 | 189.5195679 | 100 | 189.5195679 | C30H41NO7 |
| S3208 | Fexofenadine HCl | 538.12 | 153439-40-8 | Histamine Receptor | 107 | 198.8404073 | 2 | 3.716643128 | C32H40ClNO4 |
| S1007 | FG-4592 | 352.34 | 808118-40-3 | HIF | 70 | 198.6717375 | <1 |  | C19H16N2O5 |
| S7450 | FH1(BRD-K4477) | 282.34 | 2719-05-3 | Others | 56 | 198.342424 | <1 |  | C17H18N2O2 |
| S7484 | FH535 | 361.2 | 108409-83-2 | Wnt/beta-catenin | 72 | 199.3355482 | <1 |  | C13H10Cl2N2O4S |
| S4227 | Fidaxomicin | 1058.04 | 873857-62-6 | Others | 100 | 94.51438509 | <1 |  | C52H74Cl2O18 |
| S7605 | Filgotinib (GLPG0634) | 425.5 | 1206161-97-8 | JAK | 85 | 199.7649824 | <1 |  | C21H23N5O3S |
| S1197 | Finasteride | 372.54 | 98319-26-7 | 5-alpha Reductase | 75 | 201.3206636 | <1 |  | C23H36N2O2 |
| S5002 | Fingolimod (FTY720) HCl | 343.9 | 162359-56-0 | S1P Receptor, Bcr-Abl, PKC | 69 | 200.6397208 | 69 | 200.6397208 | C19H34ClNO2 |
| S2298 | Fisetin | 286.24 | 528-48-3 | Others | 57 | 199.1335942 | <1 |  | C15H10O6 |
| S1230 | *Flavopiridol (Alvocidib)* | *401.84* | 146426-40-6 | CDK | 15 | 37.32828987 | <1 |  | C21H20ClNO5 |
| S2679 | Flavopiridol HCl | 438.3 | 131740-09-5 | CDK | 88 | 200.7757244 | <1 |  | C21H21Cl2NO5 |
| S4027 | Flavoxate HCl | 427.92 | 3717-88-2 | AChR | 3 | 7.010656197 | 10 | 23.36885399 | C24H26ClNO4 |
| S7399 | FLI-06 | 438.52 | 313967-18-9 | Gamma-secretase | 88 | 200.6749977 | <1 |  | C25H30N2O5 |
| S7259 | *FLLL32* | *464.55* | 1226895-15-3 | JAK | 92 | 198.0411151 | <1 |  | C28H32O6 |
| S4249 | Flopropione | 182.17 | 2295-58-1 | Others | 36 | 197.6176099 | <1 |  | C9H10O4 |
| S4201 | Florfenicol | 358.21 | 73231-34-2 | Others | 72 | 200.9994138 | <1 |  | C12H14Cl2FNO4S |
| S1299 | Floxuridine | 246.19 | 50-91-9 | DNA/RNA Synthesis | 49 | 199.033267 | 49 | 199.033267 | C9H11FN2O5 |
| S1331 | Fluconazole | 306.27 | 86386-73-4 | Others | 61 | 199.1706664 | <1 |  | C13H12F2N6O |
| S1666 | Flucytosine | 129.09 | 2022-85-7 | Others | 8 | 61.97226741 | 5 | 38.73266713 | C4H4FN3O |
| S1491 | *Fludarabine* | *285.23* | 21679-14-1 | STAT, DNA/RNA Synthesis | 57 | 199.8387266 | <1 |  | C10H12FN5O4 |
| S1229 | Fludarabine Phosphate | 365.21 | 75607-67-9 | DNA/RNA Synthesis | 73 | 199.8849977 | 2 | 5.476301306 | C10H13FN5O7P |
| S4268 | Flufenamic acid | 281.23 | 530-78-9 | Others | 56 | 199.1252711 | <1 |  | C14H10F3NO2 |
| S1332 | Flumazenil | 303.29 | 78755-81-4 | GABA Receptor | 5 | 16.48587161 | <1 |  | C15H14FN3O3 |
| S3181 | Flumequine | 261.25 | 42835-25-6 | Others | 3 | 11.48325359 | <1 |  | C14H12FNO3 |
| S4088 | Flumethasone | 410.45 | 2135-17-3 | Others | 82 | 199.7807285 | <1 |  | C22H28F2O5 |
| S2030 | Flunarizine 2HCl | 477.42 | 30484-77-6 | Calcium Channel | 5 | 10.47295882 | <1 |  | C26H28Cl2F2N2 |
| S2108 | Flunixin Meglumin | 491.46 | 42461-84-7 | COX | 98 | 199.405852 | 98 | 199.405852 | C21H28F3N3O7 |
| S2470 | Fluocinolone Acetonide | 452.4999 | 67-73-2 | Others | 90 | 198.8950716 | <1 |  | C24H30F2O6 |
| S2608 | Fluocinonide | 494.52 | 356-12-7 | Others | 99 | 200.1941276 | <1 |  | C26H32F2O7 |
| S4228 | Fluorometholone Acetate | 418.5 | 3801-06-7 | Others | 84 | 200.7168459 | <1 |  | C24H31FO5 |
| S1209 | Fluorouracil (5-Fluoracil, 5-FU) | 130.08 | 51-21-8 | DNA/RNA Synthesis | 26 | 199.8769988 | <1 |  | C4H3FN2O2 |
| S1333 | Fluoxetine HCl | 345.79 | 56296-78-7 | 5-HT Receptor | 69 | 199.5430753 | 4 | 11.56771451 | C17H19ClF3NO |
| S1334 | Flupirtine maleate | 420.39 | 75507-68-5 | Antimetabolites | 84 | 199.814458 | <1 |  | C19H21FN4O6 |
| S1679 | Flurbiprofen | 244.26 | 51543-39-6 | Others | 49 | 200.6059117 | <1 |  | C15H13FO2 |
| S1908 | Flutamide | 276.21 | 13311-84-7 | P450 | 55 | 199.123855 | <1 |  | C11H11F3N2O3 |
| S1992 | Fluticasone propionate | 500.57 | 80474-14-2 | Others | 100 | 199.7722596 | <1 |  | C25H31F3O5S |
| S1909 | Fluvastatin Sodium | 433.45 | 93957-55-2 | HMG-CoA Reductase | 87 | 200.7151921 | 1 | 2.307071173 | C24H25FNNaO4 |
| S1336 | Fluvoxamine maleate | 434.41 | 61718-82-9 | 5-HT Receptor | 87 | 200.2716328 | <1 |  | C19H25F3N2O6 |
| S1111 | Foretinib (GSK1363089) | 632.65 | 849217-64-7 | c-Met, VEGFR | 127 | 200.7429068 | <1 |  | C34H34F2N4O6 |
| S2208 | Formestane | 302.41 | 566-48-3 | Aromatase | 61 | 201.7129063 | <1 |  | C19H26O3 |
| S2299 | Formononetin | 268.26 | 485-72-3 | Others | 54 | 201.2972489 | <1 |  | C16H12O4 |
| S2020 | Formoterol Hemifumarate | 402.4 | 43229-80-7 | Adrenergic Receptor | 80 | 198.8071571 | <1 |  | C42H52N4O12 |
| S2449 | Forskolin | 410.5 | 66575-29-9 | cAMP | 82 | 199.7563946 | <1 |  | C22H34O7 |
| S3038 | Fosaprepitant dimeglumine salt | 1004.83 | 265121-04-8 | Others | 201 | 200.0338366 | 135 | 134.3510843 | C37H56F7N6O16P |
| S3076 | Foscarnet Sodium | 191.95 | 63585-09-1 | Others | 7 | 36.46783016 | 3 | 15.62907007 | CNa3O5P |
| S2625 | Fostamatinib (R788) | 580.46 | 901119-35-5 | Syk | 116 | 199.841505 | <1 |  | C23H26FN6O9P |
| S7451 | FPH1 (BRD-6125) | 388.82 | 708219-39-0 | Others | 78 | 200.6069647 | <1 |  | C16H15ClF2N2O3S |
| S7452 | FPH2 (BRD-9424) | 353.83 | 957485-64-2 | Others | 70 | 197.8351186 | <1 |  | C14H16ClN5O2S |
| S7524 | *FR 180204* | *327.34* | 865362-74-9 | ERK | 65 | 198.5702939 | <1 |  | C18H13N7 |
| S7271 | *FRAX597* | *558.10* | 1286739-19-2 | PAK | 14 | 25.0851102 | <1 |  | C29H28ClN7OS |
| S1300 | FT-207 (NSC 148958) | 200.17 | 17902-23-7 | DNA/RNA Synthesis | 40 | 199.8301444 | 8 | 39.96602888 | C8H9FN2O3 |
| S7465 | *FTI 277 HCl* | *484.07* | 180977-34-8 | Transferase | 96 | 198.318425 | 17 | 35.11888776 | C22H30ClN3O3S2 |
| S1191 | Fulvestrant | 606.77 | 129453-61-8 | Estrogen/progestogen Receptor | 100 | 164.8070933 | <1 |  | C32H47F5O3S |
| S4203 | Furaltadone HCl | 360.75 | 3759-92-0 | Others | 17 | 47.12404712 | 72 | 199.5841996 | C13H17ClN4O6 |
| S1603 | Furosemide | 330.74 | 54-31-9 | Others | 66 | 199.5525186 | <1 |  | C12H11ClN2O5S |
| S7239 | *G007-LK* | *529.96* | 1380672-07-0 | PARP | 100 | 188.6934863 | <1 |  | C25H16ClN7O3S |
| S7545 | *G-749* | *521.41* | 1457983-28-6 | FLT3 | 24 | 46.02903665 | <1 |  | C25H25BrN6O2 |
| S2101 | Gabexate Mesylate | 417.48 | 56974-61-9 | Proteasome | 83 | 198.8119191 | 12 | 28.74389192 | C17H27N3O7S |
| S2803 | Galeterone | 388.55 | 851983-85-2 | Androgen Receptor, P450 | 24 | 61.76811221 | <1 |  | C26H32N2O |
| S2471 | Gallamine Triethiodide | 891.53 | 65-29-2 | AChR | 100 | 112.1667246 | 100 | 112.1667246 | C30H60I3N3O3 |
| S2448 | *Gambogic Acid* | *628.75* | 2752-65-0 | Others | 100 | 159.0457256 | <1 |  | C38H44O8 |
| S1878 | Ganciclovir | 255.23 | 82410-32-0 | Others | 50 | 195.9017357 | <1 |  | C9H13N5O4 |
| S1159 | Ganetespib (STA-9090) | 364.4 | 888216-25-9 | HSP | 40 | 109.7694841 | <1 |  | C20H20N4O3 |
| S2383 | Gastrodin | 286.28 | 62499-27-8 | Others | 50 | 174.6541847 | <1 |  | C13H18O7 |
| S7103 | *GDC-0032* | *460.53* | 1282512-48-4 | PI3K | 70 | 151.998784 | <1 |  | C24H28N8O2 |
| S2808 | GDC-0068 | 458 | 1001264-89-6 | Akt | 92 | 200.8733624 | <1 |  | C24H32ClN5O2 |
| S7010 | GDC-0152 | 498.64 | 873652-48-3 | IAP | 99 | 198.5400289 | 3 | 6.016364511 | C25H34N6O3S |
| S8040 | GDC-0349 | 452.55 | 1207360-89-1 | mTOR | 91 | 201.0827533 | <1 |  | C24H32N6O3 |
| S7553 | *GDC-0623* | *456.21* | 1168091-68-6 | MEK | 91 | 199.4695425 | <1 |  | C16H14FIN4O3 |
| S1104 | GDC-0879 | 334.37 | 905281-76-7 | Raf | 66 | 197.3861291 | <1 |  | C19H18N4O2 |
| S1065 | GDC-0941 | 513.64 | 957054-30-7 | PI3K | 44 | 85.66311035 | <1 |  | C23H27N7O3S2 |
| S2696 | GDC-0980 (RG7422) | 498.6 | 1032754-93-0 | mTOR, PI3K | 20 | 40.11231448 | <1 |  | C23H30N8O3S |
| S1025 | Gefitinib (ZD1839) | 446.9 | 184475-35-2 | EGFR | 89 | 199.1496979 | <1 |  | C22H24ClFN4O3 |
| S2713 | Geldanamycin | 560.64 | 30562-34-6 | HSP | 50 | 89.18378995 | <1 |  | C29H40N2O9 |
| S1714 | Gemcitabine | 263.2 | 95058-81-4 | Others | 15 | 56.99088146 | 16 | 60.79027356 | C9H11F2N3O4 |
| S1729 | Gemfibrozil | 250.33 | 25812-30-0 | Others | 50 | 199.736348 | <1 |  | C15H22O3 |
| S2412 | Genipin | 226.23 | 6902-77-8 | Others | 45 | 198.9126111 | <1 |  | C11H14O5 |
| S2411 | Geniposide | 388.37 | 24512-63-8 | Others | 78 | 200.8394057 | 78 | 200.8394057 | C17H24O10 |
| S2413 | Geniposidic acid | 374.34 | 27741-01-1 | Others | 16 | 42.7418924 | <1 |  | C16H22O10 |
| S1342 | Genistein | 270.24 | 446-72-0 | Topoisomerase | 54 | 199.8223801 | <1 |  | C15H10O5 |
| S1376 | Gestodene | 310.43 | 60282-87-3 | Estrogen/progestogen Receptor | 62 | 199.7229649 | <1 |  | C21H26O2 |
| S7208 | GF109203X | 412.48 | 133052-90-1 | PKC | 82 | 198.7975175 | <1 |  | C25H24N4O2 |
| S2055 | Gimeracil | 145.54 | 103766-25-2 | Dehydrogenase | 29 | 199.257936 | <1 |  | C5H4ClNO2 |
| S2026 | Ginkgolide A | 408.4 | 15291-75-5 | GABA Receptor | 81 | 198.3349657 | <1 |  | C20H24O9 |
| S1343 | Ginkgolide B | 424.4 | 15291-77-7 | PAFR | 85 | 200.2827521 | <1 |  | C20H24O10 |
| S2170 | Givinostat (ITF2357) | 475.97 | 732302-99-7 | HDAC | 95 | 199.5924113 | <1 |  | C24H30ClN3O5 |
| S7171 | *GKT137831* | *394.85* | 1218942-37-0 | Others | 78 | 197.5433709 | <1 |  | C21H19ClN4O2 |
| S2601 | Gliclazide | 323.41 | 21187-98-4 | Others | 65 | 200.983272 | <1 |  | C15H21N3O3S |
| S1344 | Glimepiride | 490.62 | 93479-97-1 | DPP-4 | 11 | 22.42061066 | <1 |  | C24H34N4O5S |
| S1715 | Glipizide | 445.54 | 29094-61-9 | Others | 89 | 199.7575975 | <1 |  | C21H27N5O4S |
| S3151 | Gliquidone | 527.63 | 33342-05-1 | Others | 105 | 199.0030893 | <1 |  | C27H33N3O6S |
| S1716 | Glyburide | 494 | 10238-21-8 | Others | 99 | 200.4048583 | <1 |  | C23H28ClN3O5S |
| S2302 | Glycyrrhizin (Glycyrrhizic Acid) | 822.93 | 1405-86-3 | Dehydrogenase | 117 | 142.17 | 20 | 24.30340369 | C42H62O16 |
| S7367 | GNE-0877 | 339.32 | 1374828-69-9 | Others | 67 | 197.453731 | <1 |  | C14H16F3N7 |
| S7798 | *GNE-317* | *414.48* | 1394076-92-6 | PI3K | 47 | 113.3950975 | <1 |  | C19H22N6O3S |
| S7528 | GNE-7915 | 443.4 | 1351761-44-8 | Others | 22 | 49.61659901 | <1 |  | C19H21F4N5O3 |
| S7368 | GNE-9605 | 449.83 | 1536200-31-3 | LRRK2 | 89 | 197.8525221 | <1 |  | C17H20ClF4N7O |
| S2899 | GNF-2 | 374.32 | 778270-11-4 | Bcr-Abl | 74 | 197.6918145 | <1 |  | C18H13F3N4O2 |
| S7526 | GNF-5 | 418.37 | 778277-15-9 | Bcr-Abl | 83 | 198.3889858 | <1 |  | C20H17F3N4O3 |
| S7519 | *GNF-5837* | *535.49* | 1033769-28-6 | Trk receptor | 100 | 186.7448505 | <1 |  | C28H21F4N5O2 |
| S2911 | Go 6983 | 442.51 | 133053-19-7 | PKC | 59 | 133.3303202 | <1 |  | C26H26N4O3 |
| S7119 | *Go6976* | *377.42* | 136194-77-9 | PKC | 18 | 47.69222617 | <1 |  | C24H18N4O |
| S7266 | Golgicide A | 284.3 | 1139889-93-2 | ATPase | 57 | 200.4924376 | <1 |  | C17H14F2N2 |
| S2859 | Golvatinib (E7050) | 633.69 | 928037-13-2 | c-Met, VEGFR | 20 | 31.56117344 | <1 |  | C33H37F2N7O4 |
| S2303 | Gossypol | 518.56 | 12542-36-8 | Dehydrogenase | 100 | 192.8417155 | <1 |  | C32H34O10 |
| S2304 | Gramine | 174.24 | 87-52-5 | Others | 35 | 200.87236 | <1 |  | C11H14N2 |
| S4071 | Griseofulvin | 352.77 | 126-07-8 | Microtubule Associated | 50 | 141.7354083 | <1 |  | C17H17ClO6 |
| S7523 | GS-9973 | 411.46 | 1229208-44-9 | Syk | 82 | 199.290332 | <1 |  | C23H21N7O |
| S7581 | *GSK J1* | *389.45* | 1373422-53-7 | Histone Demethylase | 77 | 197.7147259 | <1 |  | C22H23N5O2 |
| S7070 | GSK J4 HCl | 453.96 | 1373423-53-0(free base) | Others | 90 | 198.2553529 | <1 |  | C24H28ClN5O2 |
| S2740 | GSK1070916 | 507.63 | 942918-07-2 | Aurora Kinase | 102 | 200.93 | <1 |  | C30H33N7O |
| S2149 | GSK1292263 | 456.56 | 1032823-75-8 | GPR | 34 | 74.46994919 | <1 |  | C23H28N4O4S |
| S7620 | *GSK1324726A (I-BET726)* | *434.91* | 1300031-52-0 | Epigenetic Reader Domain | 86 | 197.7420616 | <1 |  | C25H23ClN2O3 |
| S2703 | GSK1838705A | 532.57 | 1116235-97-2 | IGF-1, ALK | 107 | 200.9125561 | <1 |  | C27H29FN8O3 |
| S1093 | GSK1904529A | 851.96 | 1089283-49-7 | IGF-1R | 124 | 145.5467393 | <1 |  | C44H47F2N9O5S |
| S2658 | GSK2126458 (GSK458) | 505.5 | 1086062-66-9 | PI3K, mTOR | 100 | 197.8239367 | <1 |  | C25H17F2N5O3S |
| S7087 | GSK2334470 | 462.59 | 1227911-45-6 | PDK-1 | 90 | 194.5567349 | <1 |  | C25H34N8O |
| S7664 | *GSK2578215A* | *399.42* | 1285515-21-0 | LRRK2 | 79 | 197.7867908 | <1 |  | C24H18FN3O2 |
| S7307 | GSK2606414 | 451.44 | 1337531-36-8 | PERK | 90 | 199.3620415 | <1 |  | C24H20F3N5O |
| S8002 | GSK2636771 | 433.42 | 1372540-25-4 | PI3K | 28 | 64.60246412 | <1 |  | C22H22F3N3O3 |
| S7033 | GSK2656157 | 416.45 | 1337532-29-2 | PERK | 32 | 76.83995678 | <1 |  | C23H21FN6O |
| S7573 | *GSK2830371* | *461.02* | 1404456-53-6 | Angiogenesis | 92 | 199.5575029 | <1 |  | C23H29ClN4O2S |
| S8025 | GSK3787 | 392.78 | 188591-46-0 | PPAR | 79 | 201.1304038 | <1 |  | C15H12ClF3N2O3S |
| S1474 | GSK429286A | 432.37 | 864082-47-3 | ROCK | 87 | 201.2165506 | <1 |  | C21H16F4N4O2 |
| S2193 | GSK461364 | 543.6 | 929095-18-1 | PLK | 10 | 18.39587932 | <1 |  | C27H28F3N5O2S |
| S7804 | *GSK503* | *526.67* | 1346572-63-1 | Histone Methyltransferase | 100 | 189.872216 | <1 |  | C31H38N6O2 |
| S7209 | GSK650394 | 382.45 | 890842-28-1 | Others | 76 | 198.7187868 | <1 |  | C25H22N2O2 |
| S1113 | GSK690693 | 425.48 | 937174-76-0 | Akt | 39 | 91.66118266 | <1 |  | C21H27N7O3 |
| S7090 | GSK923295 | 592.13 | 1088965-37-0 | Kinesin | 100 | 168.8818334 | <1 |  | C32H38ClN5O4 |
| S7574 | *GSK-LSD1 2HCl* | *289.24* | 1431368-48-7 | Histone Demethylase | 57 | 197.0681787 | 57 | 197.0681787 | C14H22Cl2N2 |
| S1740 | Guaifenesin | 198.22 | 93-14-1 | Others | 40 | 201.7959843 | 25 | 126.1224902 | C10H14O4 |
| S4065 | Guanabenz Acetate | 291.13 | 23256-50-0 | Adrenergic Receptor | 58 | 199.2237145 | <1 |  | C10H12Cl2N4O2 |
| S4070 | Guanidine HCl | 95.53 | 50-01-1 | Others | 19 | 198.8904009 | 19 | 198.8904009 | CH6ClN3 |
| S2439 | Guanosine | 283.24 | 118-00-3 | Others | 57 | 201.2427623 | <1 |  | C10H13N5O5 |
| S8020 | GW0742 | 471.49 | 317318-84-6 | PPAR | 94 | 199.3679611 | <1 |  | C21H17F4NO3S2 |
| S8042 | GW2580 | 366.41 | 870483-87-7 | CSF-1R | 48 | 131.0007915 | <1 |  | C20H22N4O3 |
| S2630 | GW3965 HCl | 618.51 | 405911-17-3 | Liver X Receptor | 16 | 25.86861975 | <1 |  | C33H32Cl2F3NO3 |
| S2782 | GW4064 | 542.84 | 278779-30-9 | FXR | 100 | 184.2163437 | <1 |  | C28H22Cl3NO4 |
| S2891 | *GW441756* | *275.3* | 504433-23-2 | Others | 25 | 90.81002543 | <1 |  | C17H13N3O |
| S2872 | GW5074 | 520.94 | 220904-83-6 | Raf | 104 | 199.6391139 | <1 |  | C15H8Br2INO2 |
| S2750 | GW788388 | 425.48 | 452342-67-5 | TGF-beta/Smad | 15 | 35.25430102 | <1 |  | C25H23N5O2 |
| S2778 | GW842166X | 449.25 | 666260-75-9 | Cannabinoid Receptor | 20 | 44.51864218 | <1 |  | C18H17Cl2F3N4O2 |
| S8014 | GW9508 | 347.41 | 885101-89-3 | GPR | 69 | 198.6125903 | <1 |  | C22H21NO3 |
| S2915 | GW9662 | 276.68 | 22978-25-2 | PPAR | 55 | 198.7856007 | <1 |  | C13H9ClN2O3 |
| S2306 | Gynostemma Extract | 917.13 | 80321-63-7 | Others | 189 | 206.0776553 | <1 |  | C47H80O17 |
| S7194 | GZD824 | 724.77 | 1421783-64-3 | Bcr-Abl | 100 | 137.9748058 | 100 | 137.9748058 | C31H35F3N6O7S2 |
| S1582 | H 89 2HCl | 519.28 | 130964-39-5 | S6 Kinase | 104 | 200.277307 | 6 | 11.55446002 | C20H22BrCl2N3O2S |
| S1071 | HA14-1 | 409.23 | 65673-63-4 | Bcl-2 | 82 | 200.3763165 | <1 |  | C17H17BrN2O5 |
| S4098 | Halcinonide | 454.96 | 3093-35-4 | Others | 90 | 197.8195885 | <1 |  | C24H32ClFO5 |
| S4089 | Halobetasol Propionate | 484.96 | 66852-54-8 | Others | 97 | 200.0164962 | <1 |  | C25H31ClF2O5 |
| S1920 | Haloperidol | 375.86 | 52-86-8 | Others | 75 | 199.5423828 | <1 |  | C21H23ClFNO2 |
| S2918 | HC-030031 | 355.39 | 349085-38-7 | Others | 32 | 90.04192577 | <1 |  | C18H21N5O3 |
| S2384 | Hematoxylin | 302.28 | 517-28-2 | Others | 61 | 201.7996559 | 61 | 201.7996559 | C16H14O6 |
| S1529 | Hesperadin | 516.65 | 422513-13-1 | Aurora Kinase | 103 | 199.3612697 | <1 |  | C29H32N4O3S |
| S2308 | Hesperetin | 302.27 | 520-33-2 | Histamine Receptor | 60 | 198.4980316 | <1 |  | C16H14O6 |
| S2309 | Hesperidin | 610.56 | 520-26-3 | NULL | 122 | 199.8165618 | <1 |  | C28H34O15 |
| S2473 | Hexestrol | 270.37 | 84-16-2 | Estrogen/progestogen Receptor | 42 | 155.3426786 | <1 |  | C18H22O2 |
| S4118 | Histamine 2HCl | 184.07 | 56-92-8 | Histamine Receptor | 3 | 16.29814744 | 37 | 201.0104851 | C5H11Cl2N3 |
| S7500 | HJC0350 | 277.38 | 885434-70-8 | Others | 52 | 187.4684548 | <1 |  | C15H19NO2S |
| S1485 | HMN-214 | 424.47 | 173529-46-9 | PLK | 12 | 28.27054916 | <1 |  | C22H20N2O5S |
| S7501 | HO-3867 | 464.55 | 1172133-28-6 | STAT | 13 | 27.98407061 | <1 |  | C28H30F2N2O2 |
| S4025 | Homatropine Bromide | 356.25 | 51-56-9 | AChR | 71 | 199.2982456 | 71 | 199.2982456 | C16H22BrNO3 |
| S4024 | Homatropine Methylbromide | 370.28 | 80-49-9 | AChR | 74 | 199.8487631 | 74 | 199.8487631 | C17H24BrNO3 |
| S2310 | Honokiol | 266.334 | 35354-74-6 | Akt, MEK | 53 | 198.9982503 | <1 |  | C18H18O2 |
| S2385 | Hordenine | 165.23 | 539-15-1 | Others | 33 | 199.7216002 | <1 |  | C10H15NO |
| S7278 | *HPOB* | *314.34* | 1429651-50-2 | HDAC | 62 | 197.2386588 | <1 |  | C17H18N2O4 |
| S7356 | HS-173 | 422.46 | 1276110-06-5 | PI3K | 84 | 198.8353927 | <1 |  | C21H18N4O4S |
| S7097 | HSP990 (NVP-HSP990) | 379.39 | 934343-74-5 | HSP (e.g. HSP90) | 75 | 197.6857587 | <1 |  | C20H18FN5O2 |
| S7318 | *HTH-01-015* | *468.55* | 1613724-42-7 | AMPK | 58 | 123.7861488 | <1 |  | C26H28N8O |
| S1708 | Hydrochlorothiazide | 297.74 | 58-93-5 | Others | 60 | 201.518103 | <1 |  | C7H8ClN3O4S2 |
| S1696 | Hydrocortisone | 362.46 | 50-23-7 | Others | 73 | 201.401534 | <1 |  | C21H30O5 |
| S1896 | Hydroxyurea | 76.05 | 127-07-1 | Others | 15 | 197.2386588 | 15 | 197.2386588 | CH4N2O2 |
| S4026 | Hydroxyzine 2HCl | 447.83 | 2192-20-3 | Histamine Receptor | 90 | 200.9691177 | 90 | 200.9691177 | C21H29Cl3N2O2 |
| S2311 | Hyodeoxycholic acid (HDCA) | 392.57 | 83-49-8 | Others | 78 | 198.6906794 | <1 |  | C24H40O4 |
| S4014 | Hyoscyamine | 289.37 | 101-31-5 | AChR | 58 | 200.4354287 | <1 |  | C17H23NO3 |
| S7189 | I-BET-762 | 423.9 | 1260907-17-2 | Epigenetic Reader Domain | 84 | 198.1599434 | <1 |  | C22H22ClN5O2 |
| S2680 | Ibrutinib (PCI-32765) | 440.5 | 936563-96-1 | Src | 88 | 199.7729852 | <1 |  | C25H24N6O2 |
| S1638 | Ibuprofen | 206.28 | 15687-27-1 | COX | 41 | 198.7589684 | <1 |  | C13H18O2 |
| S2118 | Ibutilide Fumarate | 885.23 | 122647-32-9 | Sodium Channel | 89 | 100.538843 | 89 | 100.538843 | C44H76N4O10S2 |
| S2312 | Icariin | 676.66 | 489-32-7 | PDE | 50 | 73.89235362 | <1 |  | C33H40O15 |
| S2662 | ICG-001 | 548.63 | 780757-88-2 | Wnt/beta-catenin | 100 | 182.2722053 | <1 |  | C33H32N4O4 |
| S2922 | Icotinib | 391.42 | 610798-31-7 | EGFR | 78 | 199.2744367 | <1 |  | C22H21N3O4 |
| S7327 | ID-8 | 298.29 | 147591-46-6 | Others | 60 | 201.1465353 | <1 |  | C16H14N2O4 |
| S7205 | *Idasanutlin (RG-7388)* | *616.48* | 1229705-06-9 | Mdm2 | 100 | 162.211264 | <1 |  | C31H29Cl2F2N3O4 |
| S2605 | Idebenone | 338.44 | 58186-27-9 | Others | 68 | 200.9218768 | <1 |  | C19H30O5 |
| S1883 | Idoxuridine | 354.1 | 54-42-2 | Others | 35 | 98.84213499 | <1 |  | C9H11IN2O5 |
| S2860 | IEM 1754 dihydrobroMide | 412.25 | 162831-31-4 | 5-HT Receptor | 82 | 198.9084294 | 82 | 198.9084294 | C16H32Br2N2 |
| S4091 | Ifenprodil Tartrate | 475.53 | 23210-58-4 | Others | 95 | 199.7770908 | 9 | 18.92625071 | C46H60N2O10 |
| S1302 | Ifosfamide | 261.09 | 3778-73-2 | DNA/RNA Synthesis | 52 | 199.1650389 | 52 | 199.1650389 | C7H15Cl2N2O2P |
| S2882 | IKK-16 (IKK Inhibitor VII) | 483.63 | 873225-46-8 | IKK | 97 | 200.5665488 | <1 |  | C28H29N5OS |
| S7157 | Ilomastat (GM6001, Galardin) | 388.46 | 142880-36-2 | MMP | 78 | 200.79 | <1 |  | C20H28N4O4 |
| S1483 | Iloperidone | 426.48 | 133454-47-4 | Others | 27 | 63.30894766 | <1 |  | C24H27FN2O4 |
| S7566 | IM-12 | 377.41 | 1129669-05-1 | GSK-3 | 75 | 198.7228743 | <1 |  | C22H20FN3O2 |
| S2475 | Imatinib (STI571) | 493.6 | 152459-95-5 | PDGFR,c-Kit, v-Abl |  | 10 |  |  | C29H31N7O |
| S1026 | Imatinib Mesylate (STI571) | 589.71 | 220127-57-1 | PDGFR, c-Kit, Bcr-Abl | 118 | 200.0983534 | 118 | 200.0983534 | C30H35N7O4S |
| S2864 | IMD 0354 | 383.67 | 978-62-1 | IKK | 10 | 26.06406547 | <1 |  | C15H8ClF6NO2 |
| S2109 | Imidapril HCl | 441.91 | 89371-37-9 | RAAS | 88 | 199.1355706 | 54 | 122.1968274 | C20H27N3O6 |
| S7587 | INCB024360 | 271.64 | 914471-09-3 | IDO | 54 | 198.7925195 | <1 |  | C9H7ClFN5O2 |
| S3083 | Indacaterol Maleate | 508.56 | 753498-25-8 | Adrenergic Receptor | 102 | 200.5663049 | <1 |  | C28H32N2O7 |
| S1730 | Indapamide | 365.83 | 26807-65-8 | Others | 73 | 199.5462373 | <1 |  | C16H16ClN3O3S |
| S2386 | Indirubin | 262.26 | 479-41-4 | GSK-3 | 53 | 202.0895295 | <1 |  | C16H10N2O2 |
| S2313 | Indole-3-carbinol | 147.18 | 700-06-1 | Others | 29 | 197.037641 | 7 | 47.56080989 | C9H9NO |
| S1723 | Indomethacin | 357.79 | 53-86-1 | Others | 72 | 201.2353615 | <1 |  | C19H16ClNO4 |
| S7493 | INH1 | 308.4 | 313553-47-8 | Microtubule Associated | 61 | 197.7950713 | <1 |  | C18H16N2OS |
| S7494 | INH6 | 322.42 | 1001753-24-7 | Microtubule Associated | 64 | 198.4988524 | <1 |  | C19H18N2OS |
| S1087 | Iniparib (BSI-201) | 292.03 | 160003-66-7 | PARP | 58 | 198.6097319 | <1 |  | C7H5IN2O3 |
| S2811 | INK 128 (MLN0128) | 309.33 | 1224844-38-5 | mTOR | 62 | 200.4331943 | <1 |  | C15H15N7O |
| S1132 | INO-1001 | 136.15 | 3544-24-9 | PARP | 27 | 198.3106867 | <1 |  | C7H8N2O |
| S2442 | Inosine | 268.23 | 58-63-9 | Others | 53 | 197.5916191 | 47 | 175.2227566 | C10H12N4O5 |
| S7329 | IOWH032 | 545.18 | 1191252-49-9 | CFTR | 100 | 183.4256576 | <1 |  | C22H15Br2N3O4 |
| S7234 | IOX1 | 189.17 | 5852-78-8 | Histone demethylases | 37 | 195.5912671 | <1 |  | C10H7NO3 |
| S2919 | IOX2 | 352.34 | 931398-72-0 | HIF | 7 | 19.86717375 | <1 |  | C19H16N2O5 |
| S7093 | IPA-3 | 350.45 | 42521-82-4 | PAK | 70 | 199.7431873 | <1 |  | C20H14O2S2 |
| S7028 | IPI-145 (INK1197) | 416.86 | 1201438-56-3 | PI3K | 83 | 199.1076141 | <1 |  | C22H17ClN6O |
| S1683 | Ipratropium Bromide | 412.37 | 22254-24-6 | Others | 83 | 201.2755535 | 83 | 201.2755535 | C20H30BrNO3 |
| S2422 | Ipriflavone (Osteofix) | 280.32 | 35212-22-7 | Others | 56 | 199.7716895 | <1 |  | C18H16O3 |
| S1198 | Irinotecan | 586.68 | 97682-44-5 | Topoisomerase | 7 | 11.93154701 | <1 |  | C33H38N4O6 |
| S2217 | Irinotecan HCl Trihydrate | 677.18 | 136572-09-3 | Topoisomerase | 100 | 147.6712248 | 1 | 1.476712248 | C33H45ClN4O9 |
| S1929 | Irsogladine | 256.09 | 57381-26-7 | Others |  | 10 |  |  | C9H7Cl2N5 |
| S2534 | Isoconazole nitrate | 479.14 | 24168-96-5 | Others | 41 | 85.56997955 | <1 |  | C18H15Cl4N3O4 |
| S2404 | Isoliquiritigenin | 256.25 | 961-29-5 | Others | 51 | 199.0243902 | <1 |  | C15H12O4 |
| S1937 | Isoniazid | 137.14 | 54-85-3 | Angiogenesis | 27 | 196.8791016 | 27 | 196.8791016 | C6H7N3O |
| S2566 | Isoprenaline HCl | 247.72 | 51-30-9 | Adrenergic Receptor | 50 | 201.840788 | 50 | 201.840788 | C11H18ClNO3 |
| S4204 | Isosorbide | 146.14 | 652-67-5 | Others | 29 | 198.4398522 | 29 | 198.4398522 | C6H10O4 |
| S1379 | Isotretinoin | 300.44 | 4759-48-2 | Hydroxylase | 60 | 199.7070963 | <1 |  | C20H28O2 |
| S4116 | Isovaleramide | 101.15 | 541-46-8 | Others | 20 | 197.7261493 | <1 |  | C5H11NO |
| S7914 | *Isoxazole 9 (ISX-9)* | *234.27* | 832115-62-5 | Others | 46 | 196.3546335 | <1 |  | C11H10N2O2S |
| S1452 | Ispinesib (SB-715992) | 517.06 | 336113-53-2 | Kinesin | 103 | 199.2031873 | <1 |  | C30H33ClN4O2 |
| S1662 | Isradipine | 371.39 | 75695-93-1 | Others | 74 | 199.2514607 | <1 |  | C19H21N3O5 |
| S2790 | Istradefylline | 384.43 | 155270-99-8 | Others | 6 | 15.60752283 | <1 |  | C20H24N4O4 |
| S2476 | Itraconazole | 705.65031 | 84625-61-6 | Others |  | 10 |  |  | C35H38Cl2N8O4 |
| S7134 | IU1 | 300.37 | 314245-33-5 | Proteasome | 60 | 199.7536372 | <1 |  | C18H21FN2O |
| S2086 | Ivabradine HCl | 505.05 | 148849-67-6 | Adrenergic Receptor | 101 | 199.9802 | 82 | 162.3601624 | C27H37ClN2O5 |
| S1144 | Ivacaftor (VX-770) | 392.49 | 873054-44-5 | CFTR | 78 | 198.7311779 | <1 |  | C24H28N2O3 |
| S1351 | Ivermectin | 875.09 | 70288-86-7 | Others | 175 | 199.9794307 | <1 |  | C48H74O14 |
| S7301 | IWP-L6 | 472.58 | 1427782-89-5 | Wnt/beta-catenin | 25 | 52.90109611 | <1 |  | C25H20N4O2S2 |
| S7086 | IWR-1-endo | 409.44 | 1127442-82-3 | Wnt/beta-catenin | 30 | 73.27080891 | <1 |  | C26H21N3O3 |
| S7281 | *JIB-04* | *308.76* | 199596-05-9 | Histone demethylases | 25 | 80.96903744 | <1 |  | C17H13ClN4 |
| S2828 | JNJ-1661010 | 365.45 | 681136-29-8 | FAAH | 36 | 98.50868792 | <1 |  | C19H19N5OS |
| S1172 | JNJ-26854165 (Serdemetan) | 328.41 | 881202-45-5 | p53 | 66 | 200.9683018 | <1 |  | C21H20N4 |
| S1114 | JNJ-38877605 | 377.35 | 943540-75-8 | c-Met | 37 | 98.05220617 | <1 |  | C19H13F2N7 |
| S1249 | JNJ-7706621 | 394.36 | 443797-96-4 | CDK, Aurora Kinase | 79 | 200.3245765 | <1 |  | C15H12F2N6O3S |
| S2905 | JNJ-7777120 | 277.75 | 459168-41-3 | Histamine Receptor | 56 | 201.620162 | <1 |  | C14H16ClN3O |
| S7508 | JNK Inhibitor IX | 332.42 | 312917-14-9 | JNK | 20 | 60.16485169 | <1 |  | C20H16N2OS |
| S4901 | JNK-IN-8 | 507.59 | 1410880-22-6 | JNK | 100 | 197.0093973 | <1 |  | C29H29N7O2 |
| S7351 | JSH-23 | 240.34 | 749886-87-1 | NF-κB | 48 | 199.7170675 | <1 |  | C16H20N2 |
| S2722 | JTC-801 | 447.96 | 244218-51-7 | Opioid Receptor | 90 | 200.9107956 | <1 |  | C26H26ClN3O2 |
| S4904 | JZL184 | 520.49 | 1101854-58-3 | Others | 100 | 192.1266499 | <1 |  | C27H24N2O9 |
| S7359 | K02288 | 352.38 | 1431985-92-0 | TGF-beta/Smad | 70 | 198.6491855 | <1 |  | C20H20N2O4 |
| S2314 | Kaempferol | 286.23 | 520-18-3 | Others | 57 | 199.1405513 | <1 |  | C15H10O6 |
| S7658 | *Kartogenin* | *317.34* | 4727-31-5 | TGF-beta/Smad | 63 | 198.5252411 | <1 |  | C20H15NO3 |
| S1353 | Ketoconazole | 531.43 | 65277-42-1 | P450 |  | 10 |  |  | C26H28Cl2N4O4 |
| S1645 | Ketoprofen | 254.28 | 22071-15-4 | COX | 51 | 200.5663049 | <1 |  | C16H14O3 |
| S1646 | Ketorolac | 376.4 | 74103-07-4 | COX | 75 | 199.2561105 | <1 |  | C15H13NO3 |
| S2024 | Ketotifen Fumarate | 425.5 | 34580-14-8 | Histamine Receptor | 8 | 18.80141011 | <1 |  | C23H23NO5S |
| S2906 | Ki16198 | 488.98 | 355025-13-7 | LPA Receptor | 96 | 196.3270481 | <1 |  | C24H25ClN2O5S |
| S1315 | Ki16425 | 474.96 | 355025-24-0 | LPA Receptor | 94 | 197.9114031 | <1 |  | C23H23ClN2O5S |
| S1363 | Ki8751 | 469.41 | 228559-41-9 | VEGFR, c-Kit, PDGFR | 47 | 100.1256897 | <1 |  | C24H18F3N3O4 |
| S2316 | Kinetin | 215.21 | 525-79-1 | Others | 8 | 37.17299382 | <1 |  | C10H9N5O |
| S7422 | KN-62 | 721.84 | 127191-97-3 | Others | 100 | 138.5348554 | <1 |  | C38H35N5O6S2 |
| S7423 | KN-93 Phosphate | 599.03 | 1188890-41-6 | CaMK | 100 | 166.9365474 | 92 | 153.5816236 | C26H32ClN2O8PS |
| S7750 | *KNK437* | *245.23* | 218924-25-5 | HSP (e.g. HSP90) | 15 | 61.16706765 | <1 |  | C13H11NO4 |
| S7125 | KPT-185 | 355.31 | 1333151-73-7 | CRM1 | 71 | 199.8255045 | <1 |  | C16H16F3N3O3 |
| S7251 | KPT-276 | 426.26 | 1421919-75-6 | CRM1 | 20 | 46.91972036 | <1 |  | C16H10F8N4O |
| S7252 | KPT-330 | 443.31 | 1393477-72-9 | CRM1 | 89 | 200.7624461 | <1 |  | C17H11F6N7O |
| S7331 | *K-Ras(G12C) inhibitor 12* | *449.67* | 1469337-95-8 | Rho | 27 | 60.04403229 | <1 |  | C15H17ClIN3O3 |
| S7333 | K-Ras(G12C) inhibitor 6 | 405.34 | NA | Rho | 81 | 199.8322396 | <1 |  | C17H22Cl2N2O3S |
| S7332 | K-Ras(G12C) inhibitor 9 | 513.78 | 1469337-91-4 | Rho | 53 | 103.1569933 | <1 |  | C16H21ClIN3O4S |
| S1557 | KRN 633 | 416.86 | 286370-15-8 | VEGFR, PDGFR | 9 | 21.58998225 | <1 |  | C20H21ClN4O4 |
| S1226 | KU-0063794 | 465.54 | 938440-64-3 | mTOR | 16 | 34.36869012 | <1 |  | C25H31N5O4 |
| S1092 | KU-55933 (ATM Kinase Inhibitor) | 395.49 | 587871-26-9 | ATM | 33 | 83.44079496 | <1 |  | C21H17NO3S2 |
| S1570 | KU-60019 | 547.67 | 925701-49-1 | ATM | 18 | 32.8665072 | <1 |  | C30H33N3O5S |
| S2158 | KW-2449 | 332.4 | 1000669-72-6 | Flt, Bcr-Abl, Aurora Kinase | 67 | 201.5643803 | <1 |  | C20H20N4O |
| S2685 | KW-2478 | 574.66 | 819812-04-9 | HSP | 115 | 200.1183308 | <1 |  | C30H42N2O9 |
| S2700 | KX2-391 | 431.53 | 897016-82-9 | Src | 86 | 199.2908952 | <1 |  | C26H29N3O3 |
| S7096 | KY02111 | 376.86 | 1118807-13-8 | Wnt/beta-catenin | 75 | 199.012896 | <1 |  | C18H17ClN2O3S |
| S2317 | L-(+)-Rhamnose Monohydrate | 182.17 | 10030-85-0 | Others | 36 | 197.6176099 | 36 | 197.6176099 | C6H14O6 |
| S4291 | Labetalol HCl | 364.87 | 32780-64-6 | Adrenergic Receptor | 72 | 197.3305561 | 8 | 21.92561734 | C19H25ClN2O3 |
| S1994 | Lacidipine | 455.54 | 103890-78-4 | Calcium Channel | 91 | 199.7629187 | <1 |  | C26H33NO6 |
| S1511 | *Lactulose* | *342.3* | 4618-18-2 | Others | 68 | 198.6561496 | 68 | 198.6561496 | C12H22O11 |
| S2522 | L-Adrenaline | 183.2 | 51-43-4 | Others | 4 | 21.83406114 | <1 |  | C9H13NO3 |
| S2260 | Laetrile | 457.43 | 29883-15-6 | Others | 91 | 198.9375424 | <1 |  | C20H27NO11 |
| S2065 | Lafutidine | 431.55 | 118288-08-7 | Histamine Receptor | 86 | 199.2816591 | <1 |  | C22H29N3O4S |
| S1706 | Lamivudine | 229.26 | 134678-17-4 | Others | 46 | 200.6455553 | 46 | 200.6455553 | C8H11N3O3S |
| S3024 | Lamotrigine | 256.09 | 84057-84-1 | Sodium Channel | 10 | 39.04877192 | <1 |  | C9H7Cl2N5 |
| S1354 | Lansoprazole | 369.36 | 103577-45-3 | Proton Pump | 74 | 200.3465454 | <1 |  | C16H14F3N3O2S |
| S2111 | Lapatinib | 581.06 | 231277-92-2 | EGFR, HER2 | 100 | 172.0992669 | <1 |  | C29H26ClFN4O4S |
| S1028 | Lapatinib (GW-572016) Ditosylate | 925.46 | 388082-77-7 | EGFR, HER2 | 100 | 108.054373 | <1 |  | C43H42ClFN4O10S3 |
| S2387 | Lappaconite HBr | 665.61 | 97792-45-5 | Others | 28 | 42.07 | <1 |  | C32H45BrN2O8 |
| S1095 | LAQ824 (Dacinostat) | 379.459 | 404951-53-7 | HDAC | 76 | 200.2851428 | <1 |  | C22H25N3O3 |
| S2787 | Laquinimod | 356.8 | 248281-84-7 | Others | 61 | 170.9641256 | <1 |  | C19H17ClN2O3 |
| S2532 | L-Ascorbyl 6-palmitate | 414.53 | 137-66-6 | Others | 83 | 200.2267628 | <1 |  | C22H38O7 |
| S7467 | LB42708 | 555.46 | 226929-39-1 | Transferase | 100 | 180.0309653 | <1 |  | C30H27BrN4O2 |
| S2388 | *L-carnitine* | *161.2* | 541-15-1 | Others | 12 | 74.44168734 | 32 | 198.5111663 | C7H15NO3 |
| S7009 | *LCL161* | *500.63* | 1005342-46-0 | IAP | 100 | 199.7483171 | <1 |  | C26H33FN4O3S |
| S7678 | *LCZ696* | *915.98* | 936623-90-4 | RAAS | 30 | 32.75180681 | 100 | 109.1726894 | C48H58N6Na3O8R+3 |
| S7461 | LDC000067 | 370.43 | 1073485-20-7 | CDK | 74 | 199.7678374 | <1 |  | C18H18N4O3S |
| S7638 | *LDC1267* | *560.55* | 1361030-48-9 | Axl | 100 | 178.396218 | <1 |  | C30H26F2N4O5 |
| S2151 | LDE225 (NVP-LDE225,Erismodegib) | 485.5 | 956697-53-3 | Smoothened | 97 | 199.7940268 | <1 |  | C26H26F3N3O3 |
| S7083 | LDK378 | 558.14 | 1032900-25-6 | ALK | 20 | 35.83330347 | <1 |  | C28H36ClN5O3S |
| S7147 | LDN-212854 | 406.48 | 1432597-26-6 | TGF-beta/Smad | 81 | 199.2717969 | <1 |  | C25H22N6 |
| S7627 | *LDN-214117* | *419.52* | 1627503-67-6 | TGF-beta/Smad | 83 | 197.8451564 | <1 |  | C25H29N3O3 |
| S7135 | LDN-57444 | 397.64 | 668467-91-2 | Proteasome | 11 | 27.66321296 | <1 |  | C17H11Cl3N2O3 |
| S7579 | *Ledipasvir (GS5885)* | *889.0* | 1256388-51-8 | HCV Protease | 100 | 112.4859393 | <1 |  | C49H54F2N8O6 |
| S7440 | LEE011 | 434.54 | 1211441-98-3 | CDK | 7 | 16.10898882 | <1 |  | C23H30N8O |
| S1247 | Leflunomide | 270.21 | 75706-12-6 | Others | 54 | 199.8445653 | <1 |  | C12H9F3N2O2 |
| S1029 | Lenalidomide (CC-5013) | 259.26 | 191732-72-6 | TNF-alpha | 52 | 200.5708555 | <1 |  | C13H13N3O3 |
| S1164 | Lenvatinib (E7080) | 426.85 | 417716-92-8 | VEGFR | 40 | 93.7097341 | <1 |  | C21H19ClN4O4 |
| S1235 | Letrozole | 285.3 | 112809-51-5 | Aromatase | 57 | 199.7896951 | <1 |  | C17H11N5 |
| S7380 | Leupeptin Hemisulfate | 475.59 | 103476-89-7 | Cysteine Protease,Serine Protease | 95 | 199.75 | 95 | 199.7518871 | C20H38N6O4R |
| S1356 | Levetiracetam | 170.21 | 102767-28-2 | Others | 34 | 199.753246 | 34 | 199.753246 | C8H14N2O2 |
| S4085 | Levobetaxolol HCl | 343.89 | 116209-55-3, 93221-48-8(free base) | Others | 69 | 200.6455553 | 69 | 200.6455553 | C18H30ClNO3 |
| S4061 | *Levobupivacaine HCl* | *324.89* | 27262-48-2 | Sodium Channel | 64 | 196.9897504 | 64 | 196.9897504 | C18H29ClN2O |
| S4131 | Levodropropizine | 236.31 | 99291-25-5 | Histamine Receptor | 47 | 198.8912869 | 15 | 63.47594262 | C13H20N2O2 |
| S1940 | Levofloxacin | 361.37 | 100986-85-4 | Others | 24 | 66.41392479 | 11 | 30.43971553 | C18H20FN3O4 |
| S1727 | Levonorgestrel | 312.45 | 797-63-7 | Others | 45 | 144.0230437 | <1 |  | C21H28O2 |
| S2446 | Levosimendan | 280.28 | 141505-33-1 | Others | 56 | 199.8001998 | <1 |  | C14H12N6O |
| S2104 | Levosulpiride | 341.43 | 23672-07-3 | Dopamine Receptor | 69 | 202.0912046 | <1 |  | C15H23N3O4S |
| S7734 | *LFM-A13* | *360* | 244240-24-2 | BTK | 72 | 200 | <1 |  | C11H8Br2N2O2 |
| S7143 | LGK-974 | 396.44 | 1243244-14-5 | Wnt/beta-catenin | 79 | 199.2735345 | <1 |  | C23H20N6O |
| S7828 | *Licochalcone A* | *338.4* | 58749-22-7 | Estrogen/progestogen Receptor | 67 | 197.9905437 | <1 |  | C21H22O4 |
| S2121 | Licofelone | 379.88 | 156897-06-2 | COX | 76 | 200.0631778 | <1 |  | C23H22ClNO2 |
| S1357 | Lidocaine | 234.34 | 137-58-6 | Histamine Receptor | 47 | 200.5632841 | 9 | 38.40573526 | C14H22N2O |
| S2319 | Limonin | 470.51 | 1180-71-8 | HIV Protease | 44 | 93.52 | <1 |  | C26H30O8 |
| S3031 | Linagliptin | 472.54 | 668270-12-0 | DPP-4 | 17 | 35.97579041 | <1 |  | C25H28N8O2 |
| S2479 | Lincomycin HCl | 443 | 859-18-7 | Others | 89 | 200.9029345 | 89 | 200.9029345 | C18H35ClN2O6S |
| S1408 | Linezolid | 337.35 | 165800-03-3 | Others | 67 | 198.6067882 | <1 |  | C16H20FN3O4 |
| S1003 | Linifanib (ABT-869) | 375.41 | 796967-16-3 | PDGFR, VEGFR | 75 | 199.7815721 | <1 |  | C21H18FN5O |
| S4217 | Liothyronine Sodium | 672.96 | 55-06-1 | Others | 100 | 148.597242 | <1 |  | C15H11I3NNaO4 |
| S7699 | *Liproxstatin-1* | *340.85* | 950455-15-9 | Ferroptosis | 68 | 199.5012469 | <1 |  | C19H21ClN4 |
| S4003 | Lithocholic acid | 376.57 | 434-13-9 | Others | 75 | 199.1661577 | <1 |  | C24H40O3 |
| S7569 | *LMK-235* | *294.35* | 1418033-25-6 | HDAC | 58 | 197.044335 | <1 |  | C15H22N2O4 |
| S8056 | Lomeguatrib | 326.17 | 192441-08-0 | DNA Methyltransferase | 65 | 199.2825827 | <1 |  | C10H8BrN5OS |
| S4084 | Lomerizine HCl | 541.46 | 101477-54-7, 101477-55-8(free base) | Others | 100 | 184.6858494 | <1 |  | C27H32Cl2F2N2O3 |
| S7635 | Lomitapide | 692.71 | 182431-12-5 | others | 100 | 144.36 | <1 |  | C39H37F6N3O2 |
| S7633 | *Lomitapide Mesylate* | *789.83* | 202914-84-9 | Others | 100 | 126.6095236 | <1 |  | C40H41F6N3O5S |
| S1840 | Lomustine | 233.7 | 13010-47-4 | Others | 46 | 196.8335473 | <1 |  | C9H16ClN3O2 |
| S2797 | Lonafarnib | 638.82 | 193275-84-2 | Others | 127 | 198.804045 | <1 |  | C27H31Br2ClN4O2 |
| S2610 | Lonidamine | 321.16 | 50264-69-2 | Others | 64 | 199.2776186 | <1 |  | C15H10Cl2N2O2 |
| S2480 | Loperamide HCl | 513.5 | 34552-83-5 | Opioid Receptor | 22 | 42.84323272 | <1 |  | C29H34Cl2N2O2 |
| S1380 | Lopinavir | 628.8 | 192725-17-0 | HIV Protease | 126 | 200.3816794 | <1 |  | C37H48N4O5 |
| S1358 | Loratadine | 382.88 | 79794-75-5 | Histamine Receptor | 17 | 44.40033431 | <1 |  | C22H23ClN2O2 |
| S4109 | Lorcaserin HCl | 232.15 | 846589-98-8 | 5-HT Receptor | 46 | 198.1477493 | 46 | 198.1477493 | C11H15Cl2N |
| S2047 | Lornoxicam | 371.82 | 70374-39-9 | Others |  | 10 |  |  | C13H10ClN3O4S2 |
| S1359 | Losartan Potassium (DuP 753) | 462.01 | 124750-99-8 | RAAS | 92 | 199.129889 | 92 | 199.129889 | C22H23ClKN6O |
| S7215 | Losmapimod (GW856553X) | 383.46 | 585543-15-3 | p38 MAPK | 76 | 198.1953789 | <1 |  | C22H26FN3O2 |
| S1669 | Loteprednol etabonate | 466.95 | 82034-46-6 | Others | 93 | 199.1647928 | <1 |  | C24H31ClO7 |
| S2061 | Lovastatin | 404.54 | 75330-75-5 | HMG-CoA Reductase | 8 | 19.77554754 | <1 |  | C24H36O5 |
| S4086 | Loxapine Succinate | 445.9 | 27833-64-3 | Others | 89 | 199.596322 | <1 |  | C22H24ClN3O5 |
| S7392 | Loxistatin Acid (E-64C) | 314.38 | 76684-89-4 | Cysteine Protease | 62 | 197.2135632 | 2 | 6.361727845 | C15H26N2O5 |
| S7584 | *LRRK2-IN-1* | *570.69* | 1234480-84-2 | LRRK2 | 100 | 175.2264802 | <1 |  | C31H38N8O3 |
| S2599 | L-Thyroxine | 776.87 | 51-48-9 | Others | 100 | 128.7216651 | <1 |  | C15H11I4NO4 |
| S4258 | Luliconazole | 354.28 | 187164-19-8 | Others | 71 | 200.4064582 | <1 |  | C14H9Cl2N3S2 |
| S2903 | Lumiracoxib | 293.72 | 220991-20-8 | COX | 59 | 200.8715784 | <1 |  | C15H13ClFNO2 |
| S2320 | Luteolin | 286.24 | 491-70-3 | PDE | 57 | 199.1335942 | <1 |  | C15H10O6 |
| S7063 | *LY2090314* | *512.53* | 603288-22-8 | GSK-3 | 100 | 195.1105301 | <1 |  | C28H25FN6O3 |
| S7366 | *LY2119620* | *437.94* | 886047-22-9 | AChR | 87 | 198.6573503 | <1 |  | C19H24ClN5O3S |
| S2230 | LY2157299 | 369.42 | 700874-72-2 | TGF-beta/Smad | 74 | 200.3140057 | <1 |  | C22H19N5O |
| S1494 | LY2228820 | 612.74 | 862507-23-1 | p38 MAPK | 4 | 6.528054313 | 100 | 163.2013578 | C26H37FN6O6S2 |
| S7697 | *LY2409881* | *594.43* | 946518-60-1 | IκB/IKK | 20 | 33.64567737 | <1 |  | C24H32Cl4N6OS |
| S2626 | LY2603618 | 436.3 | 911222-45-2 | Chk | 13 | 29.79601192 | <1 |  | C18H22BrN5O3 |
| S2155 | LY2608204 | 559.81 | 1234703-40-2 | Others | 112 | 200.07 | <1 |  | C28H37N3O3S3 |
| S2179 | LY2784544 | 469.94 | 1229236-86-5 | JAK | 94 | 200.0255352 | <1 |  | C23H25ClFN7O |
| S1528 | LY2811376 | 320.36 | 1194044-20-6 | 5-alpha Reductase | 16 | 49.94381321 | <1 |  | C15H14F2N4S |
| S7158 | LY2835219 | 602.7 | 1231930-82-7 | CDK | 83 | 137.713622 | 100 | 165.9200265 | C28H36F2N8O3S |
| S7057 | *LY2874455* | *444.31* | 1254473-64-7 | FGFR | 88 | 198.0599131 | <1 |  | C21H19Cl2N5O2 |
| S2156 | LY2886721 | 390.41 | 1262036-50-9 | Others | 9 | 23.0526882 | <1 |  | C18H16F2N4O2S |
| S1105 | LY294002 | 307.34 | 154447-36-6 | PI3K | 36 | 117.1341186 | <1 |  | C19H17NO3 |
| S2714 | LY411575 | 479.48 | 209984-57-6 | Gamma-secretase | 95 | 198.1313089 | <1 |  | C26H23F2N3O4 |
| S2779 | M344 | 307.39 | 251456-60-7 | HDAC | 62 | 201.6981685 | <1 |  | C16H25N3O3 |
| S8051 | Macitentan | 588.27 | 441798-33-0 | Endothelin Receptor | 100 | 169.9899706 | <1 |  | C19H20Br2N6O4S |
| S2321 | Magnolol | 266.33 | 528-43-8 | Others | 53 | 199.0012391 | <1 |  | C18H18O2 |
| S1137 | Malotilate | 288.38 | 59937-28-9 | Others | 58 | 201.1235176 | <1 |  | C12H16O4S2 |
| S2481 | Manidipine | 610.7 | 89226-50-6 | Calcium Channel | 122 | 199.7707549 | <1 |  | C35H38N4O6 |
| S2482 | Manidipine 2HCl | 683.62 | 89226-75-5 | Calcium Channel | 21 | 30.7188204 | <1 |  | C35H40Cl2N4O6 |
| S2517 | Maprotiline HCl | 313.86 | 10347-81-6 | Reuptake inhibitor | 63 | 200.7264385 | <1 |  | C20H24ClN |
| S2003 | Maraviroc | 513.67 | 376348-65-1 | CCR5 | 100 | 194.6775167 | <1 |  | C29H41F2N5O |
| S7156 | *Marimastat(BB-2516)* | *331.41* | 154039-60-8 | MMP | 54 | 162.9401648 | <1 |  | C15H29N3O5 |
| S1064 | Masitinib (AB1010) | 498.64 | 790299-79-5 | c-Kit, PDGFR, FGFR, FAK | 100 | 200.5454837 | <1 |  | C28H30N6OS |
| S1484 | MC1568 | 314.31 | 852475-26-4 | HDAC | 13 | 41.36044033 | <1 |  | C17H15FN2O3 |
| S7162 | Mdivi-1 | 353.22 | 338967-87-6 | Dynamin | 70 | 198.1767737 | <1 |  | C15H10Cl2N2O2S |
| S7438 | ME0328 | 321.37 | 1445251-22-8 | PARP | 64 | 199.1474002 | <1 |  | C19H19N3O2 |
| S2074 | Mecarbinate | 233.26 | 15574-49-9 | Others | 47 | 201.4918975 | <1 |  | C13H15NO3 |
| S4252 | Mechlorethamine HCl | 192.51 | 55-86-7 | Others | 39 | 202.5868786 | 39 | 202.5868786 | C5H12Cl3N |
| S4295 | Meclofenamate Sodium | 318.13 | 6385-02-0 | COX | 63 | 198.032251 | 63 | 198.032251 | C14H10Cl2NNaO2 |
| S4280 | Meclofenoxate (Centrophenoxine) HCl | 294.17 | 3685-84-5 | Others | 58 | 197.1649046 | 58 | 197.1649046 | C12H17Cl2NO3 |
| S3060 | Medetomidine HCl | 236.74 | 86347-15-1 | Adrenergic Receptor | 12 | 50.68851905 | 47 | 198.5300329 | C13H17ClN2 |
| S2567 | Medroxyprogesterone acetate | 386.52 | 71-58-9 | Estrogen/progestogen Receptor | 12 | 31.04625893 | <1 |  | C24H34O4 |
| S4078 | Mefenamic Acid | 241.29 | 61-68-7 | COX | 48 | 198.9307472 | <1 |  | C15H15NO2 |
| S4420 | Mefloquine HCl | 414.77 | 51773-92-3 | Others | 82 | 197.6999301 | <1 |  | C17H17ClF6N2O |
| S1304 | Megestrol Acetate | 384.51 | 595-33-5 | Androgen Receptor | 33 | 85.82351564 | <1 |  | C24H32O4 |
| S1974 | Meglumine | 195.21 | 6284-40-8 | Others | 39 | 199.7848471 | 39 | 199.7848471 | C7H17NO5 |
| S7007 | MEK162 (ARRY-162, ARRY-438162) | 441.23 | 606143-89-9 | MEK | 88 | 199.4424676 | <1 |  | C17H15BrF2N4O3 |
| S1204 | Melatonin | 232.28 | 73-31-4 | Others | 47 | 202.342001 | <1 |  | C13H16N2O2 |
| S1734 | Meloxicam | 351.4 | 71125-38-7 | Others | 30 | 85.37279454 | <1 |  | C14H13N3O4S2 |
| S2043 | Memantine HCl | 215.76 | 41100-52-1 | AMPA Receptor-kainate Receptor-NMDA Receptor | 43 | 199.2955135 | 30 | 139.0433815 | C12H22ClN |
| S1949 | Menadione | 172.18 | 58-27-5 | Others | 34 | 197.4677663 | <1 |  | C11H8O2 |
| S3155 | Mepivacaine HCl | 282.81 | 1722-62-9 | Others | 3 | 10.60782858 | 57 | 201.548743 | C15H23ClN2O |
| S1689 | Meprednisone | 372.455 | 1247-42-3 | Others | 75 | 201.366608 | <1 |  | C22H28O5 |
| S3204 | Meptazinol HCl | 269.81 | 59263-76-2 | Others | 54 | 200.1408399 | 54 | 200.1408399 | C15H24ClNO |
| S4077 | Mequinol | 124.14 | 150-76-5 | Others | 25 | 201.3855325 | 25 | 201.3855325 | C7H8O2 |
| S1305 | Mercaptopurine (6-MP) | 152.18 | 50-44-2 | DNA/RNA Synthesis | 30 | 197.1349717 | <1 |  | C5H4N4S |
| S1381 | Meropenem | 383.46 | 96036-03-2 | Others | 76 | 198.1953789 | 8 | 20.86267147 | C17H25N3O5S |
| S1681 | Mesalamine | 153.14 | 89-57-6 | Others | 31 | 202.4291498 | <1 |  | C7H7NO3 |
| S1735 | Mesna | 164.18 | 19767-45-4 | Others | 33 | 200.9989036 | 7 | 42.63613108 | C2H5NaO3S2 |
| S2125 | Mestranol | 310.43 | 72-33-3 | NULL | 15 | 48.32007216 | <1 |  | C21H26O2 |
| S2527 | Methacycline HCl | 478.88 | 3963-95-9 | Others | 3 | 6.264617441 | 5 | 10.44102907 | C22H23ClN2O8 |
| S4039 | Methazolamide | 236.27 | 554-57-4 | Carbonic Anhydrase | 47 | 198.9249587 | <1 |  | C5H8N4O3S2 |
| S3139 | Methenamine | 140.19 | 100-97-0 | Others | 28 | 199.7289393 | <1 |  | C6H12N4 |
| S1609 | Methimazole | 114.17 | 60-56-0 | Others | 23 | 201.4539721 | 23 | 201.4539721 | C4H6N2S |
| S1736 | Methocarbamol | 241.24 | 532-03-6 | Others | 48 | 198.9719781 | 2 | 8.290499088 | C11H15NO5 |
| S1210 | Methotrexate | 454.44 | 59-05-2 | DHFR | 90 | 198.0459467 | <1 |  | C20H22N8O5 |
| S1952 | Methoxsalen | 216.19 | 298-81-7 | Others | 43 | 198.8991165 | <1 |  | C12H8O4 |
| S1978 | Methscopolamine | 398.29 | 155-41-9 | AChR | 79 | 198.3479374 | 67 | 168.2191368 | C18H24BrNO4 |
| S4057 | Methyclothiazide | 360.24 | 135-07-9 | Others | 72 | 199.8667555 | <1 |  | C9H11Cl2N3O4S2 |
| S2323 | Methyl-Hesperidin | 642.6 | 11013-97-1 | Others | 100 | 155.6178027 | 100 | 155.6178027 | C29H38O16 |
| S1733 | Methylprednisolone | 374.47 | 83-43-2 | Others | 75 | 200.2830667 | <1 |  | C22H30O5 |
| S3138 | Methylthiouracil | 142.18 | 56-04-2 | Others | 28 | 196.9334646 | <1 |  | C5H6N2OS |
| S4289 | MetoclopraMide HCl | 336.26 | 7232-21-5 | Dopamine Receptor | 67 | 199.2505799 | 67 | 199.2505799 | C14H23Cl2N3O2 |
| S1610 | Metolazone | 365.83 | 17560-51-9 | Others | 73 | 199.5462373 | <1 |  | C16H16ClN3O3S |
| S1856 | Metoprolol Tartrate | 684.81 | 392-17-7 | Adrenergic Receptor | 100 | 146.025905 | 100 | 146.025905 | C34H56N2O12 |
| S1907 | Metronidazole | 171.15 | 443-48-1 | Others | 34 | 198.6561496 | <1 |  | C6H9N3O3 |
| S4223 | Mevastatin | 390.51 | 73573-88-3 | Others | 78 | 199.7388031 | <1 |  | C23H34O5 |
| S4225 | Mexiletine HCl | 215.72 | 5370-01-4 | Others | 43 | 199.332468 | 43 | 199.332468 | C11H18ClNO |
| S4179 | Mezlocillin Sodium | 561.56 | 42057-22-7 | Others | 100 | 178.0753615 | 100 | 178.0753615 | C21H24N5NaO8S2 |
| S7386 | *MG-101 (ALLN)* | *383.53* | 110044-82-1 | Cysteine Protease | 76 | 198.1592053 | <1 |  | C20H37N3O4 |
| S2619 | MG-132 | 475.62 | 133407-82-6 | Proteasome | 95 | 199.7392877 | <1 |  | C26H41N3O5 |
| S7476 | *MG149* | *340.46* | 1243583-85-8 | Histone Acetyltransferase | 68 | 199.7297774 | <1 |  | C22H28O3 |
| S1361 | MGCD-265 | 517.6 | 875337-44-3 | c-Met, VEGFR, Tie-2 | 104 | 200.927357 | <1 |  | C26H20FN5O2S2 |
| S7811 | *MHY1485* | *387.39* | 326914-06-1 | mTOR | 33 | 85.185472 | <1 |  | C17H21N7O4 |
| S7429 | MI-2 (MALT1 inhibitor) | 455.72 | 1047953-91-2 | Others | 91 | 199.6840165 | <1 |  | C19H17Cl3N4O3 |
| S7618 | *MI-2 (Menin-MLL Inhibitor)* | *375.55* | 1271738-62-5 | Histone Methyltransferase | 75 | 199.7070963 | <1 |  | C18H25N5S2 |
| S7619 | *MI-3 (Menin-MLL Inhibitor)* | *375.55* | 1271738-59-0 | Histone Methyltransferase | 19 | 50.59246439 | <1 |  | C18H25N5S2 |
| S7649 | *MI-773 (SAR405838)* | *562.50* | 1303607-60-4 | Mdm2 | 100 | 177.7777778 | <1 |  | C29H34Cl2FN3O3 |
| S1382 | Mianserin HCl | 300.83 | 21535-47-7 | Others | 60 | 199.4481933 | 30 | 99.72409667 | C18H21ClN2 |
| S4287 | Micafungin Sodium | 1292.26 | 208538-73-2 | Others | 100 | 77.38 | 100 | 77.38380821 | C56H70N9NaO23S |
| S2536 | Miconazole | 416.13 | 22916-47-8 | Others | 83 | 199.4569005 | <1 |  | C18H14Cl4N2O |
| S1956 | Miconazole Nitrate | 479.14 | 22832-87-7 | Others | 96 | 200.3589765 | <1 |  | C18H15Cl4N3O4 |
| S2606 | Mifepristone | 429.59 | 84371-65-3 | Estrogen/progestogen Receptor | 85 | 197.8630787 | <1 |  | C29H35NO2 |
| S2589 | Miglitol | 207.22 | 72432-03-2 | Others | 3 | 14.47736705 | <1 |  | C8H17NO5 |
| S2751 | Milciclib (PHA-848125) | 460.57 | 802539-81-7 | CDK | 92 | 199.7524806 | <1 |  | C25H32N8O |
| S3140 | Milnacipran HCl | 282.81 | 101152-94-7 | Others | 57 | 201.548743 | 57 | 201.548743 | C15H23ClN2O |
| S2484 | Milrinone | 211.22 | 78415-72-2 | ATPase | 42 | 198.8448064 | <1 |  | C12H9N3O |
| S4009 | Mirabegron | 396.51 | 223673-61-8 | Adrenergic Receptor | 79 | 199.2383546 | <1 |  | C21H24N4O2S |
| S2016 | Mirtazapine | 265.35 | 85650-52-8 | Others | 53 | 199.7361975 | <1 |  | C17H19N3 |
| S1732 | Mitotane | 320.04 | 53-19-0 | Others | 64 | 199.9750031 | <1 |  | C14H10Cl4 |
| S1889 | Mitoxantrone | 444.48 | 65271-80-9 | Topoisomerase | 88 | 197.9841613 | <1 |  | C22H28N4O6 |
| S2485 | Mitoxantrone HCl | 517.4 | 70476-82-3 | Others | 89 | 172.0139157 | 89 | 172.0139157 | C22H30Cl2N4O6 |
| S2660 | MK-0752 | 442.9 | 471905-41-6 | Gamma-secretase | 89 | 200.9482953 | <1 |  | C21H21ClF2O4S |
| S1525 | MK-1775 | 500.6 | 955365-80-7 | Wee1 | 80 | 159.8082301 | 0.0001 | 0.00019976 | C27H32N8O2 |
| S2765 | MK-2048 | 461.87 | 869901-69-9 | Integrase | 9 | 19.48600255 | <1 |  | C21H21ClFN5O4 |
| S1078 | MK-2206 2HCl | 480.39 | 1032350-13-2 | Akt | 14 | 29.14298799 | <1 |  | C25H23Cl2N5O |
| S2774 | MK-2461 | 495.55 | 917879-39-1 | c-Met, FGFR, PDGFR | 99 | 199.7780244 | <1 |  | C24H25N5O5S |
| S1174 | MK-2866 (GTx-024) | 389.33 | 841205-47-8 | Androgen Receptor | 78 | 200.344181 | <1 |  | C19H14F3N3O3 |
| S2770 | MK-5108 (VX-689) | 461.94 | 1010085-13-8 | Aurora Kinase | 92 | 199.1600641 | <1 |  | C22H21ClFN3O3S |
| S2857 | MK-801 (Dizocilpine) | 221.3 | 77086-21-6 | GluR | 67 | 302.7564392 | <1 |  | C16H15N |
| S1158 | MK-8245 | 467.25 | 1030612-90-8 | Dehydrogenase | 93 | 199.04 | <1 |  | C17H16BrFN6O4 |
| S7065 | MK-8745 | 431.91 | 885325-71-3 | Aurora Kinase | 86 | 199.1155565 | <1 |  | C20H19ClFN5OS |
| S2863 | ML130 (Nodinitib-1) | 287.34 | 799264-47-4 | NOD1 | 57 | 198.3712675 | <1 |  | C14H13N3O2S |
| S2825 | ML133 HCl | 313.82 | 1222781-70-5 | Potassium Channel | 63 | 200.7520235 | <1 |  | C19H20ClNO |
| S7686 | *ML141* | *407.49* | 71203-35-5 | Rho | 81 | 198.7778841 | <1 |  | C22H21N3O3S |
| S2916 | ML161 | 361.23 | 423735-93-7 | Others | 72 | 199.3189934 | <1 |  | C17H17BrN2O2 |
| S7509 | ML167 | 335.36 | 1285702-20-6 | CDK | 67 | 199.7853053 | <1 |  | C19H17N3O3 |
| S7529 | ML323 | 384.48 | 1572414-83-5 | DUB | 76 | 197.6695797 | <1 |  | C23H24N6 |
| S7296 | *ML324* | *349.43* | 1222800-79-4 | Histone Demethylase | 43 | 123.0575509 | <1 |  | C21H23N3O2 |
| S7148 | ML347 | 352.39 | 1062368-49-3 | TGF-beta/Smad | 10 | 28.37764976 | <1 |  | C22H16N4O |
| S2898 | *MLN0905* | *486.56* | 1228960-69-7 | PLK | 97 | 199.3587636 | <1 |  | C24H25F3N6S |
| S2180 | MLN2238 | 361.03 | 1072833-77-2 | Proteasome | 72 | 199.4294103 | <1 |  | C14H19BCl2N2O4 |
| S1100 | MLN8054 | 476.86 | 869363-13-3 | Aurora Kinase | 95 | 199.2198968 | <1 |  | C25H15ClF2N4O2 |
| S2181 | MLN9708 | 517.12 | 1201902-80-8 | Proteasome | 100 | 193.3787129 | <1 |  | C20H23BCl2N2O9 |
| S7265 | MM-102 | 669.8 | 1417329-24-8 | Histone Methyltransferase | 100 | 149.298298 | 100 | 149.298298 | C35H49F2N7O4 |
| S4921 | MNS (3,4-Methylenedioxy-β-nitrostyrene, MDBN) | 193.16 | 1485-00-3 | Others | 39 | 201.9051563 | <1 |  | C9H7NO4 |
| S1122 | Mocetinostat (MGCD0103) | 396.44 | 726169-73-9 | HDAC | 13 | 32.79184744 | <1 |  | C23H20N6O |
| S3212 | Moclobemide (Ro 111163) | 268.74 | 71320-77-9 | MAO | 53 | 197.2166406 | <1 |  | C13H17ClN2O2 |
| S2079 | Moexipril HCl | 535.03 | 82586-52-5 | RAAS | 20 | 37.38108143 | <1 |  | C27H35ClN2O7 |
| S3104 | Moguisteine | 339.41 | 119637-67-1 | Others | 68 | 200.3476621 | <1 |  | C16H21NO5S |
| S1987 | Mometasone furoate | 521.43 | 83919-23-7 | Others | 6 | 11.50681779 | <1 |  | C27H30Cl2O6 |
| S1652 | Monobenzone | 200.23 | 103-16-2 | Others | 40 | 199.7702642 | <1 |  | C13H12O2 |
| S7721 | *Monomethyl auristatin E (MMAE)* | *717.98* | 474645-27-7 | Others | 100 | 139.2796457 | <1 |  | C39H67N5O7 |
| S4211 | Montelukast Sodium | 608.17 | 151767-02-1 | Others | 100 | 164.4277094 | 100 | 164.4277094 | C35H35ClNNaO3S |
| S2325 | Morin Hydrate | 320.25 | 6202-27-3 | Others | 64 | 199.843872 | <1 |  | C15H12O8 |
| S2486 | Moroxydine HCl | 207.66 | 3160-91-6 | Others | 42 | 202.2536839 | 42 | 202.2536839 | C6H14ClN5O |
| S1385 | Mosapride Citrate | 614.02 | 112885-42-4 | 5-HT Receptor | 90 | 146.5750301 | <1 |  | C27H33ClFN3O10 |
| S1032 | Motesanib Diphosphate (AMG-706) | 569.44 | 857876-30-3 | VEGFR, PDGFR, c-Kit | 100 | 175.6111267 | 19 | 33.36611408 | C22H29N5O9P2 |
| S1465 | Moxifloxacin HCl | 437.89 | 186826-86-8 | Topoisomerase | 88 | 200.9637123 | <1 |  | C21H25ClFN3O4 |
| S2066 | Moxonidine | 241.68 | 75438-57-2 | Others | 24 | 99.30486594 | <1 |  | C9H12ClN5O |
| S2809 | MPEP | 193.24 | 96206-92-7 | GluR | 39 | 201.821569 | <1 |  | C14H11N |
| S7488 | MPI-0479605 | 407.51 | 1246529-32-7 | Kinesin | 62 | 152.1435057 | <1 |  | C22H29N7O |
| S2855 | MRS 2578 | 472.67 | 711019-86-2 | P2 Receptor | 42 | 88.8569192 | <1 |  | C20H20N6S4 |
| S7305 | *MS436* | *383.42* | 1395084-25-9 | Epigenetic Reader Domain | 55 | 143.4458296 | <1 |  | C18H17N5O3S |
| S2216 | Mubritinib (TAK 165) | 468.47 | 366017-09-6 | HER2 | 13 | 27.74990928 | <1 |  | C25H23F3N4O2 |
| S4297 | Mupirocin | 500.62 | 12650-69-0 | DNA/RNA Synthesis | 100 | 199.7523071 | 46 | 91.88606128 | C26H44O9 |
| S1501 | Mycophenolate Mofetil | 433.49 | 128794-94-5 | Others | 86 | 198.3898129 | <1 |  | C23H31NO7 |
| S2487 | Mycophenolic acid | 320.34 | 24280-93-1 | Others | 64 | 199.7877255 | <1 |  | C17H20O6 |
| S2326 | Myricetin | 318.24 | 529-44-2 | NULL | 63 | 197.9638009 | <1 |  | C15H10O8 |
| S2327 | Myricitrin | 464.38 | 17912-87-7 | Others | 93 | 200.2670227 | <1 |  | C21H20O12 |
| S7589 | N6022 | 414.46 | 1208315-24-5 | Others | 82 | 197.847802 | <1 |  | C24H22N4O3 |
| S3190 | N6-methyladenosine (m6A) | 281.27 | 1867-73-8 | Others | 56 | 199.10 | 14 | 49.77423828 | C11H15N5O4 |
| S4051 | Nabumetone | 228.29 | 42924-53-8 | COX | 46 | 201.4980945 | <1 |  | C15H16O2 |
| S3105 | Nadifloxacin | 360.38 | 124858-35-1 | Others | 20 | 55.49697541 | <1 |  | C19H21FN2O4 |
| S1386 | Nafamostat Mesylate | 539.58 | 82956-11-4 | Proteasome | 20 | 37.06586604 | 53 | 98.22454502 | C21H25N5O8S2 |
| S4042 | Nafcillin Sodium | 454.47 | 7177-50-6 | Others | 91 | 200.2332387 | 91 | 200.2332387 | C21H23N2NaO6S |
| S2126 | Naftopidil | 392.49 | 57149-07-2 | Adrenergic Receptor | 79 | 201.2790135 | <1 |  | C24H28N2O3 |
| S1387 | Naftopidil DiHCl | 465.41 | 57149-08-3 | Adrenergic Receptor | 41 | 88.09436841 | <1 |  | C24H30Cl2N2O3 |
| S2328 | Nalidixic acid | 232.24 | 389-08-2 | Others | 6 | 25.83534275 | <1 |  | C12H12N2O3 |
| S3066 | Naloxone HCl | 363.84 | 357-08-4 | Opioid Receptor | 73 | 200.6376429 | 73 | 200.6376429 | C19H22ClNO4 |
| S4501 | Naloxone HCl Dihydrate | 399.87 | 51481-60-8 | Opioid Receptor | 79 | 197.56 | 20 | 50.01625528 | C19H26ClNO6 |
| S2103 | Naltrexone HCl | 377.86 | 16676-29-2 | Opioid Receptor | 14 | 37.05075954 | 14 | 37.05075954 | C20H24ClNO4 |
| S2519 | Naphazoline HCl | 246.74 | 550-99-2 | Adrenergic Receptor | 17 | 68.8984356 | 49 | 198.5896085 | C14H15ClN2 |
| S1626 | Naproxen | 252.24 | 26159-34-2 | COX | 3 | 11.89343482 | 50 | 198.2239137 | C14H13NaO3 |
| S1488 | Naratriptan | 371.93 | 143388-64-1 | 5-HT Receptor | 74 | 198.9621703 | 22 | 59.15091549 | C17H26ClN3O2S |
| S2394 | Naringenin | 272.25 | 480-41-1 | P450 | 54 | 198.3471074 | <1 |  | C15H12O5 |
| S2329 | Naringin | 580.53 | 10236-47-2 | Others | 116 | 199.8174082 | <1 |  | C27H32O14 |
| S2389 | Naringin Dihydrochalcone | 582.55 | 18916-17-1 | Others | 100 | 171.6590851 | <1 |  | C27H34O14 |
| S1517 | Natamycin | 665.73 | 7681-93-8 | Others | 7 | 10.51477326 | <1 |  | C33H47NO13 |
| S2489 | Nateglinide | 317.42 | 105816-04-4 | Potassium Channel | 63 | 198.4752064 | <1 |  | C19H27NO3 |
| S1549 | Nebivolol | 441.9 | 152520-56-4 | Adrenergic Receptor | 88 | 199.1400769 | <1 |  | C22H26ClF2NO4 |
| S8037 | Necrostatin-1 | 259.33 | 4311-88-0 | TNF-alpha | 51 | 196.6606255 | <1 |  | C13H13N3OS |
| S1969 | Nefiracetam | 246.3 | 77191-36-7 | GABA Receptor | 49 | 198.9443768 | 5 | 20.30044661 | C14H18N2O2 |
| S1213 | Nelarabine | 297.27 | 121032-29-9 | DNA/RNA Synthesis | 60 | 201.8367141 | 3 | 10.0918357 | C11H15N5O5 |
| S4282 | Nelfinavir Mesylate | 663.89 | 159989-65-8 | HIV Protease | 100 | 150.627363 | <1 |  | C33H49N3O7S2 |
| S2332 | Neohesperidin | 610.56 | 13241-33-3 | Others | 122 | 199.8165618 | <1 |  | C28H34O15 |
| S2331 | Neohesperidin dihydrochalcone (Nhdc) | 612.58 | 20702-77-6 | Others | 100 | 163.2439845 | <1 |  | C28H36O15 |
| S1255 | Nepafenac | 254.28 | 78281-72-8 | Others | 50 | 196.6336322 | <1 |  | C15H14N2O2 |
| S2695 | *Nepicastat (SYN-117) HCl* | *331.81* | 170151-24-3 | Hydroxylase | 66 | 198.9090142 | <1 |  | C14H16ClF2N3S |
| S1742 | Nevirapine | 266.3 | 129618-40-2 | Others | 53 | 199.0236575 | <1 |  | C15H14N4O |
| S7473 | Nexturastat A | 341.4 | 1403783-31-2 | Nexturastat A | 68 | 199.1798477 | <1 |  | C19H23N3O3 |
| S7436 | NH125 | 524.56 | 278603-08-0 | Others | 100 | 190.6359616 | <1 |  | C27H45IN2 |
| S4265 | Nicaraven | 284.31 | 79455-30-4 | Others | 57 | 200.4853857 | 57 | 200.4853857 | C15H16N4O2 |
| S4181 | Nicardipine HCl | 515.99 | 54527-84-3 | Others | 100 | 193.8022055 | <1 |  | C26H30ClN3O6 |
| S1971 | Nicorandil | 211.17 | 65141-46-0 | Others | 42 | 198.8918881 | 17 | 80.50385945 | C8H9N3O4 |
| S1899 | Nicotinamide (Vitamin B3) | 122.12 | 98-92-0 | NULL | 24 | 196.5280052 | 24 | 196.5280052 | C6H6N2O |
| S1744 | Nicotinic Acid | 123.11 | 59-67-6 | Others | 25 | 203.0704248 | 2 | 16.24563399 | C6H5NO2 |
| S1808 | Nifedipine | 346.33 | 21829-25-4 | Others | 69 | 199.2319464 | <1 |  | C17H18N2O6 |
| S3018 | Niflumic acid | 282.22 | 4394-00-7 | GABA Receptor | 56 | 198.4267593 | <1 |  | C13H9F3N2O2 |
| S4182 | Nifuroxazide | 275.22 | 965-52-6 | Others | 55 | 199.8401279 | <1 |  | C12H9N3O5 |
| S1033 | Nilotinib (AMN-107) | 529.52 | 641571-10-0 | Bcr-Abl | 27 | 50.98957546 | <1 |  | C28H22F3N7O |
| S2721 | Nilvadipine | 385.37 | 75530-68-6 | Calcium Channel | 77 | 199.8079767 | <1 |  | C19H19N3O6 |
| S2040 | Nimesulide | 308.31 | 51803-78-2 | Others | 62 | 201.0962992 | <1 |  | C13H12N2O5S |
| S1747 | Nimodipine | 418.44 | 66085-59-4 | Others | 84 | 200.7456266 | <1 |  | C21H26N2O7 |
| S1010 | Nintedanib (BIBF 1120) | 539.62 | 656247-17-5 | VEGFR, PDGFR, FGFR | 6 | 11.11893555 | <1 |  | C31H33N5O4 |
| S1748 | Nisoldipine | 388.41 | 63675-72-9 | Others | 77 | 198.2441235 | <1 |  | C20H24N2O6 |
| S1627 | Nitazoxanide | 307.28 | 55981-09-4 | Others | 62 | 201.7703723 | <1 |  | C12H9N3O5S |
| S4422 | *Nitenpyram* | *270.72* | 150824-47-8 | AChR | 54 | 199.4680851 | 54 | 199.4680851 | C11H15ClN4O2 |
| S4232 | Nithiamide | 187.18 | 140-40-9 | Others | 37 | 197.6706913 | <1 |  | C5H5N3O3S |
| S2491 | Nitrendipine | 360.3699 | 39562-70-4 | Calcium Channel | 72 | 199.7947109 | <1 |  | C18H20N2O6 |
| S1644 | Nitrofural | 198.14 | 59-87-0 | Others | 40 | 201.8774604 | <1 |  | C6H6N4O4 |
| S1890 | Nizatidine | 331.46 | 76963-41-2 | Others | 66 | 199.1190491 | 28 | 84.47474808 | C12H21N5O2S2 |
| S7111 | NLG919 | 282.38 | 1402836-58-1 | IDO | 15 | 53.11990934 | <1 |  | C18H22N2O |
| S7072 | NMDA (N-Methyl-D-aspartic acid) | 147.13 | 6384-92-5 | GluR | 5 | 33.98355196 | 30 | 203.9013118 | C5H9NO4 |
| S7285 | NMS-873 | 520.67 | 1418013-75-8 | p97 | 100 | 192.0602301 | <1 |  | C27H28N4O3S2 |
| S7282 | NMS-E973 | 454.43 | 1253584-84-7 | HSP | 90 | 198.0503048 | <1 |  | C22H22N4O7 |
| S7255 | NMS-P937 (NMS1286937) | 532.52 | 1034616-18-6 | PLK | 42 | 78.87 | <1 |  | C24H27F3N8O3 |
| S2333 | Nobiletin | 402.39 | 478-01-3 | Others | 81 | 201.2972489 | <1 |  | C21H22O8 |
| S2775 | Nocodazole | 301.32 | 31430-18-9 | Microtubule Associated | 7 | 23.23111642 | <1 |  | C14H11N3O3S |
| S2615 | Noradrenaline bitartrate monohydrate | 337.28 | 108341-18-0 | Others | 64 | 189.7533207 | 64 | 189.7533207 | C12H19NO10 |
| S4040 | Norethindrone | 298.42 | 68-22-4 | Others | 60 | 201.0589103 | <1 |  | C20H26O2 |
| S2492 | Novobiocin Sodium | 634.61 | 1476-53-5 | Others | 127 | 200.1229101 | 127 | 200.1229101 | C31H35N2NaO11 |
| S7669 | *NPS-1034* | *551.54* | 1221713-92-3 | c-Met | 100 | 181.3105124 | <1 |  | C31H23F2N5O3 |
| S2633 | NPS-2143 | 408.92 | 284035-33-2 | CaSR | 82 | 200.5282207 | <1 |  | C24H25ClN2O2 |
| S8031 | NSC 23766 | 530.96 | 1177865-17-6 | Rac | 100 | 188.3381046 | 100 | 188.3381046 | C24H38Cl3N7 |
| S7149 | NSC 319726 | 234.32 | 71555-25-4 | p53 | 19 | 81.08569478 | <1 |  | C11H14N4S |
| S8072 | NSC 405020 | 260.16 | 7497-07-6 | MMP | 52 | 199.8769988 | <1 |  | C12H15Cl2NO |
| S7142 | NSC697923 | 267.26 | 343351-67-7 | E2 conjugating | 53 | 198.308763 | <1 |  | C11H9NO5S |
| S7730 | *NU1025* | *176.17* | 90417-38-2 | PARP | 35 | 198.6717375 | <1 |  | C9H8N2O2 |
| S7114 | NU6027 | 251.28 | 220036-08-8 | CDK | 50 | 198.9812162 | <1 |  | C11H17N5O2 |
| S2638 | NU7441 (KU-57788) | 413.49 | 503468-95-9 | DNA-PK, PI3K |  | 10 |  |  | C25H19NO3S |
| S1061 | Nutlin-3 | 581.5 | 548472-68-0 | Mdm2 | 100 | 171.9690456 | <1 |  | C30H30Cl2N4O4 |
| S8059 | Nutlin-3a | 581.49 | 675576-98-4 | Mdm2 | 100 | 171.972003 | <1 |  | C30H30Cl2N4O4 |
| S8065 | Nutlin-3b | 581.49 | 675576-97-3 | Mdm2 | 100 | 171.972003 | <1 |  | C30H30Cl2N4O4 |
| S1034 | NVP-AEW541 | 439.55 | 475489-16-8 | IGF-1R | 88 | 200.2047549 | <1 |  | C27H29N5O |
| S2202 | NVP-BHG712 | 503.48 | 940310-85-0 | VEGFR, Src, Raf, Bcr-Abl | 101 | 200.6037976 | <1 |  | C26H20F3N7O |
| S2686 | NVP-BSK805 2HCl | 563.47 | 1092499-93-8 (free base) | JAK | 113 | 200.5430635 | 3 | 5.324152129 | C27H30Cl2F2N6O |
| S2761 | NVP-BVU972 | 340.38 | 1185763-69-2 | c-Met | 68 | 199.7767201 | <1 |  | C20H16N6 |
| S6002 | NXY-059 | 381.33 | 168021-79-2 | Others | 76 | 199.3024415 | 76 | 199.3024415 | C11H13NNa2O7S2 |
| S1934 | Nystatin (Fungicidin) | 926.09 | 1400-61-9 | Others | 19 | 20.5163645 | <1 |  | C47H75NO17 |
| S7217 | OAC1 | 237.26 | 300586-90-7 | Others | 47 | 198.094917 | <1 |  | C14H11N3O |
| S1057 | Obatoclax Mesylate (GX15-070) | 413.49 | 803712-79-0 | Bcl-2 | 83 | 200.7303683 | <1 |  | C21H23N3O4S |
| S7660 | *Obeticholic Acid* | *420.63* | 459789-99-2 | FXR | 84 | 199.7004493 | <1 |  | C26H44O4 |
| S2822 | OC000459 | 348.37 | 851723-84-7, 950688-14-9 (sodium salt) | GPR | 4 | 11.48204495 | <1 |  | C21H17FN2O2 |
| S1115 | Odanacatib (MK-0822) | 525.56 | 603139-19-1 | Cathepsin K | 100 | 190.2732324 | <1 |  | C25H27F4N3O3S |
| S7681 | *OF-1* | *440.31* | 919973-83-4 | Epigenetic Reader Domain | 76 | 172.6056642 | <1 |  | C17H18BrN3O4S |
| S7237 | OG-L002 | 225.29 | 1357302-64-7 | Histone demethylases | 45 | 199.742554 | <1 |  | C15H15NO |
| S2493 | Olanzapine | 312.43975 | 132539-06-1 | 5-HT Receptor, Dopamine Receptor | 63 | 201.638876 | <1 |  | C17H20N4S |
| S1060 | Olaparib (AZD2281, Ku-0059436) | 434.46 | 763113-22-0 | PARP | 86 | 197.9468766 | 0.002 | 0.004603416 | C24H23FN4O3 |
| S2334 | Oleanolic Acid | 456.7 | 508-02-1 | Others | 21 | 45.98204511 | <1 |  | C30H48O3 |
| S7867 | *Oleuropein* | *540.51* | 32619-42-4 | Others | 100 | 185.0104531 | 100 | 185.0104531 | C25H32O13 |
| S1604 | Olmesartan Medoxomil | 558.59 | 144689-63-4 | RAAS | 89 | 159.329741 | <1 |  | C29H30N6O6 |
| S2494 | Olopatadine HCl | 373.87 | 140462-76-6 | Histamine Receptor | 75 | 200.6044882 | 13 | 34.77144462 | C21H24ClNO3 |
| S4041 | Olsalazine Sodium | 346.2 | 6054-98-4 | Others | 4 | 11.55401502 | 41 | 118.428654 | C14H8N2Na2O6 |
| S7864 | *Oltipraz* | *226.34* | 64224-21-1 | Others | 39 | 172.3071485 | <1 |  | C8H6N2S3 |
| S7672 | *Omaveloxolone (RTA-408)* | *554.71* | 1474034-05-3 | Others | 100 | 180.2743776 | <1 |  | C33H44F2N2O3 |
| S2623 | Omecamtiv mecarbil (CK-1827452) | 401.43 | 873697-71-3 | ATPase | 80 | 199.287547 | <1 |  | C20H24FN5O3 |
| S1389 | Omeprazole | 345.42 | 73590-58-6 | Proton Pump, ATPase | 69 | 199.7568178 | <1 |  | C17H19N3O3S |
| S1390 | Ondansetron HCl | 329.82 | 99614-01-4 | 5-HT Receptor | 66 | 200.1091504 | 24 | 72.7669638 | C18H20ClN3O |
| S7877 | *ONO-4059* | *456.50* | 1351635-67-0 | BTK | 20 | 43.81161008 | <1 |  | C25H24N6O3 |
| S7172 | ONX-0914 (PR-957) | 580.67 | 960374-59-8 | Proteasome | 100 | 172.2148553 | <1 |  | C31H40N4O7 |
| S7049 | Oprozomib (ONX 0912) | 532.61 | 935888-69-0 | Proteasome | 100 | 187.7546422 | <1 |  | C25H32N4O7S |
| S8073 | Optovin | 315.41 | 348575-88-2 | Others | 63 | 199.7400209 | <1 |  | C15H13N3OS2 |
| S1534 | Org 27569 | 409.95 | 868273-06-7 | Cannabinoid Receptor | 82 | 200.0243932 | <1 |  | C24H28ClN3O |
| S2335 | Oridonin | 364.43 | 28957-04-2 | Others | 73 | 200.31 | <1 |  | C20H28O6 |
| S1629 | Orlistat | 495.73 | 96829-58-2 | Others | 99 | 199.7054848 | <1 |  | C29H53NO5 |
| S3121 | Ornidazole | 219.63 | 16773-42-5 | Others | 44 | 200.3369303 | 2 | 9.106224104 | C7H10ClN3O3 |
| S2336 | Orotic acid (6-Carboxyuracil) | 156.1 | 65-86-1 | Others | 2 | 12.81229981 | <1 |  | C5H4N2O4 |
| S2054 | Orphenadrine Citrate | 461.5 | 4682-36-4 | AChR | 92 | 199.3499458 | <1 |  | C24H31NO8 |
| S2624 | OSI-027 | 406.44 | 936890-98-1 | mTOR | 18 | 44.28697963 | <1 |  | C21H22N6O3 |
| S2205 | OSI-420 | 415.87 | 183320-51-6 | EGFR | 83 | 199.5816 | <1 |  | C21H22ClN3O4 |
| S1091 | OSI-906 (Linsitinib) | 421.49 | 867160-71-2 | IGF-1R | 84 | 199.2929844 | <1 |  | C26H23N5O |
| S1220 | OSI-930 | 443.44 | 728033-96-3 | c-Kit, VEGFR | 89 | 200.7035901 | <1 |  | C22H16F3N3O2S |
| S4285 | Ospemifene | 378.89 | 128607-22-7 | Estrogen/progestogen Receptor | 75 | 197.9466336 | <1 |  | C24H23ClO2 |
| S2337 | Osthole | 244.29 | 484-12-8 | Others | 49 | 200.5812764 | <1 |  | C15H16O3 |
| S1106 | OSU-03012 (AR-12) | 460.45 | 742112-33-0 | PDK-1 | 11 | 23.88967315 | <1 |  | C26H19F3N4O |
| S3047 | Otilonium Bromide | 563.57 | 26095-59-0 | AChR | 113 | 200.5074791 | 113 | 200.5074791 | C29H43BrN2O4 |
| S7360 | OTX015 | 491.99 | 202590-98-5 | BET | 98 | 199.1910405 | <1 |  | C25H22ClN5O2S |
| S4016 | Ouabain | 728.77 | 630-60-4 | Sodium Channel | 100 | 137.2175035 | <1 |  | C29H60O20 |
| S1224 | Oxaliplatin | 397.29 | 61825-94-3 | DNA/RNA Synthesis | 14 | 35.23874248 | 0.01 | 0.02517053 | C8H14N2O4Pt |
| S4230 | Oxaprozin | 293.32 | 21256-18-8 | Others | 59 | 201.1455066 | <1 |  | C18H15NO3 |
| S1391 | Oxcarbazepine | 252.27 | 28721-07-5 | Sodium Channel | 7 | 27.74804773 | <1 |  | C15H12N2O2 |
| S1830 | Oxfendazole | 315.35 | 53716-50-0 | Others | 10 | 31.71079753 | <1 |  | C15H13N3O3S |
| S4270 | Oxiracetam | 158.16 | 62613-82-5 | Others | 31 | 196.0040465 | 31 | 196.0040465 | C6H10N2O3 |
| S4229 | Oxybuprocaine HCl | 344.88 | 5987-82-6 | Others | 69 | 200.0695894 | 69 | 200.0695894 | C17H29ClN2O3 |
| S1754 | *Oxybutynin* | *357.49* | 5633-20-5 | Others | 71 | 198.606954 | <1 |  | C22H31NO3 |
| S3117 | Oxybutynin chloride | 393.95 | 1508-65-2 | AChR | 79 | 200.5330626 | 4 | 10.15357279 | C22H32ClNO3 |
| S2338 | Oxymatrine | 264.36 | 16837-52-8 | Others | 53 | 200.4841882 | 53 | 200.4841882 | C15H24N2O2 |
| S2495 | Oxymetazoline hydrochloride | 296.84 | 2315-02-8 | Adrenergic Receptor | 59 | 198.7602749 | 59 | 198.7602749 | C16H25ClN2O |
| S1773 | Oxytetracycline (Terramycin) | 460.43 | 79-57-2 | NULL | 92 | 199.8132181 | <1 |  | C22H24N2O9 |
| S2052 | *Oxytetracycline Dihydrate* | *496.46* | 6153-64-6 | Others | 5 | 10.07130484 | <1 |  | C22H28N2O11 |
| S2496 | Ozagrel | 228.25 | 82571-53-7 | Factor Xa | 46 | 201.5334064 | <1 |  | C13H12N2O2 |
| S2067 | Ozagrel HCl | 264.71 | 78712-43-3 | Others | 53 | 200.2191077 | 53 | 200.2191077 | C13H13ClN2O2 |
| S7133 | P22077 | 315.32 | 1247819-59-5 | DUB | 63 | 199.7970316 | <1 |  | C12H7F2NO3S2 |
| S8058 | P276-00 | 438.3 | 920113-03-7 | CDK | 88 | 200.7757244 | 88 | 200.7757244 | C21H21Cl2NO5 |
| S7132 | P5091 (P005091) | 348.22 | 882257-11-6 | DUB | 28 | 80.40893688 | <1 |  | C12H7Cl2NO3S2 |
| S1162 | PA-824 | 359.26 | 187235-37-6 | Others | 72 | 200.4119579 | <1 |  | C14H12F3N3O5 |
| S2738 | PAC-1 | 392.49 | 315183-21-2 | Caspase | 78 | 198.7311779 | <1 |  | C23H28N4O2 |
| S1150 | Paclitaxel | 853.91 | 33069-62-4 | Microtubule Associated | 171 | 200.2552962 | <1 |  | C47H51NO14 |
| S8057 | Pacritinib (SB1518) | 472.58 | 937272-79-2 | JAK | 11 | 23.27648229 | <1 |  | C28H32N4O3 |
| S2410 | Paeoniflorin | 480.46 | 23180-57-6 | Others | 96 | 199.8085168 | 96 | 199.8085168 | C23H28O11 |
| S2339 | Paeonol | 166.17 | 552-41-0 | Others | 33 | 198.5918036 | <1 |  | C9H10O3 |
| S1116 | Palbociclib (PD-0332991) HCl | 483.99 | 827022-32-2, 571190-30-2 (free base) | CDK | 3 | 6.198475175 | 30 | 61.98475175 | C24H30ClN7O2 |
| S2397 | Palmatine chloride | 387.86 | 10605-02-4 | Others | 77 | 198.5252411 | 5 | 12.89124942 | C21H22ClNO4 |
| S2238 | Palomid 529 (P529) | 406.43 | 914913-88-5 | mTOR | 81 | 199.30 | <1 |  | C24H22O6 |
| S2497 | Pancuronium dibromide | 732.67 | 15500-66-0 | AChR | 100 | 136.4870951 | 100 | 136.4870951 | C35H60Br2N2O4 |
| S1030 | Panobinostat (LBH589) | 349.43 | 404950-80-7 | HDAC | 69 | 197.4644421 | <1 |  | C21H23N3O2 |
| S3005 | Paroxetine HCl | 365.83 | 78246-49-8 | 5-HT Receptor | 73 | 199.5462373 | 10 | 27.335101 | C19H21ClFNO3 |
| S3012 | Pazopanib | 437.52 | 444731-52-6 | VEGFR | 87 | 198.8480527 | <1 |  | C21H23N7O2S |
| S1035 | Pazopanib HCl | 473.98 | 635702-64-6 | VEGFR, PDGFR, c-Kit | 17 | 35.86649226 | <1 |  | C21H24ClN7O2S |
| S1090 | PCI-24781 (Abexinostat) | 397.42 | 783355-60-2 | HDAC | 80 | 201.2983745 | <1 |  | C21H23N3O5 |
| S2012 | PCI-34051 | 296.32 | 950762-95-5 | HDAC | 59 | 199.1090713 | <1 |  | C17H16N2O3 |
| S7424 | PD 151746 | 237.25 | 179461-52-0 | Cysteine Protease | 47 | 198.1032666 | <1 |  | C11H8FNO2S |
| S1036 | PD0325901 | 482.19 | 391210-10-9 | MEK | 96 | 199.0916444 | <1 |  | C16H14F3IN2O4 |
| S7098 | PD123319 | 508.61 | 130663-39-7 | RAAS | 100 | 196.6143017 | 100 | 196.6143017 | C31H32N4O3 |
| S2168 | PD128907 HCl | 285.77 | 112960-16-4 | Dopamine Receptor | 12 | 41.9918116 | 50 | 174.9658817 | C14H20ClNO3 |
| S7039 | PD168393 | 369.22 | 194423-15-9 | EGFR | 74 | 200.4225123 | <1 |  | C17H13BrN4O |
| S1264 | PD173074 | 523.67 | 219580-11-7 | FGFR, VEGFR | 100 | 190.9599557 | <1 |  | C28H41N7O3 |
| S7269 | PD173955 | 443.35 | 260415-63-2 | Bcr-Abl | 15 | 33.83331454 | <1 |  | C21H18Cl2N4OS |
| S1020 | PD184352 (CI-1040) | 478.67 | 212631-79-3 | MEK | 96 | 200.5557064 | <1 |  | C17H14ClF2IN2O2 |
| S1568 | PD318088 | 561.09 | 391210-00-7 | MEK | 112 | 199.6114705 | <1 |  | C16H13BrF3IN2O4 |
| S1177 | PD98059 | 267.28 | 167869-21-8 | MEK | 14 | 52.37952709 | <1 |  | C16H13NO3 |
| S1855 | Pefloxacin Mesylate | 429.46 | 70458-95-6 | Others | 14 | 32.59907791 | 67 | 156.0098729 | C18H24FN3O6S |
| S4119 | Pefloxacin Mesylate Dihydrate | 465.49 | 149676-40-4 | Topoisomerase | 9 | 19.33446476 | 67 | 143.9343488 | C18H28FN3O8S |
| S1392 | Pelitinib (EKB-569) | 467.92 | 257933-82-7 | EGFR | 13 | 27.78252693 | <1 |  | C24H23ClFN5O2 |
| S4184 | Penciclovir | 253.26 | 39809-25-1 | Others | 34 | 134.249388 | 1 | 3.948511411 | C10H15N5O3 |
| S4151 | Penfluridol | 523.97 | 26864-56-2 | Others | 100 | 190.8506212 | <1 |  | C28H27ClF5NO |
| S4160 | Penicillin G Sodium | 356.37 | 69-57-8 | Others | 71 | 199.2311362 | 71 | 199.2311362 | C16H17N2NaO4S |
| S4007 | Pentamidine | 413.34 | 50357-45-4 | Others | 119 | 287.8985823 | 119 | 287.8985823 | C19H26Cl2N4O2 |
| S4143 | Pentoxyverine Citrate | 525.59 | 23142-01-0 | Others | 100 | 190.2623718 | 100 | 190.2623718 | C21H32N3O5P |
| S4000 | Pergolide mesylate | 410.59 | 66104-23-2 | Dopamine Receptor | 10 | 24.35519618 | 82 | 199.7126087 | C20H30N2O3S2 |
| S7818 | *Pexidartinib (PLX3397)* | *417.81* | 1029044-16-3 | CSF-1R | 83 | 198.654891 | <1 |  | C20H15ClF3N5 |
| S7799 | *Pexmetinib (ARRY-614)* | *556.63* | 945614-12-0 | p38 MAPK | 100 | 179.652552 | <1 |  | C31H33FN6O3 |
| S2672 | PF-00562271 | 665.66 | 939791-38-5 | FAK | 14 | 21.03175795 | <1 |  | C27H26F3N7O6S2 |
| S1094 | PF-04217903 | 372.38 | 956905-27-4 | c-Met | 5 | 13.42714431 | <1 |  | C19H16N8O |
| S7192 | PF-04620110 | 396.44 | 1109276-89-2 | Transferase | 49 | 123.6000404 | <1 |  | C21H24N4O4 |
| S2743 | PF-04691502 | 425.48 | 1013101-36-4 | mTOR, PI3K, Akt | 14 | 32.90401429 | <1 |  | C22H27N5O4 |
| S2656 | PF-04929113 (SNX-5422) | 521.53 | 908115-27-5 | HSP | 104 | 199.4132648 | <1 |  | C25H30F3N5O4 |
| S7536 | *PF-06463922* | *406.41* | 1454846-35-5 | ALK | 81 | 199.3061194 | <1 |  | C21H19FN6O2 |
| S2687 | *PF-2545920* | *392.45* | 1292799-56-4 | PDE | 78 | 198.7514333 | <1 |  | C25H20N4O |
| S2222 | PF-3716556 | 394.47 | 928774-43-0 | ATPase,Proton Pump | 79 | 200.27 | <1 |  | C22H26N4O3 |
| S7094 | PF-3758309 | 490.62 | 898044-15-0 | PAK | 98 | 199.7472586 | <1 |  | C25H30N8OS |
| S2666 | PF-3845 | 456.46 | 1196109-52-0 | FAAH | 91 | 199.3602944 | <1 |  | C24H23F3N4O2 |
| S7644 | *PF-431396* | *506.5* | 717906-29-1 | FAK | 100 | 197.4333662 | <1 |  | C22H21F3N6O3S |
| S2163 | PF-4708671 | 390.41 | 1255517-76-0 | S6 Kinase | 30 | 76.842294 | <1 |  | C19H21F3N6 |
| S2904 | PF-477736 | 419.48 | 952021-60-2 | Chk | 6 | 14.30342329 | <1 |  | C22H25N7O2 |
| S2921 | PF-4981517 | 456.59 | 1390637-82-7 | P450 | 91 | 199.3035327 | <1 |  | C26H32N8 |
| S7675 | *PF-4989216* | *380.4* | 1276553-09-3 | PI3K | 76 | 199.7896951 | <1 |  | C18H13FN6OS |
| S2777 | PF-5274857 | 436.96 | 1373615-35-0 | Hedgehog/Smoothened | 93 | 212.83 | 93 | 212.8341267 | C20H25ClN4O3S |
| S7177 | PF-543 | 465.6 | 1415562-82-1 | S1P Receptor | 93 | 199.742268 | <1 |  | C27H31NO4S |
| S2890 | PF-562271 | 507.49 | 717907-75-0 | FAK | 100 | 197.0482177 | <1 |  | C21H20F3N7O3S |
| S7357 | PF-562271 HCl | 543.95 | 939791-41-0 | FAK | 100 | 183.8404265 | <1 |  | C21H21ClF3N7O3S |
| S2013 | PF-573228 | 491.49 | 869288-64-2 | FAK | 26 | 52.9003642 | <1 |  | C22H20F3N5O3S |
| S1216 | PFI-1 (PF-6405761) | 347.39 | 1403764-72-6 | Others | 69 | 198.6240249 | <1 |  | C16H17N3O4S |
| S7294 | *PFI-2* | *499.52* | 1627676-59-8 | Histone Methyltransferase | 99 | 198.1902627 | 4 | 8.00768738 | C23H25F4N3O3S |
| S7315 | *PFI-3* | *321.37* | 1819363-80-8 | Epigenetic Reader Domain | 64 | 199.1474002 | <1 |  | C19H19N3O2 |
| S7289 | PFK15 | 260.29 | 4382-63-2 | Others | 19 | 72.99550501 | <1 |  | C17H12N2O |
| S2726 | PH-797804 | 477.3 | 586379-66-0 | p38 MAPK | 96 | 201.1313639 | <1 |  | C22H19BrF2N2O3 |
| S1070 | PHA-665752 | 641.61 | 477575-56-7 | c-Met | 128 | 199.4981375 | <1 |  | C32H34Cl2N4O4S |
| S1454 | PHA-680632 | 501.62 | 398493-79-3 | Aurora Kinase | 100 | 199.3540927 | <1 |  | C28H35N7O2 |
| S2742 | PHA-767491 | 249.7 | 942425-68-5 | CDK | 24 | 96.11 | <1 |  | C12H11N3O.HCl |
| S1487 | PHA-793887 | 361.48 | 718630-59-2 | CDK | 72 | 199.1811442 | <1 |  | C19H31N5O2 |
| S2577 | Phenacetin | 179.22 | 62-44-2 | COX | 36 | 200.8704386 | <1 |  | C10H13NO2 |
| S4235 | Phenazopyridine HCl | 249.7 | 136-40-3 | Others | 50 | 200.2402883 | <1 |  | C11H12ClN5 |
| S2542 | Phenformin HCl | 241.72 | 834-28-6 | AMPK | 48 | 198.5768658 | 48 | 198.5768658 | C10H16ClN5 |
| S1921 | Phenindione | 222.24 | 83-12-5 | Others | 44 | 197.9841613 | <1 |  | C15H10O2 |
| S4045 | Pheniramine Maleate | 356.42 | 132-20-7 | Others | 71 | 199.2031873 | 71 | 199.2031873 | C20H24N2O4 |
| S4251 | Phenothiazine | 199.27 | 92-84-2 | Others | 40 | 200.7326743 | <1 |  | C12H9NS |
| S2499 | Phenoxybenzamine HCl | 340.3 | 63-92-3 | Androgen Receptor | 68 | 199.823685 | 17 | 49.95592125 | C18H23Cl2NO |
| S2038 | Phentolamine Mesylate | 377.46 | 65-28-1 | adrenergic receptor | 76 | 201.345838 | 76 | 201.345838 | C18H23N3O4S |
| S1654 | Phenylbutazone | 308.37 | 50-33-9 | Others | 62 | 201.0571716 | <1 |  | C19H20N2O2 |
| S2569 | Phenylephrine HCl | 203.67 | 61-76-7 | Adrenergic Receptor | 41 | 201.3060343 | 41 | 201.3060343 | C9H14ClNO2 |
| S2525 | Phenytoin | 252.27 | 57-41-0 | Sodium Channel | 50 | 198.2003409 | <1 |  | C15H12N2O2 |
| S2524 | Phenytoin sodium | 274.25 | 630-93-3 | Sodium Channel | 32 | 116.6818596 | 3 | 10.93892434 | C15H11N2NaO2 |
| S2342 | Phloretin | 274.27 | 60-82-2 | Others | 54 | 196.8862799 | <1 |  | C15H14O5 |
| S2343 | Phlorizin | 436.41 | 60-81-1 | Others | 87 | 199.3538187 | <1 |  | C21H24O10 |
| S7382 | Phosphoramidon Disodium Salt | 588.48 | 164204-38-0 | Others | 100 | 169.9293094 | 100 | 169.9293094 | C23H34N3Na2O10P |
| S1556 | PHT-427 | 409.61 | 1191951-57-1 | Akt, PDK-1 | 82 | 200.190425 | <1 |  | C20H31N3O2S2 |
| S1038 | PI-103 | 348.36 | 371935-74-9 | DNA-PK, PI3K, mTOR | 24 | 68.89424733 | <1 |  | C19H16N4O3 |
| S7462 | PI-1840 | 394.47 | 1401223-22-0 | Proteasome | 78 | 197.733668 | <1 |  | C22H26N4O3 |
| S7623 | *PI-3065* | *506.64* | 955977-50-1 | PI3K | 50 | 98.68940471 | <1 |  | C27H31FN6OS |
| S3026 | Piceatannol | 244.24 | 10083-24-6 | Others | 48 | 196.5280052 | <1 |  | C14H12O4 |
| S7668 | *Picropodophyllin (PPP)* | *414.41* | 477-47-4 | IGF-1R | 82 | 197.871673 | <1 |  | C22H22O8 |
| S3106 | Pidotimod | 244.27 | 121808-62-6 | Others | 49 | 200.5976993 | 10 | 40.93830597 | C9H12N2O4S |
| S2929 | Pifithrin-α (PFTα) | 367.3 | 63208-82-2 | p53 | 67 | 182.4121971 | <1 |  | C16H19BrN2OS |
| S2930 | Pifithrin-μ | 181.21 | 64984-31-2 | p53 | 36 | 198.6645329 | <1 |  | C8H7NO2S |
| S2207 | PIK-293 | 397.43 | 900185-01-5 | PI3K | 80 | 201.2933095 | <1 |  | C22H19N7O |
| S2227 | PIK-294 | 489.53 | 900185-02-6 | PI3K | 98 | 200.1920209 | <1 |  | C28H23N7O2 |
| S1205 | PIK-75 | 488.74 | 372196-77-5 | PI3K, DNA-PK |  | 10 |  |  | C16H15BrClN5O4S |
| S1489 | PIK-93 | 389.88 | 593960-11-3 | PI3K, VEGFR | 78 | 200.0615574 | <1 |  | C14H16ClN3O4S2 |
| S7645 | *Pilaralisib (XL147)* | *541.02* | 934526-89-3 | PI3K | 100 | 184.8360504 | <1 |  | C25H25ClN6O4S |
| S4231 | Pilocarpine HCl | 244.72 | 54-71-7 | Others | 49 | 200.228833 | 49 | 200.228833 | C11H17ClN2O2 |
| S1475 | Pimasertib (AS-703026) | 431.2 | 1236699-92-5 | MEK | 86 | 199.4434137 | <1 |  | C15H15FIN3O3 |
| S5004 | *Pimecrolimus* | *810.45* | 137071-32-0 | Others | 100 | 123.3882411 | <1 |  | C43H68ClNO11 |
| S1550 | Pimobendan | 334.37 | 74150-27-9 | PDE | 67 | 200.3768281 | <1 |  | C19H18N4O2 |
| S2590 | Pioglitazone | 356.44 | 111025-46-8 | Others | 15 | 42.08281899 | <1 |  | C19H20N2O3S |
| S2046 | Pioglitazone HCl | 392.9 | 112529-15-4 | Others | 79 | 201.0689743 | <1 |  | C19H21ClN2O3S |
| S4222 | Piperacillin Sodium | 539.54 | 59703-84-3 | Others | 100 | 185.34307 | 100 | 185.34307 | C23H26N5NaO7S |
| S2344 | Piperine | 285.34 | 94-62-2 | Others | 57 | 199.7616878 | <1 |  | C17H19NO3 |
| S7551 | *Piperlongumine* | *317.34* | 20069-09-4 | Others | 16 | 50.41910884 | <1 |  | C17H19NO5 |
| S3070 | Piracetam | 142.16 | 7491-74-9 | Others | 72 | 506.4715813 | 72 | 506.4715813 | C6H10N2O2 |
| S1393 | Pirarubicin | 627.64 | 72496-41-4 | Topoisomerase | 7 | 11.15 | <1 |  | C32H37NO12 |
| S2907 | Pirfenidone | 185.22 | 53179-13-8 | TGF-beta/Smad | 37 | 199.7624447 | <1 |  | C12H11NO |
| S1713 | Piroxicam | 331.35 | 36322-90-4 | COX | 66 | 199.1851517 | <1 |  | C15H13N3O4S |
| S1759 | Pitavastatin Calcium | 880.98 | 147526-32-7 | Others | 51 | 57.89007696 | <1 |  | C50H46CaF2N2O8 |
| S1394 | Pizotifen Malate | 429.53 | 5189-11-7 | Others | 21 | 48.89064792 | <1 |  | C23H27NO5S |
| S2886 | PJ34 | 295.34 | 344458-19-1 | PARP | 28 | 94.81 | <1 |  | C17H17N3O2 |
| S7300 | PJ34 HCl | 331.8 | 344458-15-7 | PARP | 66 | 198.915009 | 66 | 198.915009 | C17H18ClN3O2 |
| S1176 | *Plinabulin (NPI-2358)* | *336.39* | 714272-27-2 | VDA | 54 | 160.5279586 | <1 |  | C19H20N4O2 |
| S8076 | PluriSIn #1 (NSC 14613) | 213.24 | 91396-88-2 | Dehydrogenase | 43 | 201.6507222 | <1 |  | C12H11N3O |
| S1152 | PLX-4720 | 413.83 | 918505-84-7 | Raf | 83 | 200.5654496 | <1 |  | C17H14ClF2N3O3S |
| S3025 | PMSF | 174.19 | 329-98-6 | Others | 35 | 200.9300189 | <1 |  | C7H7FO2S |
| S7653 | *PND-1186 (VS-4718)* | *501.5* | 1061353-68-1 | FAK | 24 | 47.85643071 | <1 |  | C25H26F3N5O3 |
| S2629 | PNU-120596 | 311.72 | 501925-31-1 | AChR | 62 | 198.8964455 | <1 |  | C13H14ClN3O4 |
| S2390 | Polydatin | 390.38 | 65914-17-2 | Others | 78 | 199.8053179 | <1 |  | C20H22O8 |
| S1567 | Pomalidomide | 273.24 | 19171-19-8 | TNF-alpha, COX | 55 | 201.2882448 | <1 |  | C13H11N3O4 |
| S1490 | Ponatinib (AP24534) | 532.56 | 943319-70-8 | Bcr-Abl, VEGFR, FGFR, PDGFR, Flt | 30 | 56.33168094 | <1 |  | C29H27F3N6O |
| S1257 | Posaconazole | 700.78 | 171228-49-2 | Others | 100 | 142.6981364 | <1 |  | C37H42F2N8O4 |
| S1897 | Potassium Iodide | 166 | 7681-11-0 | Others | 33 | 198.7951807 | 33 | 198.7951807 | IK |
| S7358 | *Poziotinib (HM781-36B)* | *491.34* | 1092364-38-9 | EGFR | 98 | 199.4545529 | <1 |  | C23H21Cl2FN4O3 |
| S7060 | PP1 | 281.36 | 172889-26-8 | Src | 4 | 14.21666193 | <1 |  | C16H19N5 |
| S2622 | PP121 | 319.36 | 1092788-83-4 | DNA-PK, mTOR, PDGF | 64 | 200.4008016 | <1 |  | C17H17N7 |
| S7008 | PP2 | 301.77 | 172889-27-9 | Src | 60 | 198.8269212 | <1 |  | C15H16ClN5 |
| S2218 | PP242 | 308.34 | 1092351-67-1 | mTOR | 62 | 201.0767335 | <1 |  | C16H16N6O |
| S8003 | PQ 401 | 341.79 | 196868-63-0 | IGF-1R | 32 | 93.62474034 | <1 |  | C18H16ClN3O2 |
| S7130 | PR-619 | 223.28 | 2645-32-1 | DUB | 45 | 201.5406664 | <1 |  | C7H5N5S2 |
| S1515 | Pracinostat (SB939) | 358.48 | 929016-96-6 | HDAC | 72 | 200.848025 | <1 |  | C20H30N4O2 |
| S1497 | Pralatrexate | 477.47 | 146464-95-1 | DHFR | 28 | 58.6424278 | <1 |  | C23H23N7O5 |
| S2460 | Pramipexole | 211.33 | 104632-26-0 | Dopamine Receptor | 42 | 198.7413051 | <1 |  | C10H17N3S |
| S2011 | Pramipexole dihydrochloride monohydrate | 302.26 | 191217-81-9 | Others | 41 | 135.6448091 | 60 | 198.5045987 | C10H21Cl2N3OS |
| S2594 | Pramiracetam | 269.38 | 68497-62-1 | Others | 54 | 200.4603163 | <1 |  | C14H27N3O2 |
| S4092 | Pramoxine HCl | 329.86 | 637-58-1 | Others | 66 | 200.0848845 | 66 | 200.0848845 | C17H28ClNO3 |
| S1829 | Pranlukast | 481.5 | 103177-37-3 | Others | 11 | 22.84527518 | <1 |  | C27H23N5O4 |
| S1960 | Pranoprofen | 255.27 | 52549-17-4 | Others | 51 | 199.7884593 | <1 |  | C15H13NO3 |
| S1258 | Prasugrel | 373.44 | 150322-43-3 | P2 Receptor | 30 | 80.33419023 | <1 |  | C20H20FNO3S |
| S3036 | Pravastatin sodium | 446.51 | 81131-70-6 | HMG-CoA Reductase | 89 | 199.3236434 | 89 | 199.3236434 | C23H35NaO7 |
| S1691 | Praziquantel | 312.41 | 55268-74-1 | Others | 63 | 201.6580775 | <1 |  | C19H24N2O2 |
| S1737 | Prednisolone | 360.44 | 50-24-8 | Others | 72 | 199.755854 | <1 |  | C21H28O5 |
| S2570 | *Prednisolone Acetate* | *402.48* | 52-21-1 | Others | 81 | 201.2522361 | <1 |  | C23H30O6 |
| S1622 | Prednisone | 358.43 | 53-03-2 | Others | 71 | 198.0860977 | <1 |  | C21H26O5 |
| S1914 | Pregnenolone | 316.48 | 145-13-1 | Estrogen/progestogen Receptor | 22 | 69.51466127 | <1 |  | C21H32O2 |
| S1619 | Prilocaine | 220.31 | 721-50-6 | Others | 44 | 199.7185784 | <1 |  | C13H20N2O |
| S4237 | Primaquine Diphosphate | 455.34 | 63-45-6 | Others | 91 | 199.850661 | 91 | 199.850661 | C15H27N3O9P2 |
| S1965 | Primidone | 218.25 | 125-33-7 | Others | 44 | 201.6036655 | <1 |  | C12H14N2O2 |
| S7546 | Pritelivir (BAY 57-1293) | 402.49 | 348086-71-5 | Others | 80 | 198.7627022 | <1 |  | C18H18N4O3S2 |
| S4022 | Probenecid | 285.36 | 57-66-9 | Others | 57 | 199.7476871 | <1 |  | C13H19NO4S |
| S2119 | Probucol | 516.84 | 23288-49-5 | Others | 103 | 199.2879808 | <1 |  | C31H48O2S2 |
| S4294 | Procainamide HCl | 271.79 | 614-39-1 | Sodium Channel | 54 | 198.6828066 | 54 | 198.6828066 | C13H22ClN3O |
| S4023 | Procaine HCl | 272.77 | 51-05-8 | Sodium Channel | 55 | 201.6350772 | 55 | 201.6350772 | C13H21ClN2O2 |
| S1705 | Progesterone | 314.46 | 57-83-0 | Others | 22 | 69.96120333 | <1 |  | C21H30O2 |
| S4293 | Promethazine HCl | 320.88 | 58-33-3 | Histamine Receptor | 64 | 199.4515084 | 64 | 199.4515084 | C17H21ClN2S |
| S2500 | Propafenone HCl | 377.9 | 34183-22-7 | Sodium Channel | 69 | 182.5879862 | <1 |  | C21H27NO3 |
| S1828 | Proparacaine HCl | 330.85 | 5875-06-9 | Sodium Channel | 12 | 36.27021309 | 66 | 199.486172 | C16H27ClN2O3 |
| S4076 | Propranolol HCl | 295.8 | 318-98-9 | Adrenergic Receptor | 59 | 199.459094 | 6 | 20.28397566 | C16H22ClNO2 |
| S1988 | Propylthiouracil | 170.23 | 51-52-5 | Others | 34 | 199.7297774 | <1 |  | C7H10N2OS |
| S1881 | Protionamide | 180.27 | 14222-60-7 | Others | 36 | 199.7004493 | <1 |  | C9H12N2S |
| S8032 | PRT062607 (P505-15, BIIB057) HCl | 429.91 | 1370261-97-4,1370261-96-3(free base) | Syk | 86 | 200.0418692 | 86 | 200.0418692 | C19H24ClN9O |
| S2875 | Prucalopride | 367.87 | 179474-81-8 | 5-HT Receptor | 60 | 163.1010955 | <1 |  | C18H26ClN3O3 |
| S4247 | Prucalopride Succinat | 485.96 | 179474-85-2 | Others | 97 | 199.6049058 | 97 | 199.6049058 | C22H32ClN3O7 |
| S8010 | PRX-08066 Maleic acid | 517.96 | 866206-55-5 | 5-HT Receptor | 104 | 200.7877056 | 104 | 200.7877056 | C23H21ClFN5O4S |
| S7372 | PTC-209 | 495.19 | 315704-66-6 | Others | 99 | 199.9232618 | <1 |  | C17H13Br2N5OS |
| S7539 | PTC-209 HBr | 576.1 | 1217022-63-3 | Others | 100 | 173.5809755 | <1 |  | C17H14Br3N5OS |
| S2346 | Puerarin | 416.38 | 3681-99-0 | Others | 86 | 206.542101 | <1 |  | C21H20O10 |
| S8039 | PU-H71 | 512.37 | 873436-91-0 | HSP | 100 | 195.1714581 | 34 | 66.35829576 | C18H21IN6O2S |
| S7417 | Puromycin 2HCl | 544.43 | 58-58-2 | Others | 100 | 183.68 | 100 | 183.6783425 | C22H31Cl2N7O5 |
| S7793 | *Purvalanol A* | *388.89* | 212844-53-6 | CDK | 60 | 154.2852735 | <1 |  | C19H25ClN6O |
| S7612 | *PX-478 2HCl* | *394.12* | 685898-44-6 | HIF | 78 | 197.9092662 | 78 | 197.9092662 | C13H20Cl4N2O3 |
| S7129 | PYR-41 | 371.3 | 418805-02-4 | E1 Activating | 74 | 199.2997576 | <1 |  | C17H13N3O7 |
| S1762 | Pyrazinamide | 123.11 | 98-96-4 | Others | 25 | 203.0704248 | 12 | 97.47380392 | C5H5N3O |
| S1608 | Pyridostigmine Bromide | 261.12 | 101-26-8 | Others | 52 | 199.1421569 | 52 | 199.1421569 | C9H13BrN2O2 |
| S3113 | Pyridoxine HCl | 205.64 | 58-56-0 | Others | 41 | 199.377553 | 41 | 199.377553 | C8H12ClNO3 |
| S2006 | Pyrimethamine | 248.71 | 58-14-0 | NULL | 10 | 40.20747055 | <1 |  | C12H13ClN4 |
| S4902 | QNZ (EVP4593) | 356.42 | 545380-34-5 | NF-κB | 5 | 14.02839347 | <1 |  | C22H20N4O |
| S2391 | Quercetin | 302.24 | 117-39-5 | PI3K, PKC, Src, Sirtuin | 61 | 201.8263632 | <1 |  | C15H10O7 |
| S2347 | Quercetin Dihydrate | 338.27 | 6151-25-3 | Others | 68 | 201.0228516 | <1 |  | C15H14O9 |
| S1763 | Quetiapine Fumarate | 883.09 | 111974-72-2 | Dopamine Receptor | 36 | 40.76594685 | <1 |  | C46H54N6O8S2 |
| S4255 | Quinacrine 2HCl | 472.88 | 69-05-6 | Others | 14 | 29.60581966 | 53 | 112.0791744 | C23H32Cl3N3O |
| S2581 | Quinapril HCl | 474.98 | 82586-55-8 | RAAS | 18 | 37.89633248 | 95 | 200.0084214 | C25H31ClN2O5 |
| S2502 | Quinine HCl Dihydrate | 396.91 | 6119-47-7 | Others | 79 | 199.0375652 | 43 | 108.3369026 | C20H29ClN2O4 |
| S1096 | Quisinostat (JNJ-26481585) | 394.476 | 875320-31-3 | HDAC | 79 | 200.2656689 | <1 |  | C21H28Cl2N6O2 |
| S1526 | Quizartinib (AC220) | 560.67 | 950769-58-1 | Flt | 33.2 | 59.21486793 | <0.3 |  | C29H32N6O4S |
| S2194 | R406 | 628.63 | 841290-81-1 | Syk, Flt | 126 | 200.4358685 | <1 |  | C28H29FN6O8S |
| S1533 | R406 (free base) | 470.45 | 841290-80-0 | Syk | 21 | 44.63811245 | <1 |  | C22H23FN6O5 |
| S2688 | R547 | 441.45 | 741713-40-6 | CDK | 60 | 135.9157322 | <1 |  | C18H21F2N5O4S |
| S2503 | Racecadotril | 385.48 | 81110-73-8 | Opioid Receptor | 22 | 57.07170281 | <1 |  | C21H23NO4S |
| S2161 | RAF265 (CHIR-265) | 518.41 | 927880-90-8 | Raf, VEGFR | 100 | 192.8975136 | <1 |  | C24H16F6N6O |
| S1227 | Raloxifene HCl | 510.04 | 82640-04-8 | Estrogen/progestogen Receptor | 100 | 196.0630539 | <1 |  | C28H28ClNO4S |
| S2005 | Raltegravir (MK-0518) | 444.42 | 518048-05-0 | Integrase | 88 | 198.0108906 | <1 |  | C20H21FN6O5 |
| S1192 | Raltitrexed | 458.49 | 112887-68-0 | DNA/RNA Synthesis | 92 | 200.6586839 | <1 |  | C21H22N4O6S |
| S1259 | Ramelteon | 259.34 | 196597-26-9 | Others | 52 | 200.5089843 | <1 |  | C16H21NO2 |
| S1793 | Ramipril | 416.51 | 87333-19-5 | RAAS | 83 | 199.2749274 | <1 |  | C23H32N2O5 |
| S1801 | Ranitidine | 350.86 | 66357-59-3 | NULL | 70 | 199.509776 | 70 | 199.509776 | C13H23ClN4O3S |
| S1799 | Ranolazine | 427.54 | 95635-55-5 | Others | 86 | 201.1507695 | <1 |  | C24H33N3O4 |
| S1425 | Ranolazine 2HCl | 500.46 | 95635-56-6 | Calcium Channel | 100 | 199.8161691 | 100 | 199.8161691 | C24H35Cl2N3O4 |
| S1039 | Rapamycin (Sirolimus) | 914.18 | 53123-88-9 | mTOR | 20 | 21.87752959 | <1 |  | C51H79NO13 |
| S2102 | Rasagiline Mesylate | 267.34 | 161735-79-1 | MAO | 53 | 198.2494202 | 53 | 198.2494202 | C13H17NO3S |
| S7606 | *RBC8* | *424.45* | 361185-42-4 | Others | 84 | 197.9031688 | <1 |  | C25H20N4O3 |
| S2032 | Rebamipide | 370.79 | 90098-04-7 | Others | 74 | 199.5738828 | <1 |  | C19H15ClN2O4 |
| S3199 | Reboxetine mesylate | 409.5 | 98769-84-7 | Others | 82 | 200.2442002 | 82 | 200.2442002 | C20H27NO6S |
| S1089 | Refametinib (RDEA119, Bay 86-9766) | 572.34 | 923032-37-5 | MEK | 100 | 174.7213195 | <1 |  | C19H20F3IN2O5S |
| S1178 | Regorafenib (BAY 73-4506) | 482.82 | 755037-03-7 | c-Kit, Raf, VEGFR | 97 | 200.903028 | <1 |  | C21H15ClF4N4O3 |
| S7641 | *Remodelin* | *363.28* | 1622921-15-6 | Histone Acetyltransferase | 72 | 198.1942303 | <1 |  | C15H15BrN4S |
| S1426 | Repaglinide | 452.59 | 135062-02-1 | Potassium Channel | 91 | 201.0649816 | <1 |  | C27H36N2O4 |
| S7223 | RepSox | 287.32 | 446859-33-2 | TGF-beta/Smad | 57 | 198.3850759 | <1 |  | C17H13N5 |
| S1601 | Reserpine | 608.68 | 50-55-5 | Others | 13 | 21.35769205 | <1 |  | C33H40N2O9 |
| S2693 | Resminostat | 349.4 | 864814-88-0 | HDAC | 70 | 200.3434459 | <1 |  | C16H19N3O4S |
| S1396 | Resveratrol | 228.24 | 501-36-0 | Sirtuin | 45 | 197.1608833 | <1 |  | C14H12O3 |
| S4056 | Retapamulin | 517.76 | 224452-66-8 | Others | 104 | 200.8652658 | <1 |  | C30H47NO4S |
| S2821 | RG108 | 334.33 | 48208-26-0 | Transferases | 67 | 200.4008016 | <1 |  | C19H14N2O4 |
| S7292 | RG2833 (RGFP109) | 339.43 | 1215493-56-3 | HDAC | 68 | 200.3358572 | <1 |  | C20H25N3O2 |
| S7229 | RGFP966 | 362.4 | 1396841-57-8 | HDAC | 72 | 198.6754967 | <1 |  | C21H19FN4O |
| S8077 | RI-1 | 361.61 | 415713-60-9 | Others | 50 | 138.2705124 | <1 |  | C14H11Cl3N2O3 |
| S2504 | Ribavirin | 244.20864 | 36791-04-5 | Others | 49 | 200.6481016 | 49 | 200.6481016 | C8H12N4O5 |
| S2612 | Ribitol | 152.15 | 488-81-3 | Others | 31 | 203.75 | 31 | 203.746303 | C5H12O5 |
| S1022 | Ridaforolimus (Deforolimus, MK-8669) | 990.21 | 572924-54-0 | mTOR | 198 | 199.96 | <1 |  | C53H84NO14P |
| S1741 | Rifabutin | 847 | 72559-06-9 | Others | 60 | 70.83825266 | <1 |  | C46H62N4O11 |
| S1764 | Rifampin | 822.94 | 13292-46-1 | Others | 100 | 121.5155418 | <1 |  | C43H58N4O12 |
| S1760 | Rifapentine | 877.03 | 61379-65-5 | Others | 100 | 114.0211851 | <1 |  | C47H64N4O12 |
| S1790 | Rifaximin | 785.88 | 80621-81-4 | Others | 47 | 59.80556828 | <1 |  | C43H51N3O11 |
| S1362 | Rigosertib (ON-01910) | 473.47 | 1225497-78-8 | PLK | 95 | 200.6462923 | 95 | 200.6462923 | C21H24NNaO8S |
| S7303 | Rilpivirine | 366.42 | 500287-72-9 | Reverse Transcriptase | 73 | 199.2249331 | <1 |  | C22H18N6 |
| S1614 | Riluzole | 234.2 | 1744-22-5 | Sodium Channel | 47 | 200.6831768 | <1 |  | C8H5F3N2OS |
| S1964 | Rimantadine | 179.3 | 13392-28-4 | Others | 33 | 184.0490798 | 33 | 184.0490798 | C12H21N |
| S3021 | Rimonabant | 463.79 | 168273-06-1 | Cannabinoid Receptor | 25 | 53.90370642 | <1 |  | C22H21Cl3N4O |
| S1615 | Risperidone | 410.48 | 106266-06-2 | 5-HT Receptor |  | 10 |  |  | C23H27FN4O2 |
| S2781 | RITA (NSC 652287) | 292.37 | 213261-59-7 | p53 | 58 | 198.3787666 | <1 |  | C14H12O3S2 |
| S2533 | Ritodrine HCl | 323.81 | 23239-51-2 | Adrenergic Receptor | 65 | 200.7349989 | 65 | 200.7349989 | C17H22ClNO3 |
| S1185 | Ritonavir | 720.94 | 155213-67-5 | HIV Protease | 100 | 138.7077982 | <1 |  | C37H48N6O5S2 |
| S3002 | Rivaroxaban | 435.88 | 366789-02-8 | Factor Xa | 87 | 199.5962191 | <1 |  | C19H18ClN3O5S |
| S2087 | Rivastigmine Tartrate | 400.42 | 129101-54-8 | AChR | 42 | 104.8898656 | 80 | 199.7902203 | C18H28N2O8 |
| S1607 | Rizatriptan Benzoate | 391.47 | 145202-66-0 | NULL | 20 | 51.08948323 | 46 | 117.5058114 | C22H25N5O2 |
| S7195 | RKI-1447 | 326.37 | 1342278-01-6 | ROCK | 65 | 199.1604621 | <1 |  | C16H14N4O2S |
| S7080 | *RN486* | *606.69* | 1242156-23-5 | BTK | 62 | 102.1938717 | <1 |  | C35H35FN6O3 |
| S7207 | Ro 31-8220 Mesylate | 553.65 | 138489-18-6 | PKC | 100 | 180.62 | <1 |  | C26H27N5O5S2 |
| S7248 | Ro3280 | 543.61 | 1062243-51-9 | PLK | 100 | 183.9554092 | <1 |  | C27H35F2N7O3 |
| S7747 | *Ro-3306* | *351.45* | 872573-93-8 | CDK | 13 | 36.98961445 | <1 |  | C18H13N3OS2 |
| S1575 | RO4929097 | 469.4 | 847925-91-1 | Y-Secretase | 94 | 200.2556455 | <1 |  | C22H20F5N3O3 |
| S8001 | Rocilinostat (ACY-1215) | 433.5 | 1316214-52-4 | HDAC | 86 | 198.3852364 | <1 |  | C24H27N5O3 |
| S1397 | Rocuronium Bromide | 609.68 | 119302-91-9 | AChR | 122 | 200.1049731 | 122 | 200.1049731 | C32H53BrN2O4 |
| S3043 | Rofecoxib | 314.36 | 162011-90-7 | COX | 63 | 200.4071765 | <1 |  | C17H14O4S |
| S2131 | Roflumilast | 403.21 | 162401-32-3 | PDE | 81 | 200.8878748 | <1 |  | C17H14Cl2F2N2O3 |
| S1430 | Rolipram | 275.34 | 61413-54-5 | PDE | 55 | 199.7530326 | <1 |  | C16H21NO3 |
| S4062 | Ronidazole | 200.15 | 7681-76-7 | Others | 40 | 199.8501124 | <1 |  | C6H8N4O4 |
| S3189 | Ropinirole HCl | 296.84 | 91374-20-8 | Others | 10 | 33.68818219 | 60 | 202.1290931 | C16H25ClN2O |
| S4058 | Ropivacaine HCl | 310.86 | 98717-15-8 | Others | 62 | 199.4466963 | 46 | 147.9765811 | C17H27ClN2O |
| S1153 | Roscovitine (Seliciclib,CYC202) | 354.45 | 186692-46-6 | CDK | 71 | 200.31034 | <1 |  | C19H26N6O |
| S2556 | Rosiglitazone | 357.43 | 122320-73-4 | PPAR | 71 | 198.6402932 | <1 |  | C18H19N3O3S |
| S2075 | *Rosiglitazone HCl* | *393.89* | 302543-62-0 | PPAR | 79 | 200.5636091 | <1 |  | C18H20ClN3O3S |
| S2505 | *Rosiglitazone maleate* | *473.5* | 155141-29-0 | PPAR | 94 | 198.5216473 | <1 |  | C22H23N3O7S |
| S2169 | Rosuvastatin Calcium | 500.57 | 147098-20-2 | HMG-CoA Reductase | 100 | 199.7722596 | <1 |  | C44H54CaF2N6O12S2 |
| S4274 | Rotigotine | 315.47 | 99755-59-6 | Dopamine Receptor | 63 | 199.7020319 | <1 |  | C19H25NOS |
| S2437 | Rotundine | 355.43 | 483-14-7 | Dopamine Receptor | 8 | 22.50794812 | <1 |  | C21H25NO4 |
| S1880 | Roxatidine Acetate HCl | 384.9 | 93793-83-0 | Histamine Receptor | 77 | 200.0519615 | 77 | 200.0519615 | C19H29ClN2O4 |
| S2506 | Roxithromycin | 837.06727 | 80214-83-1 | Others | 167 | 199.5060684 | <1 |  | C41H76N2O15 |
| S2698 | RS-127445 | 281.33 | 199864-87-3 | 5-HT Receptor | 56 | 199.0544912 | <1 |  | C17H16FN3 |
| S1098 | Rucaparib (AG-014699,PF-01367338) | 421.36 | 459868-92-9 | PARP | 84 | 199.3544712 | <1 |  | C19H21FN3O5P |
| S1256 | Rufinamide | 238.19 | 106308-44-5 | Sodium Channel | 47 | 197.3214661 | <1 |  | C10H8F2N4O |
| S3052 | Rupatadine Fumarate | 532.03 | 182349-12-8 | Histamine Receptor | 9 | 16.9163393 | <1 |  | C30H30ClN3O4 |
| S2349 | Rutaecarpine | 287.32 | 84-26-4 | Others | 24 | 83.53055826 | <1 |  | C18H13N3O |
| S2350 | Rutin | 610.52 | 153-18-4 | NULL | 122 | 199.8296534 | <1 |  | C27H30O16 |
| S1378 | Ruxolitinib (INCB018424) | 306.37 | 941678-49-5 | JAK | 61 | 199.1056566 | <1 |  | C17H18N6 |
| S7295 | RVX-208 | 370.4 | 1044870-39-4 | Epigenetic Reader Domain | 74 | 199.7840173 | <1 |  | C20H22N2O5 |
| S2127 | S- (+)-Rolipram | 275.34 | 85416-73-5 | PDE | 55 | 199.7530326 | <1 |  | C16H21NO3 |
| S1155 | S3I-201 | 365.36 | 501919-59-1 | STAT | 73 | 199.8029341 | <1 |  | C16H15NO7S |
| S1472 | Safinamide Mesylate | 398.45 | 202825-46-5 | MAO | 80 | 200.7780148 | 80 | 200.7780148 | C18H23FN2O5S |
| S7437 | Sal003 | 463.21 | 1164470-53-4 | Others | 93 | 200.7728676 | <1 |  | C18H15Cl4N3OS |
| S2351 | Salicin | 286.28 | 138-52-3 | Others | 57 | 199.1057706 | 24 | 83.83400866 | C13H18O7 |
| S4187 | Salicylanilide | 213.23 | 87-17-2 | Others | 43 | 201.6601791 | <1 |  | C13H11NO2 |
| S2396 | Salidroside | 300.3 | 10338-51-9 | Others | 60 | 199.8001998 | 60 | 199.8001998 | C14H20O7 |
| S4296 | *Salmeterol Xinafoate* | *603.75* | 94749-08-3 | Adrenergic Receptor | 100 | 165.63147 | <1 |  | C36H45NO7 |
| S2923 | Salubrinal | 479.81 | 137975-06-5 | Others | 96 | 200.079198 | <1 |  | C21H17Cl3N4OS |
| S7092 | SANT-1 | 373.49 | 304909-07-7 | Smoothened | 21 | 56.22640499 | <1 |  | C23H27N5 |
| S7595 | Santacruzamate A (CAY10683) | 278.35 | 1477949-42-0 | HDAC | 55 | 197.5929585 | <1 |  | C15H22N2O3 |
| S2842 | SAR131675 | 358.39 | 1433953-83-3 | VEGFR | 30 | 83.71 | <1 |  | C18H22N4O4 |
| S1523 | SAR245409 (XL765) | 599.66 | 1349796-36-6 | PI3K, mTOR | 12 | 20.01133976 | <1 |  | C31H29N5O6S |
| S1006 | Saracatinib (AZD0530) | 542.03 | 379231-04-6 | Src, Bcr-Abl | 35 | 64.57207166 | <1 |  | C27H32ClN5O5 |
| S4188 | Sasapyrine | 258.23 | 552-94-3 | Others | 52 | 201.3708709 | <1 |  | C14H10O5 |
| S1540 | Saxagliptin | 315.41 | 361442-04-8 | DPP-4 | 63 | 199.7400209 | 63 | 199.7400209 | C18H25N3O2 |
| S1077 | SB202190 (FHPI) | 331.34 | 152121-30-7 | p38 MAPK | 66 | 199.1911632 | <1 |  | C20H14FN3O |
| S1076 | SB203580 | 377.43 | 152121-47-6 | p38 MAPK | 43 | 113.9284106 | <1 |  | C21H16FN3OS |
| S1075 | SB216763 | 371.22 | 280744-09-4 | GSK-3 | 23 | 61.95786865 | <1 |  | C19H12Cl2N2O2 |
| S7651 | *SB225002* | *352.14* | 182498-32-4 | CXCR | 70 | 198.7845743 | <1 |  | C13H10BrN3O4 |
| S2849 | SB269970 HCl | 388.95 | 261901-57-9 | 5-HT Receptor | 11 | 28.28127009 | <1 |  | C18H29ClN2O3S |
| S2856 | SB271046 | 451.99 | 209481-20-9 | 5-HT Receptor | 40 | 88.49753313 | <1 |  | C20H22ClN3O3S2 |
| S7540 | *SB273005* | *451.44* | 205678-31-5 | Integrin | 90 | 199.3620415 | <1 |  | C22H24F3N3O4 |
| S7585 | *SB-334867* | *319.32* | 792173-99-0 | OX Receptor | 63 | 197.2942503 | <1 |  | C17H13N5O2 |
| S7430 | SB-3CT | 306.4 | 292605-14-2 | MMP | 61 | 199.0861619 | <1 |  | C15H14O3S2 |
| S1545 | SB408124 | 356.37 | 288150-92-5 | Others | 36 | 101.0186043 | <1 |  | C19H18F2N4O |
| S2729 | SB415286 | 359.72 | 264218-23-7 | GSK-3 | 72 | 200.1556766 | <1 |  | C16H10ClN3O5 |
| S1067 | SB431542 | 384.39 | 301836-41-9 | TGF-beta/Smad | 77 | 200.317386 | <1 |  | C22H16N4O3 |
| S2186 | SB505124 | 335.4 | 694433-59-5 | TGF-beta/Smad | 67 | 199.7614788 | <1 |  | C20H21N3O2 |
| S1476 | SB525334 | 343.42 | 356559-20-1 | TGF-beta/Smad | 68 | 198.0082698 | <1 |  | C21H21N5 |
| S2220 | SB590885 | 453.54 | 405554-55-4 | Raf | 5 | 11.02438594 | <1 |  | C27H27N5O2 |
| S2773 | SB705498 | 429.23 | 501951-42-4 | TRPV | 86 | 200.358782 | <1 |  | C17H16BrF3N4O |
| S2894 | SB742457 | 353.44 | 607742-69-8 | 5-HT Receptor | 78 | 220.6880942 | <1 |  | C19H19N3O2S |
| S2182 | SB743921 | 553.52 | 940929-33-9 | Kinesin | 111 | 200.53 | 22 | 39.74562798 | C31H34Cl2N2O3 |
| S7720 | *SBE 13 HCl* | *479.4* | 1052532-15-6 | PLK | 95 | 198.1643721 | <1 |  | C24H28Cl2N2O4 |
| S7124 | SC144 | 322.3 | 895158-95-9 | Others | 28 | 86.87558176 | <1 |  | C16H11FN6O |
| S4907 | SC-514 | 224.3 | 354812-17-2 | IκB/IKK | 45 | 200.6241641 | <1 |  | C9H8N2OS2 |
| S7273 | SC75741 | 565.67 | 913822-46-5 | NF-κB | 35 | 61.8735305 | <1 |  | C29H23N7O2S2 |
| S7863 | *SC79* | *364.78* | 305834-79-1 | Akt | 72 | 197.3792423 | <1 |  | C17H17ClN2O5 |
| S3600 | Schisandrin B (Sch B) | 400.46 | 61281-37-6 | ATM/ATR | 80 | 199.77 | <1 |  | C23H28O6 |
| S2354 | Sclareol | 308.5 | 515-03-7 | Others | 61 | 197.7309562 | <1 |  | C20H36O2 |
| S2355 | Sclareolide | 250.38 | 564-20-5 | Others | 50 | 199.6964614 | <1 |  | C16H26O2 |
| S2545 | Scopine | 155.19 | 498-45-3 | Others | 31 | 199.7551389 | <1 |  | C8H13NO2 |
| S2508 | Scopolamine HBr | 384.26 | 114-49-8 | Others | 76 | 197.7827513 | 76 | 197.7827513 | C17H22BrNO4 |
| S7742 | *SCR7* | *334.39* | 1533426-72-0 | DNA/RNA Synthesis | 66 | 197.3743234 | <1 |  | C18H14N4OS |
| S8043 | Scriptaid | 326.35 | 287383-59-9 | HDAC | 65 | 199.1726674 | <1 |  | C18H18N2O4 |
| S7624 | *SD-208* | *352.75* | 627536-09-8 | TGF-beta/Smad | 9 | 25.51381999 | <1 |  | C17H10ClFN6 |
| S7685 | *SecinH3* | *460.5* | 853625-60-2 | Others | 92 | 199.7828447 | <1 |  | C24H20N4O4S |
| S2537 | Secnidazole | 185.18 | 3366-95-8 | Others | 37 | 199.8055946 | 37 | 199.8055946 | C7H11N3O3 |
| S1008 | Selumetinib (AZD6244) | 457.68 | 606143-52-6 | MEK | 91 | 198.8288761 | <1 |  | C17H15BrClFN4O3 |
| S1594 | Semagacestat (LY450139) | 361.44 | 425386-60-3 | Gamma-secretase | 72 | 199.2031873 | <1 |  | C19H27N3O4 |
| S2845 | Semaxanib (SU5416) | 238.28 | 194413-58-6 | VEGFR | 22 | 92.3283532 | <1 |  | C15H14N2O |
| S4244 | Serotonin HCl | 212.68 | 153-98-0 | Others | 43 | 202.1816814 | 43 | 202.1816814 | C10H13ClN2O |
| S3161 | Sertaconazole nitrate | 500.78 | 99592-39-9 | Others | 100 | 199.688486 | <1 |  | C20H16Cl3N3O4S |
| S4053 | Sertraline HCl | 342.69 | 79559-97-0 | 5-HT Receptor | 69 | 201.3481572 | <1 |  | C17H18Cl3N |
| S2392 | Sesamin | 354.35 | 607-80-7 | Others | 70 | 197.5448003 | <1 |  | C20H18O6 |
| S7310 | SF1670 | 307.34 | 345630-40-2 | Others | 29 | 94.35803996 | <1 |  | C19H17NO3 |
| S7079 | SGC 0946 | 618.57 | 1561178-17-3 | Histone Methyltransferase | 100 | 161.6631909 | <1 |  | C28H40BrN7O4 |
| S7256 | SGC-CBP30 | 509.04 | 1613695-14-9 | Epigenetic Reader Domain | 100 | 196.4482163 | <1 |  | C28H33ClN4O3 |
| S7276 | SGI-1027 | 461.52 | 1020149-73-8 | DNA Methyltransferase | 92 | 199.341307 | <1 |  | C27H23N7O |
| S2198 | SGI-1776 free base | 405.42 | 1025065-69-3 | Pim | 81 | 199.7928075 | <1 |  | C20H22F3N5O |
| S1112 | SGX-523 | 359.41 | 1022150-57-7 | c-Met |  | 10 |  |  | C18H13N7S |
| S7337 | SH-4-54 | 610.59 | 1456632-40-8 | STAT | 100 | 163.7760199 | <1 |  | C29H27F5N2O5S |
| S2356 | Shikimic Acid | 174.15 | 138-59-0 | Others | 35 | 200.97617 | 35 | 200.97617 | C7H10O5 |
| S1431 | Sildenafil Citrate | 666.7 | 171599-83-0 | PDE | 20 | 29.99850007 | <1 |  | C28H38N6O11S |
| S2357 | Silibinin | 482.44 | 22888-70-6 | Others | 96 | 198.9884753 | <1 |  | C25H22O10 |
| S1613 | Silodosin | 495.53 | 160970-54-7 | Adrenergic Recepto | 99 | 199.7860876 | <1 |  | C25H32F3N3O4 |
| S2358 | Silymarin | 482.44 | 65666-07-1 | NULL | 96 | 198.9884753 | <1 |  | C25H22O10 |
| S1792 | Simvastatin | 418.57 | 79902-63-9 | Others | 83 | 198.2941921 | <1 |  | C25H38O5 |
| S2359 | Sinomenine | 329.39 | 115-53-7 | Others | 66 | 200.3703816 | 66 | 200.3703816 | C19H23NO4 |
| S2804 | Sirtinol | 394.47 | 410536-97-9 | Sirtuin | 23 | 58.30608158 | <1 |  | C26H22N2O2 |
| S4002 | Sitagliptin phosphate monohydrate | 523.32 | 654671-77-9 | DPP-4 | 100 | 191.087671 | 41 | 78.34594512 | C16H20F6N5O6P |
| S3034 | Sitaxentan sodium | 476.89 | 210421-74-2 | Endothelin Receptor | 40 | 83.88 | <1 |  | C18H14ClN2NaO6S2 |
| S7214 | Skepinone-L | 425.42 | 1221485-83-1 | p38 MAPK | 85 | 199.8025481 | <1 |  | C24H21F2NO4 |
| S7176 | SKI II | 302.78 | 312636-16-1 | S1P Receptor | 61 | 201.4664113 | <1 |  | C15H11ClN2OS |
| S7258 | SKLB1002 | 320.39 | 1225451-84-2 | VEGFR | 7 | 21.8483723 | <1 |  | C13H12N4O2S2 |
| S1066 | SL-327 | 335.35 | 305350-87-2 | MEK | 4 | 11.92783659 | <1 |  | C16H12F3N3S |
| S8005 | SMI-4a | 273.23 | 438190-29-5 | Pim | 55 | 201.2956118 | <1 |  | C11H6F3NO2S |
| S7779 | *Smoothened Agonist (SAG) HCl* | *526.52* | 912545-86-9 | Hedgehog/Smoothened | 71 | 134.8476791 | 100 | 189.9263086 | C28H29Cl2N3OS |
| S4908 | SN-38 | 392.4 | 86639-52-3 | Topoisomerase | 21 | 53.51681957 | <1 |  | C22H20N2O5 |
| S1145 | SNS-032 (BMS-387032) | 380.53 | 345627-80-7 | CDK | 76 | 199.7214411 | <1 |  | C17H24N4O2S2 |
| S1154 | SNS-314 Mesylate | 527.04 | 1146618-41-8 | Aurora Kinase | 105 | 199.2258652 | 6 | 11.38433515 | C19H19ClN6O4S3 |
| S2639 | SNX-2112 (PF-04928473) | 464.48 | 908112-43-6 | HSP | 93 | 200.2239063 | <1 |  | C23H27F3N4O3 |
| S4073 | Sodium 4-Aminosalicylate | 211.15 | 133-10-8 | NF-κB | 42 | 198.910727 | 42 | 198.910727 | C7H6NNaO3 |
| S4245 | Sodium ascorbate | 201.13 | 134-03-2 | Others | 2 | 9.943817432 | 40 | 198.8763486 | C6H10NaO6 |
| S2401 | Sodium Danshensu | 220.15 | 67920-52-9 | P450 | 7 | 31.79650238 | 2 | 9.084714967 | C9H9NaO5 |
| S4074 | Sodium Nitrite | 69 | 7632-00-0 | Others | 13 | 188.4057971 | 13 | 188.4057971 | NNaO2 |
| S4059 | Sodium Nitroprusside | 261.92 | 14402-89-2 | Others | 60 | 229.0775809 | 60 | 229.0775809 | C5FeN6Na2O |
| S4125 | Sodium Phenylbutyrate | 186.18 | 1716-12-7 | HDAC | 8 | 42.96916962 | 30 | 161.1343861 | C10H11NaO2 |
| S4020 | Sodium Picosulfate | 481.41 | 10040-45-6 | Others | 96 | 199.4142207 | 96 | 199.4142207 | C18H13NNa2O8S2 |
| S3137 | Sodium salicylate | 161.11 | 54-21-7 | Others | 32 | 198.6220595 | 32 | 198.6220595 | C7H6NaO3 |
| S7896 | *Sodium Tauroursodeoxycholate (TUDC)* | *521.69* | 35807-85-3 | Others | 100 | 191.684717 | 100 | 191.684717 | C26H44NNaO6S |
| S2794 | Sofosbuvir (PSI-7977, GS-7977) | 529.45 | 1190307-88-0 | DNA/RNA Synthesis | 100 | 188.8752479 | 11 | 20.77627727 | C22H29FN3O9P |
| S3048 | Solifenacin succinate | 480.55 | 242478-38-2 | AChR | 3 | 6.242846738 | 96 | 199.7710956 | C27H32N2O6 |
| S2405 | Sophocarpine | 246.35 | 6483-15-4 | Others | 49 | 198.90 | 49 | 198.9039984 | C15H22N2O |
| S7397 | Sorafenib | 464.82 | 284461-73-0 | Raf | 63 | 135.5363366 | <1 |  | C21H16ClF3N4O3 |
| S1040 | Sorafenib Tosylate | 637.03 | 475207-59-1 | VEGFR, PDGFR, Raf | 127 | 199.3626674 | 0.01 | 0.015697848 | C28H24ClF3N4O6S |
| S2393 | Sorbitol | 182.17 | 50-70-4 | Others | 36 | 197.6176099 | 36 | 197.6176099 | C6H14O6 |
| S2509 | Sotalol | 308.82 | 959-24-0 | Adrenergic Receptor | 62 | 200.7641992 | 62 | 200.7641992 | C12H21ClN2O3S |
| S2791 | Sotrastaurin | 438.48 | 425637-18-9 | PKC | 87 | 198.4126984 | <1 |  | C25H22N6O2 |
| S7680 | *SP2509* | *437.90* | 1423715-09-6 | Histone Demethylase | 38 | 86.77780315 | <1 |  | C19H20ClN3O5S |
| S1460 | SP600125 | 220.23 | 129-56-6 | JNK | 44 | 199.7911275 | <1 |  | C14H8N2O |
| S1884 | Sparfloxacin | 392.4 | 110871-86-8 | Others | 9 | 22.93577982 | <1 |  | C19H22F2N4O3 |
| S2510 | Spectinomycin HCl | 405.27 | 21736-83-4 | Others | 81 | 199.8667555 | 81 | 199.8667555 | C14H26Cl2N2O7 |
| S4082 | Spiramycin | 843.058 | 8025-81-8 | Others | 100 | 118.615801 | <1 |  | C43H74N2O14 |
| S4054 | Spironolactone | 416.57 | 52-01-7 | Androgen Receptor | 83 | 199.2462251 | <1 |  | C24H32O4S |
| S7593 | *Splitomicin* | *198.22* | 5690-03-9 | Sirtuin | 39 | 196.7510847 | <1 |  | C13H10O2 |
| S7270 | SRPIN340 | 349.35 | 218156-96-8 | Others | 70 | 200.3721197 | <1 |  | C18H18F3N3O |
| S1129 | SRT1720 | 506.02 | 1001645-58-4 | Sirtuin | 38 | 75.09584601 | <1 |  | C25H24ClN7OS |
| S7792 | *SRT2104 (GSK2245840)* | *516.64* | 1093403-33-8 | Sirtuin | 16 | 30.96934035 | <1 |  | C26H24N6O2S2 |
| S2902 | S-Ruxolitinib (INCB018424) | 306.37 | 941685-37-6 | JAK | 61 | 199.1056566 | 5 | 16.32013578 | C17H18N6 |
| S7167 | SSR128129E | 346.31 | 848318-25-2 | FGFR | 69 | 199.2434524 | 1 | 2.887586267 | C18H15N2NaO4 |
| S7024 | Stattic | 211.19 | 19983-44-9 | STAT | 42 | 198.8730527 | <1 |  | C8H5NO4S |
| S1398 | Stavudine (d4T) | 224.21 | 3056-17-5 | Reverse Transcriptase | 45 | 200.7046965 | <1 |  | C10H12N2O4 |
| S2858 | StemRegenin 1 (SR1) | 429.54 | 1227633-49-9 | Others | 86 | 200.2141826 | <1 |  | C24H23N5OS |
| S7771 | *STF-083010* | *317.38* | 307543-71-1 | Others | 63 | 198.5002206 | <1 |  | C15H11NO3S2 |
| S7316 | STF-118804 | 461.53 | 894187-61-2 | Others | 61 | 132.1690898 | <1 |  | C25H23N3O4S |
| S1041 | STF-62247 | 267.35 | 315702-99-9 | Others | 53 | 198.24 | <1 |  | C15H13N3S |
| S1312 | Streptozotocin (STZ) | 265.22 | 18883-66-4 | Others | 53 | 199.8341 | 53 | 199.8341 | C8H15N3O7 |
| S1080 | SU11274 | 568.09 | 658084-23-2 | c-Met | 92 | 161.9461705 | <1 |  | C28H30ClN5O4S |
| S7774 | *SU6656* | *371.45* | 330161-87-0 | Src | 74 | 199.2192758 | <1 |  | C19H21N3O3S |
| S7636 | *SU9516* | *241.25* | 377090-84-1 | CDK | 48 | 198.9637306 | <1 |  | C13H11N3O2 |
| S4214 | Sucralose | 397.63 | 56038-13-2 | Others | 80 | 201.192063 | 80 | 201.192063 | C12H19Cl3O8 |
| S1958 | Sulbactam | 233.24 | 68373-14-8 | Others | 47 | 201.5091751 | 47 | 201.5091751 | C8H11NO5S |
| S2551 | Sulbactam sodium | 255.22 | 69388-84-7 | Others | 3 | 11.75456469 | 51 | 199.8275997 | C8H10NNaO5S |
| S4120 | Sulconazole Nitrate | 460.76 | 82382-23-8 | Others | 92 | 199.6701103 | <1 |  | C18H16Cl3N3O3S |
| S4081 | Sulfacetamide Sodium | 236.22 | 127-56-0 | Autophagy | 19 | 80.4334942 | 51 | 215.9004318 | C8H9N2NaO3S |
| S1770 | Sulfadiazine | 250.28 | 68-35-9 | Others | 50 | 199.7762506 | <1 |  | C10H10N4O2S |
| S4175 | Sulfaguanidine | 214.24 | 57-67-0 | Others | 43 | 200.7094847 | <1 |  | C7H10N4O2S |
| S3132 | Sulfamerazine | 264.3 | 127-79-7 | Others | 53 | 200.5297011 | <1 |  | C11H12N4O2S |
| S1618 | Sulfameter | 280.3 | 651-06-9 | Others | 56 | 199.7859436 | <1 |  | C11H12N4O3S |
| S3133 | Sulfamethazine | 278.33 | 57-68-1 | Others | 56 | 201.2000144 | <1 |  | C12H14N4O2S |
| S1957 | Sulfamethizole | 270.33 | 144-82-1 | Others | 54 | 199.755854 | <1 |  | C9H10N4O2S2 |
| S1915 | Sulfamethoxazole | 253.28 | 723-46-6 | Others | 51 | 201.3581807 | <1 |  | C10H11N3O3S |
| S4250 | Sulfamethoxypyridazine | 280.3 | 80-35-3 | Others | 56 | 199.7859436 | <1 |  | C11H12N4O3S |
| S1685 | Sulfanilamide | 172.2 | 63-74-1 | Others | 34 | 197.4448316 | <1 |  | C6H8N2O2S |
| S1576 | Sulfasalazine | 398.39 | 599-79-1 | Others | 80 | 200.8082532 | <1 |  | C18H14N4O5S |
| S3116 | Sulfathiazole | 255.32 | 72-14-0 | Others | 51 | 199.7493342 | <1 |  | C9H9N3O2S2 |
| S1916 | Sulfisoxazole | 267.3 | 127-69-5 | Others | 53 | 198.2790872 | <1 |  | C11H13N3O3S |
| S2007 | Sulindac | 356.41 | 38194-50-2 | NULL | 71 | 199.2087764 | <1 |  | C20H17FO3S |
| S1962 | Sulphadimethoxine | 310.33 | 122-11-2 | Others | 62 | 199.7873232 | <1 |  | C12H14N4O4S |
| S1432 | Sumatriptan Succinate | 413.49 | 103628-48-4 | 5-HT Receptor | 83 | 200.7303683 | 83 | 200.7303683 | C18H27N3O6S |
| S7781 | *Sunitinib* | *398.47* | 557795-19-4 | PDGFR | 25 | 62.73998043 | <1 |  | C22H27FN4O2 |
| S1042 | Sunitinib Malate | 532.56 | 341031-54-7 | VEGFR, PDGFR, c-Kit, Flt | 15 | 28.16584047 | <1 |  | C26H33FN4O7 |
| S2015 | Suplatast Tosylate | 499.64 | 94055-76-2 | Others | 100 | 200.1441038 | 100 | 200.1441038 | C23H33NO7S2 |
| S1761 | Suprofen | 260.31 | 40828-46-4 | Others | 52 | 199.7618224 | <1 |  | C14H12O3S |
| S7279 | Suvorexant (MK-4305) | 450.92 | 1030377-33-3 | OX Receptor | 10 | 22.17688282 | <1 |  | C23H23ClN6O2 |
| S2362 | Synephrine | 167.21 | 94-07-5 | Androgen Receptor | 33 | 197.3566174 | <1 |  | C9H13NO2 |
| S2438 | Synephrine HCl | 203.67 | 5985-28-4 | Others | 14 | 68.73864585 | 41 | 201.3060343 | C9H14ClNO2 |
| S2871 | T0070907 | 277.66 | 313516-66-4 | PPAR | 26 | 93.63970323 | <1 |  | C12H8ClN3O3 |
| S7076 | T0901317 | 481.33 | 293754-55-9 | Liver X Receptor | 96 | 199.4473646 | <1 |  | C17H12F9NO3S |
| S5003 | Tacrolimus (FK506) | 804.02 | 104987-11-3 | Others | 94 | 116.9125146 | <1 |  | C44H69NO12 |
| S1512 | Tadalafil | 389.4 | 171596-29-5 | PDE | 78 | 200.3081664 | <1 |  | C22H19N3O4 |
| S2820 | TAE226 (NVP-TAE226) | 468.94 | 761437-28-9 | FAK | 94 | 200.4520834 | <1 |  | C23H25ClN6O3 |
| S7495 | *TAI-1* | *431.51* | 1334921-03-7 | Microtubule Associated | 86 | 199.3001321 | <1 |  | C24H21N3O3S |
| S2784 | TAK-285 | 547.96 | 871026-44-7 | EGFR, HER2 | 110 | 200.7445799 | <1 |  | C26H25ClF3N5O3 |
| S8016 | TAK-438 | 461.46 | 1260141-27-2 | Potassium Channel | 62 | 134.3561739 | <1 |  | C21H20FN3O6S |
| S7291 | TAK-632 | 554.52 | 1228591-30-7 | Raf | 100 | 180.3361466 | <1 |  | C27H18F4N4O3S |
| S1195 | TAK-700 (Orteronel) | 307.35 | 566939-85-3 | P450 | 61 | 198.4707988 | <1 |  | C18H17N3O2 |
| S2928 | TAK-715 | 399.51 | 303162-79-0 | p38 MAPK | 80 | 200.2453005 | <1 |  | C24H21N3OS |
| S2617 | TAK-733 | 504.23 | 1035555-63-5 | MEK | 101 | 200.3054162 | <1 |  | C17H15F2IN4O4 |
| S2637 | TAK-875 | 533.63 | 1374598-80-7 | GPR | 100 | 187.3957611 | <1 |  | C58H66O15S2 |
| S2718 | TAK-901 | 504.64 | 934541-31-8 | Aurora Kinase | 101 | 200.142676 | <1 |  | C28H32N4O3S |
| S2225 | TAME | 342.41 | 901-47-3 | APC | 69 | 201.5128063 | 69 | 201.5128063 | C14H22N4O4S |
| S4260 | Tamibarotene | 351.44 | 94497-51-5 | Others | 70 | 199.1805145 | <1 |  | C22H25NO3 |
| S1972 | Tamoxifen Citrate | 563.64 | 54965-24-1 | Estrogen/progestogen Receptor | 100 | 177.4182102 | <1 |  | C32H37NO8 |
| S2363 | Tangeretin | 372.37 | 481-53-8 | Others | 8 | 21.48400784 | <1 |  | C20H20O7 |
| S2364 | Tanshinone I | 276.29 | 568-73-0 | Phospholipase (e.g. PLA) | 23 | 83.25 | <1 |  | C18H12O3 |
| S2365 | Tanshinone IIA | 294.34 | 568-72-9 | Others | 5 | 16.98715771 | <1 |  | C19H18O3 |
| S7434 | TAPI-1 | 499.6 | 171235-71-5 | Others | 99 | 198.1585268 | 60 | 120.0960769 | C26H37N5O5 |
| S8028 | Tariquidar | 646.73 | 206873-63-4 | P-gp | 52 | 80.40449647 | <1 |  | C38H38N4O6 |
| S7326 | Tasisulam | 415.11 | 519055-62-0 | Caspase | 83 | 199.947002 | <1 |  | C11H6BrCl2NO3S2 |
| S7617 | *Tasquinimod* | *406.36* | 254964-60-8 | HDAC | 81 | 199.3306428 | <1 |  | C20H17F3N2O4 |
| S2366 | Taxifolin (Dihydroquercetin) | 304.25 | 480-18-2 | Others | 60 | 197.2062449 | <1 |  | C15H12O7 |
| S1569 | Tazarotene | 351.46 | 118292-40-3 | Others | 10 | 28.45274 | <1 |  | C21H21NO2S |
| S7140 | TCID | 283.92 | 30675-13-9 | DUB | 23 | 81.00873485 | <1 |  | C9H2Cl4O2 |
| S8023 | TCS 359 | 360.43 | 301305-73-7 | FLT3 | 15 | 41.61695752 | <1 |  | C18H20N2O4S |
| S2926 | *TDZD-8* | *222.26* | 327036-89-5 | GSK-3 | 44.5 | 200.2159633 | <1 |  | C10H10N2O2S |
| S2159 | Tebipenem Pivoxil | 497.63 | 161715-24-8 | Others | 99 | 198.9429898 | <1 |  | C22H31N3O6S2 |
| S1538 | Telaprevir (VX-950) | 679.85 | 402957-28-2 | HCV Protease | 136 | 200.0441274 | <1 |  | C36H53N7O6 |
| S2231 | Telatinib | 409.83 | 332012-40-5 | VEGFR, PDGFR, c-Kit | 82 | 200.0829612 | <1 |  | C20H16ClN5O3 |
| S1651 | Telbivudine | 242.23 | 3424-98-4 | Others | 48 | 198.1587747 | 48 | 198.1587747 | C10H14N2O5 |
| S1738 | Telmisartan | 514.62 | 144701-48-4 | Others | 13 | 25.2613579 | <1 |  | C33H30N4O2 |
| S2173 | Telotristat Etiprate (LX 1606 Hippurate) | 754.15 | 11137608-69-5 | Hydroxylase | 100 | 132.5996155 | <1 |  | C36H35ClF3N7O6 |
| S2099 | Temocapril HCl | 513.07 | 110221-44-8 | RAAS | 103 | 200.752334 | <1 |  | C23H29ClN2O5S2 |
| S1237 | Temozolomide | 194.15 | 85622-93-1 | Others | 38 | 195.7249549 | <1 |  | C6H6N6O2 |
| S2910 | Tempol | 172.24 | 2226-96-2 | Others | 34 | 197.3989782 | 34 | 197.3989782 | C9H18NO2 * |
| S1044 | Temsirolimus (CCI-779, NSC 683864) | 1030.29 | 162635-04-3 | mTOR | 75 | 72.79503829 | <1 |  | C56H87NO16 |
| S4212 | *Tenatoprazole* | *346.4* | 113712-98-4 | Proton Pump | 46 | 132.7944573 | <1 |  | C16H18N4O3S |
| S1787 | Teniposide | 656.65 | 29767-20-2 | Others | 40 | 60.91525166 | <1 |  | C32H32O13S |
| S7856 | *Tenofovir Alafenamide (GS-7340)* | *476.47* | 379270-37-8 | Reverse Transcriptase | 95 | 199.3829622 | <1 |  | C21H29N6O5P |
| S1400 | Tenofovir Disoproxil Fumarate | 635.51 | 202138-50-9 | Reverse Transcriptase | 128 | 201.4130383 | <1 |  | C23H34N5O14P |
| S8000 | Tenovin-1 | 369.48 | 380315-80-0 | p53 | 74 | 200.2814767 | <1 |  | C20H23N3O2S |
| S4900 | Tenovin-6 | 454.63 | 1011557-82-6 | p53 | 98 | 215.5599059 | 98 | 215.5599059 | C25H35ClN4O2S |
| S2512 | Tenoxicam | 337.37832 | 59804-37-4 | Others | 68 | 201.5541485 | <1 |  | C13H11N3O4S2 |
| S2059 | Terazosin HCl | 459.92 | 70024-40-7 | Adrenergic Receptor | 26 | 56.53157071 | <1 |  | C19H30ClN5O6 |
| S1725 | Terbinafine | 291.43 | 91161-71-6 | Others | 58 | 199.0186323 | <1 |  | C21H25N |
| S2557 | Terbinafine HCl | 327.89 | 78628-80-5 | Others | 66 | 201.287017 | <1 |  | C21H26ClN |
| S4169 | Teriflunomide | 270.21 | 108605-62-5 | Others | 32 | 118.4264091 | <1 |  | C12H9F3N2O2 |
| S2573 | Tetracaine HCl | 300.82 | 136-47-0 | Calcium Channel | 60 | 199.4548235 | 60 | 199.4548235 | C15H25ClN2O2 |
| S2574 | Tetracycline HCl | 480.9 | 64-75-5 | Others | 96 | 199.6257018 | 96 | 199.6257018 | C22H25ClN2O8 |
| S2367 | Tetrahydropapaverine HCl | 379.88 | 6429-04-5 | Others | 7 | 18.42687164 | 76 | 200.0631778 | C20H26ClNO4 |
| S4043 | Tetrahydrozoline HCl | 236.74 | 522-48-5 | Adrenergic Receptor | 12 | 50.68851905 | 47 | 198.5300329 | C13H17ClN2 |
| S7320 | TG003 | 249.33 | 300801-52-9 | CDK | 6 | 24.06449284 | <1 |  | C13H15NO2S |
| S1352 | TG100-115 | 346.34 | 677297-51-7 | PI3K | 9 | 25.98602529 | <1 |  | C18H14N6O2 |
| S2692 | TG101209 | 509.67 | 936091-14-4 | Flt, JAK, c-RET | 102 | 200.1294956 | <1 |  | C26H35N7O2S |
| S2736 | TG101348 (SAR302503) | 524.68 | 936091-26-8 | JAK | 100 | 190.5923611 | <1 |  | C27H36N6O3S |
| S1169 | TGX-221 | 364.44 | 663619-89-4 | PI3K | 12 | 32.92723082 | <1 |  | C21H24N4O2 |
| S7631 | *TH287* | *269.13* | 1609960-30-6 | Others | 53 | 196.9308513 | <1 |  | C11H10Cl2N4 |
| S7632 | TH588 | 295.17 | 1609960-31-7 | others | 20 | 67.76 | <1 |  | C13H12Cl2N4 |
| S1193 | Thalidomide | 258.23 | 50-35-1 | Others | 52 | 201.3708709 | <1 |  | C13H10N2O4 |
| S1739 | Thiabendazole | 201.25 | 148-79-8 | Others | 40 | 198.757764 | <1 |  | C10H7N3S |
| S7213 | Thiamet G | 248.3 | 1009816-48-1 | Others | 50 | 201.3693113 | 50 | 201.3693113 | C9H16N2O4S |
| S2583 | Thiamphenicol | 356.22 | 15318-45-3 | Others | 71 | 199.31503 | <1 |  | C12H15Cl2NO5S |
| S1459 | Thiazovivin | 311.36 | 1226056-71-8 | ROCK | 15 | 48.17574512 | <1 |  | C15H13N5OS |
| S1436 | Tianeptine sodium | 458.93 | 30123-17-2 | 5-HT Receptor | 91 | 198.2873205 | 91 | 198.2873205 | C21H24ClN2NaO4S |
| S7127 | TIC10 Analogue | 386.49 | 41276-02-2 | Akt | 11 | 28.46127972 | <1 |  | C24H26N4O |
| S4079 | Ticagrelor | 522.57 | 274693-27-5 | P2 Receptor | 105 | 200.9300189 | <1 |  | C23H28F2N6O4S |
| S1577 | Tie2 kinase inhibitor | 439.53 | 948557-43-5 | Tie-2 | 35 | 79.63051441 | <1 |  | C26H21N3O2S |
| S1403 | Tigecycline | 585.65 | 220620-09-7 | Others | 100 | 170.7504482 | 100 | 170.7504482 | C29H39N5O8 |
| S4122 | Tilmicosin | 869.13 | 108050-54-0 | Others | 100 | 115.0575863 | 8 | 9.204606906 | C46H80N2O13 |
| S4123 | Timolol Maleate | 432.49 | 26921-17-5 | Adrenergic Receptor | 86 | 198.8485283 | <1 |  | C17H28N4O7S |
| S4068 | Tinidazole | 247.27 | 19387-91-8 | Others | 49 | 198.1639503 | <1 |  | C8H13N3O4S |
| S1910 | Tioconazole | 387.71 | 65899-73-2 | Others | 78 | 201.1812953 | <1 |  | C16H13Cl3N2OS |
| S2062 | Tiopronin | 163.19 | 1953-02-2 | Others | 32 | 196.0904467 | 32 | 196.0904467 | C5H9NO3S |
| S2547 | Tiotropium Bromide hydrate | 490.43 | 139404-48-1 | AChR | 8 | 16.31221581 | <1 |  | C19H24BrNO5S2 |
| S2603 | Tioxolone | 168.17 | 4991-65-5 | Carbonic Anhydrase | 34 | 202.1763692 | <1 |  | C7H4O3S |
| S1453 | Tipifarnib | 489.4 | 192185-72-1 | Farnesyltransferase, Ras | 14 | 28.60645689 | <1 |  | C27H22Cl2N4O |
| S4185 | Tiratricol | 621.93 | 51-24-1 | Others | 100 | 160.7897995 | <1 |  | C14H9I3O4 |
| S2753 | *Tivantinib (ARQ 197)* | *369.42* | 905854-02-6 | c-Met | 73 | 197.6070597 | <1 |  | C23H19N3O2 |
| S1207 | Tivozanib (AV-951) | 454.86 | 475108-18-0 | VEGFR, c-Kit, PDGFR | 20 | 43.96957306 | <1 |  | C22H19ClN4O5 |
| S1437 | Tizanidine HCl | 290.17 | 64461-82-1 | Adrenergic Receptor | 10 | 34.46255643 | 15 | 51.69383465 | C9H9Cl2N5S |
| S7324 | TMP269 | 514.52 | 1314890-29-3 | HDAC | 100 | 194.3559045 | <1 |  | C25H21F3N4O3S |
| S5001 | Tofacitinib (CP-690550) Citrate | 504.49 | 540737-29-9 | JAK | 100 | 198.2199845 | <1 |  | C22H28N6O8 |
| S2789 | Tofacitinib (CP-690550,Tasocitinib) | 312.37 | 477600-75-2 | JAK | 62 | 198.4825687 | <1 |  | C16H20N6O |
| S4124 | Tolazoline HCl | 196.68 | 59-97-2 | Adrenergic Receptor | 39 | 198.2916412 | 39 | 198.2916412 | C10H13ClN2 |
| S2443 | Tolbutamide | 270.35 | 64-77-7 | Potassium Channel | 54 | 199.7410764 | <1 |  | C12H18N2O3S |
| S4021 | Tolcapone | 273.24 | 134308-13-7 | Transferase | 55 | 201.2882448 | <1 |  | C14H11NO5 |
| S1959 | Tolfenamic Acid | 261.7 | 13710-19-5 | Others | 52 | 198.7008024 | <1 |  | C14H12ClNO2 |
| S2058 | Tolnaftate | 307.41 | 2398-96-1 | Others | 62 | 201.685046 | <1 |  | C19H17NOS |
| S4200 | Tolperisone HCl | 281.82 | 3644-61-9 | Others | 56 | 198.7083954 | 56 | 198.7083954 | C16H24ClNO |
| S2550 | Tolterodine tartrate | 475.57 | 124937-52-6 | AChR | 95 | 199.7602877 | 17 | 35.74657779 | C26H37NO7 |
| S4044 | Toltrazuril | 425.38 | 69004-03-1 | Others | 85 | 199.8213362 | <1 |  | C18H14F3N3O4S |
| S2593 | Tolvaptan | 448.94 | 150683-30-0 | Estrogen/progestogen Receptor | 90 | 200.4722235 | <1 |  | C26H25ClN2O3 |
| S1438 | Topiramate | 339.36 | 97240-79-4 | Carbonic Anhydrase | 68 | 200.3771806 | <1 |  | C12H21NO8S |
| S1231 | Topotecan HCl | 457.91 | 119413-54-6 | Topoisomerase | 92 | 200.9128431 | 92 | 200.9128431 | C23H24ClN3O5 |
| S2792 | Torcetrapib | 600.47 | 262352-17-0 | CETP | 120 | 199.843456 | <1 |  | C26H25F9N2O4 |
| S1776 | Toremifene Citrate | 598.08 | 89778-27-8 | Others | 100 | 167.2017121 | <1 |  | C32H36ClNO8 |
| S2817 | Torin 2 | 432.4 | 1223001-51-1 | mTOR | 20 | 46.25346901 | <1 |  | C24H15F3N4O |
| S2824 | TPCA-1 | 279.29 | 507475-17-4 | IKK | 56 | 200.5084321 | <1 |  | C12H10FN3O2S |
| S2673 | Trametinib (GSK1120212) | 615.39 | 871700-17-3 | MEK | 22 | 35.74968719 | <1 |  | C26H23FIN5O4 |
| S1439 | Tranilast | 327.33 | 53902-12-8 | Others | 66 | 201.6313812 | <1 |  | C18H17NO5 |
| S4246 | Tranylcypromine (2-PCPA) HCl | 169.65 | 4548-34-9 | MAO | 33 | 194.5181256 | 33 | 194.5181256 | C9H12ClNR |
| S7513 | Trelagliptin | 357.38 | 865759-25-7 | DPP-4 | 71 | 198.6680844 | <1 |  | C18H20FN5O2 |
| S1653 | Tretinoin | 300.4 | 302-79-4 | Others | 60 | 199.7336884 | <1 |  | C20H28O2 |
| S1933 | Triamcinolone | 394.43 | 124-94-7 | Others | 79 | 200.2890247 | <1 |  | C21H27FO6 |
| S1628 | Triamcinolone Acetonide | 434.5 | 76-25-5 | Others | 87 | 200.2301496 | <1 |  | C24H31FO6 |
| S4080 | Triamterene | 253.26 | 396-01-0 | Sodium Channel | 20 | 78.97022822 | <1 |  | C12H11N7 |
| S7470 | Triapine | 195.24 | 236392-56-6 | DNA/RNA Synthesis | 20 | 102.438025 | <1 |  | C7H9N5S |
| S1667 | Trichlormethiazide | 380.66 | 133-67-5 | Others | 54 | 141.8588767 | <1 |  | C8H8Cl3N3O4S2 |
| S1045 | Trichostatin A (TSA) | 302.4 | 58880-19-6 | HDAC | 23 | 76.05820106 | <1 |  | C17H22N2O3 |
| S1117 | Triciribine | 320.3 | 35943-35-2 | Akt | 64 | 199.8126756 | <1 |  | C13H16N6O4 |
| S4114 | Triclabendazole | 359.66 | 68786-66-3 | Others | 72 | 200.1890675 | <1 |  | C14H9Cl3N2OS |
| S3201 | Trifluoperazine 2HCl | 480.42 | 440-17-5 | Others | 88 | 183.1730569 | 96 | 199.825153 | C21H26Cl2F3N3S |
| S1778 | Trifluridine | 296.2 | 70-00-8 | NULL | 59 | 199.1897367 | 59 | 199.1897367 | C10H11F3N2O5 |
| S3200 | Triflusal | 248.16 | 322-79-2 | COX | 50 | 201.4829142 | <1 |  | C10H7F3O4 |
| S1404 | Trilostane | 329.43 | 13647-35-3 | Dehydrogenase | 65 | 197.310506 | <1 |  | C20H27NO3 |
| S2085 | Trimebutine | 387.47 | 39133-31-8 | Opioid Receptor | 50 | 129.0422484 | <1 |  | C22H29NO5 |
| S3129 | Trimethoprim | 290.32 | 738-70-5 | Others | 58 | 199.7795536 | <1 |  | C14H18N4O3 |
| S3146 | Tripelennamine HCl | 291.82 | 154-69-8 | Histamine Receptor | 2 | 6.853539853 | 58 | 198.7526557 | C16H22ClN3 |
| S3604 | Triptolide (PG490) | 360.4 | 38748-32-2 | Others | 72 | 199.7780244 | <1 |  | C20H24O6 |
| S4176 | Trometamol | 121.14 | 77-86-1 | Others | 2 | 16.50982334 | 24 | 198.1178801 | C4H11NO3 |
| S1913 | Tropicamide | 284.35 | 1508-75-4 | AChR | 57 | 200.457183 | <1 |  | C17H20N2O2 |
| S1898 | Tropisetron | 320.81 | 105826-92-4 | Others | 35 | 109.0988436 | 46 | 143.3870515 | C17H21ClN2O2 |
| S2549 | Trospium chloride | 427.96 | 10405-02-4 | AChR | 41 | 95.80334611 | 86 | 200.9533601 | C25H30ClNO3 |
| S2369 | Troxerutin | 742.68 | 7085-55-4 | Others | 100 | 134.6474929 | 100 | 134.6474929 | C33H42O19 |
| S4128 | Troxipide | 294.35 | 30751-05-4 | Others | 18 | 61.15169016 | <1 |  | C15H22N2O4 |
| S1470 | TSU-68 (SU6668, Orantinib) | 310.35 | 252916-29-3 | VEGFR, PDGFR , FGFR | 62 | 199.7744482 | <1 |  | C18H18N2O3 |
| S4627 | TTNPB (Arotinoid Acid) | 348.48 | 71441-28-6 | Others | 15 | 43.04407713 | <1 |  | C24H28O2 |
| S8049 | Tubastatin A | 335.4 | 1252003-15-8 | HDAC | 9 | 26.83363148 | <1 |  | C20H21N3O2 |
| S2627 | Tubastatin A HCl | 371.86 | 1310693-92-5 | HDAC | 74 | 198.9996235 | <1 |  | C20H22ClN3O2 |
| S1121 | TW-37 | 573.7 | 877877-35-5 | Bcl-2 | 115 | 200.4531985 | <1 |  | C33H35NO6S |
| S1590 | TWS119 | 318.33 | 601514-19-6 | GSK-3 | 64 | 201.0492256 | <1 |  | C18H14N4O2 |
| S3162 | Tylosin tartrate | 1066.19 | 74610-55-2 | Others | 300 | 281.3757398 | 200 | 187.5838265 | C50H83NO23 |
| S2895 | Tyrphostin 9 | 282.38 | 10537-47-0 | EGFR | 56 | 198.3143282 | <1 |  | C18H22N2O |
| S8024 | Tyrphostin AG 1296 | 266.29 | 146535-11-7 | PDGFR | 6 | 22.5318262 | <1 |  | C16H14N2O2 |
| S2816 | Tyrphostin AG 879 | 316.46 | 148741-30-4 | HER2 | 36 | 113.7584529 | <1 |  | C18H24N2OS |
| S1102 | U0126-EtOH | 426.56 | 1173097-76-1 | MEK | 85 | 199.2685671 | <1 |  | C20H22N6OS2 |
| S2866 | U-104 | 309.32 | 178606-66-1 | Carbonic Anhydrase | 62 | 200.4396741 | <1 |  | C13H12FN3O3S |
| S2224 | UK 383367 | 324.38 | 348622-88-8 | Procollagen C Proteinase | 65 | 200.3822677 | <1 |  | C15H24N4O4 |
| S3081 | Ulipristal | 475.62 | 159811-51-5 | Estrogen/progestogen Receptor | 83 | 174.5090619 | <1 |  | C28H35NO3 |
| S7854 | *Ulixertinib (BVD-523, VRT752271)* | *433.33* | 869886-67-9 | ERK | 86 | 198.4630651 | <1 |  | C21H22Cl2N4O2 |
| S7608 | *UM171* | *453.54* | 1448724-09-1 | Others | 45 | 99.21947348 | <1 |  | C25H27N9 |
| S7510 | *UM729* | *367.44* | 1448723-60-1 | Others | 48 | 130.6335728 | <1 |  | C20H25N5O2 |
| S7531 | *UMI-77* | *468.34* | 518303-20-3 | Bcl-2 | 93 | 198.5736858 | <1 |  | C18H14BrNO5S2 |
| S7570 | *UNC0379* | *413.56* | 1620401-82-2 | Histone Methyltransferase | 82 | 198.2783635 | <1 |  | C23H35N5O2 |
| S7610 | *UNC0631* | *635.93* | 1320288-19-4 | Histone Methyltransferase | 100 | 157.2500118 | <1 |  | C37H61N7O2 |
| S7088 | UNC1215 | 529.72 | 1415800-43-9 | Epigenetic Reader Domain | 100 | 188.7789776 | <1 |  | C32H43N5O2 |
| S7165 | UNC1999 | 569.74 | 1431612-23-5 | Histone Methyltransferase | 100 | 175.5186576 | <1 |  | C33H43N7O2 |
| S7325 | UNC2881 | 463.58 | 1493764-08-1 | Others | 92 | 198.4554985 | <1 |  | C25H33N7O2 |
| S7373 | UNC669 | 338.24 | 1314241-44-5 | Epigenetic Reader Domain | 11 | 32.52128666 | <1 |  | C15H20BrN3O |
| S8038 | UPF 1069 | 279.29 | 1048371-03-4 | PARP | 56 | 200.5084321 | <1 |  | C17H13NO3 |
| S4177 | Uracil | 112.09 | 66-22-8 | Others | 22 | 196.2708538 | <1 |  | C4H4N2O2 |
| S2025 | Urapidil HCl | 423.94 | 64887-14-5 | 5-HT Receptor | 24 | 56.61178469 | 85 | 200.5000708 | C20H30ClN5O3 |
| S2631 | URB597 | 338.4 | 546141-08-6 | FAAH | 68 | 200.9456265 | <1 |  | C20H22N2O3 |
| S2029 | Uridine | 244.2 | 58-96-8 | DNA/RNA Synthesis | 49 | 200.6552007 | 49 | 200.6552007 | C9H12N2O6 |
| S7343 | *URMC-099* | *421.54* | 1229582-33-5 | Others | 84 | 199.2693457 | <1 |  | C27H27N5 |
| S1643 | Ursodiol | 392.57 | 128-13-2 | Others | 79 | 201.2379958 | <1 |  | C24H40O4 |
| S2370 | Ursolic Acid | 456.7 | 77-52-1 | Others | 91 | 199.2555288 | <1 |  | C30H48O3 |
| S1876 | Valaciclovir HCl | 360.8 | 124832-27-5 | Others | 14 | 38.80266075 | 72 | 199.556541 | C13H21ClN6O4 |
| S4049 | Valdecoxib | 314.36 | 181695-72-7 | COX | 63 | 200.4071765 | <1 |  | C16H14N2O3S |
| S4050 | Valganciclovir HCl | 390.82 | 175865-59-5 | Others | 78 | 199.5803695 | 78 | 199.5803695 | C14H25ClN6O5 |
| S4216 | Valnemulin HCl | 601.28 | 133868-46-9 | Others | 100 | 166.311868 | 100 | 166.311868 | C31H53ClN2O5S |
| S1168 | Valproic acid sodium salt (Sodium valproate) | 166.19 | 1069-66-5 | GABA Receptor, HDAC | 33 | 198.5679042 | 33 | 198.5679042 | C8H15NaO2 |
| S1894 | Valsartan | 435.52 | 137862-53-4 | RAAS | 87 | 199.761205 | <1 |  | C24H29N5O3 |
| S1046 | Vandetanib (ZD6474) | 475.35 | 443913-73-3 | VEGFR |  | 10 |  |  | C22H24BrFN4O2 |
| S3071 | Vanillin | 152.15 | 121-33-5 | Others | 30 | 197.1738416 | <1 |  | C8H8O3 |
| S2371 | Vanillylacetone | 194.23 | 122-48-5 | Others | 38 | 195.6443392 | 10 | 51.48535242 | C11H14O3 |
| S2515 | Vardenafil HCl Trihydrate | 579.11 | 224785-90-4 | PDE | 116 | 200.3073682 | 116 | 200.3073682 | C23H39ClN6O7S |
| S1110 | Varespladib (LY315920) | 380.39 | 172732-68-2 | Phospholipase | 76 | 199.7949473 | <1 |  | C21H20N2O5 |
| S2755 | Varlitinib | 466.94 | 845272-21-1 | EGFR | 6 | 12.84961665 | <1 |  | C22H19ClN6O2S |
| S1101 | Vatalanib (PTK787) 2HCl | 419.73 | 212141-51-0 | VEGFR, c-Kit, Flt | 85 | 202.5111381 | 10 | 23.82483978 | C20H17Cl3N4 |
| S8007 | VE-821 | 368.41 | 1232410-49-9 | ATM/ATR | 74 | 200.8631688 | <1 |  | C18H16N4O3S |
| S7102 | VE-822 | 463.55 | 1232416-25-9 | ATM/ATR | 36 | 77.66152519 | <1 |  | C24H25N5O3S |
| S1405 | Vecuronium Bromide | 637.73 | 50700-72-6 | Others | 100 | 156.8061719 | 4 | 6.272246876 | C34H57BrN2O4 |
| S1004 | Veliparib (ABT-888) | 244.29 | 912444-00-9 | PARP | 17 | 69.58942241 | <1 |  | C13H16N4O |
| S1267 | Vemurafenib (PLX4032, RG7204) | 489.92 | 918504-65-1 | Raf | 97 | 197.9915088 | <1 |  | C23H18ClF2N3O3S |
| S1441 | Venlafaxine | 313.86 | 99300-78-4 | 5-HT Receptor | 55.5 | 176.830434 | 55.5 | 176.830434 | C17H28ClNO2 |
| S7751 | *VER155008* | *556.4* | 1134156-31-2 | HSP (e.g. HSP90) | 100 | 179.7268152 | <1 |  | C25H23Cl2N7O4 |
| S7458 | VER-49009 | 387.82 | 940289-57-6 | HSP | 77 | 198.5457171 | <1 |  | C19H18ClN3O4 |
| S7459 | VER-50589 | 388.8 | 747413-08-7 | HSP (e.g. HSP90) | 77 | 198.0452675 | <1 |  | C19H17ClN2O5 |
| S4202 | Verapamil HCl | 491.06 | 152-11-4 | Others | 98 | 199.5682809 | 50 | 101.8205515 | C27H39ClN2O4 |
| S7707 | *Verdinexor (KPT-335)* | *442.32* | 1392136-43-4 | CRM1 | 88 | 198.9509857 | <1 |  | C18H12F6N6O |
| S7515 | VGX-1027 | 205.21 | 6501-72-0 | Others | 41 | 199.7953316 | <1 |  | C11H11NO3 |
| S1784 | Vidarabine | 267.24 | 5536-17-4 | 5-alpha Reductase | 53 | 198.3236043 | 3 | 11.22586439 | C10H13N5O4 |
| S7262 | *Vidofludimus* | *355.36* | 717824-30-1 | Dehydrogenase | 100 | 281.4047726 | <1 |  | C20H18FNO4 |
| S4259 | Vilazodone HCl | 477.99 | 163521-08-2 | 5-HT Receptor | 96 | 200.84 | <1 |  | C26H28ClN5O2 |
| S3033 | Vildagliptin (LAF-237) | 303.4 | 274901-16-5 | DPP-4 | 60 | 197.7587343 | 60 | 197.7587343 | C17H25N3O2 |
| S1241 | Vincristine | 923.04 | 2068-78-2 | Autophagy,Microtubule Associated | 100 | 108.3376668 | 60 | 65.0026001 | C46H58N4O14S |
| S4269 | Vinorelbine Tartrate | 1079.11 | 125317-39-7 | Microtubule Associated | 100 | 92.66895868 | 100 | 92.66895868 | C53H66N4O20 |
| S1082 | Vismodegib (GDC-0449) | 421.3 | 879085-55-9 | Hedgehog, P-gp | 84 | 199.3828626 | <1 |  | C19H14Cl2N2O3S |
| S1902 | Vitamin B12 | 1355.37 | 68-19-9 | Others | 75 | 55.33544346 | 6 | 4.426835477 | C63H88CoN14O14P |
| S3114 | Vitamin C | 176.12 | 50-81-7 | Others | 35 | 198.7281399 | 35 | 198.7281399 | C6H8O6 |
| S4035 | Vitamin D2 | 396.65 | 50-14-6 | Others | 20 | 50.42228665 | <1 |  | C28H44O |
| S4063 | Vitamin D3 | 384.64 | 67-97-0 | Others | 77 | 200.187188 | <1 |  | C27H44O |
| S4101 | Voglibose | 267.28 | 83480-29-9 | Others | 74 | 276.8632146 | 74 | 276.8632146 | C10H21NO7 |
| S2235 | Volasertib (BI 6727) | 618.81 | 755038-65-4 | PLK | 20 | 32.32009825 | <1 |  | C34H50N8O3 |
| S7518 | Voreloxin (SNS-595) | 401.44 | 175414-77-4 | Topoisomerase | 20 | 49.82064568 | 1 | 2.491032284 | C18H19N5O4S |
| S1442 | Voriconazole | 349.31 | 137234-62-9 | P450 | 70 | 200.3950646 | <1 |  | C16H14F3N5O |
| S1047 | Vorinostat (SAHA, MK0683) | 264.3 | 149647-78-9 | HDAC | 53 | 200.5297011 | <1 |  | C14H20N2O3 |
| S8021 | Vortioxetine (Lu AA21004) HBr | 379.36 | 960203-27-4 | 5-HT Receptor | 76 | 200.3374104 | <1 |  | C18H23BrN2S |
| S7016 | VS-5584 (SB2343) | 354.41 | 1246560-33-7 | PI3K | 71 | 200.3329477 | <1 |  | C17H22N8O |
| S2795 | VU 0357121 | 305.32 | 433967-28-3 | GluR | 61 | 199.7903839 | <1 |  | C17H17F2NO2 |
| S2892 | VU 0361737 | 262.69 | 1161205-04-4 | GluR | 53 | 201.758727 | <1 |  | C13H11ClN2O2 |
| S2862 | VU 0364770 | 232.67 | 61350-00-3 | GluR | 47 | 202.0028366 | <1 |  | C12H9ClN2O |
| S2865 | VUF 10166 | 262.74 | 155584-74-0 | 5-HT Receptor | 21 | 79.92692396 | <1 |  | C13H15ClN4 |
| S1480 | VX-222 (VCH-222, Lomibuvir) | 445.61 | 1026785-55-6 | HCV Protease | 89 | 199.726218 | <1 |  | C25H35NO4S |
| S7059 | VX-661 | 520.5 | 1152311-62-0 | CFTR | 100 | 192.1229587 | <1 |  | C26H27F3N2O6 |
| S1048 | VX-680 (Tozasertib, MK-0457) | 464.59 | 639089-54-6 | Aurora Kinase | 93 | 200.1764997 | <1 |  | C23H28N8OS |
| S6005 | VX-702 | 404.3 | 745833-23-2 | p38 MAPK | 81 | 200.3462775 | <1 |  | C19H12F4N4O2 |
| S1458 | VX-745 | 436.26 | 209410-46-8 | p38 MAPK | 15 | 34.383166 | <1 |  | C19H9Cl2F2N3OS |
| S2228 | VX-765 | 509 | 273404-37-8 | Caspase | 100 | 196.4636542 | <1 |  | C24H33ClN4O6 |
| S1565 | VX-809 (Lumacaftor) | 452.41 | 936727-05-8 | Others | 90 | 198.9345947 | <1 |  | C24H18F2N2O5 |
| S2663 | WAY-100635 Maleate | 538.64 | 1092679-51-0 | 5-HT Receptor | 85 | 157.8048418 | <1 |  | C29H38N4O6 |
| S2689 | WAY-600 | 494.59 | 1062159-35-6 | mTOR | 22 | 44.48128753 | <1 |  | C28H30N8O |
| S7565 | WH-4-023 | 568.67 | 837422-57-8 | Src | 12 | 21.10186927 | <1 |  | C32H36N6O4 |
| S2867 | WHI-P154 | 376.2 | 211555-04-3 | JAK, EGFR | 75 | 199.3620415 | <1 |  | C16H14BrN3O3 |
| S7490 | WIKI4 | 521.59 | 838818-26-1 | Wnt/beta-catenin | 7 | 13.42050269 | <1 |  | C29H23N5O3S |
| S7037 | Wnt-C59 (C59) | 379.45 | 1243243-89-1 | Wnt/beta-catenin | 76 | 200.2898933 | <1 |  | C25H21N3O |
| S2796 | WP1066 | 356.22 | 857064-38-1 | JAK | 71 | 199.31503 | <1 |  | C17H14BrN3O |
| S7441 | *WS3* | *569.58* | 1421227-52-2 | IκB/IKK | 100 | 175.5679624 | <1 |  | C28H30F3N7O3 |
| S7442 | *WS6* | *568.59* | 1421227-53-3 | IκB/IKK | 100 | 175.8736524 | <1 |  | C29H31F3N6O3 |
| S8029 | WY-14643 (Pirinixic Acid) | 323.8 | 50892-23-4 | PPAR | 65 | 200.7411983 | <1 |  | C14H14ClN3O2S |
| S2661 | WYE-125132 (WYE-132) | 519.6 | 1144068-46-1 | mTOR | 104 | 200.1539646 | <1 |  | C27H33N7O4 |
| S1266 | WYE-354 | 495.53 | 1062169-56-5 | mTOR | 99 | 199.7860876 | <1 |  | C24H29N7O5 |
| S1170 | WZ3146 | 464.95 | 1214265-56-1 | EGFR | 93 | 200.0215077 | <1 |  | C24H25ClN6O2 |
| S1173 | WZ4002 | 494.18 | 1213269-23-8 | EGFR | 13 | 26.30620422 | <1 |  | C25H27ClN6O3 |
| S7317 | WZ4003 | 496.99 | 1214265-58-3 | AMPK | 7 | 14.08479044 | <1 |  | C25H29ClN6O3 |
| S1179 | WZ8040 | 481.01 | 1214265-57-2 | EGFR | 96 | 199.5800503 | <1 |  | C24H25ClN6OS |
| S2912 | WZ811 | 290.36 | 55778-02-4 | CXCR | 30 | 103.3200165 | <1 |  | C18H18N4 |
| S7889 | *Xanthohumol* | *354.4* | 6754-58-1 | COX | 70 | 197.51693 | <1 |  | C21H22O5 |
| S2372 | Xanthone | 196.2 | 90-47-1 | Others | 39 | 198.7767584 | <1 |  | C13H8O2 |
| S1180 | XAV-939 | 312.31 | 284028-89-3 | Wnt/beta-catenin | 12 | 38.4233614 | <1 |  | C14H11F3N2OS |
| S7457 | *XEN445* | *366.33* | 1515856-92-4 | Others | 80 | 218.3823329 | <1 |  | C18H17F3N2O3R |
| S7036 | XL019 | 444.53 | 945755-56-6 | JAK | 16 | 35.99307133 | <1 |  | C25H28N6O2 |
| S2694 | XL335 | 438.47 | 629664-81-9 | FXR | 33 | 75.26170548 | <1 |  | C25H24F2N2O3 |
| S7035 | *XL388* | *455.5* | 1251156-08-7 | mTOR | 23 | 50.49396268 | <1 |  | C23H22FN3O4S |
| S7122 | XL888 | 503.64 | 1149705-71-4 | HSP (e.g. HSP90) | 100 | 198.5545231 | <1 |  | C29H37N5O3 |
| S7525 | XMD8-92 | 474.55 | 1234480-50-2 | ERK | 73 | 153.8299442 | <1 |  | C26H30N6O3 |
| S2516 | Xylazine HCl | 256.79 | 23076-35-9 | Adrenergic Receptor | 50 | 194.7116321 | 12 | 46.7307917 | C12H17ClN2S |
| S2576 | Xylometazoline HCl | 280.84 | 1218-35-5 | Others | 27 | 96.14015098 | 56 | 199.4017946 | C16H25ClN2 |
| S2124 | Xylose | 150.13 | 25990-60-7 | Others | 30 | 199.8268168 | 30 | 199.8268168 | C5H10O5 |
| S1049 | Y-27632 2HCl | 320.26 | 129830-38-2 | ROCK | 64 | 199.8376319 | 14 | 43.71448198 | C14H23Cl2N3O |
| S7516 | Y-320 | 505.01 | 288250-47-5 | Others | 11 | 21.7817469 | <1 |  | C27H29ClN6O2 |
| S7489 | YH239-EE | 504.41 | 1364488-67-4 | Mdm2 | 100 | 198.2514225 | <1 |  | C25H27Cl2N3O4 |
| S7679 | *YK-4-279* | *366.20* | 1037184-44-3 | DNA/RNA Synthesis | 73 | 199.3446204 | <1 |  | C17H13Cl2NO4 |
| S1130 | YM155 (Sepantronium Bromide) | 443.29 | 781661-94-7 | Survivin | 55 | 124.0722777 | 89 | 200.771504 | C20H19BrN4O3 |
| S1219 | YM201636 | 467.48 | 371942-69-7 | PI3K | 35 | 74.86951313 | <1 |  | C25H21N7O3 |
| S2711 | YO-01027 | 463.48 | 209984-56-5 | Gamma-secretase | 92 | 198.4983171 | <1 |  | C26H23F2N3O3 |
| S2373 | Yohimbine HCl | 390.9 | 65-19-0 | NULL | 12 | 30.69838833 | <1 |  | C21H27ClN2O3 |
| S1633 | Zafirlukast | 575.68 | 107753-78-6 | Others | 100 | 173.7076153 | <1 |  | C31H33N3O6S |
| S1719 | Zalcitabine | 211.22 | 7481-89-2 | Others | 42 | 198.8448064 | 42 | 198.8448064 | C9H13N3O3 |
| S3008 | Zaltoprofen | 298.36 | 74711-43-6 | COX | 60 | 201.0993431 | <1 |  | C17H14O3S |
| S3007 | Zanamivir | 332.31 | 139110-80-8 | Others | 2 | 6.018476724 | 25 | 75.23095904 | C12H20N4O7 |
| S7293 | ZCL278 | 584.89 | 587841-73-4 | Rho | 100 | 170.9723196 | <1 |  | C21H19BrClN5O4S2 |
| S7312 | Z-DEVD-FMK | 668.66 | 210344-95-9 | Caspase | 100 | 149.55 | <1 |  | C31H42FN3O12 |
| S7113 | Zebularine | 228.2 | 3690-10-6 | DNA Methyltransferase | 46 | 201.5775635 | 46 | 201.5775635 | C9H12N2O5 |
| S7391 | Z-FA-FMK | 386.42 | 197855-65-5 | Cysteine Protease | 77 | 199.2650484 | <1 |  | C21H23FN2O4 |
| S1456 | Zibotentan (ZD4054) | 424.43 | 186497-07-4 | ETA Receptor | 24 | 56.54642697 | <1 |  | C19H16N6O4S |
| S2579 | Zidovudine | 267.24 | 30516-87-1 | Others | 53 | 198.3236043 | 53 | 198.3236043 | C10H13N5O4 |
| S1443 | Zileuton | 236.29 | 111406-87-2 | Others | 47 | 198.9081214 | <1 |  | C11H12N2O2S |
| S4075 | Zinc Pyrithione | 317.7 | 13463-41-7 | Proton Pump | 30 | 94.42870633 | <1 |  | C10H8N2O2S2Zn |
| S1444 | Ziprasidone HCl | 449.4 | 122883-93-6 | Others | 90 | 200.2670227 | <1 |  | C21H22Cl2N4OS |
| S7447 | ZLN005 | 250.34 | 49671-76-3 | Others | 37 | 147.7989934 | <1 |  | C17H18N2 |
| S2897 | ZM 306416 | 333.74 | 690206-97-4 | VEGFR | 67 | 200.7550788 | <1 |  | C16H13ClFN3O2 |
| S2896 | ZM 323881 HCl | 411.86 | 193000-39-4 | VEGFR | 10 | 24.28009518 | <1 |  | C22H19ClFN3O2 |
| S2720 | ZM 336372 | 389.45 | 208260-29-1 | Raf | 78 | 200.2824496 | <1 |  | C23H23N3O3 |
| S8004 | ZM 39923 HCl | 367.91 | 1021868-92-7 | JAK | 30 | 81.54168139 | <1 |  | C23H26ClNO |
| S1103 | ZM 447439 | 513.59 | 331771-20-1 | Aurora Kinase | 103 | 200.5490761 | <1 |  | C29H31N5O4 |
| S1649 | Zolmitriptan | 287.36 | 139264-17-8 | Others | 58 | 201.8374165 | <1 |  | C16H21N3O2 |
| S1445 | Zonisamide | 212.23 | 68291-97-4 | Others | 42 | 197.8985063 | <1 |  | C8H8N2O3S |
| S1481 | Zosuquidar (LY335979) 3HCl | 636.99 | 167465-36-3 | P-gp | 100 | 156.9883358 | 23 | 36.10731723 | C32H34Cl3F2N3O2 |
| S7091 | Zotarolimus(ABT-578) | 966.21 | 221877-54-9 | mTOR | 100 | 103.4971694 | <1 |  | C52H79N5O12 |
| S4233 | Zoxazolamine | 168.58 | 61-80-3 | Others | 34 | 201.6846601 | <1 |  | C7H5ClN2O |
| S1072 | ZSTK474 | 417.41 | 475110-96-4 | PI3K | 21 | 50.31024652 | <1 |  | C19H21F2N7O2 |
| S7023 | Z-VAD-FMK | 467.49 | 187389-52-2 | Caspase | 93 | 198.9347366 | <1 |  | C22H30FN3O7 |

**Table S3.** The 181 distinct mechanisms of actions (MoAs) in the HTS customized library.

| **Table S3. MoA distribution for all HTS library members.** | |
| --- | --- |
| Others | 781 |
| Adrenergic Receptor | 45 |
| VEGFR | 44 |
| 5-HT Receptor | 42 |
| PI3K | 41 |
| EGFR | 38 |
| HDAC | 38 |
| AChR | 31 |
| DNA/RNA Synthesis | 30 |
| mTOR | 30 |
| Histamine Receptor | 29 |
| CDK | 27 |
| PDGFR | 24 |
| COX | 23 |
| Estrogen/progestogen Receptor | 23 |
| JAK | 23 |
| Methyltransferase | 23 |
| Aurora Kinase | 21 |
| HSP | 20 |
| MEK | 19 |
| RAAS | 19 |
| Sodium Channel | 19 |
| c-Kit | 18 |
| c-Met | 18 |
| Histone Methyltransferase | 18 |
| PDE | 18 |
| Bcr-Abl | 17 |
| Microtubule Associated | 17 |
| Akt | 16 |
| Dopamine Receptor | 16 |
| P450 | 16 |
| Topoisomerase | 16 |
| FGFR | 15 |
| Raf | 15 |
| TGF-beta/Smad | 15 |
| FLT | 14 |
| GSK-3 | 14 |
| PARP | 14 |
| Proteasome | 14 |
| Androgen Receptor | 13 |
| Calcium Channel | 13 |
| Src | 13 |
| IKK | 12 |
| p38 MAPK | 12 |
| Epigenetic Reader Domain | 11 |
| ATPase | 10 |
| Dehydrogenase | 10 |
| HER2 | 10 |
| Opioid Receptor | 10 |
| Farnesyltransferase | 9 |
| GluR | 9 |
| IGF-1 | 9 |
| PKC | 9 |
| PLK | 9 |
| PPAR | 9 |
| Wnt/beta-catenin | 9 |
| Bcl-2 | 8 |
| Cysteine Protease | 8 |
| FAK | 8 |
| GABA Receptor | 8 |
| IGF-1R | 8 |
| IκB | 8 |
| Rho | 8 |
| Sirtuin | 8 |
| 5-alpha Reductase | 7 |
| ALK | 7 |
| ATM | 7 |
| Caspase | 7 |
| DPP-4 | 7 |
| DUB | 7 |
| Gamma-secretase | 7 |
| Histone Demethylase | 7 |
| HIV Protease | 7 |
| p53 | 7 |
| Potassium Channel | 7 |
| Reverse Transcriptase | 7 |
| STAT | 7 |
| BTK | 6 |
| Cannabinoid Receptor | 6 |
| Carbonic Anhydrase | 6 |
| HCV Protease | 6 |
| Integrase | 6 |
| Kinesin | 6 |
| Mdm2 | 6 |
| MMP | 6 |
| AMPK | 5 |
| Aromatase | 5 |
| ATR | 5 |
| GPR | 5 |
| HMG-CoA Reductase | 5 |
| NF-κB | 5 |
| P2 Receptor | 5 |
| PDK-1 | 5 |
| Proton Pump | 5 |
| ROCK | 5 |
| Smoothened | 5 |
| Syk | 5 |
| Transferase | 5 |
| CETP | 4 |
| CFTR | 4 |
| Chk | 4 |
| CRM1 | 4 |
| DNA-PK | 4 |
| ERK | 4 |
| Factor Xa | 4 |
| FLT3 | 4 |
| Hedgehog | 4 |
| HIF | 4 |
| Histone Acetyltransferase | 4 |
| Hydroxylase | 4 |
| IAP | 4 |
| Integrin | 4 |
| MAO | 4 |
| P-gp | 4 |
| Pim | 4 |
| S1P Receptor | 4 |
| S6 Kinase | 4 |
| Autophagy | 3 |
| c-RET | 3 |
| CSF-1R | 3 |
| DNA Methyltransferase | 3 |
| Dynamin | 3 |
| Endothelin Receptor | 3 |
| FAAH | 3 |
| Ferroptosis | 3 |
| FXR | 3 |
| Histone Demethylases | 3 |
| IDO | 3 |
| LRRK2 | 3 |
| OX Receptor | 3 |
| PAK | 3 |
| Phospholipase | 3 |
| PKA | 3 |
| SGLT | 3 |
| Telomerase | 3 |
| Tie-2 | 3 |
| TNF-alpha | 3 |
| AMPA Receptor-kainate Receptor | 2 |
| NMDA Receptor | 2 |
| Angiogenesis | 2 |
| Antimetabolites | 2 |
| BET | 2 |
| Beta Amyloid | 2 |
| CaSR | 2 |
| CXCR | 2 |
| DHFR | 2 |
| ETA Receptor | 2 |
| Liver X Receptor | 2 |
| LPA Receptor | 2 |
| PERK | 2 |
| Rac | 2 |
| Serine Protease | 2 |
| Trk receptor | 2 |
| TRPV | 2 |
| VDA | 2 |
| APC | 1 |
| Axl | 1 |
| BACE | 1 |
| CaMK | 1 |
| cAMP | 1 |
| Cathepsin K | 1 |
| CCR5 | 1 |
| c-Myc | 1 |
| Diacylglycerol Acyltransferase 1 | 1 |
| E1 Activating | 1 |
| E2 conjugating | 1 |
| gp120/CD4 | 1 |
| IL Receptor | 1 |
| Nexturastat A | 1 |
| NOD1 | 1 |
| p97 | 1 |
| PAFR | 1 |
| PDGF | 1 |
| Phosphorylase | 1 |
| Procollagen C Proteinase | 1 |
| Reuptake inhibitor | 1 |
| Substance P | 1 |
| Survivin | 1 |
| v-Abl | 1 |
| Wee1 | 1 |
| Y-Secretase | 1 |

**Table S4.** The 16 hits identified by the HTS.

| Hits | | | | | | | | | |
| --- | --- | --- | --- | --- | --- | --- | --- | --- | --- |
| Product Name | 1# | 2# | 3# | 4# | 5# | 6# | 7# | 8# | 9# |
| Elesclomol | 17.21995178 | 12.05264509 | 1.799440093 | 38.99406336 | 2.40504684 | 2.586775959 | 2.165720899 | 1.537838214 | 1.494389131 |
| Quisinostat | 19.67474956 | 40.0810765 | 12.80811658 | 105.5792632 | 69.26534901 | 13.43505132 | 30.92239367 | 85.48487743 | 46.41308566 |
| Panobinostat | 15.66221591 | 40.13352492 | 9.879728407 | 37.8744349 | 33.21166135 | 15.78429286 | 26.3090237 | 82.09690155 | 16.09795428 |
| Dacinostat | 14.55755691 | 32.43409802 | 9.770845089 | 55.83064702 | 59.5249093 | 11.61830453 | 22.08010124 | 76.25311634 | 47.27759247 |
| Givinostat | 29.38796044 | 45.34904128 | 11.77985888 | 10.33098459 | 51.85536444 | 18.80476431 | 30.06097695 | 86.74322315 | 33.18090629 |
| CUDC-907 | 12.07400254 | 34.13238583 | 12.73663954 | 23.89 | 45.64976553 | 14.43735149 | 21.19750885 | 73.97561667 | 40.21584004 |
| BGT226 | 3.376847625 | 3.009068497 | 4.247628684 | 4.232342352 | 3.172820583 | 0.958139415 | 3.709180869 | 1.902230143 | 2.006126591 |
| BAY 11-7082 | 50.78412269 | 10.8037773 | 2.529837672 | 18.23574324 | 23.69902721 | 1.153920224 | 3.050111541 | 22.7981997 | 20.22455529 |
| SC144 | 68.47908011 | 50.75945465 | 22.53316514 | 23.42575456 | 20.87633552 | 24.03180008 | 11.4372148 | 39.31033852 | 23.41899541 |
| LDN-212854 | 50.78053576 | 62.19301029 | 3.53721969 | 29.23467352 | 2.39564506 | 1.666204806 | 3.132589502 | 2.954125142 | 22.49388513 |
| UNC0631 | 9.18290647 | 6.629925995 | 2.811229532 | 8.324534732 | 2.821314887 | 1.806490771 | 2.799324045 | 16.36636903 | 46.10173101 |
| SF1670 | 35.96777082 | 2.342815379 | 5.336571315 | 8.173425633 | 2.962380631 | 0.654374306 | 1.85995356 | 16.27252517 | 3.252968016 |
| Bay 11-7085 | 58.7040184 | 5.591358352 | 2.307522545 | 14.23799332 | 7.970214556 | 1.009407175 | 2.104189886 | 9.772273864 | 8.697512671 |
| ZLN005 | 50.67521739 | 23.35569558 | 40.98405087 | 36.23324325 | 31.9513911 | 25.25954436 | 25.0060423 | 29.13539013 | 99.97717108 |
| NSC697923 | 5.227696614 | 1.8671734 | 2.48681098 | 7.233242563 | 2.532539063 | 0.698524322 | 1.582610321 | 4.779248002 | 98.37862556 |
| Zinc Pyrithione | 3.120947242 | 2.154093315 | 2.561070236 | 6.233468853 | 2.849063381 | 0.753459478 | 2.283751858 | 1.959354171 | 2.122761858 |

**Table S5. The 13 MoAs enriched among the HTS screened hits.**

| **HTS librariry** | |
| --- | --- |
| **Product Name** | **MoAs** |
| Elesclomol | HSP inhibitor |
| Quisinostat | HDAC inhibitor |
| Panobinostat | HDAC inhibitor |
| Dacinostat | HDAC inhibitor |
| Givinostat | HDAC inhibitor |
| CUDC-907 | HDAC&PI3K inhibitor |
| BGT226 | PI3K inhibitor |
| BAY 11-7082 | NFκB inhibitor |
| SC144 | gp130 inhibitor |
| LDN-212854 | BMP inhibitor |
| UNC0631 | HDM inhibitor |
| SF1670 | PTEN inhibitor |
| Bay 11-7085 | TNFα inhibitor |
| ZLN005 | PGC1α inhibitor |
| NSC697923 | E2 inhibitor |
| Zinc Pyrithione | Antifungal & Proton pump inhibitor |

**Table S6. The detailed information of targeted antibodies.**

| **Primary antibodies** | **catalog no.** | **dilution** | **Company** |
| --- | --- | --- | --- |
| NF-κB | ab19870 | 1 : 1,000 | Abcam |
| Nrf2 | ab137550 | 1 : 1,000 | Abcam |
| NQO1 | ab2346 | 1 : 2,000 | Abcam |
| PARP1 | ab227244 | 1 : 5,000 | Abcam |
| Bcl-2 | ab194583 | 1 : 1,000 | Abcam |
| tubulin | ab11321 | 1 : 5,000 | Abcam |
| β-actin | ab8227 | 1 : 5,000 | Abcam |
| Bax | 2772 | 1 : 1,000 | Cell Signaling Technology |
| caspase-3 | 9662 | 1 : 5,00 | Cell Signaling Technology |
| Akt | 4060 | 1 : 400 | Cell Signaling Technology |
| mTOR | 2972 | 1 : 500 | Cell Signaling Technology |
| S6K | 9202 | 1 : 400 | Cell Signaling Technology |
| 4EBP1 | 9644 | 1 : 500 | Cell Signaling Technology |
| 8-OHdG | 251640 | 1 : 400 | Abbiotec |
| DAPI | C1002 | 1 : 1,000 | Beyotime |
| Alexa Fluor 488 donkeyanti-rabbit secondary antibody | A-21206 | 1:500 | Invitrogen |

**Supplementary Figures**

**Figure S1**


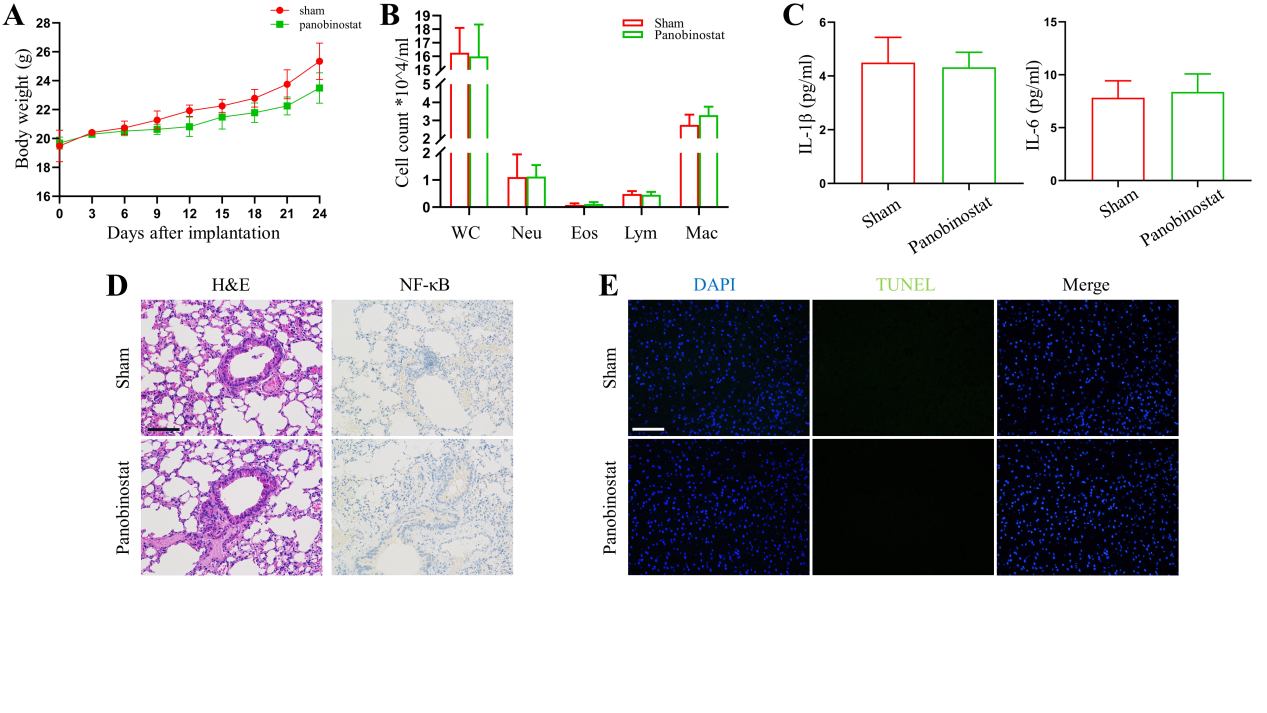


**Figure S1.** **Panobinostat demonstrated safety in GH3 xenograft PitNET model.** (**A**) Whole body weight curves in the sham and Panobinostat groups. (**B**) Immune cell counts from bronchoalveolar lavage fluid in each group. (**C**) The concentrations of inflammatory mediators IL-1β and IL6 in the bronchoalveolar lavage fluid from each group. (**D**) Representative images of H&E and NF-κB staining from mice lungs tissues. (**E**) Representative images of TUNEL staining from brain tissues. Scale bar = 100 μm.

**Figure S2**

**
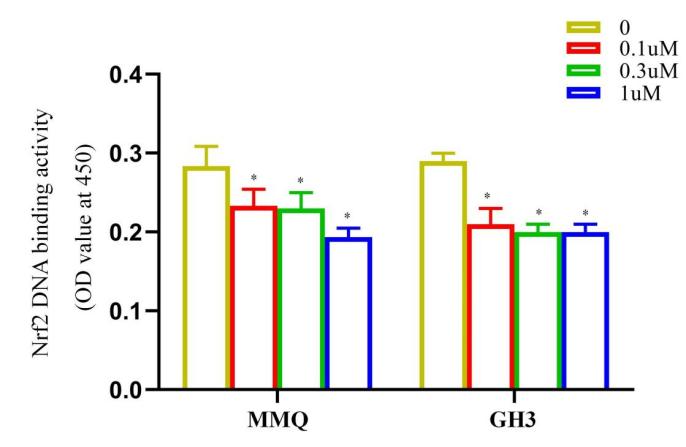
**

**Figure S2.** **The role of Panobinostat on Nrf2 activation in MMQ and GH3 cells.** The effects of Panobinostat on Nrf2 DNA binding activity in MMQ and GH3 cells at different dosage at 24 h. n = 3; ^*^*p* < 0.05.

**Figure S3**


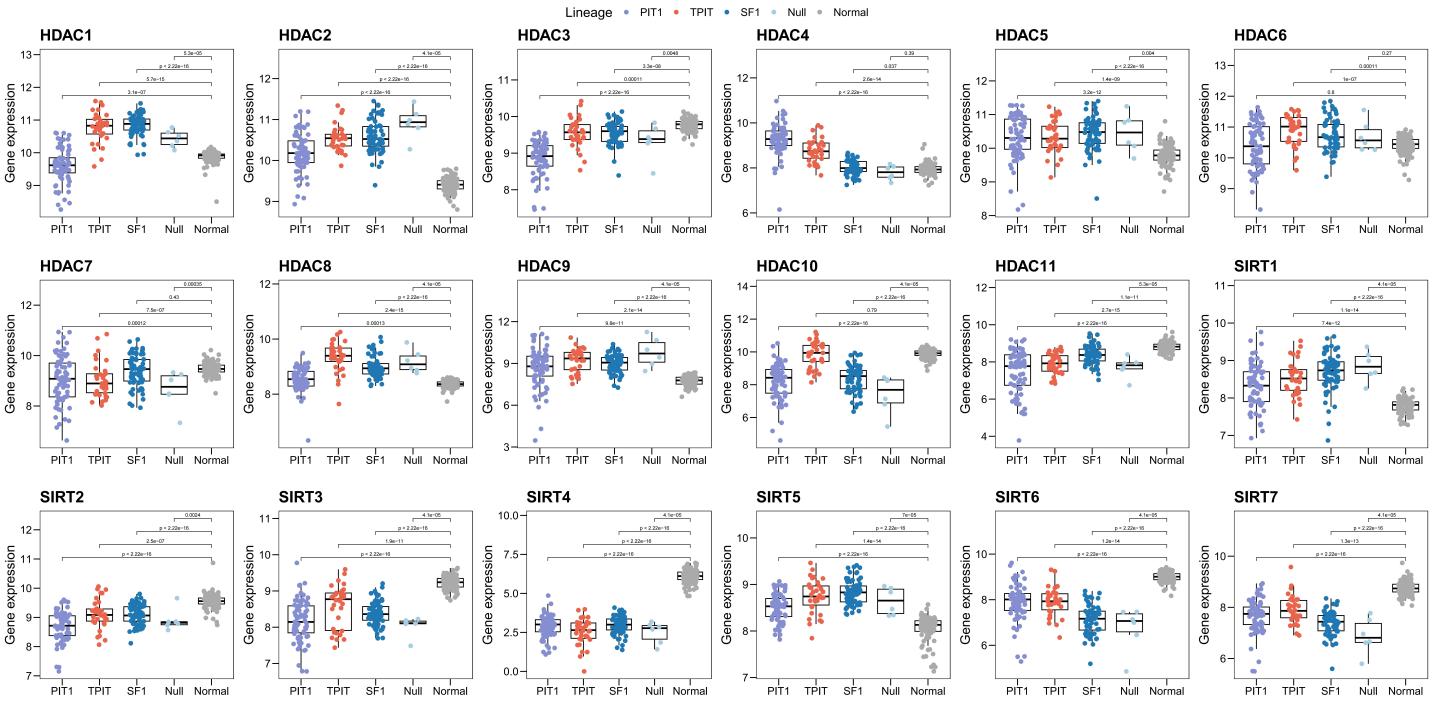


**Figure S3.** **The gene expressions of HDACs in PitNETs by RNA sequencing.**

**Figure S4**


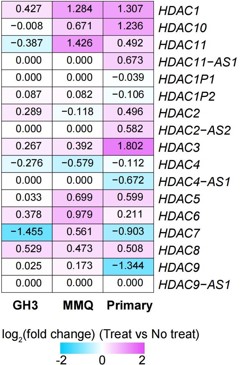


**Figure S4. The gene expressions of HDACs in PitNET cell lines following Panobinostat treatment by RNA sequencing.**

**Figure S5**

**
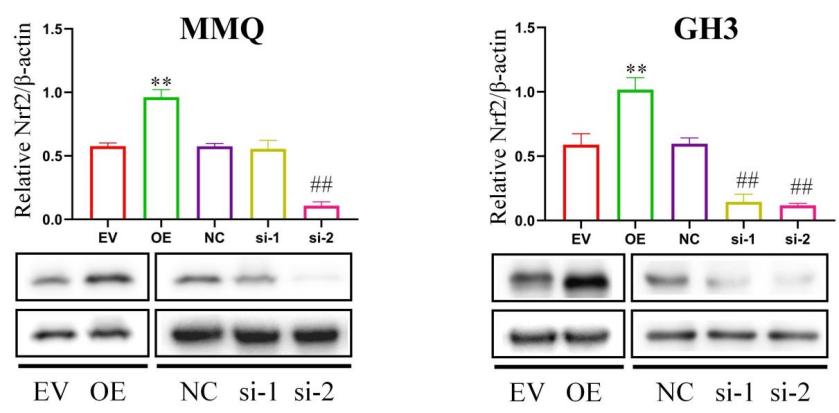
**

**Figure S5. The effect of Nrf2 over-expression and knockdown by RNAi technologies.** n = 3; ^**^*p* < 0.01, ^##^*p* < 0.01.

**Figure S6**

**
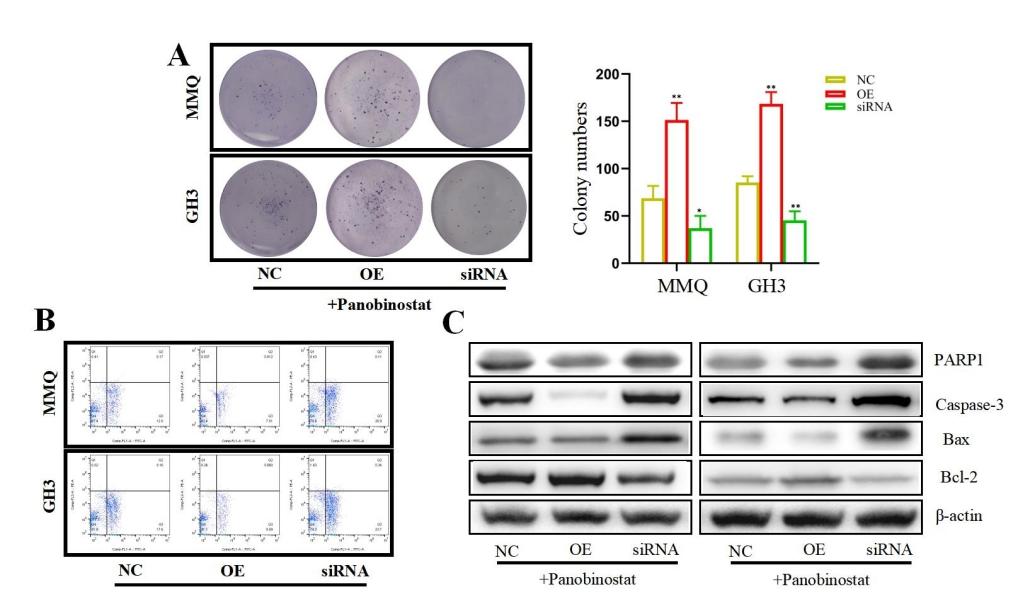
**

**Figure S6. The role of** **Nrf2 in the colony formation and apoptosis mediated by Panobinostat** (**A**)Representation of colony formation for different Nrf2 expressions with Panobinostat administration in MMQ and GH3 cell lines. n = 3; ^*^*p* < 0.05, ^**^*p* < 0.01. (**B**) The presentation of Anneix staining in MMQ and GH3 cell lines. (**E**) Representation of immuno-blots for different apoptosis expressions with Panobinostat administration in MMQ and GH3 cell lines.

**Figure S7**

**
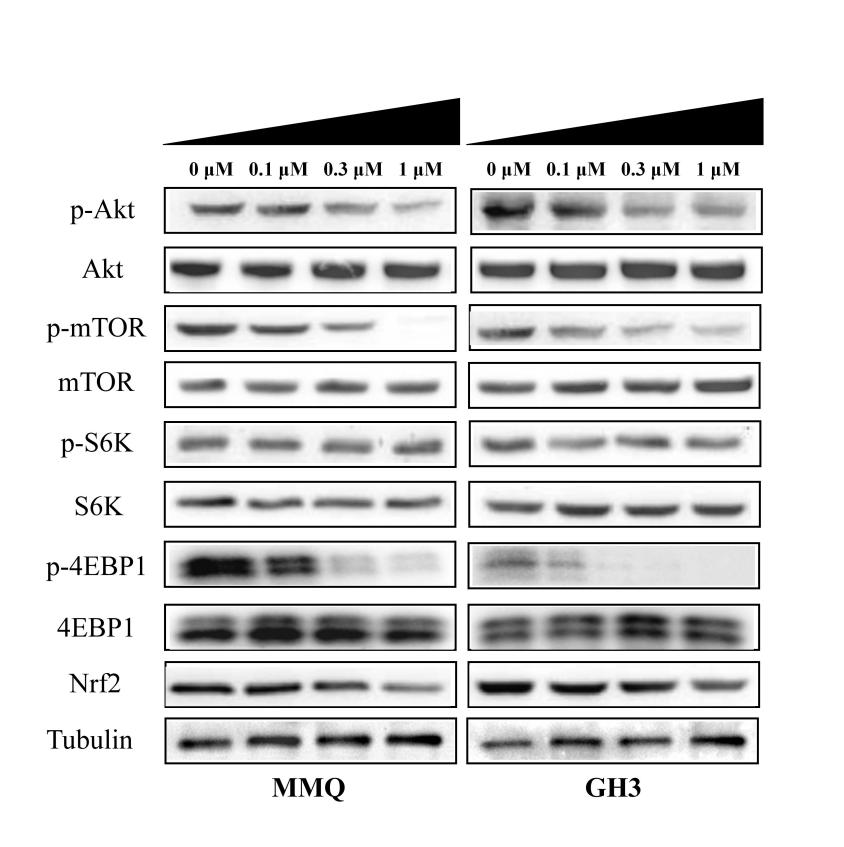
**

**Figure S7.** **The role of Panobinostat on Akt/mTOR/4EBP1/Nrf2 pathway in MMQ and GH3 cells.** Representation of immuno-blots for Akt/mTOR/4EBP1/Nrf2 signaling in different MMQ and GH3 cell lines at different dosage at 24 h.
